# Supplementary material for: Herbivore camping reshapes the taxonomy, function and network of pasture soil microbial communities
Source: PeerJ. 2022 Nov 9;10:e14314. doi: 10.7717/peerj.14314 (PMC9653066; doi:10.7717/peerj.14314)
Supplement: Supplemental Information 1 [file peerj-10-14314-s001.zip › Basic information on the nutrient content of grassland soil.docx]

The grassland soil nutrition information before camping was listed below. For surface soil (0-10 cm), the organic carbon content was 61.05 g/kg, total nitrogen content 3.88 g/kg, total phosphorus content 1.01 g/kg, alkali hydrolyzed nitrogen content 354.04 mg/kg, and available phosphorus content 6.43 mg/kg; for subsurface soil (10-20 cm), the organic carbon content 41.30 g/kg, total nitrogen content was 2.78 g/kg, total phosphorus content 0.82 g/kg, alkali hydrolyzed nitrogen content 259.05 mg/kg, and available phosphorus content 3.50 mg/kg. These nutrient contents were determined using the methods described in our previous works (Ding et al 2020a, Ding et al 2020b), and in a website (https://iforest.sisef.org/pdf/Ding_3091@suppl001.pdf).

Ding L, Shang Y, Zhang W, Zhang Y, Li S, Wei X *et al* (2020a). Disentangling the effects of driving forces on soil bacterial and fungal communities under shrub encroachment on the Guizhou Plateau of China. *Sci Total Environ* **709:** 136207.doi:10.1016/j.scitotenv.2019.136207

Ding L, Wang P, Zhang W, Zhang Y, Li S, Wei X *et al* (2020b). Soil stoichiometry modulates effects of shrub encroachment on soil carbon concentration and stock in a subalpine grassland. *iForest Biogeosci For* **13:** 65-72.doi:10.3832/ifor3091-012
