# Supplementary material for: Herbivore camping reshapes the taxonomy, function and network of pasture soil microbial communities
Source: PeerJ. 2022 Nov 9;10:e14314. doi: 10.7717/peerj.14314 (PMC9653066; doi:10.7717/peerj.14314)

Figure S1. Principal coordinates analysis (PCoA) of subsoil bacterial and fungal communities under the camping and no camping treatments based on Bray-Curtis, jaccard, unweighted unifrac, and weighted unifrac distances.


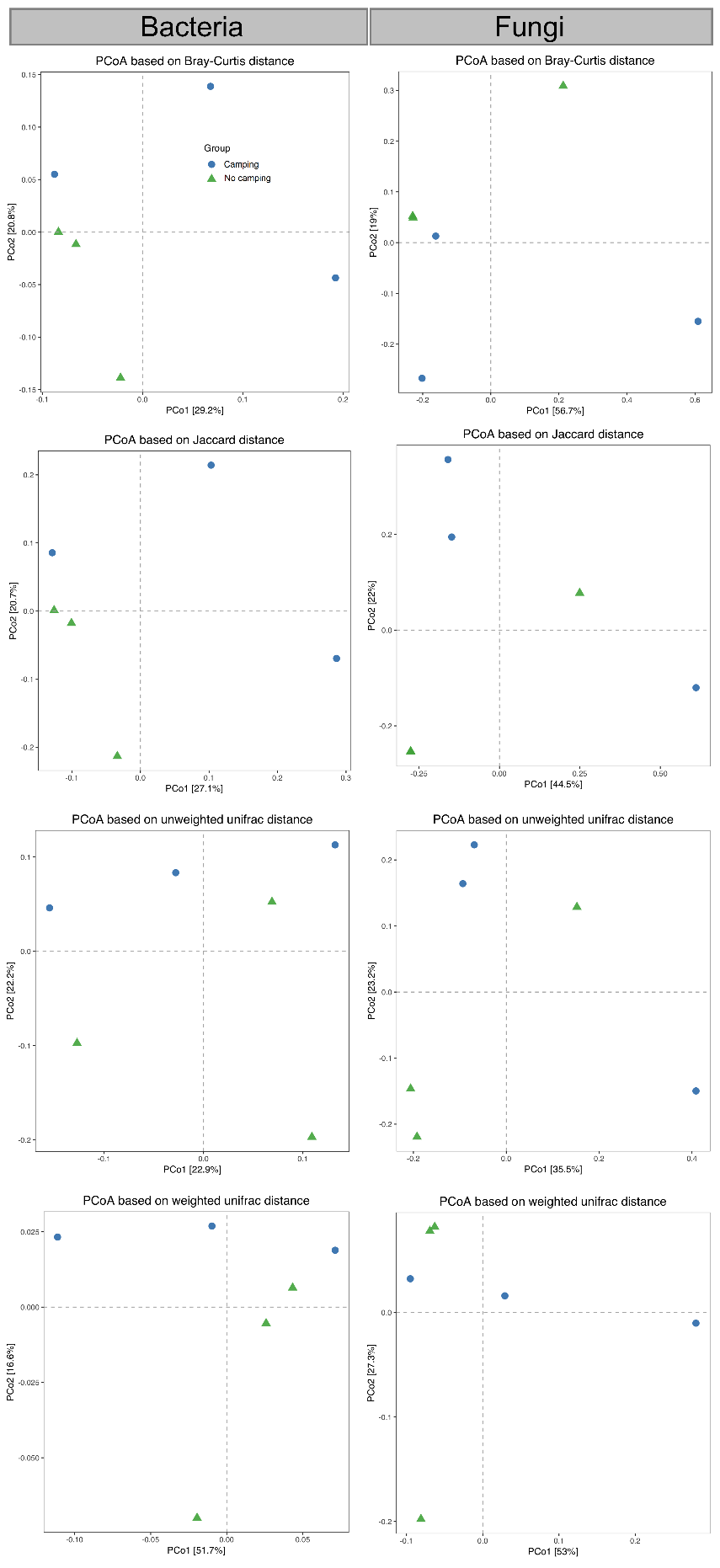


Figure S2. Venn plots showing the unique and overlap OTUs of subsoil bacterial and fungal communities under the camping and no camping treatments. The numbers outside the brackets represent the number of OTUs; the numbers in the brackets represent the percentage of OTUs sequences in the total number of sequences.


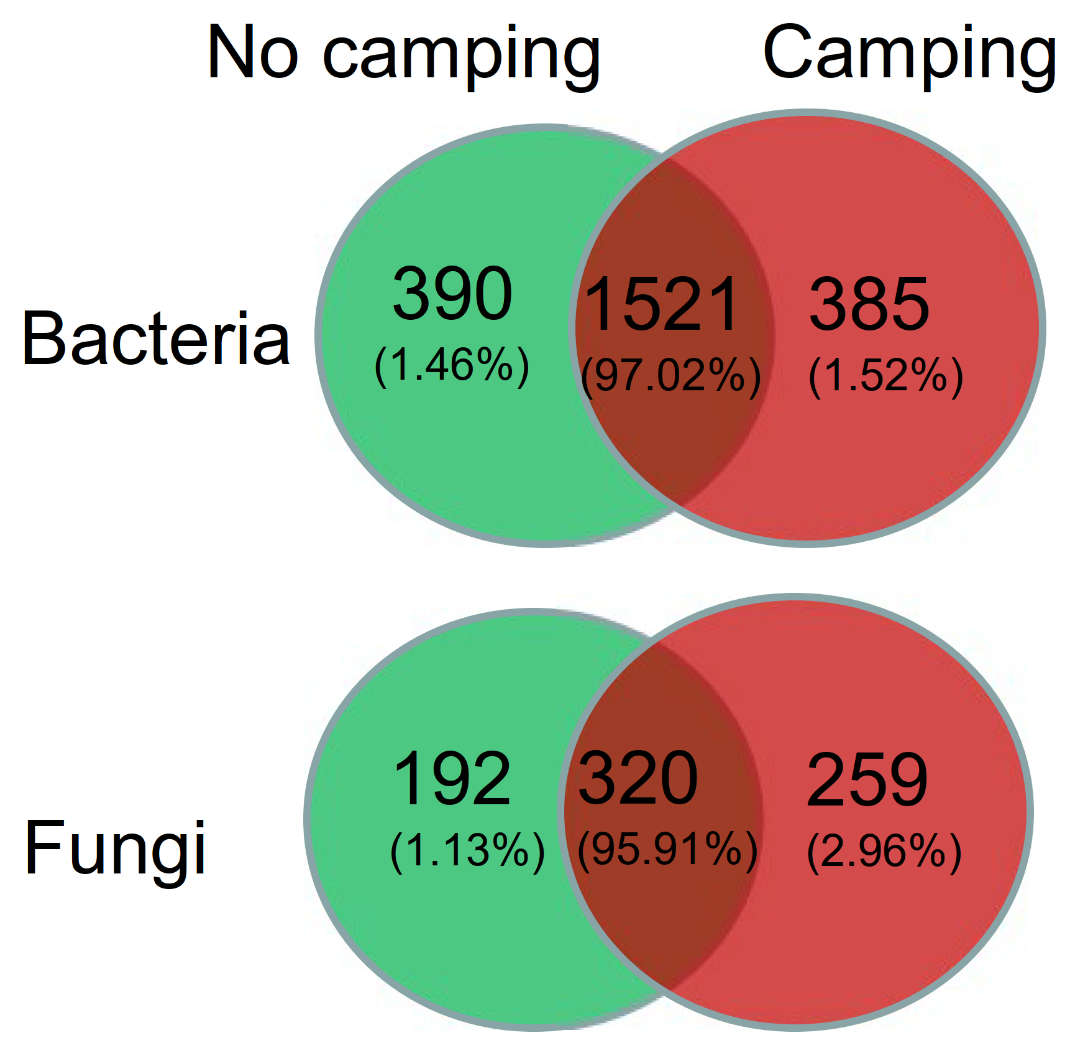


Figure S3. Functional diversities of FAPROTAX1.2.4-based aromatic compound degradation of subsoil bacterial communities under camping and no camping. ns, *p* > 0.05.


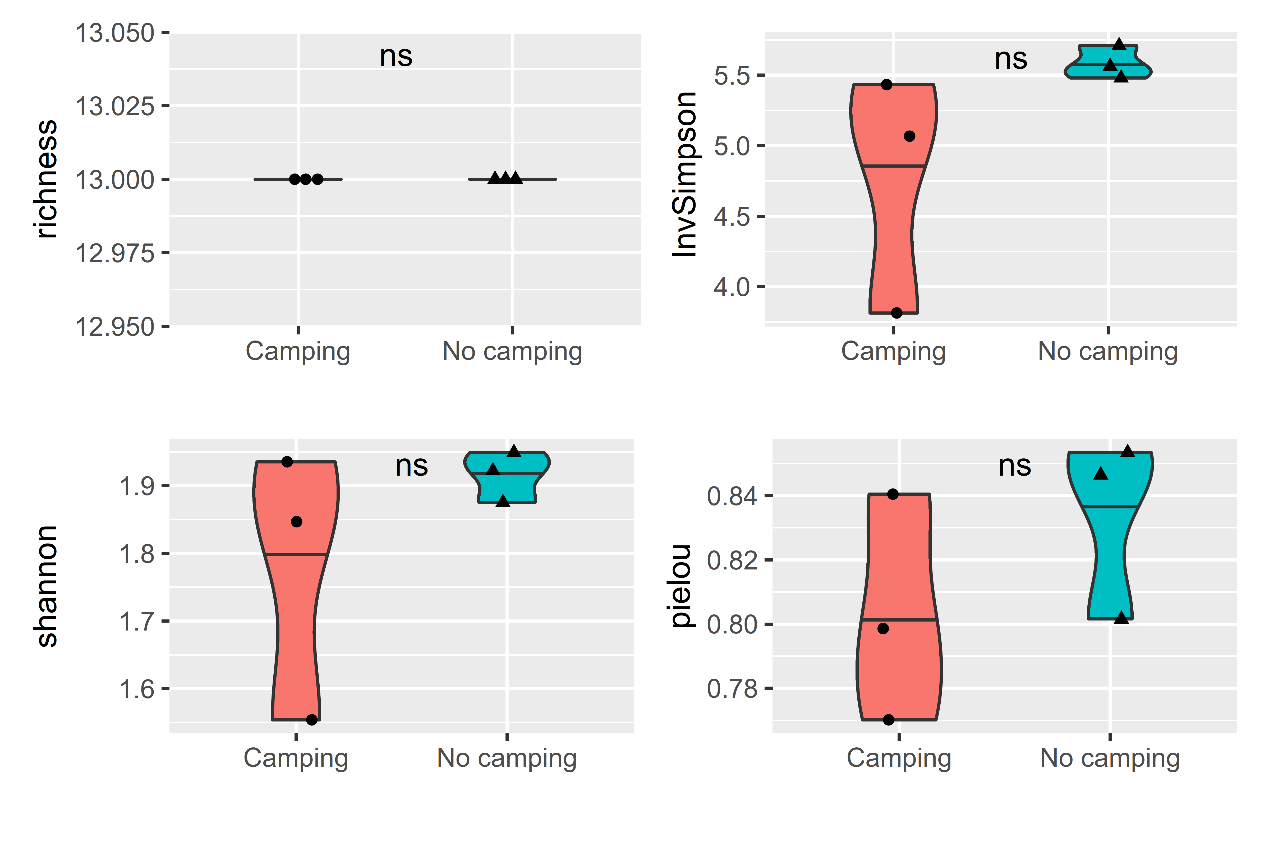


Figure S4. Functional diversities of FAPROTAX1.2.4-based aromatic hydrocarbon degradation of subsoil bacterial communities under camping and no camping. ns, *p* > 0.05.


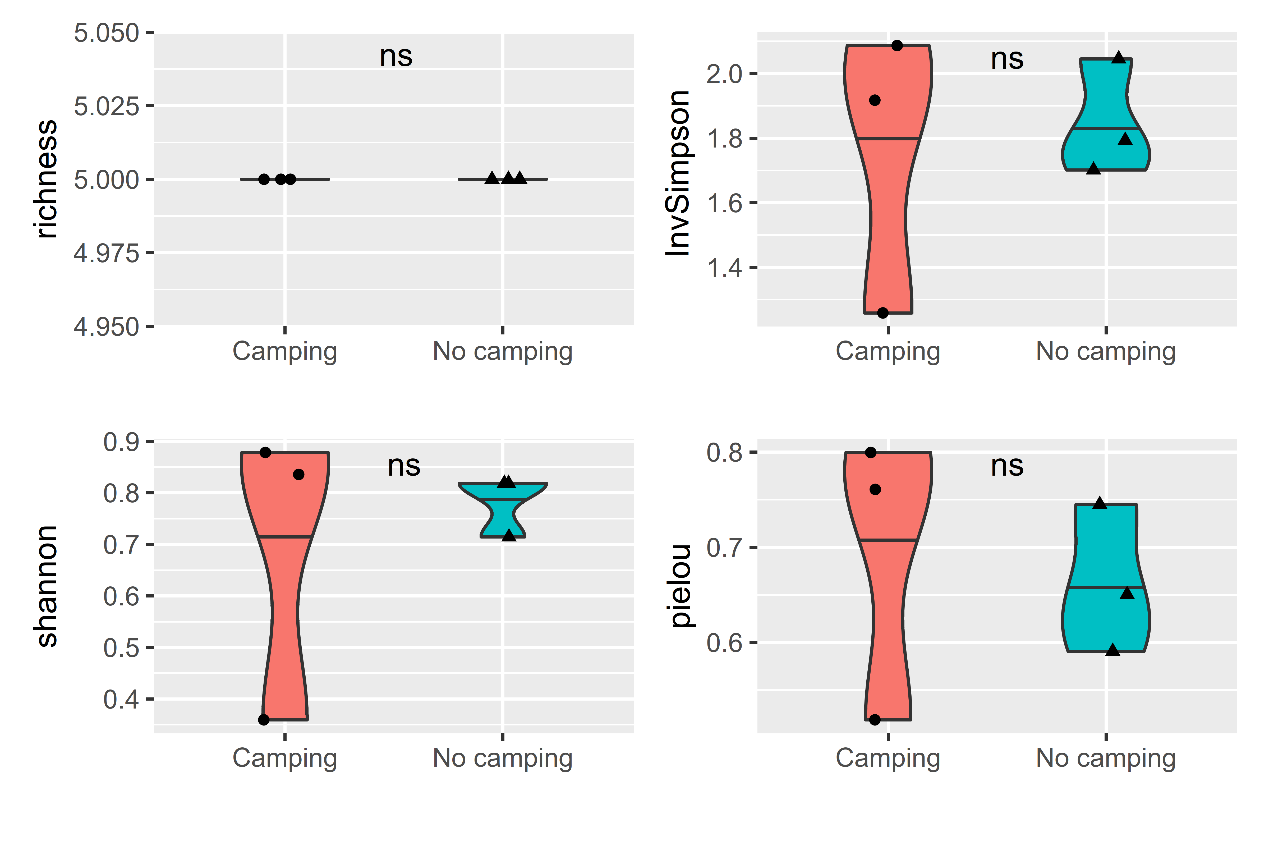


Figure S5. Functional diversities of FAPROTAX1.2.4-based cellulolysis of subsoil bacterial communities under camping and no camping. ns, *p* > 0.05.


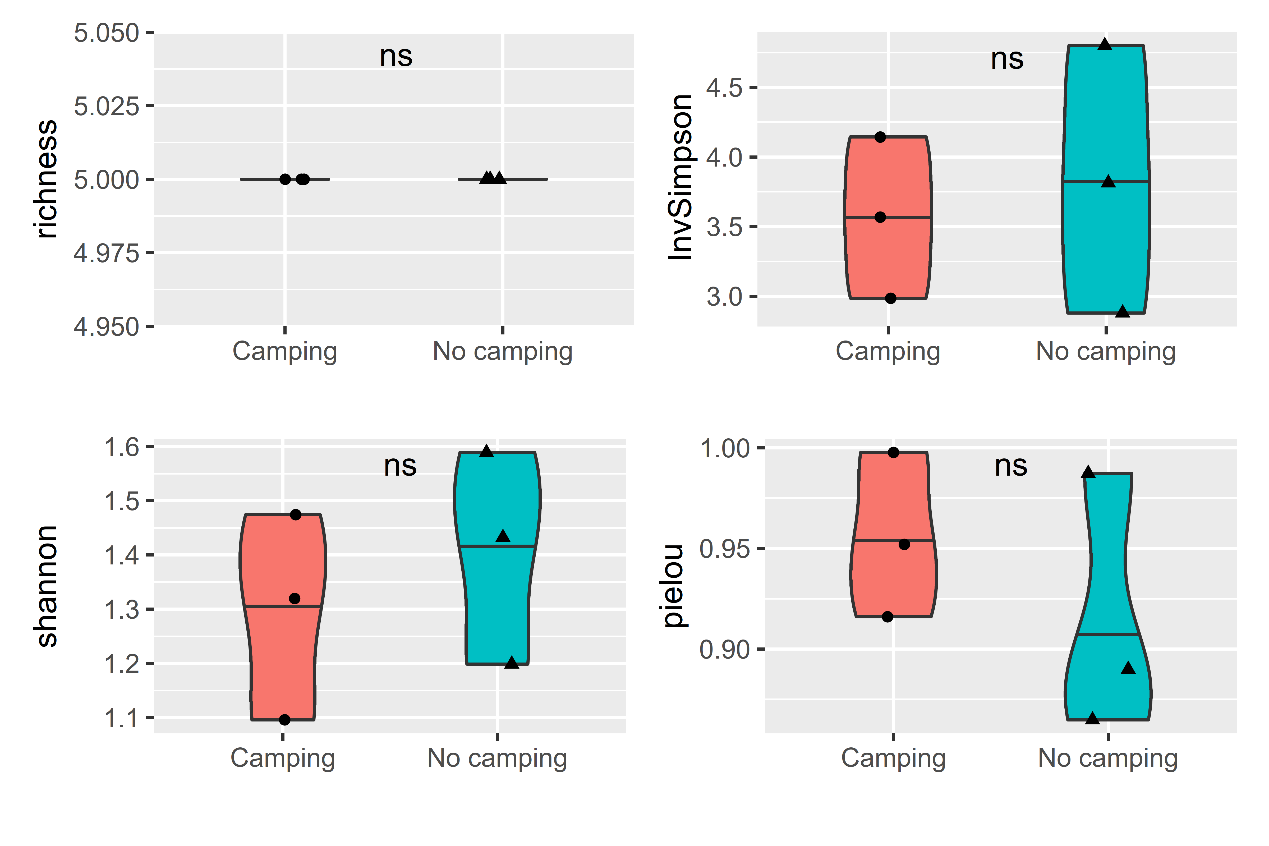


Figure S6. Functional diversities of FAPROTAX1.2.4-based chitinolysis of subsoil bacterial communities under camping and no camping. ns, *p* > 0.05.


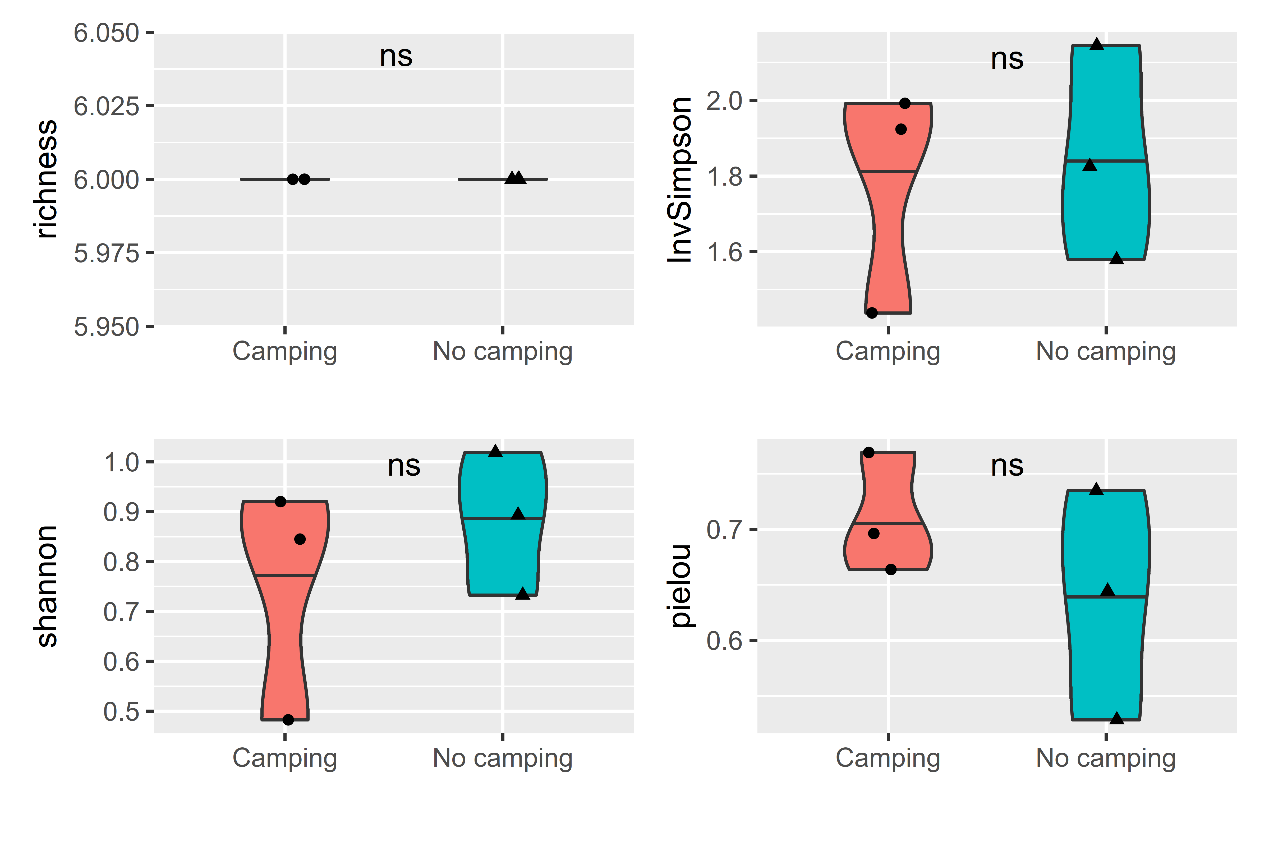


Figure S7. Functional diversities of FAPROTAX1.2.4-based ligninolysis of subsoil bacterial communities under camping and no camping. ns, *p* > 0.05.


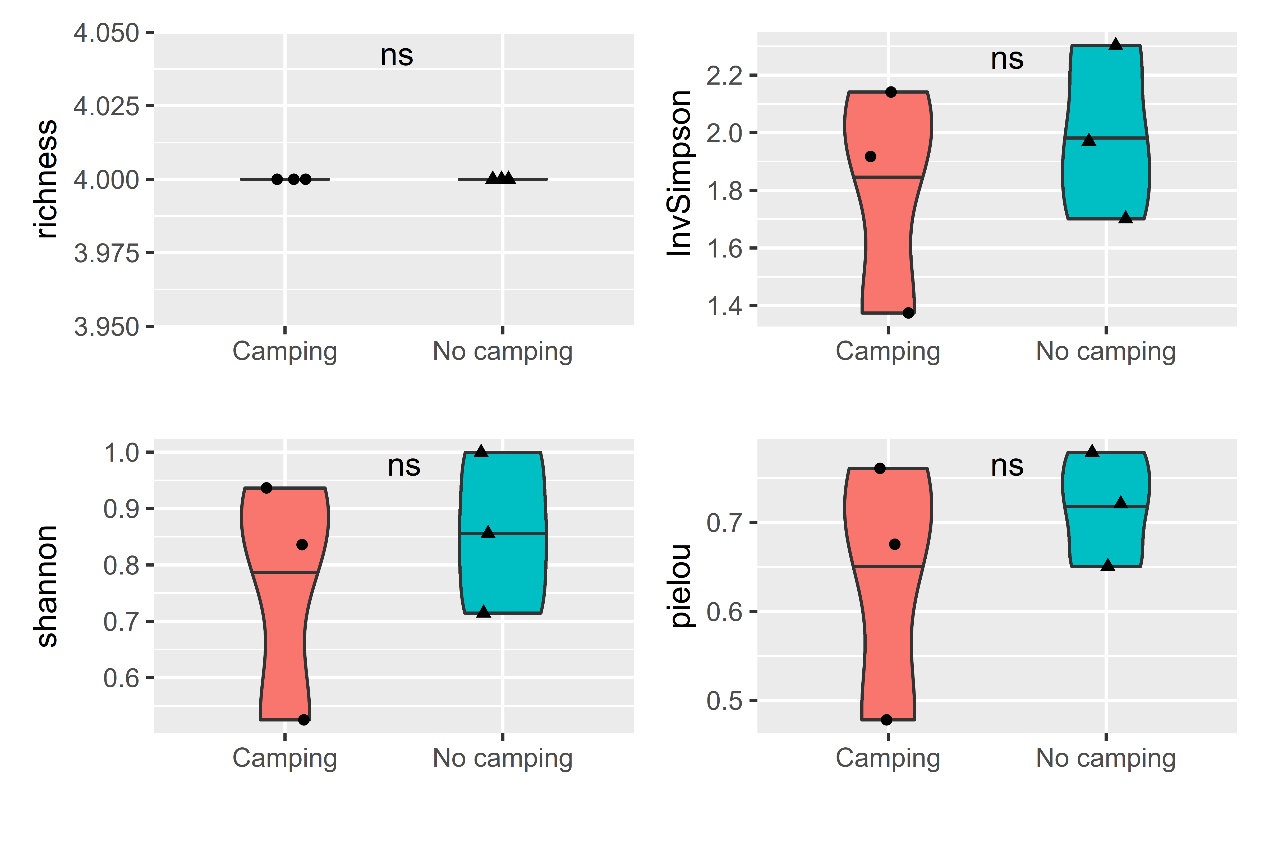


Figure S8. Functional diversities of FAPROTAX1.2.4-based xylanolysis of subsoil bacterial communities under camping and no camping. ns, *p* > 0.05.


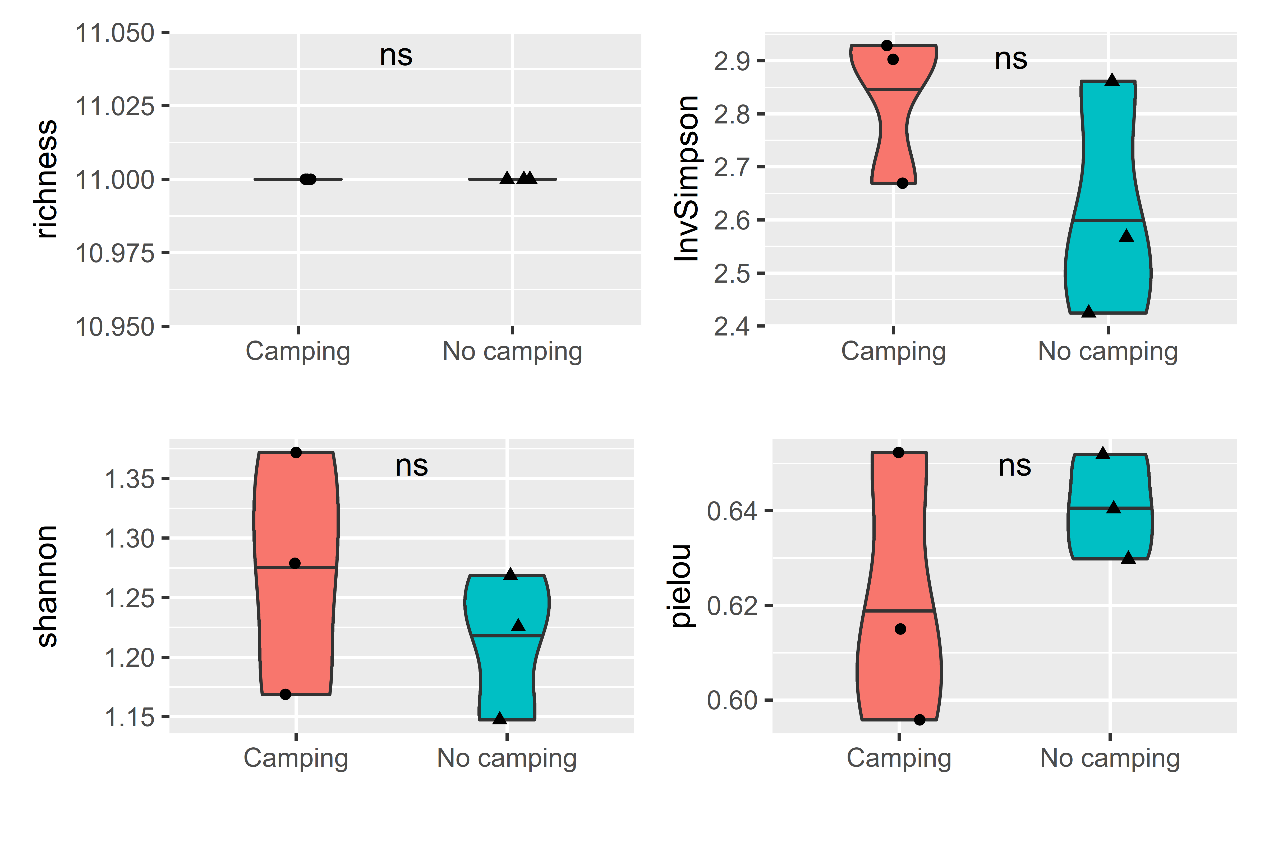


Figure S9. Functional diversities of FAPROTAX1.2.4-based hydrocarbon degradation of subsoil bacterial communities under camping and no camping. ns, *p* > 0.05.


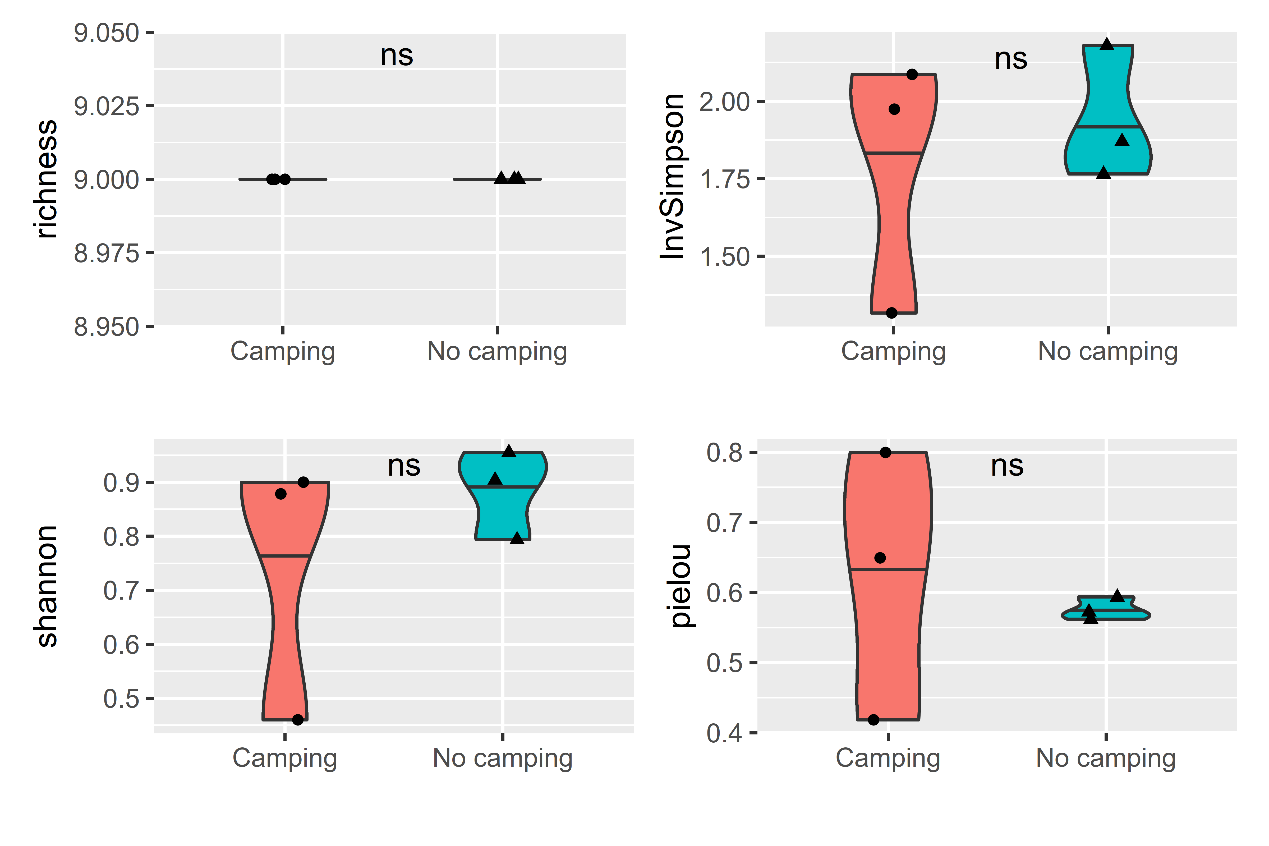


Figure S10. Functional diversities of FAPROTAX1.2.4-based aliphatic non methane hydrocarbon degradation of subsoil bacterial communities under camping and no camping. ns, *p* > 0.05.


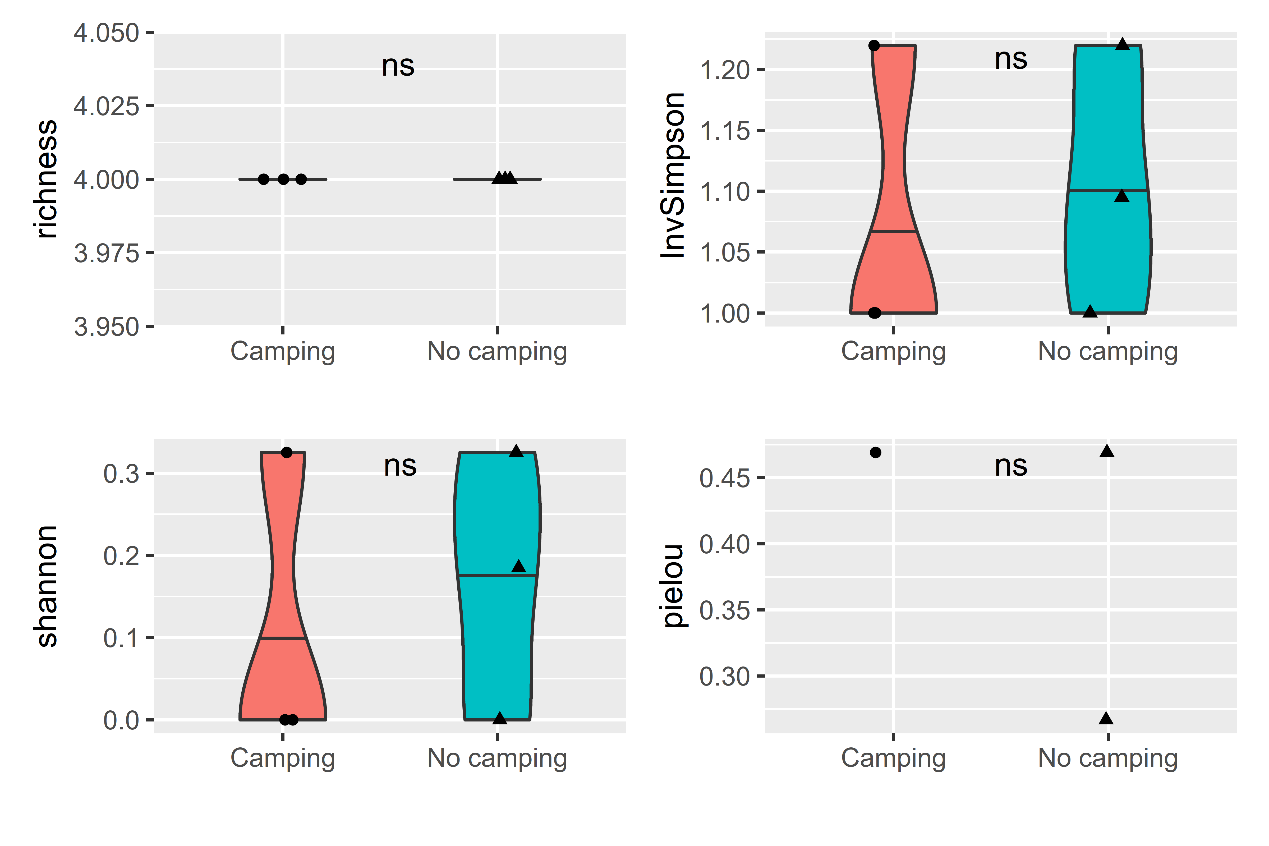


Figure S11. Functional diversities of FAPROTAX1.2.4-based fumarate respiration of subsoil bacterial communities under camping and no camping. ns, *p* > 0.05.


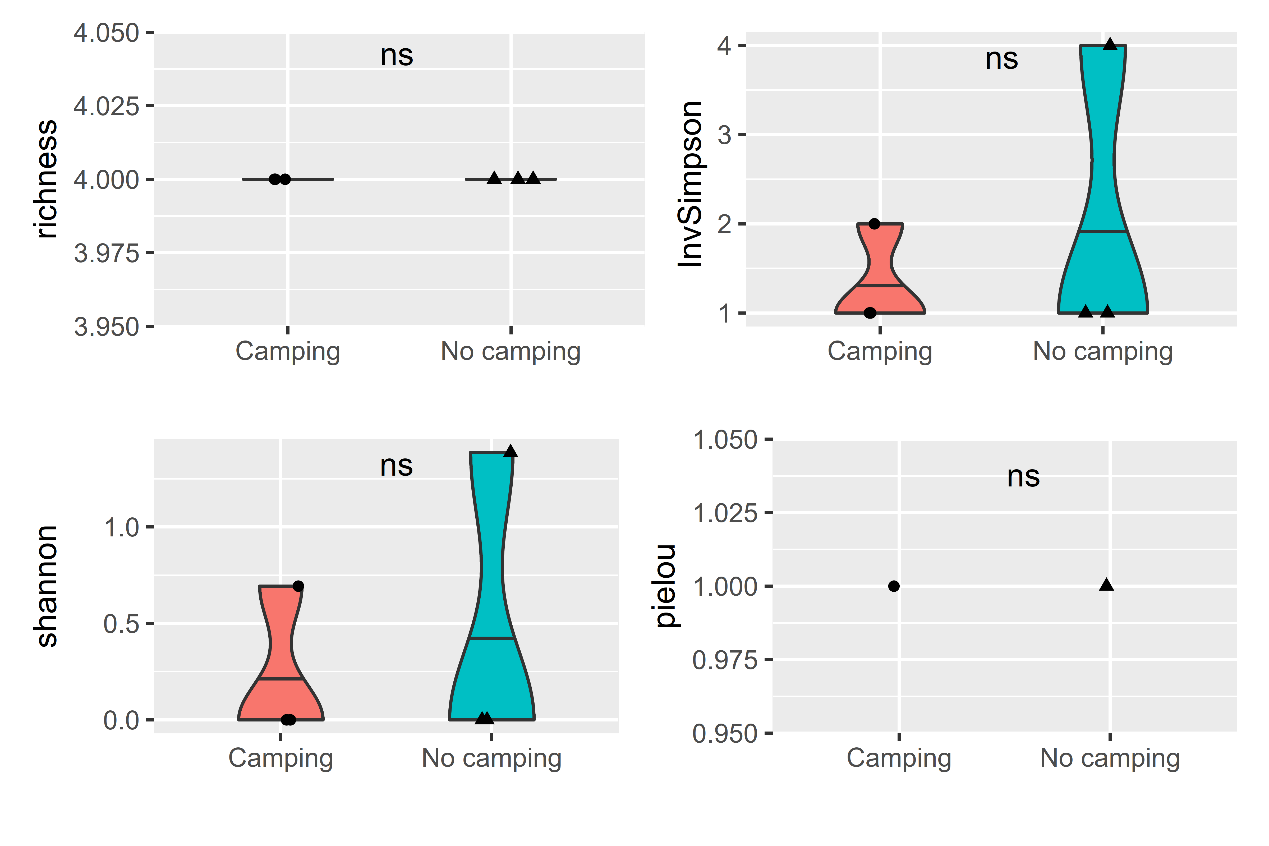


Figure S12. Functional diversities of FAPROTAX1.2.4-based methanogenesis of subsoil bacterial communities under camping and no camping. ns, *p* > 0.05.


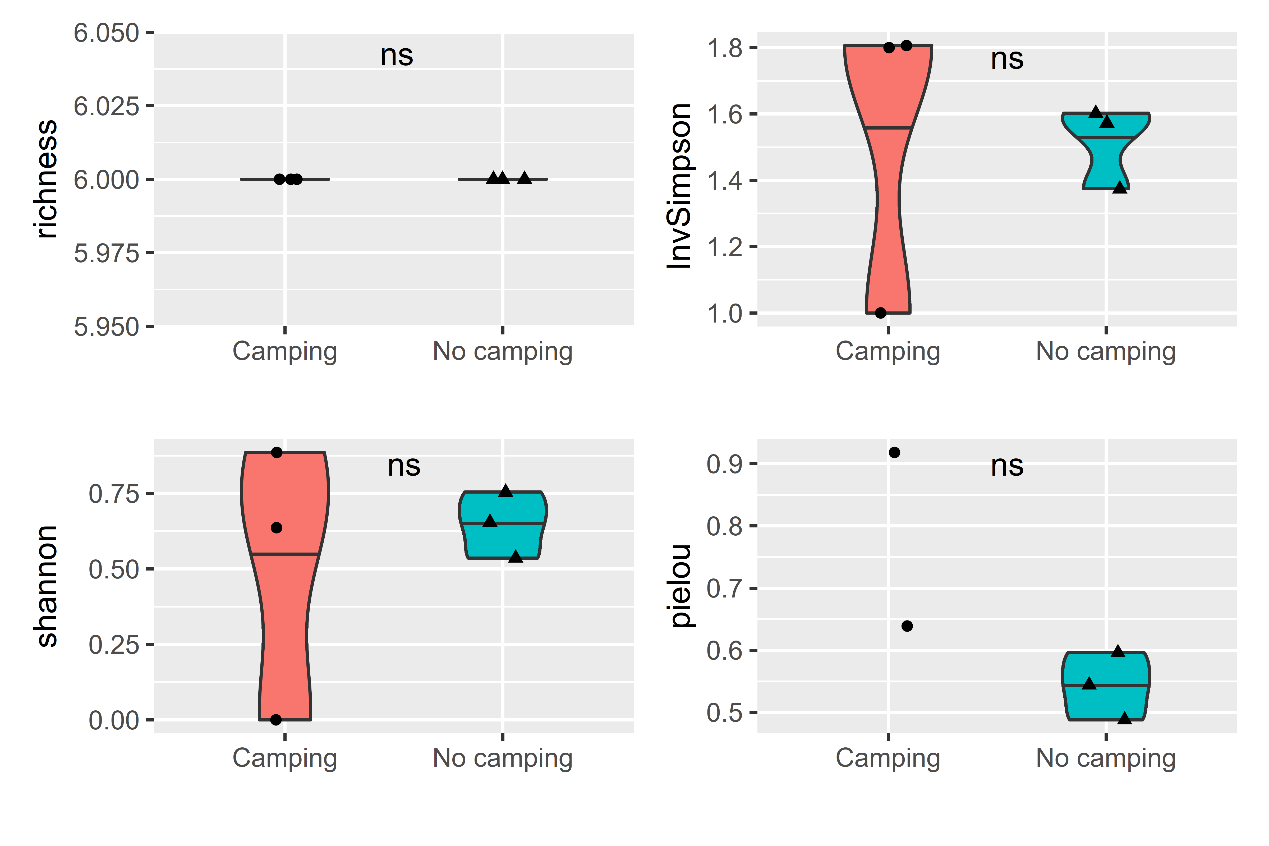


Figure S13. Functional diversities of FAPROTAX1.2.4-based function (methanogenesis by CO_2_ reduction with H_2_) of subsoil bacterial communities under camping and no camping. ns, *p* > 0.05.


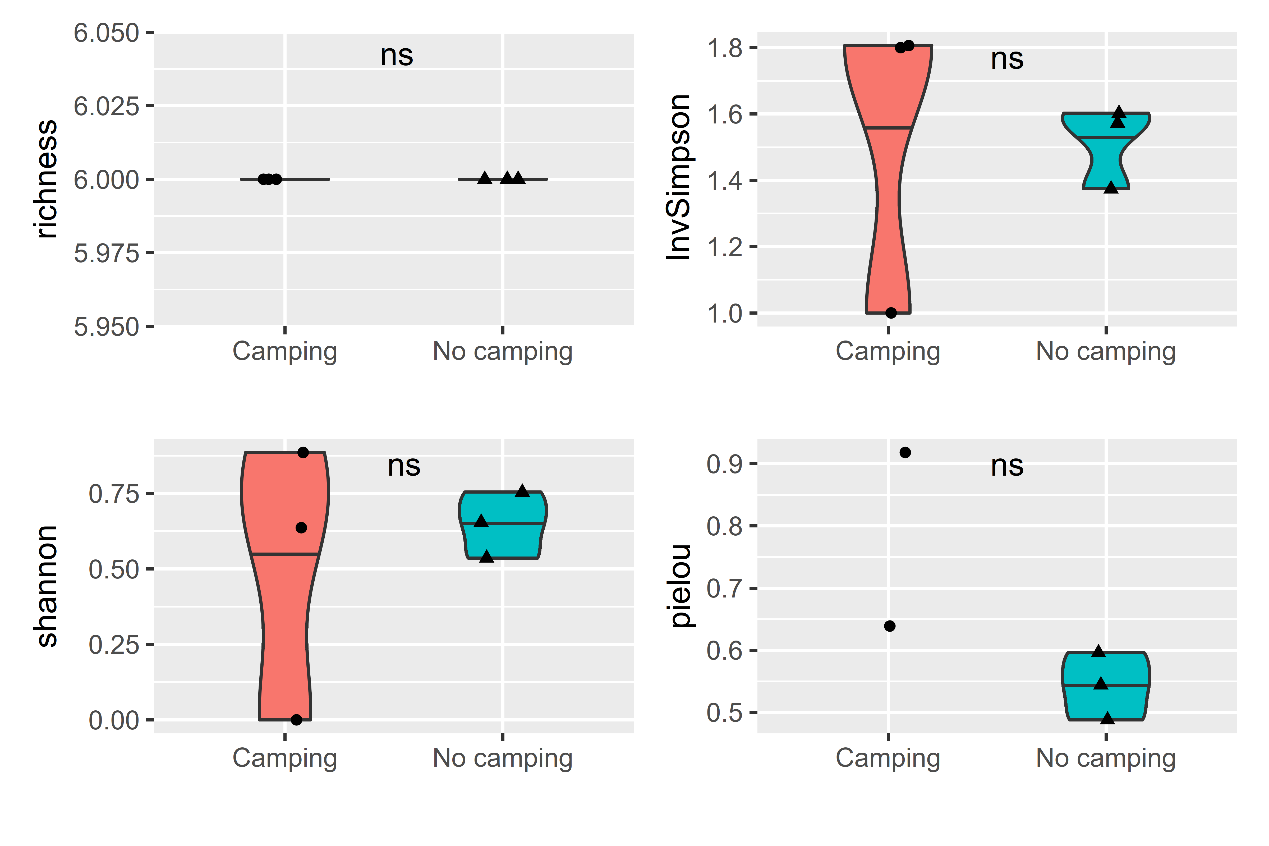


Figure S14. Functional diversities of FAPROTAX1.2.4-based methanol oxidation of subsoil bacterial communities under camping and no camping. ns, *p* > 0.05.


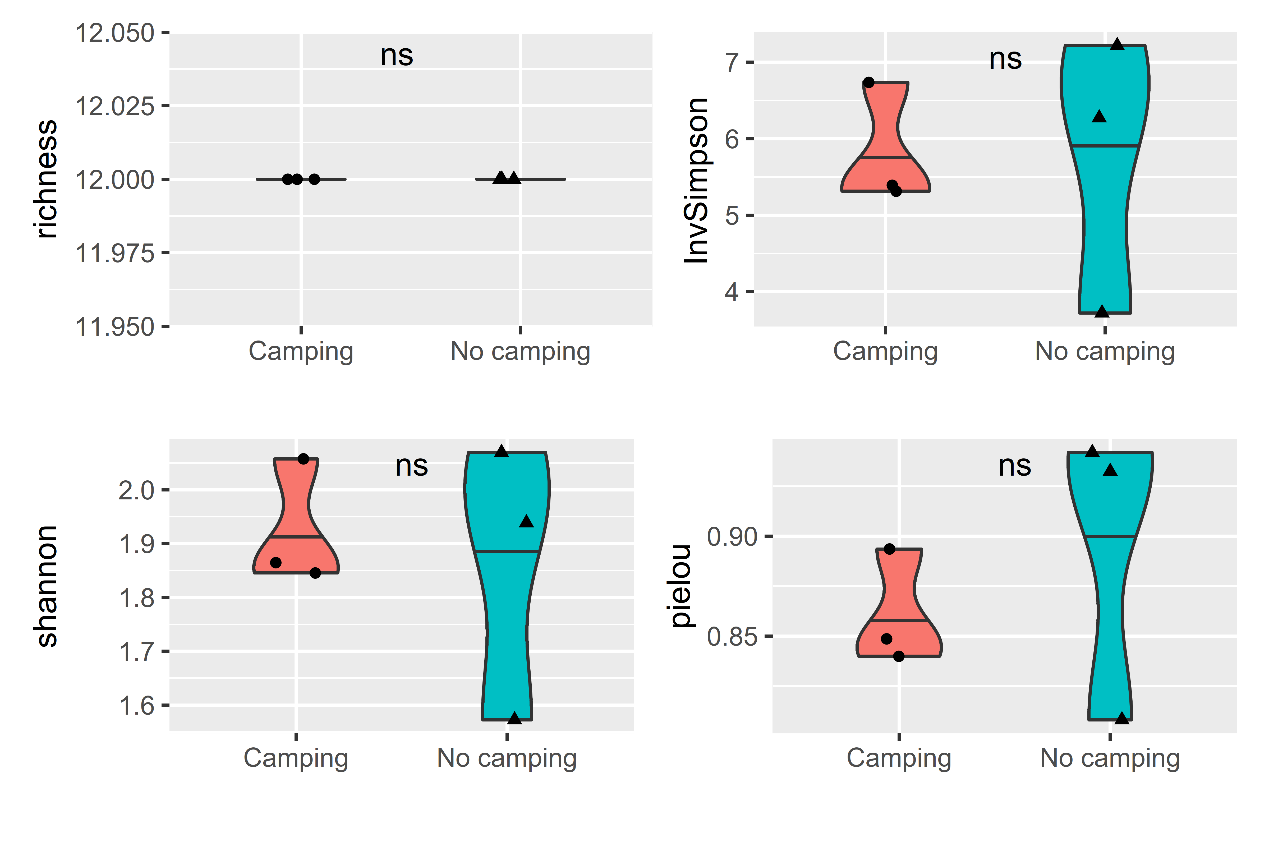


Figure S15. Functional diversities of FAPROTAX1.2.4-based methylotrophy of subsoil bacterial communities under camping and no camping. ns, *p* > 0.05.


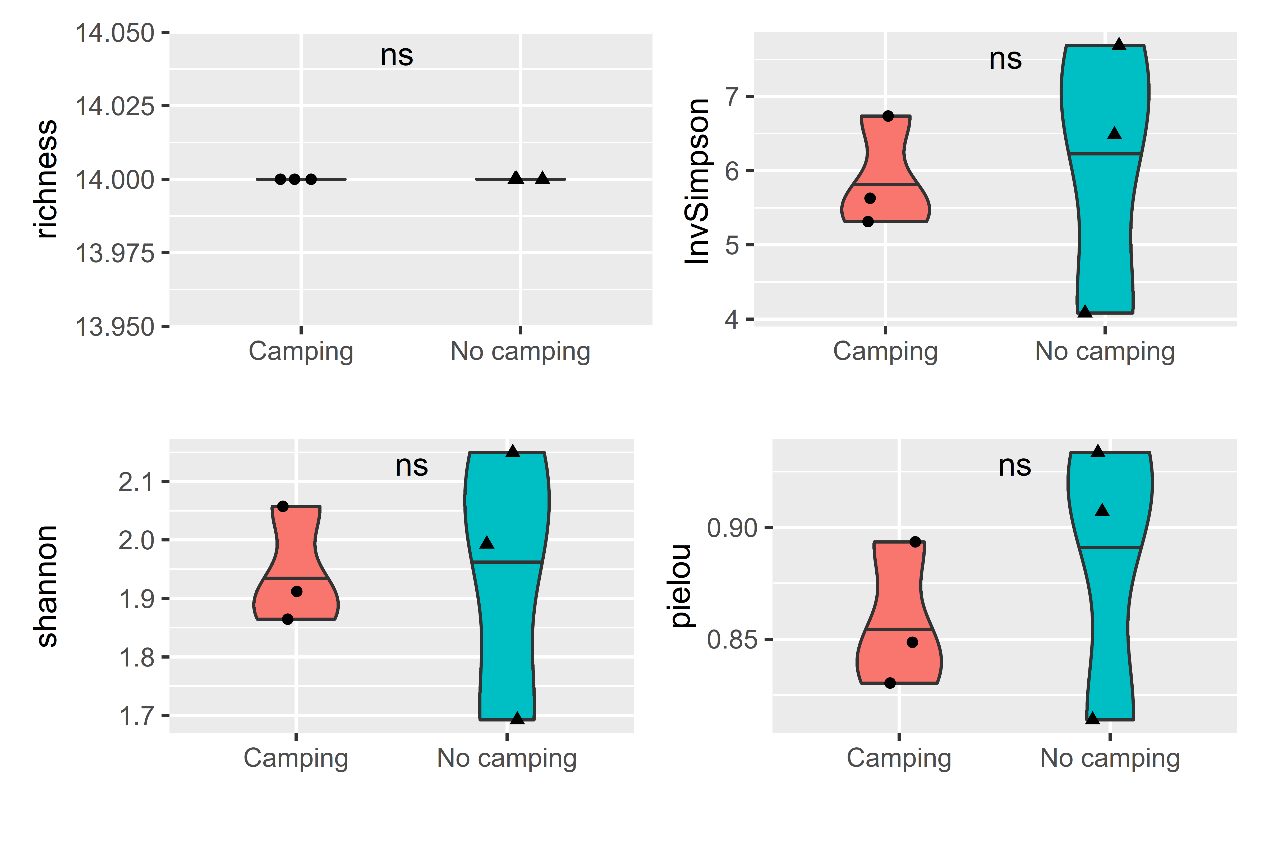


Figure S16. Functional diversities of FAPROTAX1.2.4-based hydrogenotrophic methanogenesis of subsoil bacterial communities under camping and no camping. ns, *p* > 0.05.


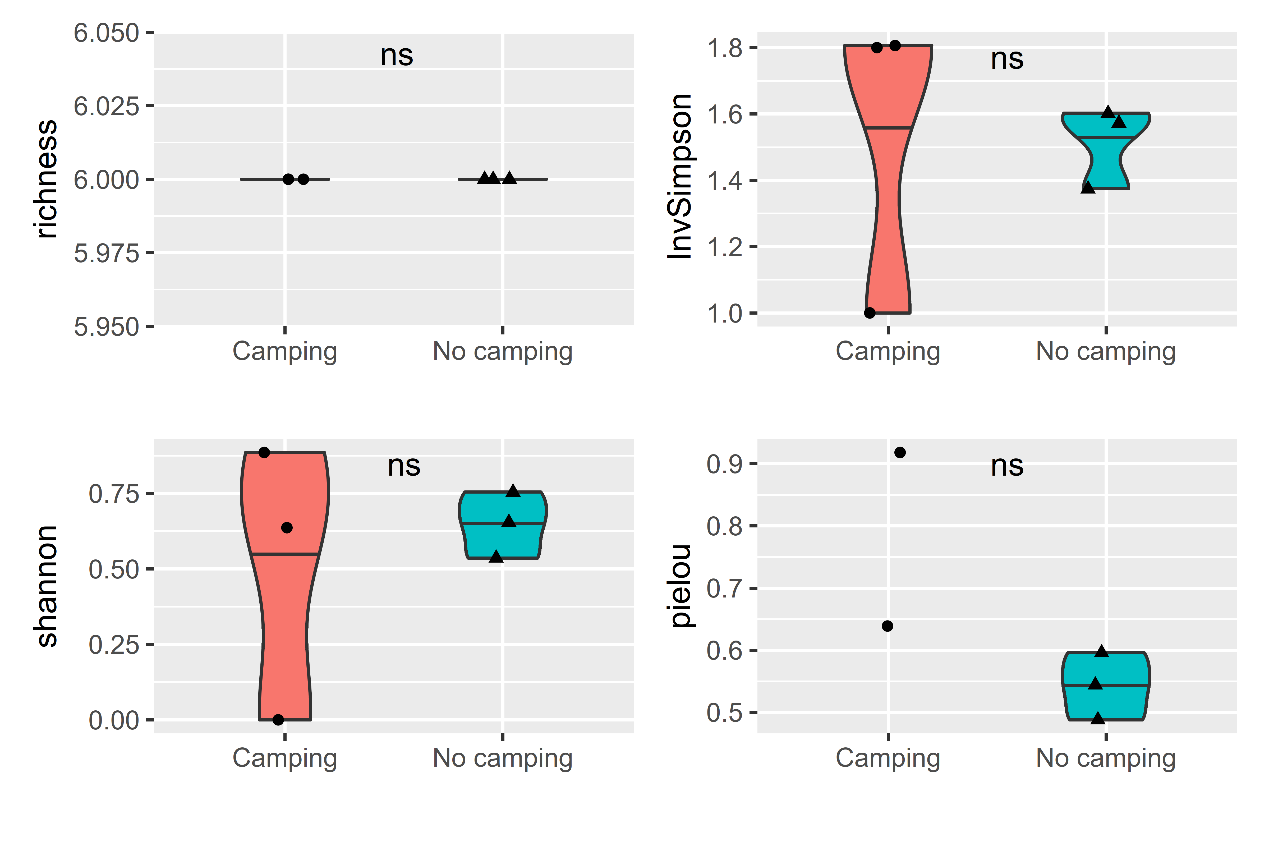


Figure S17. Functional diversities of FAPROTAX1.2.4-based plastic degradation of subsoil bacterial communities under camping and no camping. ns, *p* > 0.05.


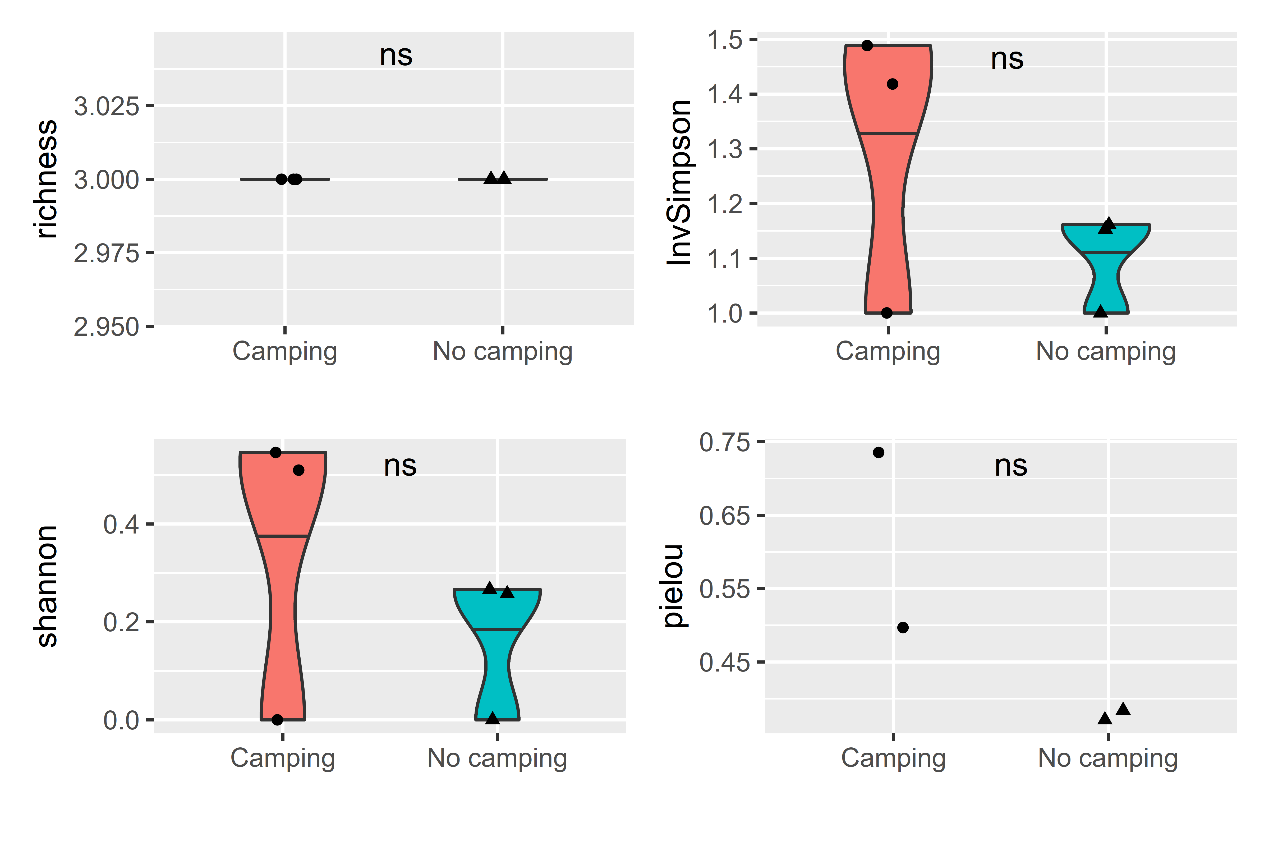


Figure S18. Functional diversities of FAPROTAX1.2.4-based nitrogen fixation of subsoil bacterial communities under camping and no camping. ns, *p* > 0.05.


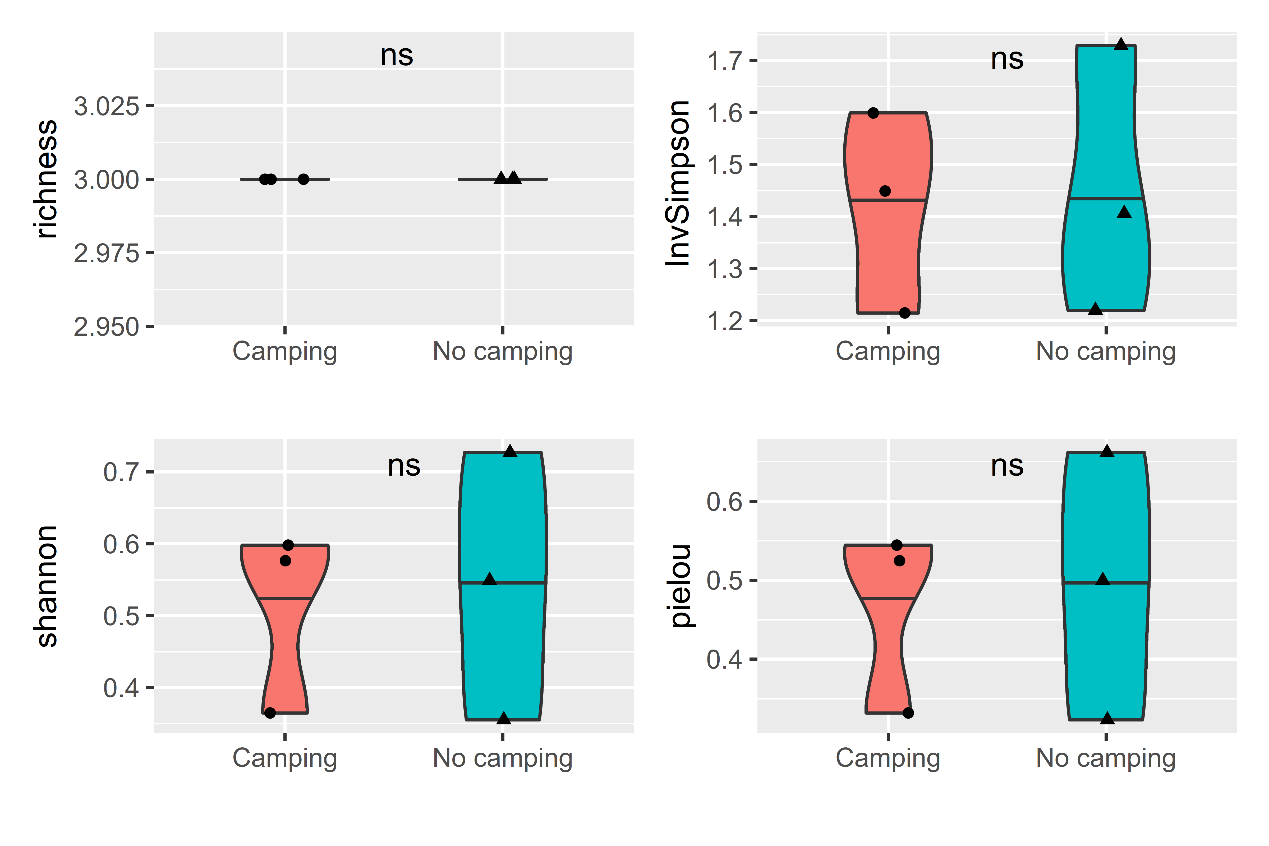


Figure S19. Functional diversities of FAPROTAX1.2.4-based nitrate ammonification of subsoil bacterial communities under camping and no camping. ns, *p* > 0.05.


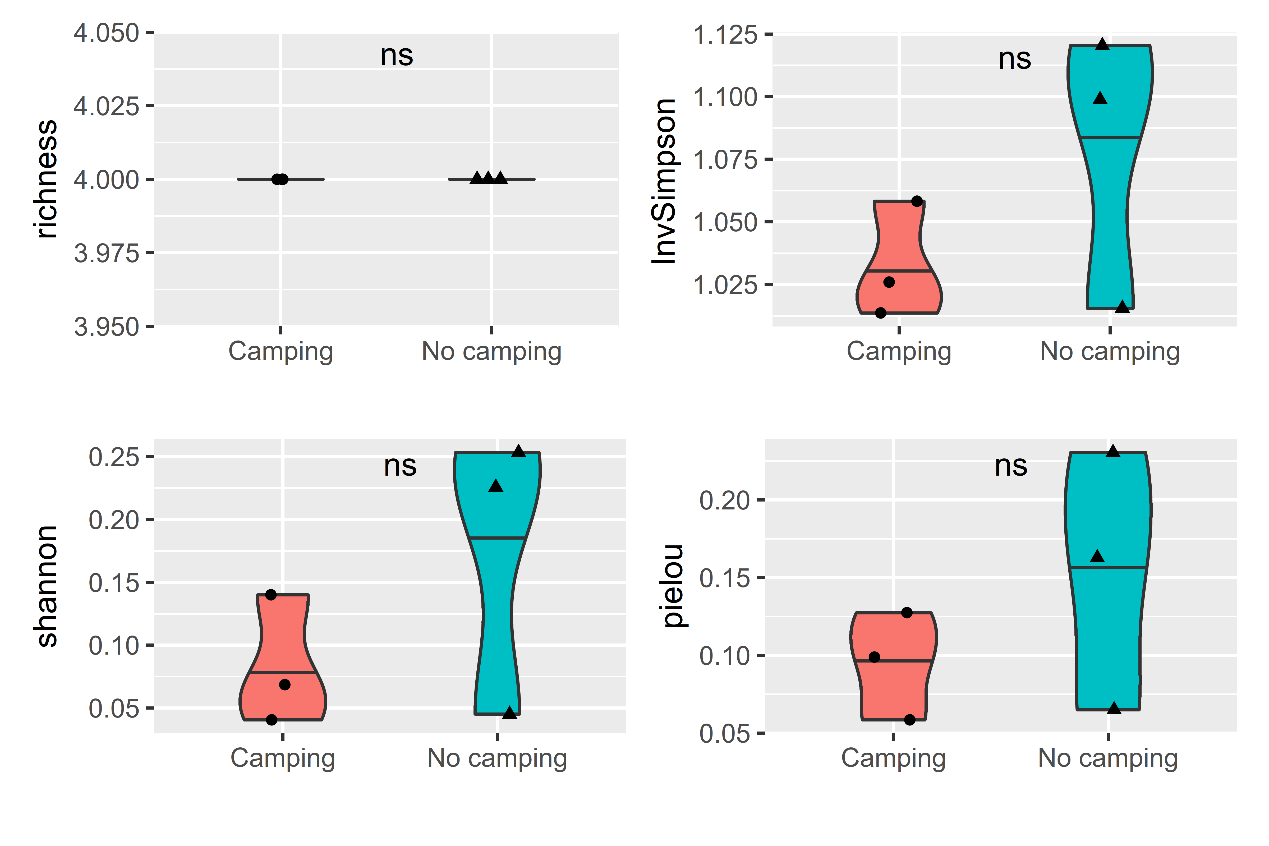


Figure S20. Functional diversities of FAPROTAX1.2.4-based nitrate denitrification of subsoil bacterial communities under camping and no camping. ns, *p* > 0.05.


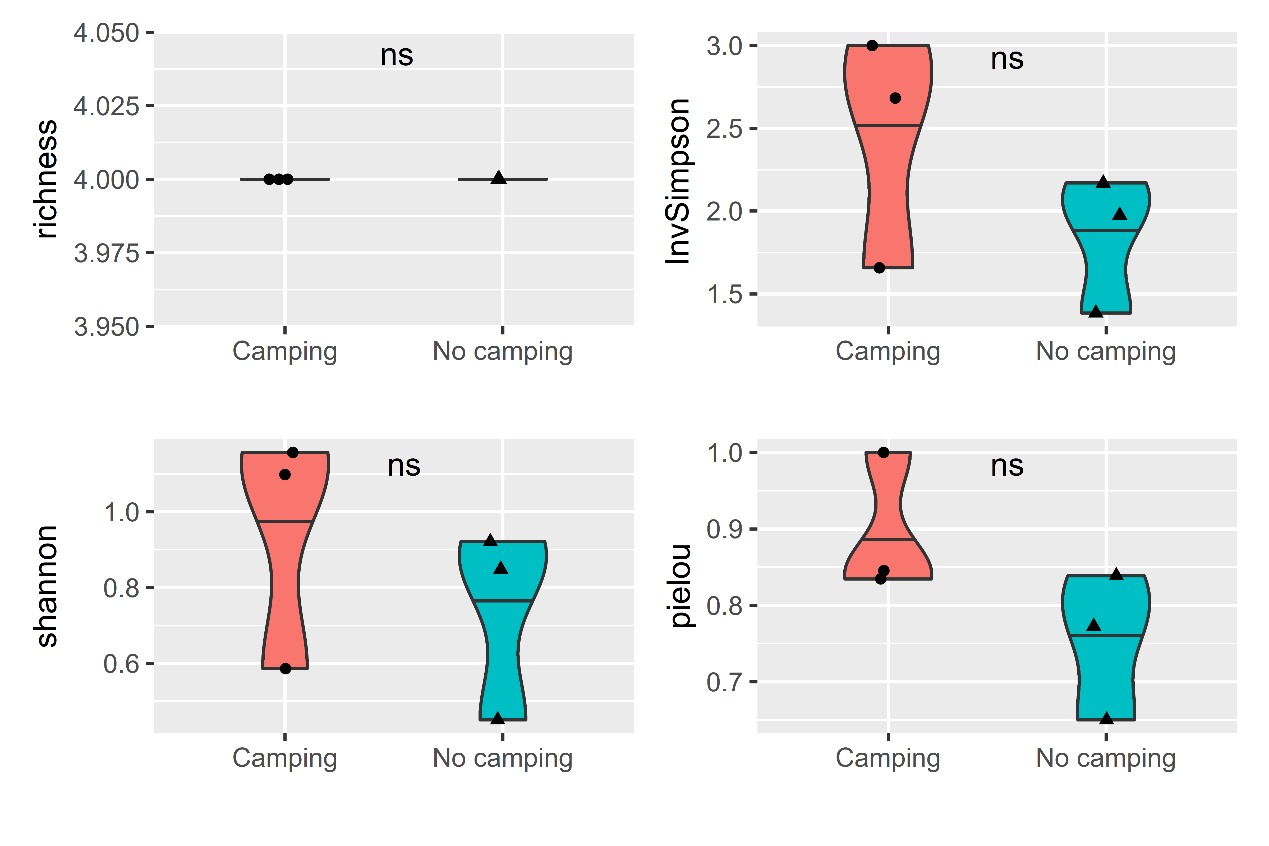


Figure S21. Functional diversities of FAPROTAX1.2.4-based nitrate reduction of subsoil bacterial communities under camping and no camping. ns, *p* > 0.05.


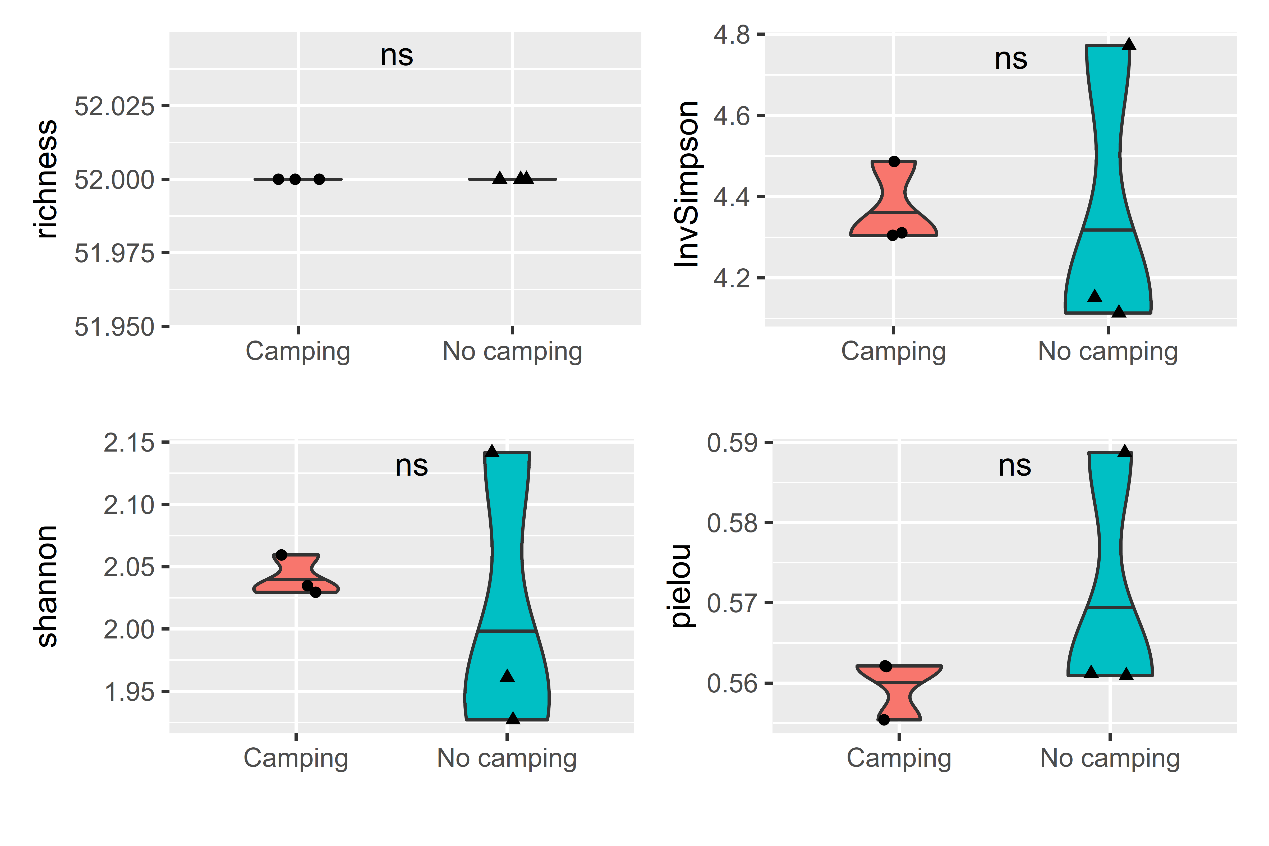


Figure S22. Functional diversities of FAPROTAX1.2.4-based nitrate respiration of subsoil bacterial communities under camping and no camping. ns, *p* > 0.05.


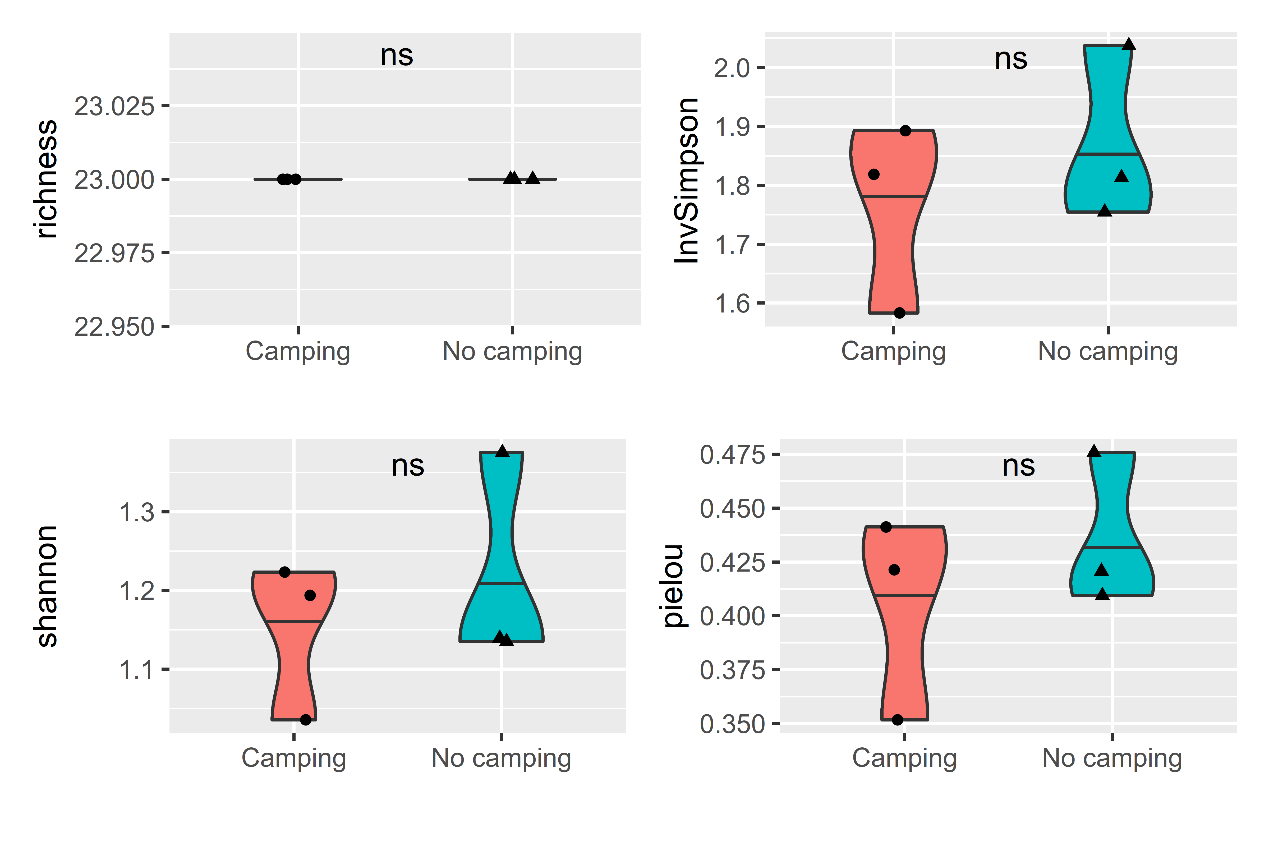


Figure S23. Functional diversities of FAPROTAX1.2.4-based nitrification of subsoil bacterial communities under camping and no camping. ns, *p* > 0.05.


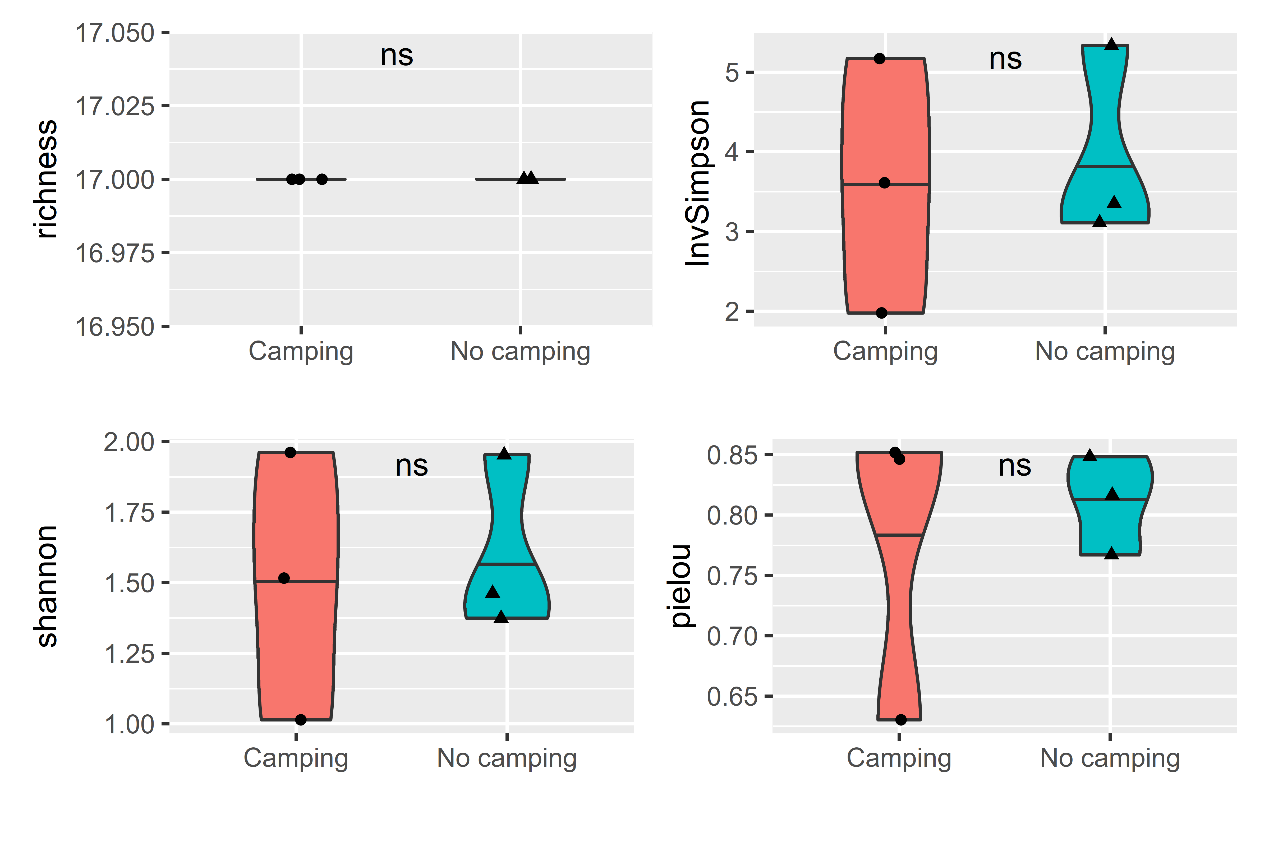


Figure S24. Functional diversities of FAPROTAX1.2.4-based nitrite ammonification of subsoil bacterial communities under camping and no camping. ns, *p* > 0.05.


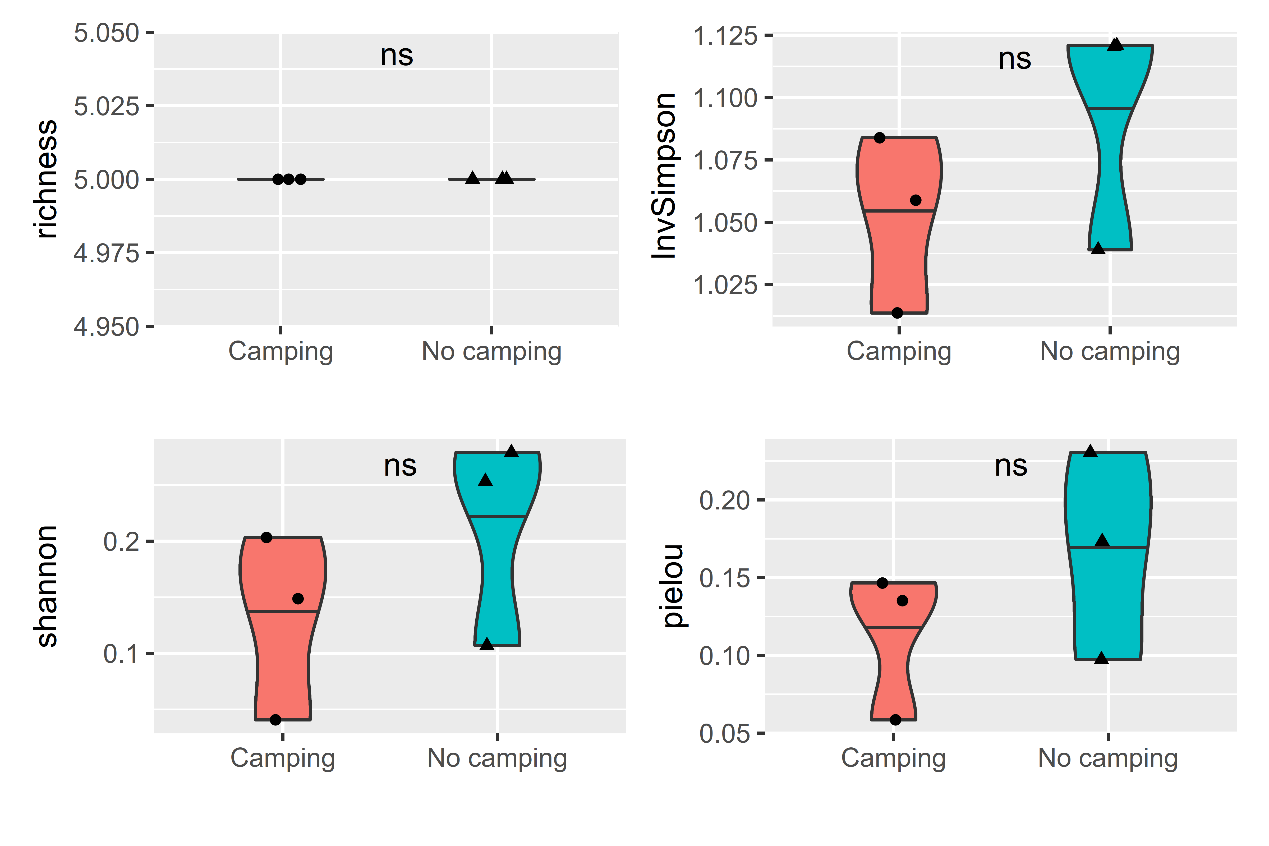


Figure S25. Functional diversities of FAPROTAX1.2.4-based nitrite denitrification of subsoil bacterial communities under camping and no camping. ns, *p* > 0.05.


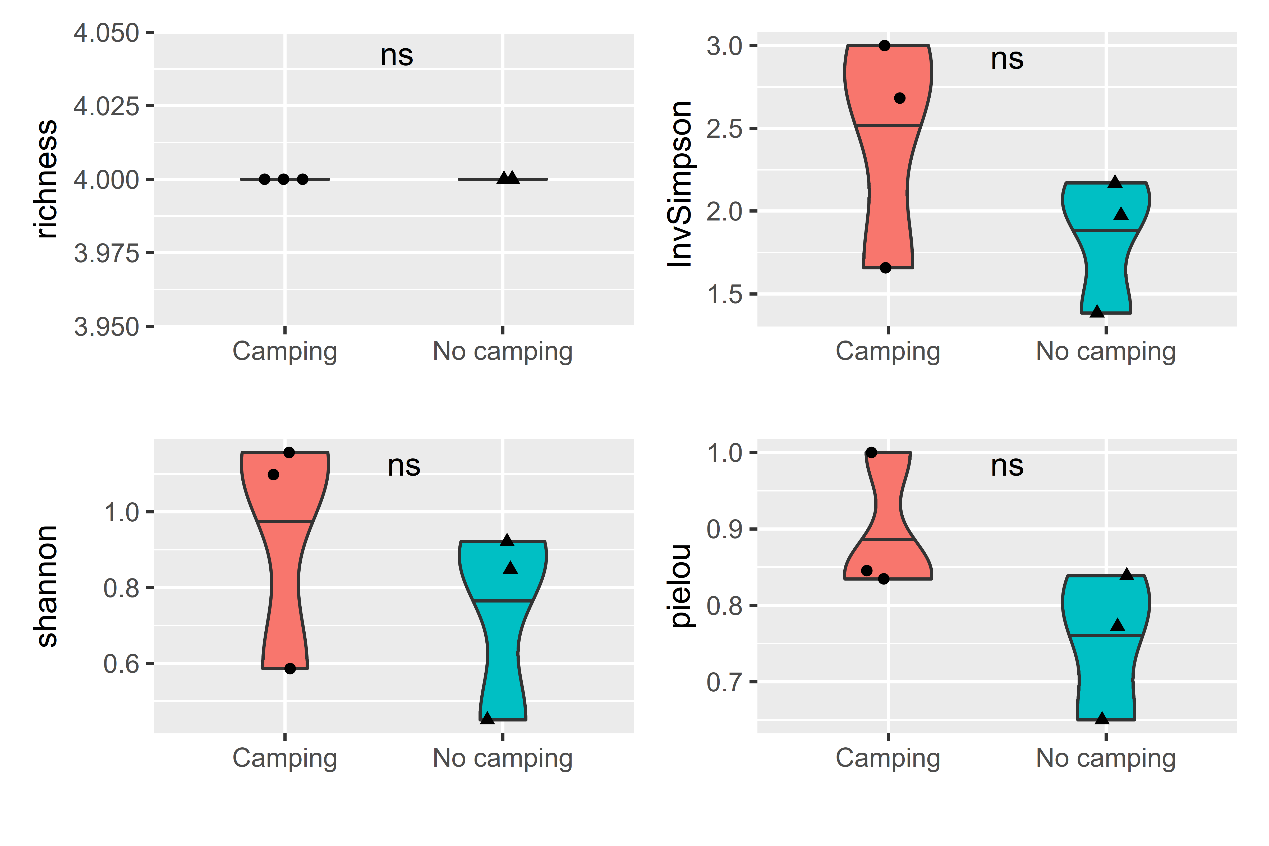


Figure S26. Functional diversities of FAPROTAX1.2.4-based nitrite respiration of subsoil bacterial communities under camping and no camping. ns, *p* > 0.05.


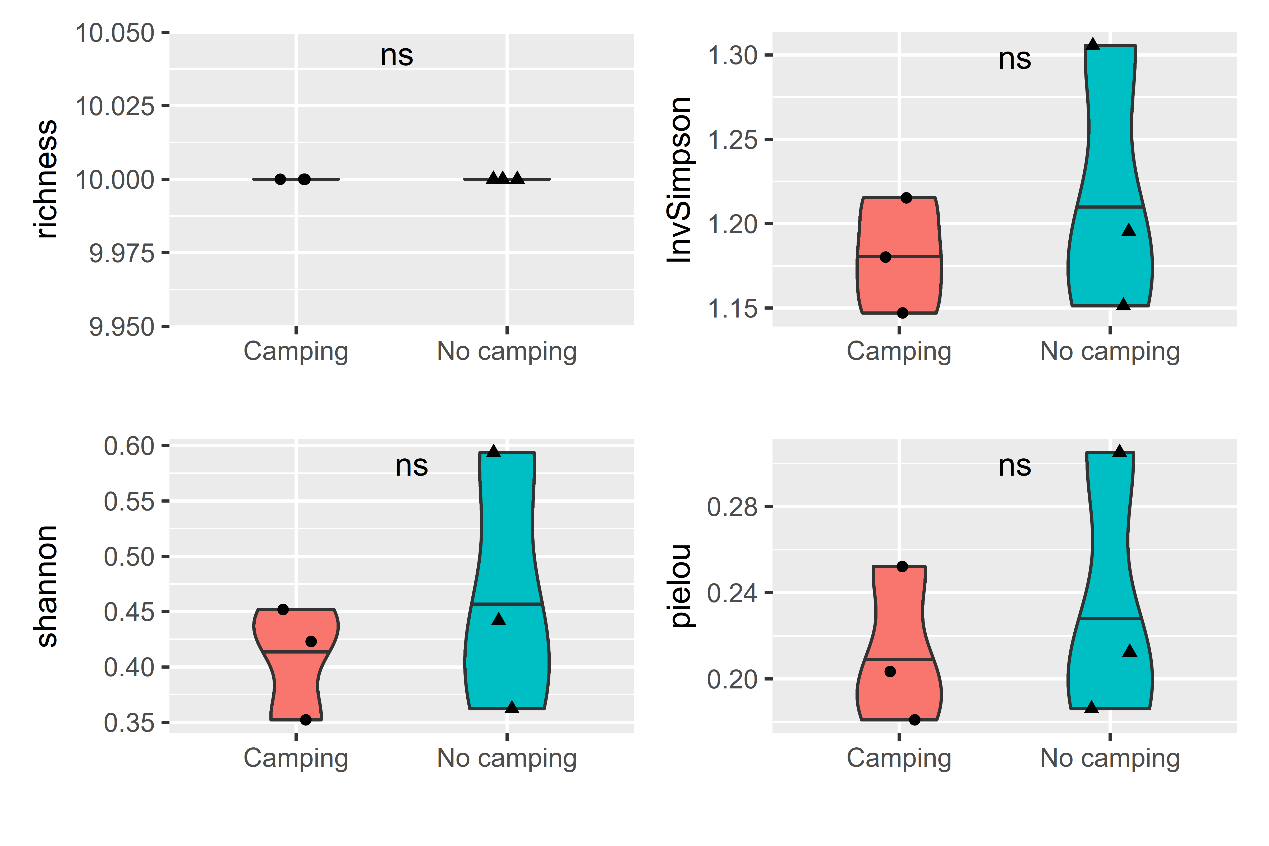


Figure S27. Functional diversities of FAPROTAX1.2.4-based nitrogen respiration of subsoil bacterial communities under camping and no camping. ns, *p* > 0.05.


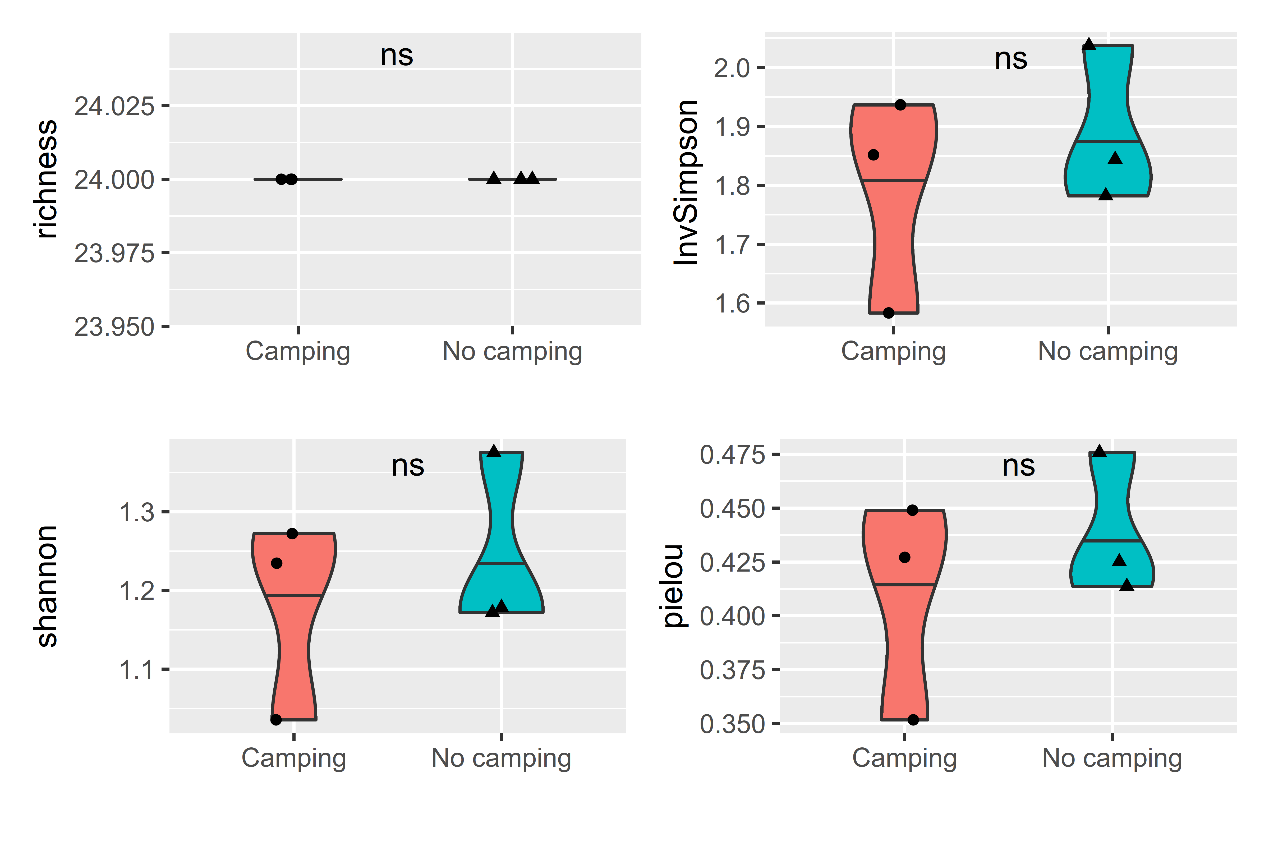


Figure S28. Functional diversities of FAPROTAX1.2.4-based nitrous oxide denitrification of subsoil bacterial communities under camping and no camping. ns, *p* > 0.05.


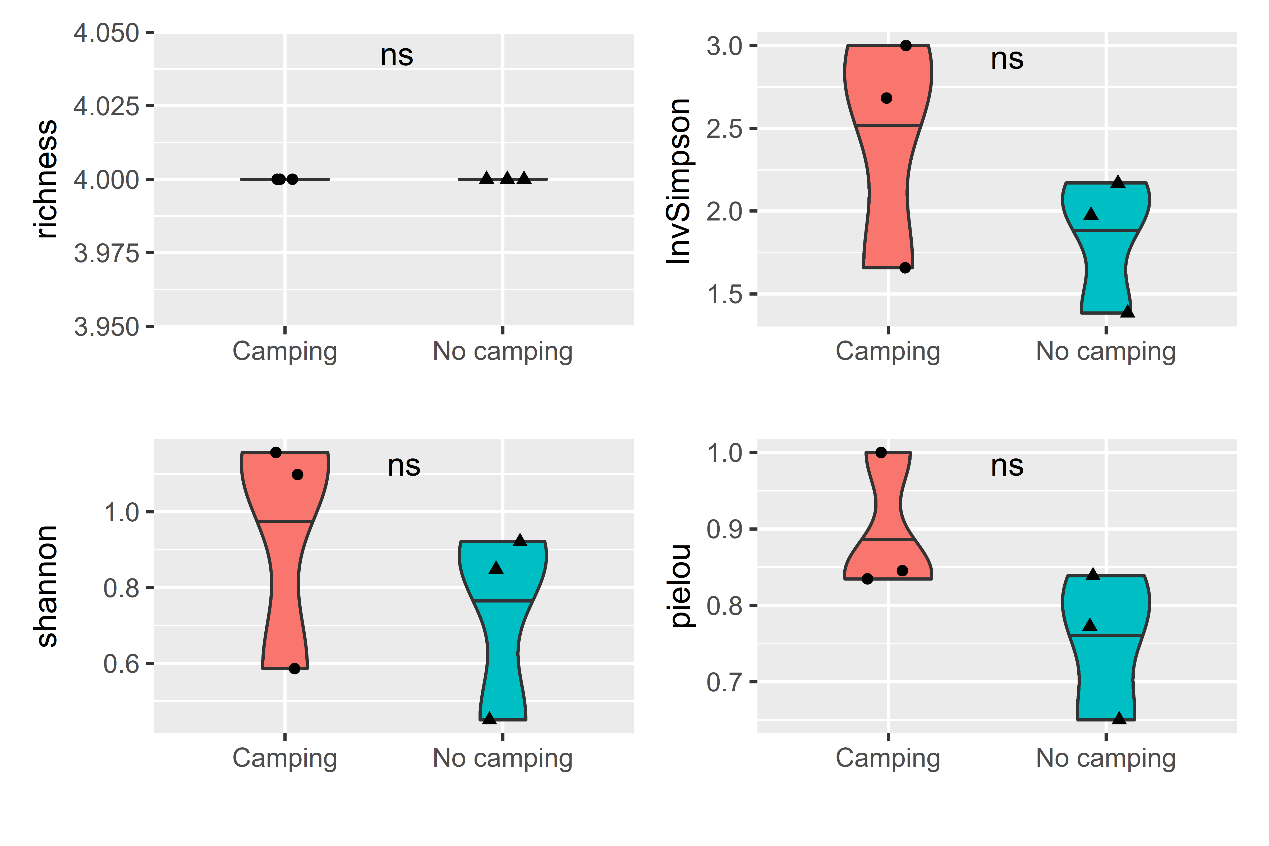


Figure S29. Functional diversities of FAPROTAX1.2.4-based denitrification of subsoil bacterial communities under camping and no camping. ns, *p* > 0.05.


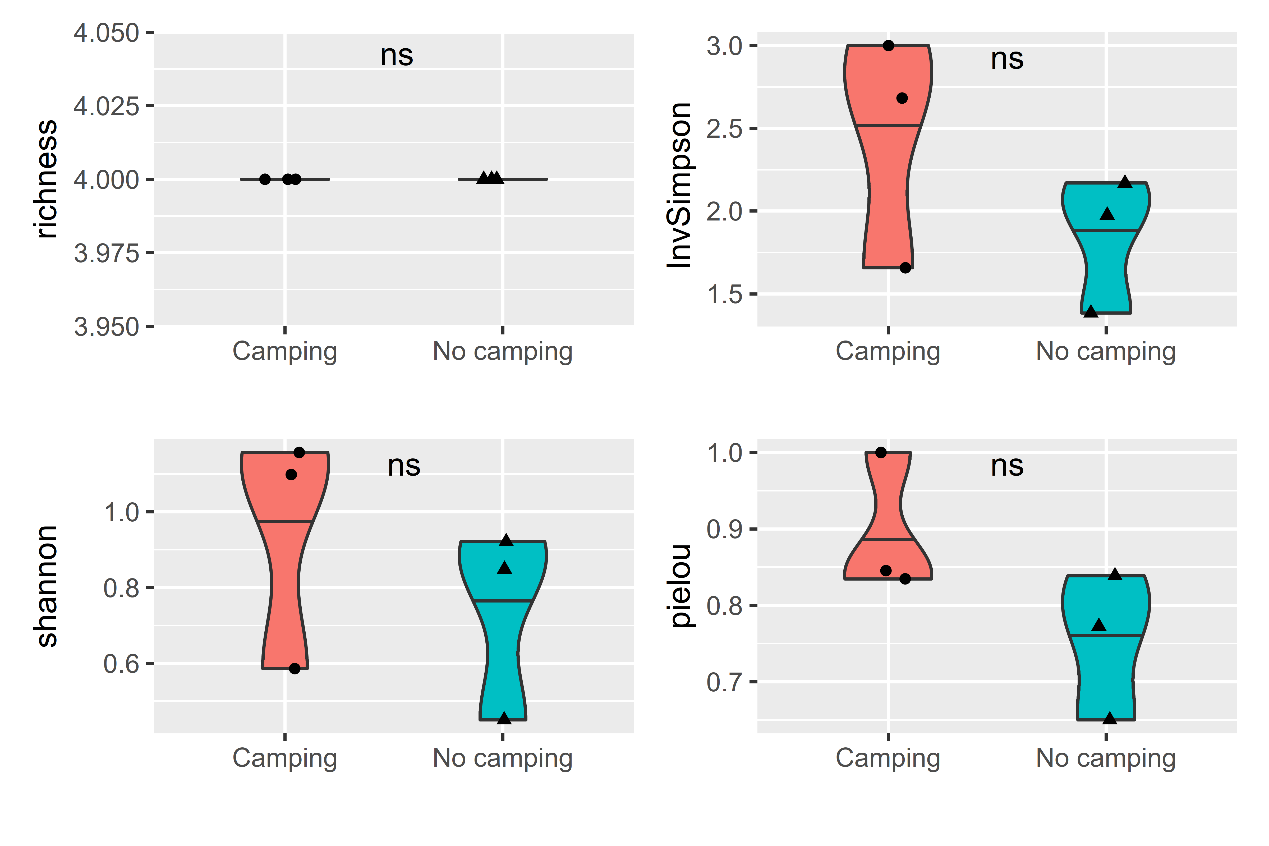


Figure 30. Functional diversities of FAPROTAX1.2.4-based aerobic ammonia oxidation of subsoil bacterial communities under camping and no camping. ns, *p* > 0.05.


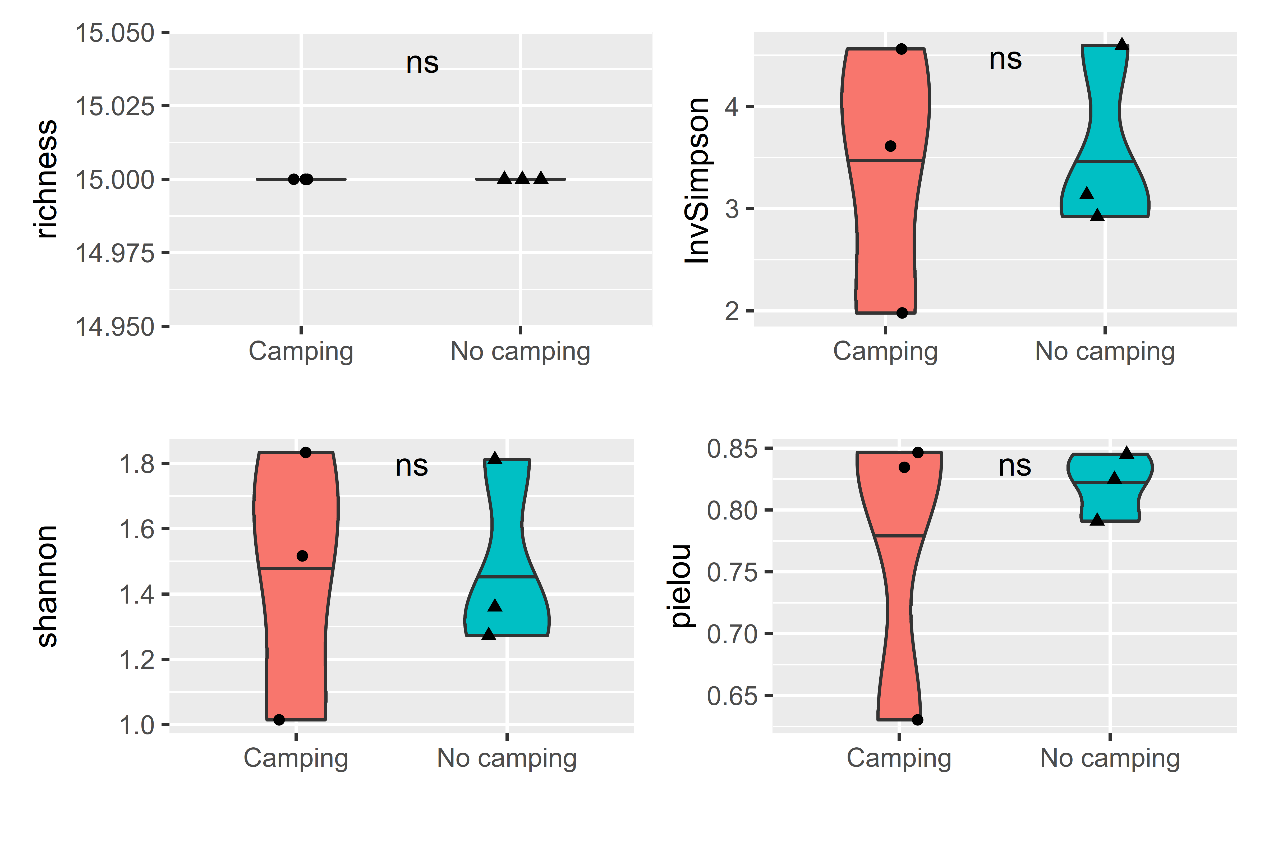


Figure S31. Functional diversities of FAPROTAX1.2.4-based ureolysis of subsoil bacterial communities under camping and no camping. ns, *p* > 0.05.


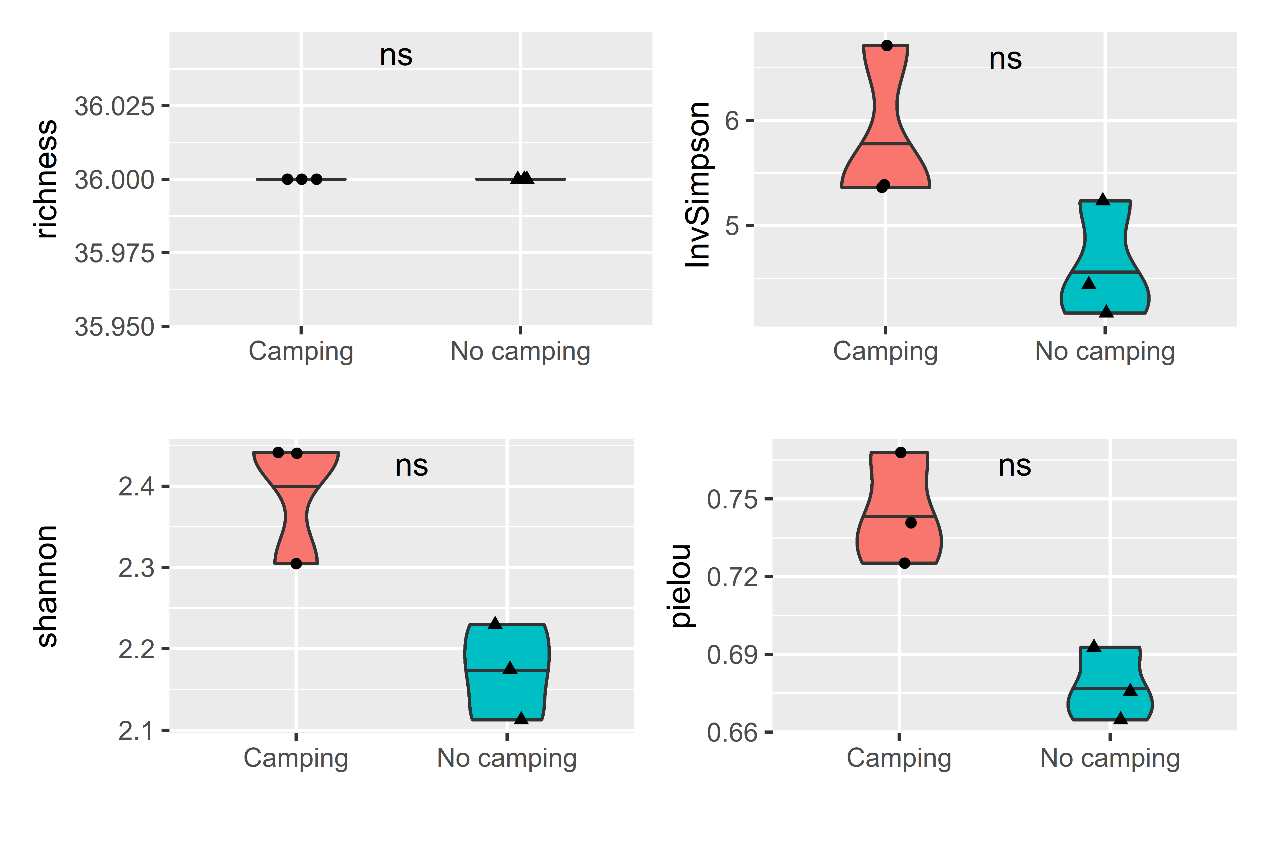


Figure S32. Functional diversities of FAPROTAX1.2.4-based dark hydrogen oxidation of subsoil bacterial communities under camping and no camping. ns, *p* > 0.05.


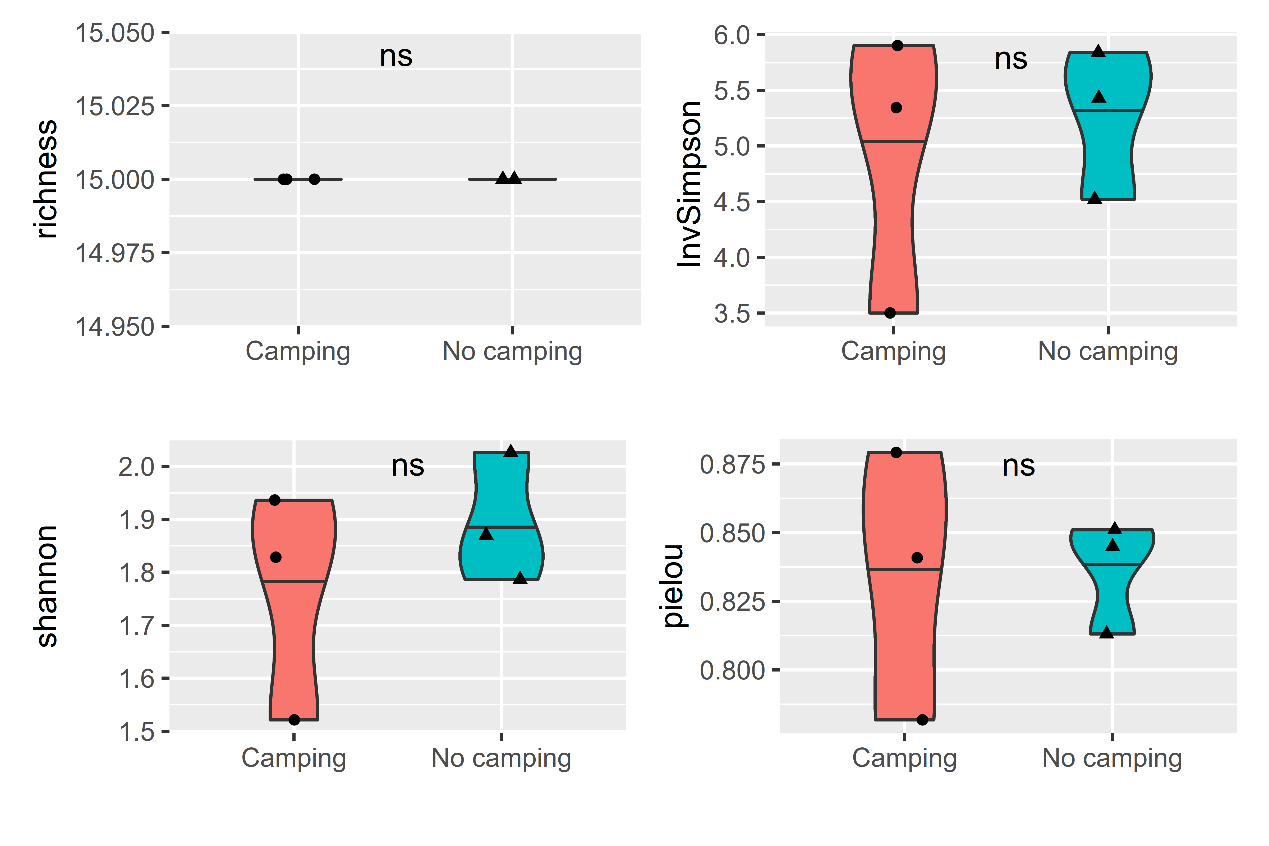


Figure S33. Functional diversities of FAPROTAX1.2.4-based dark oxidation of sulfur compounds of subsoil bacterial communities under camping and no camping. ns, *p* > 0.05.


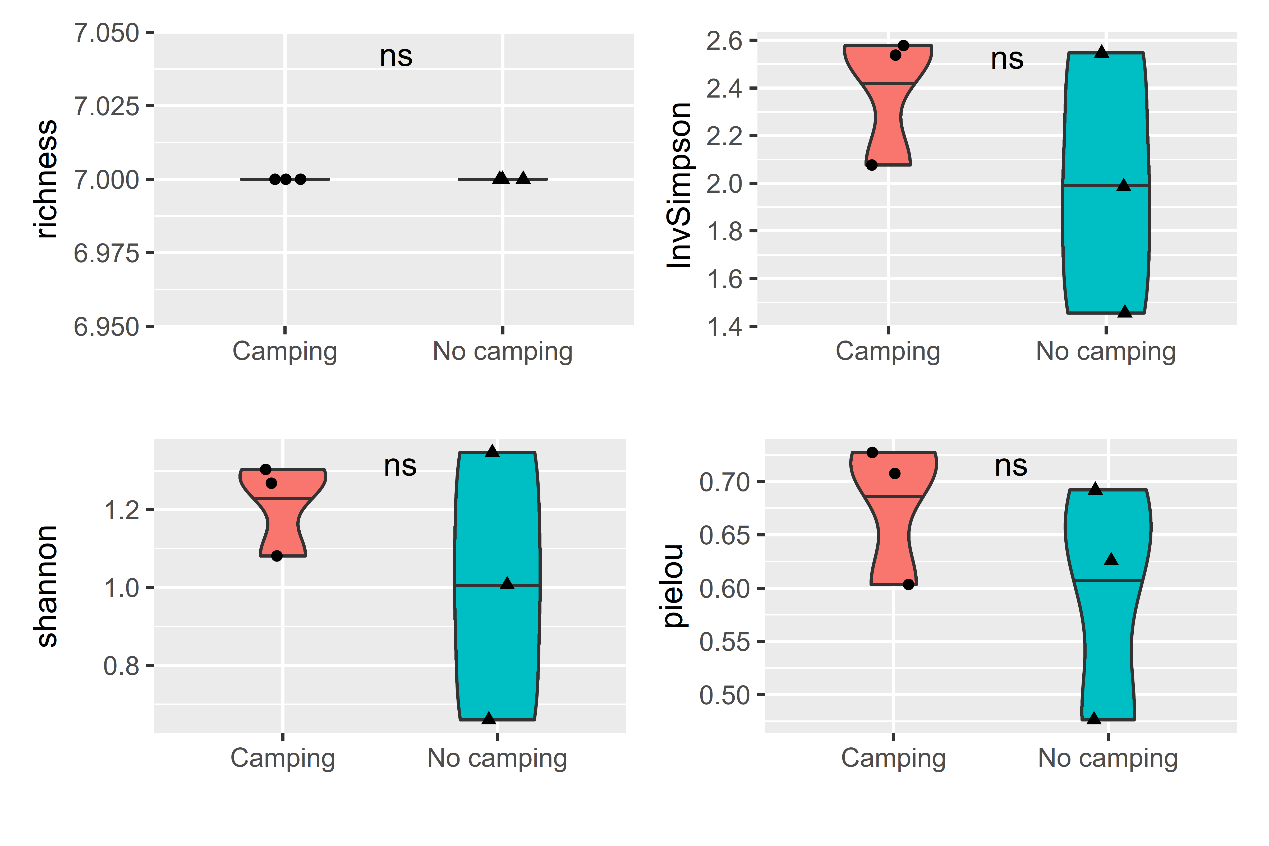


Figure S34. Functional diversities of FAPROTAX1.2.4-based respiration of sulfur compounds of subsoil bacterial communities under camping and no camping. ns, *p* > 0.05.


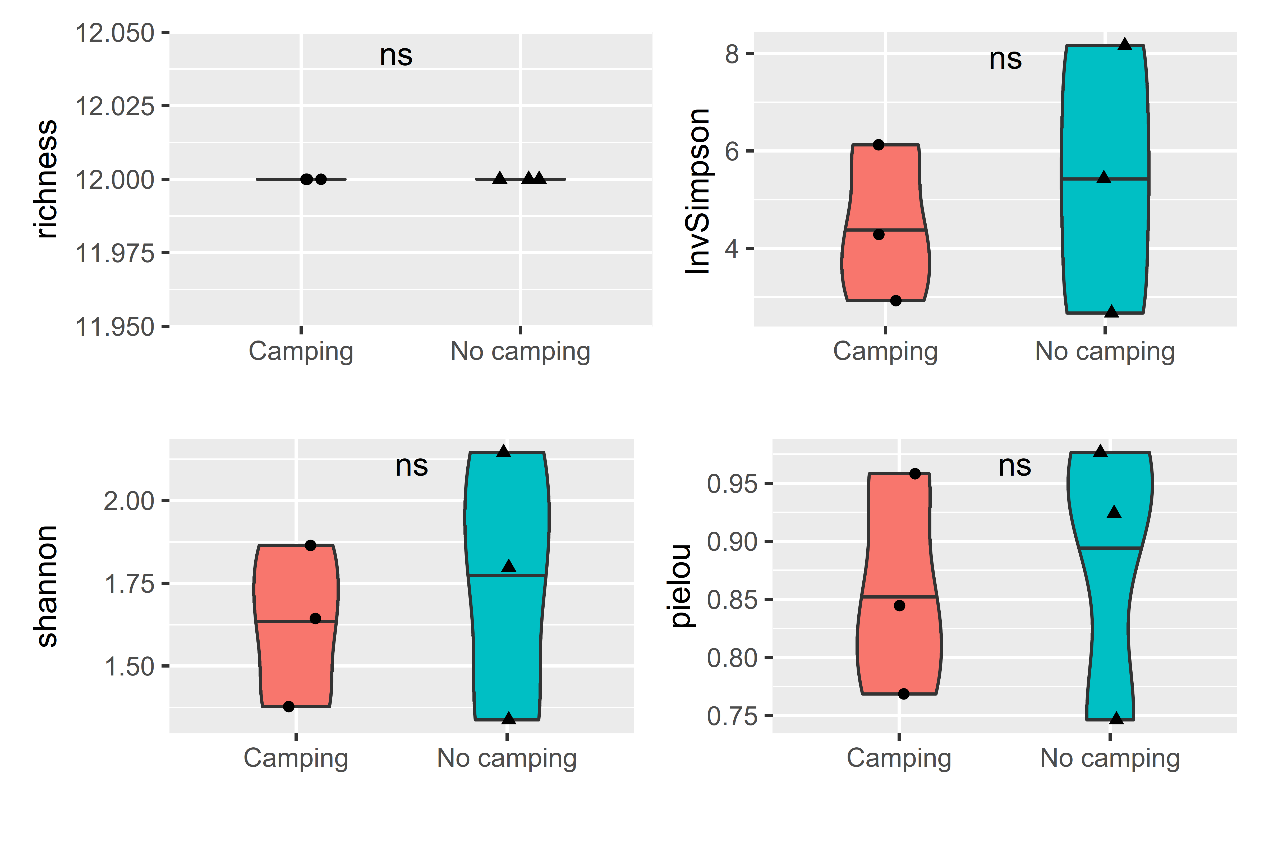


Figure S35. Functional diversities of FAPROTAX1.2.4-based sulfate respiration of subsoil bacterial communities under camping and no camping. ns, *p* > 0.05.


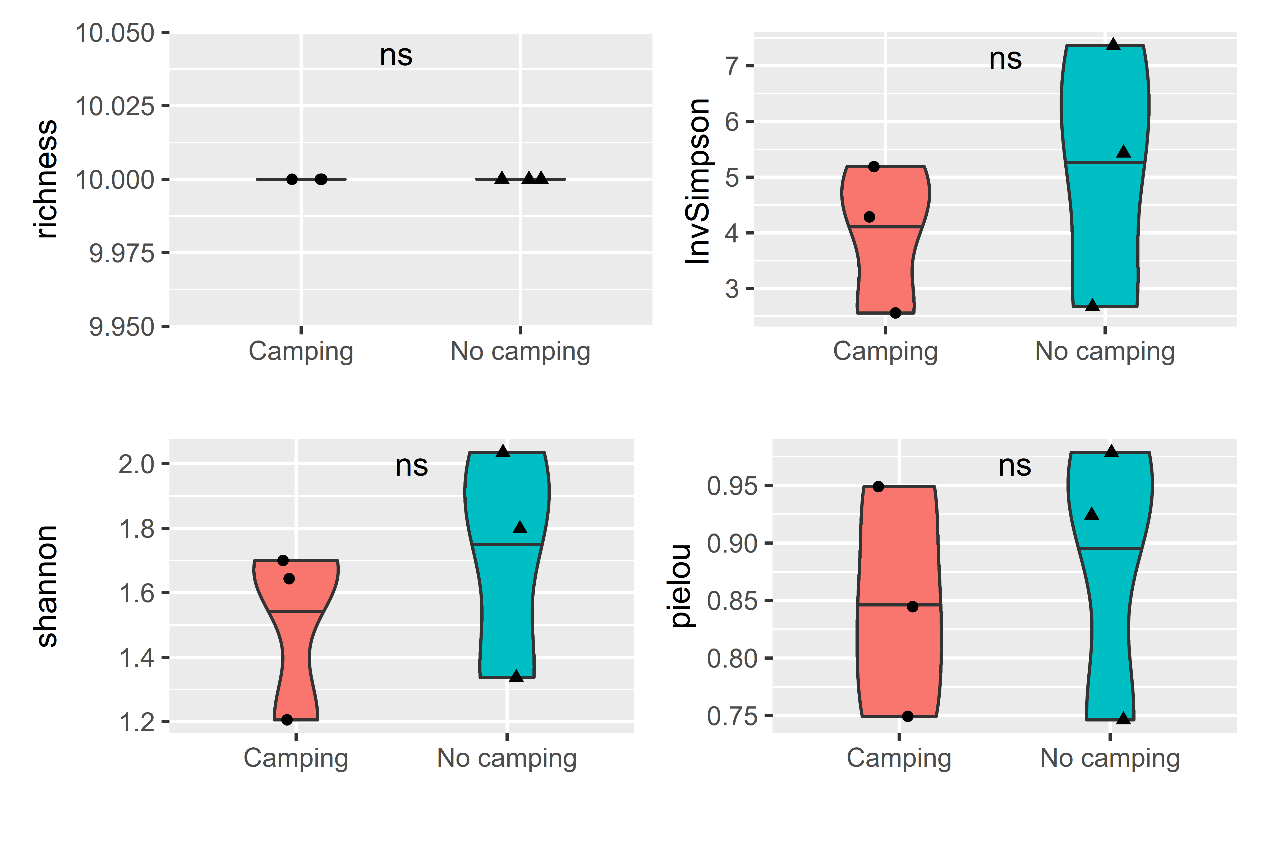


Figure S36. Functional diversities of FAPROTAX1.2.4-based thiosulfate respiration of subsoil bacterial communities under camping and no camping. ns, *p* > 0.05.


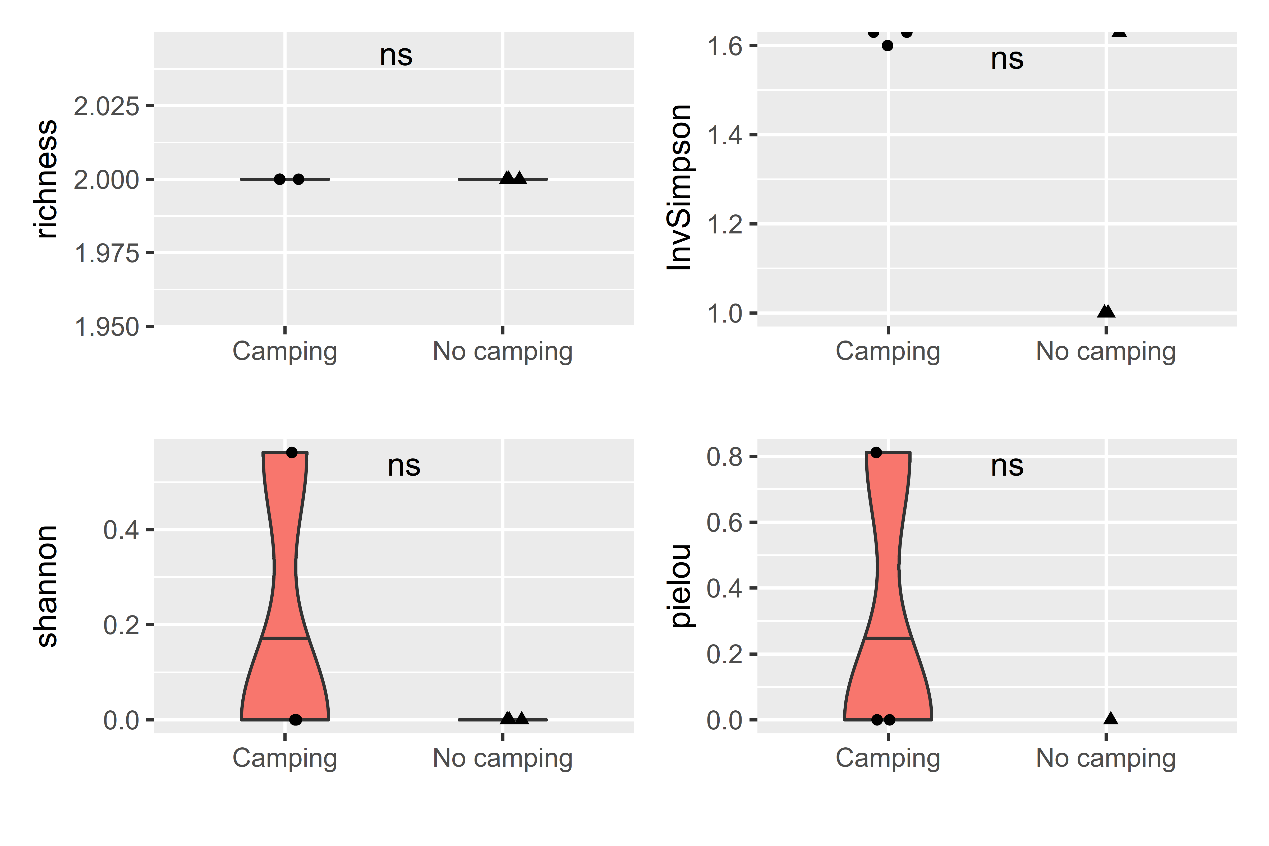


Figure S37. Functional diversities of FAPROTAX1.2.4-based dark thiosulfate oxidation of subsoil bacterial communities under camping and no camping. ns, *p* > 0.05.


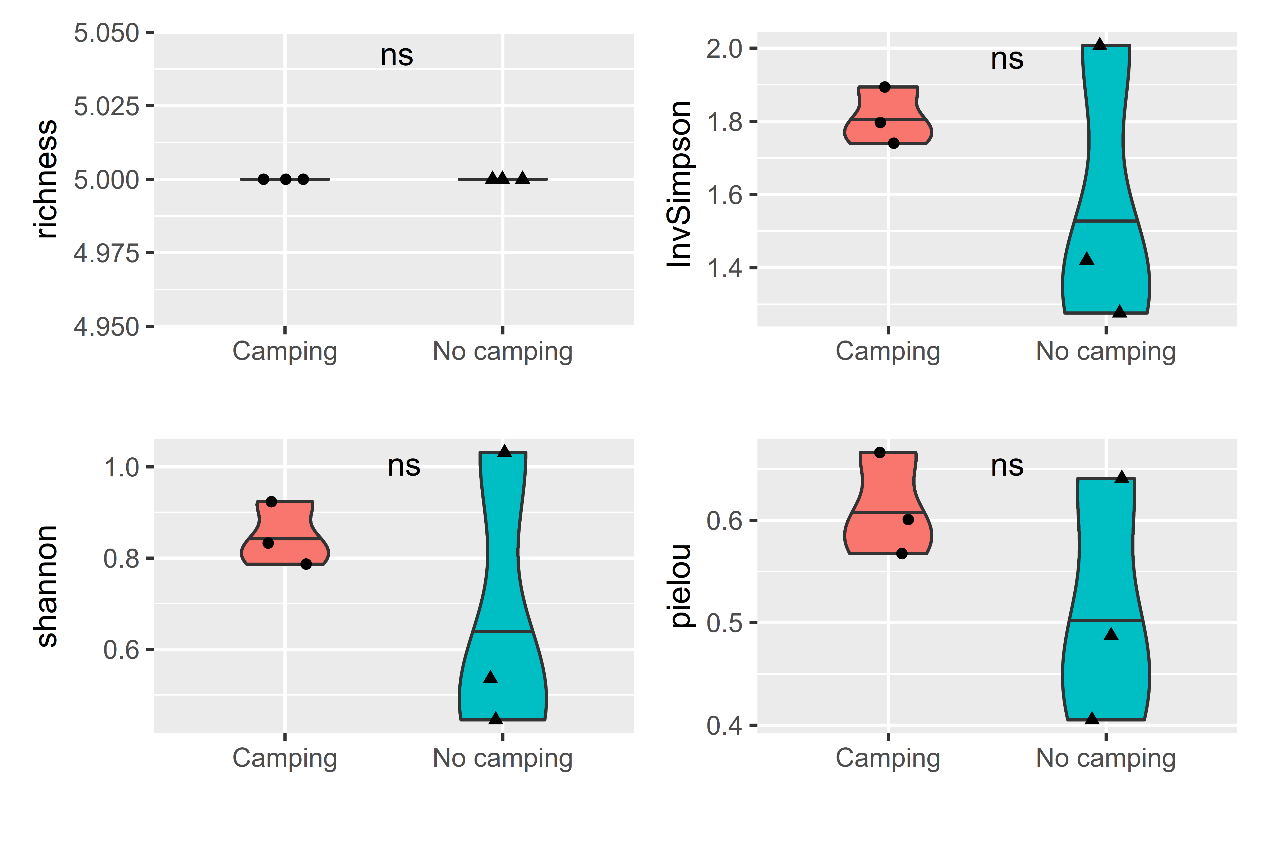


Figure S38. Functional diversities of FAPROTAX1.2.4-based iron respiration of subsoil bacterial communities under camping and no camping. ns, *p* > 0.05.


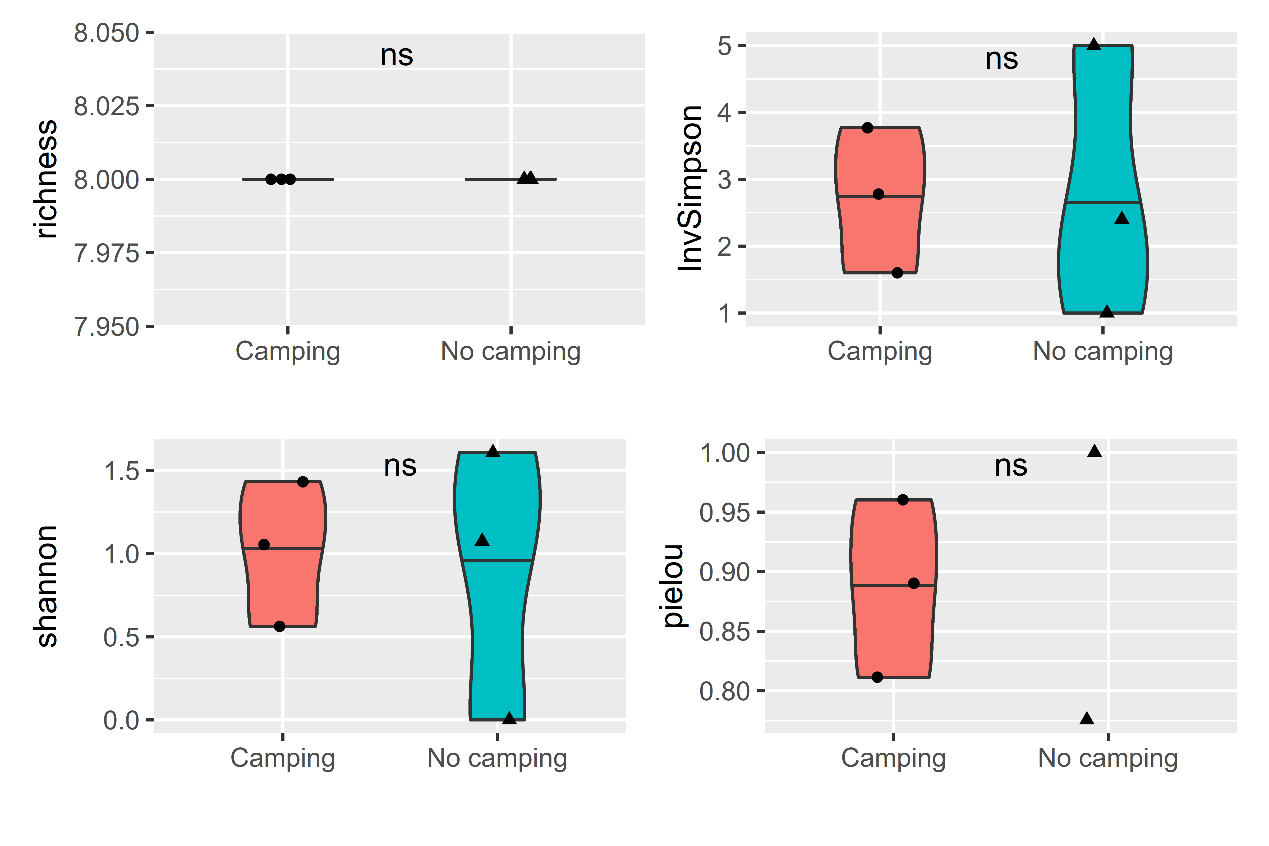


Figure S39. Functional diversities of FAPROTAX1.2.4-based manganese oxidation of subsoil bacterial communities under camping and no camping. ns, *p* > 0.05.


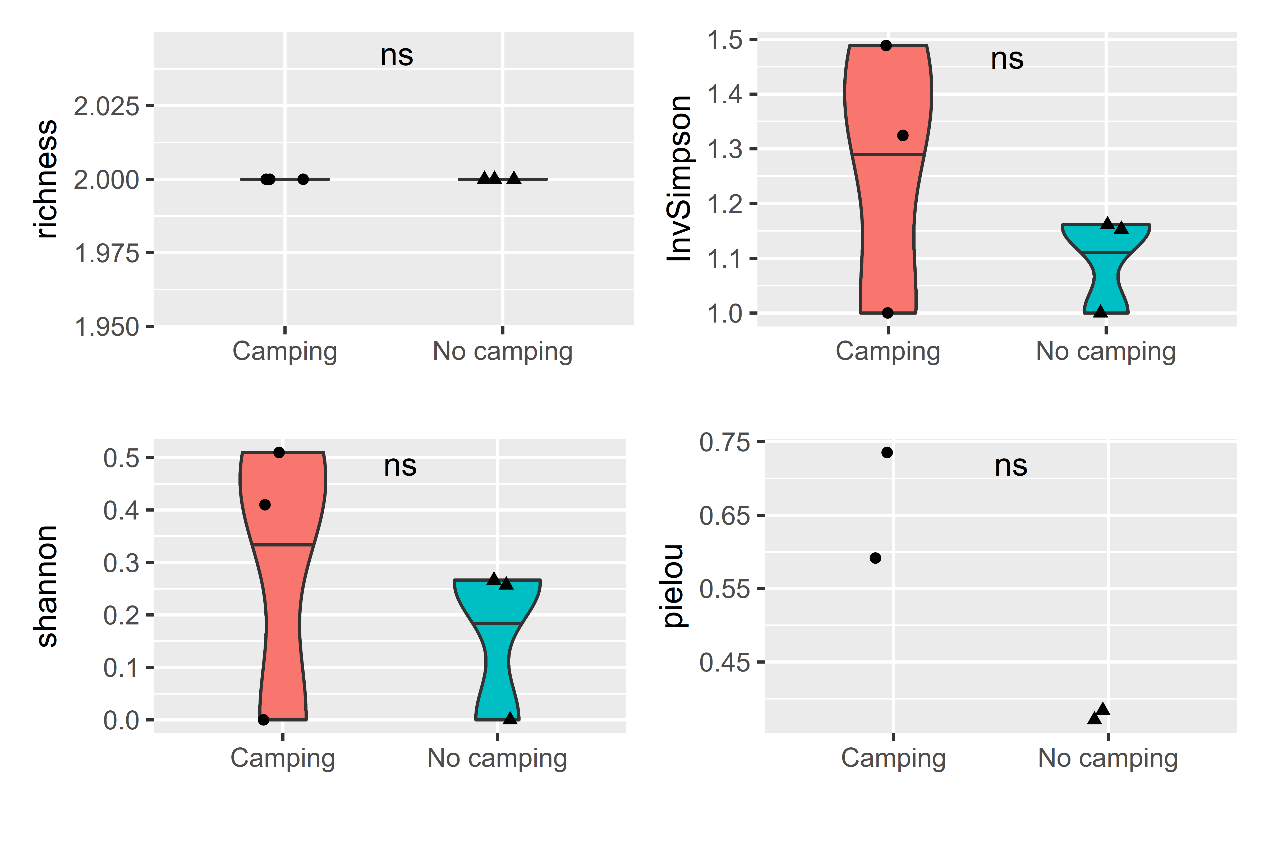


Figure S40. Functional diversities of FAPROTAX1.2.4-based fermentation of subsoil bacterial communities under camping and no camping. ns, *p* > 0.05.


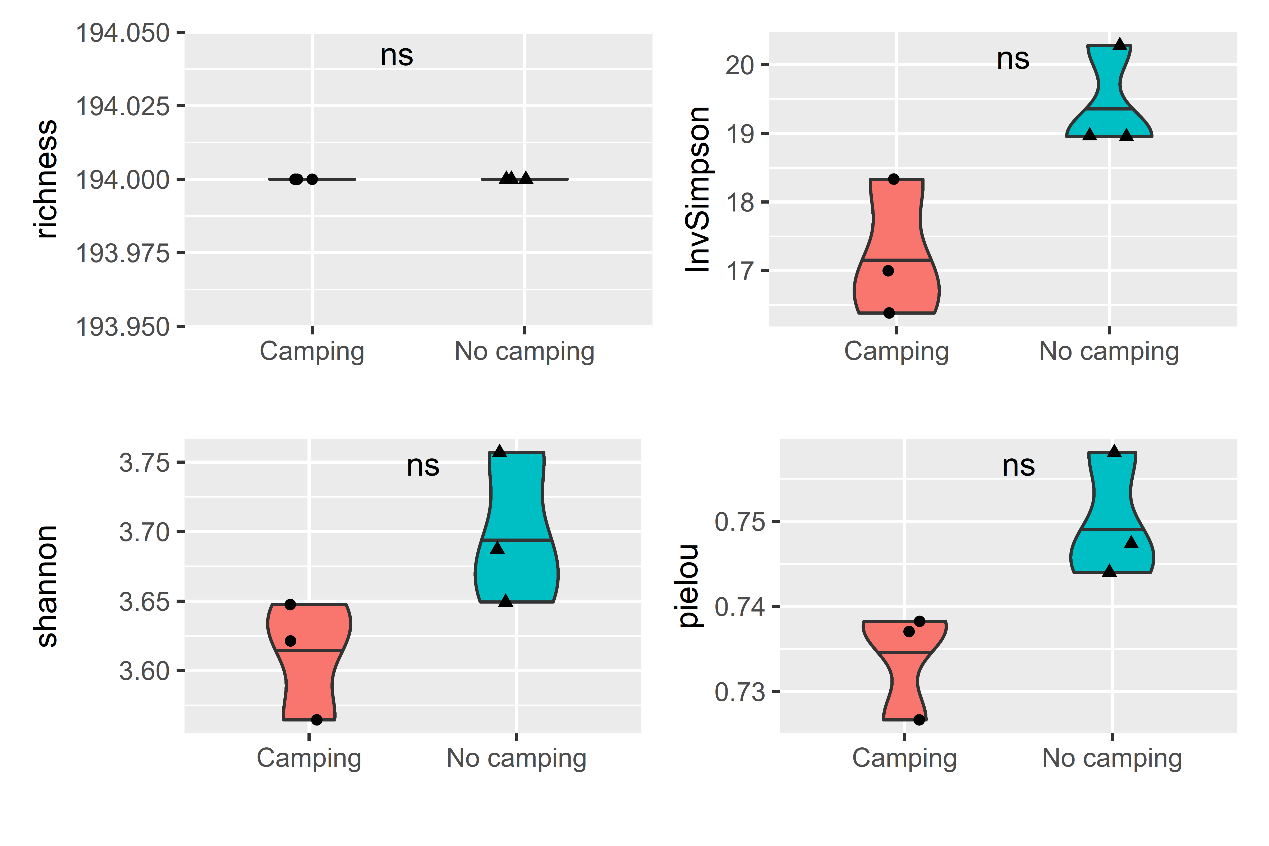


Figure S41. Functional diversities of FAPROTAX1.2.4-based plant pathogen of subsoil bacterial communities under camping and no camping. ns, *p* > 0.05.


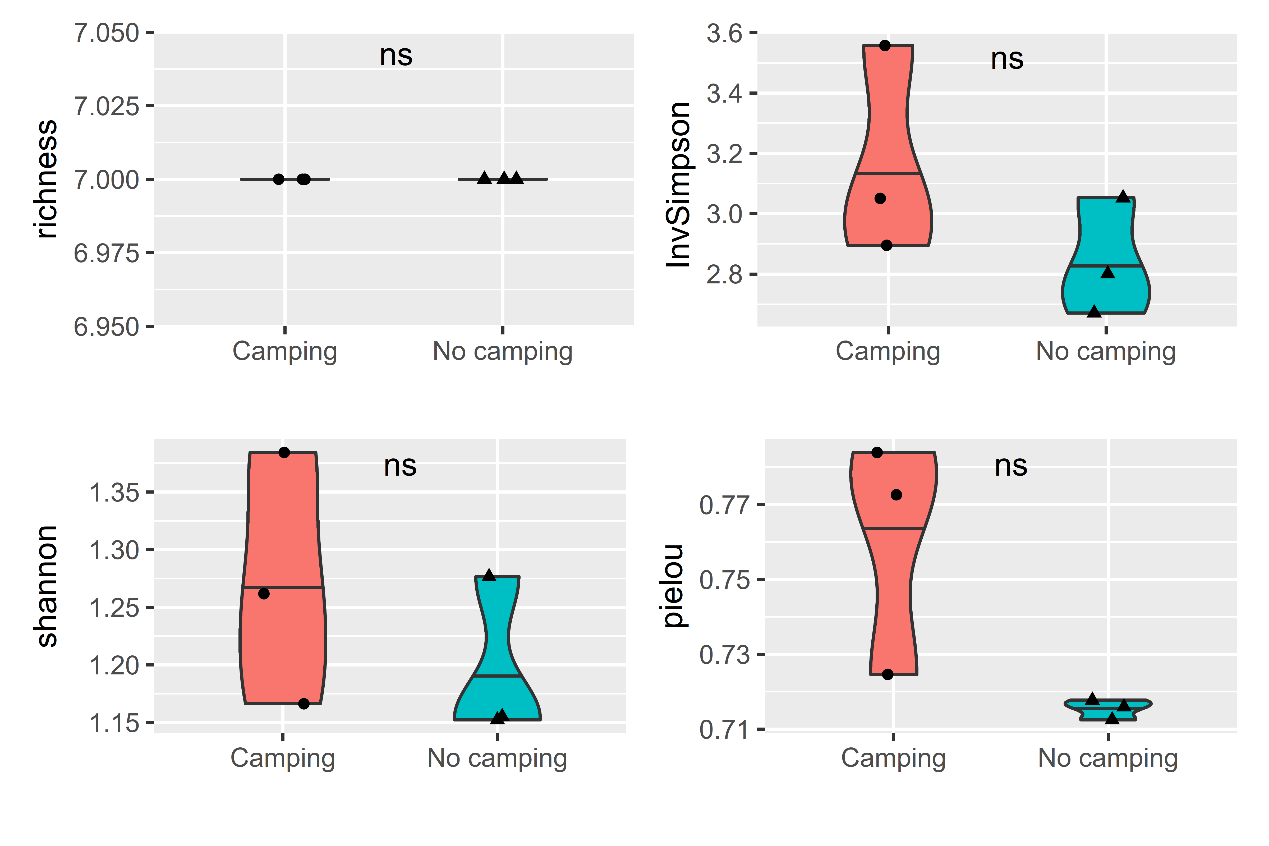


Figure S42. Functional diversities of FAPROTAX1.2.4-based animal parasites or symbionts of subsoil bacterial communities under camping and no camping. ns, *p* > 0.05.


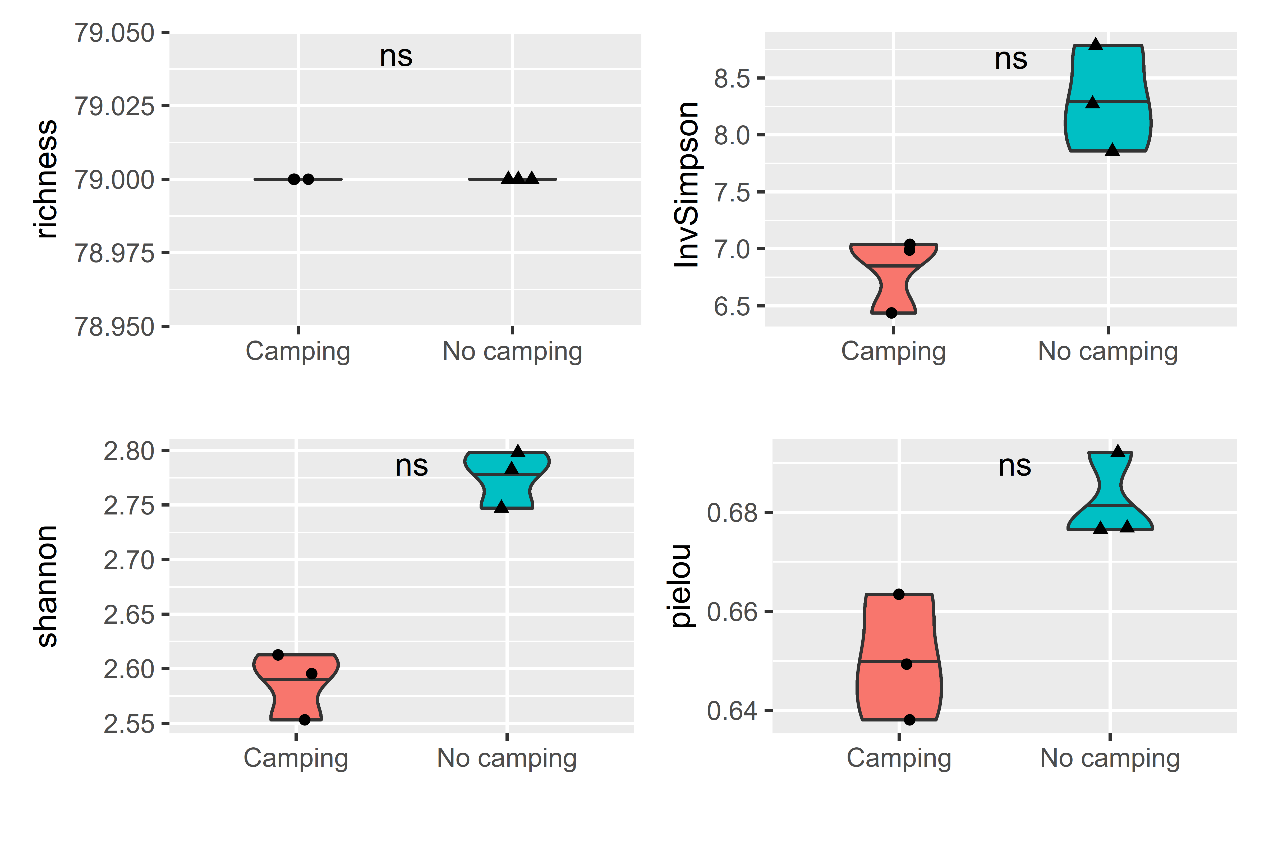


Figure S43. Functional diversities of FAPROTAX1.2.4-based intracellular parasites of subsoil bacterial communities under camping and no camping. ns, *p* > 0.05.


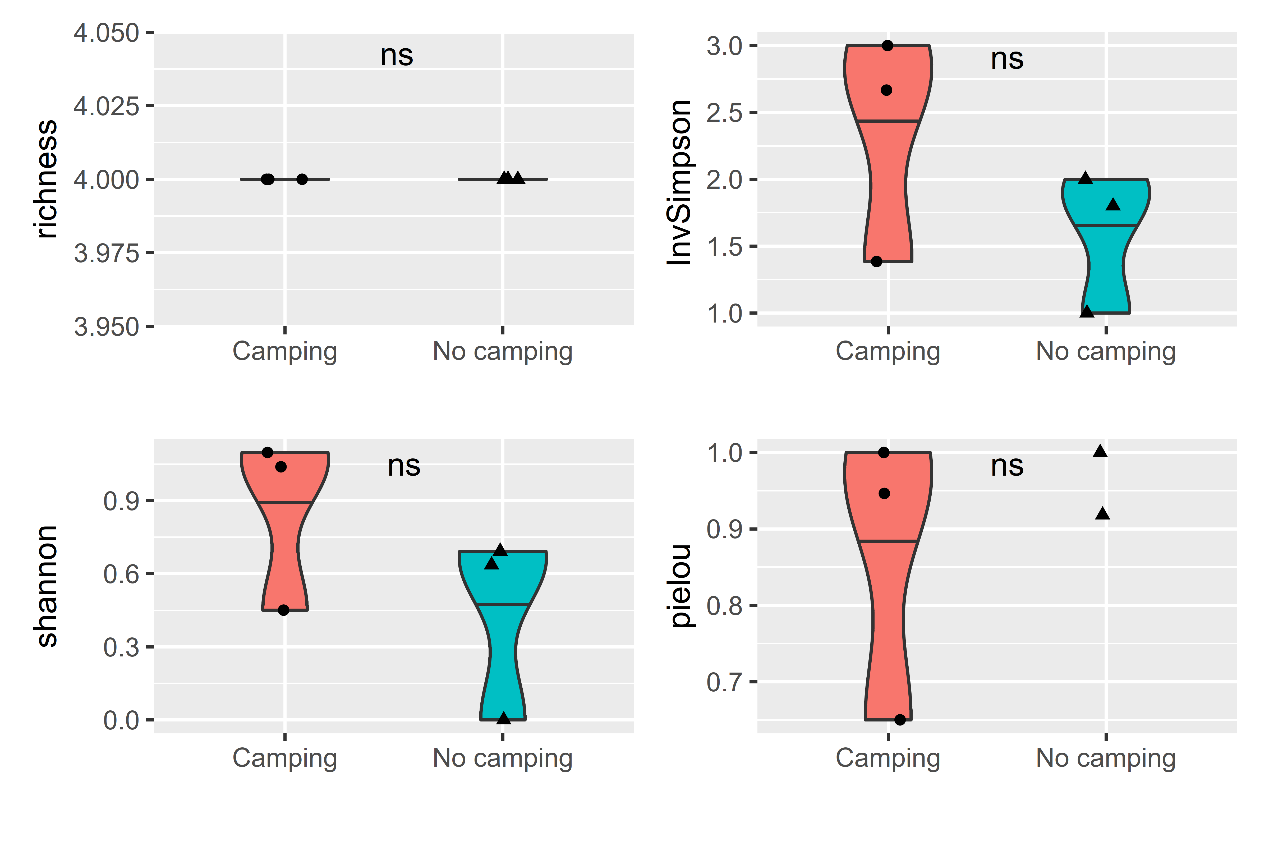


Figure S44. Functional diversities of FAPROTAX1.2.4-based invertebrate parasites of subsoil bacterial communities under camping and no camping. ns, *p* > 0.05.


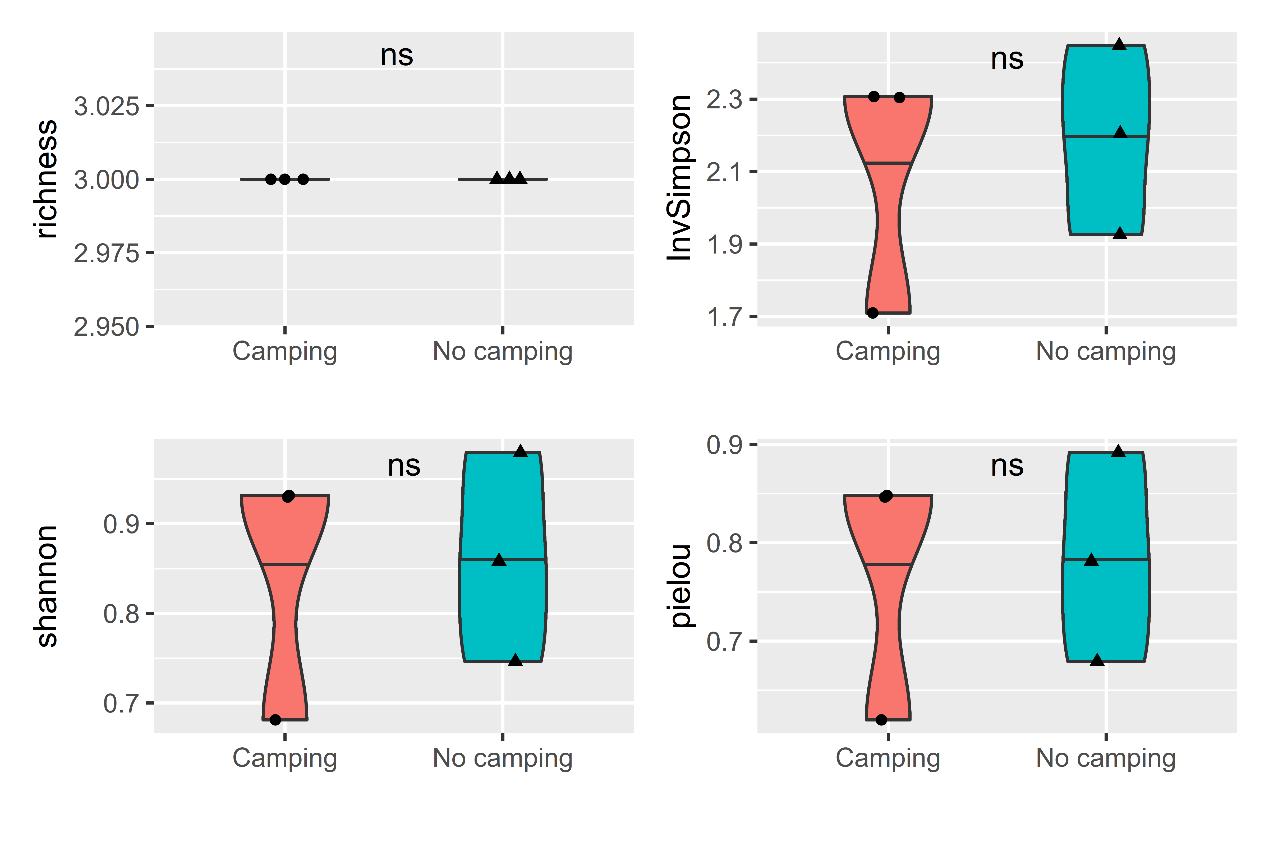


Figure S45. Functional diversities of FAPROTAX1.2.4-based predatory or exoparasitic bacteria of subsoil under camping and no camping. ns, *p* > 0.05.


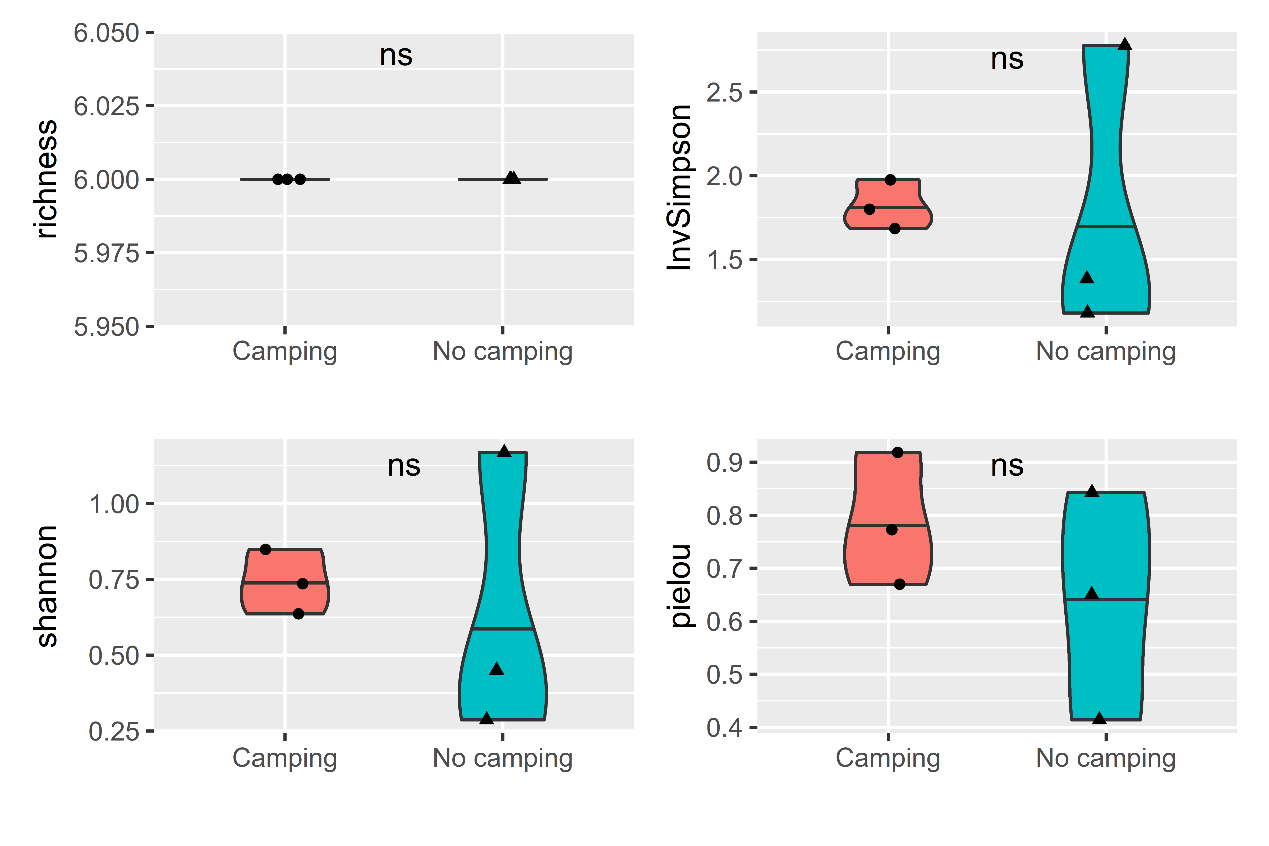


Figure S46. Functional diversities of FAPROTAX1.2.4-based mammal gut of subsoil bacterial communities under camping and no camping. ns, *p* > 0.05.


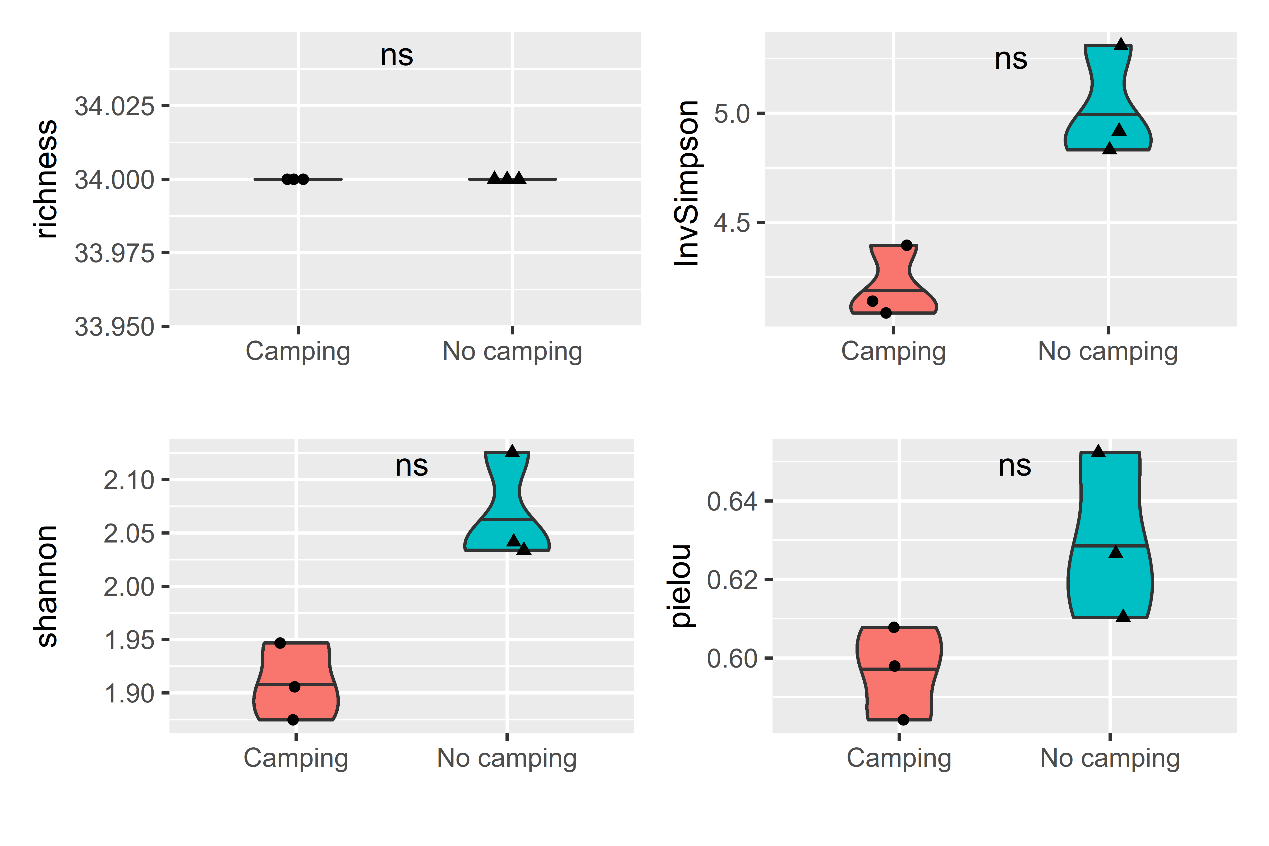


Figure S47. Functional diversities of FAPROTAX1.2.4-based chemoheterotrophy of subsoil bacterial communities under camping and no camping. ns, *p* > 0.05.


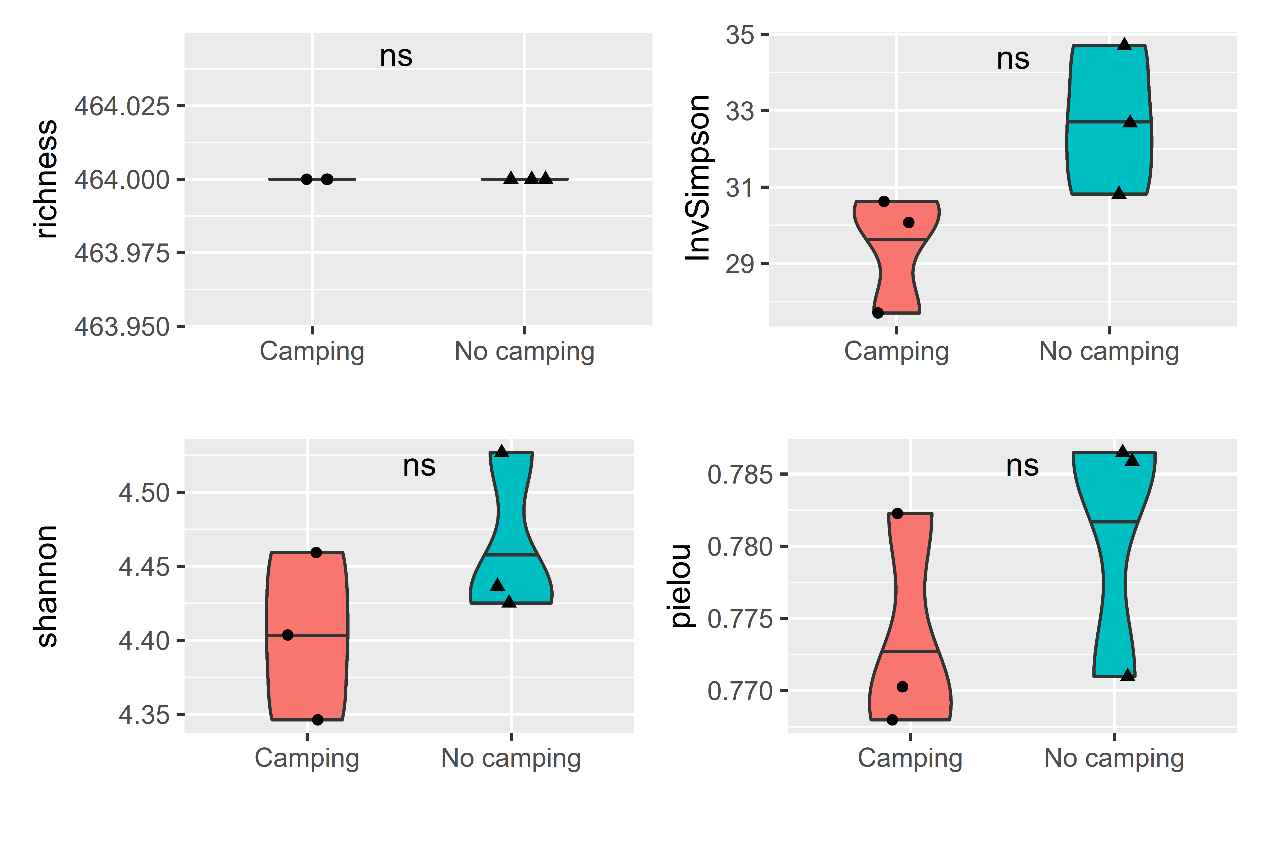


Figure S48. Functional diversities of FAPROTAX1.2.4-based aerobic chemoheterotrophy of subsoil bacterial communities under camping and no camping. ns, *p* > 0.05.


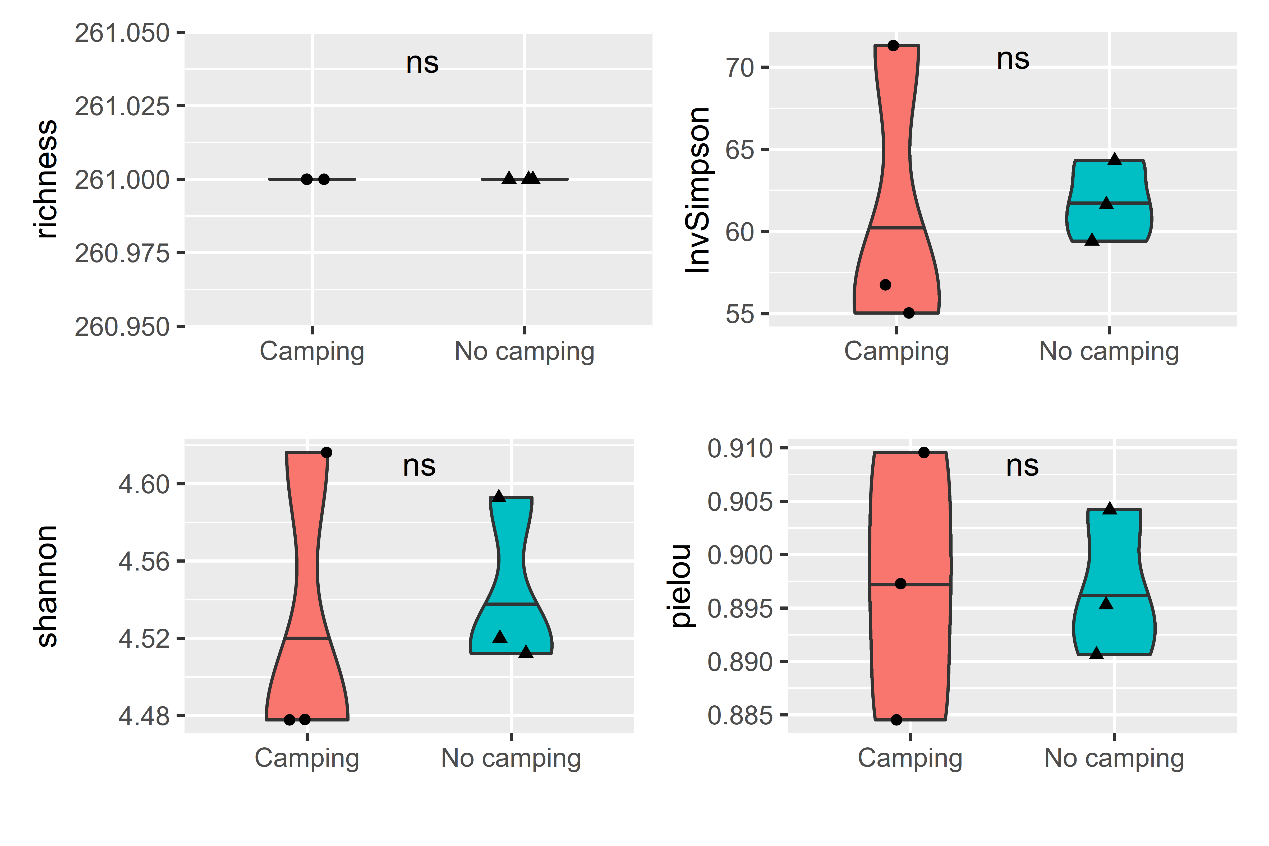


Figure S49. Functional diversities of FAPROTAX1.2.4-based nonphotosynthetic cyanobacteria of subsoil bacterial communities under camping and no camping. ns, *p* > 0.05.


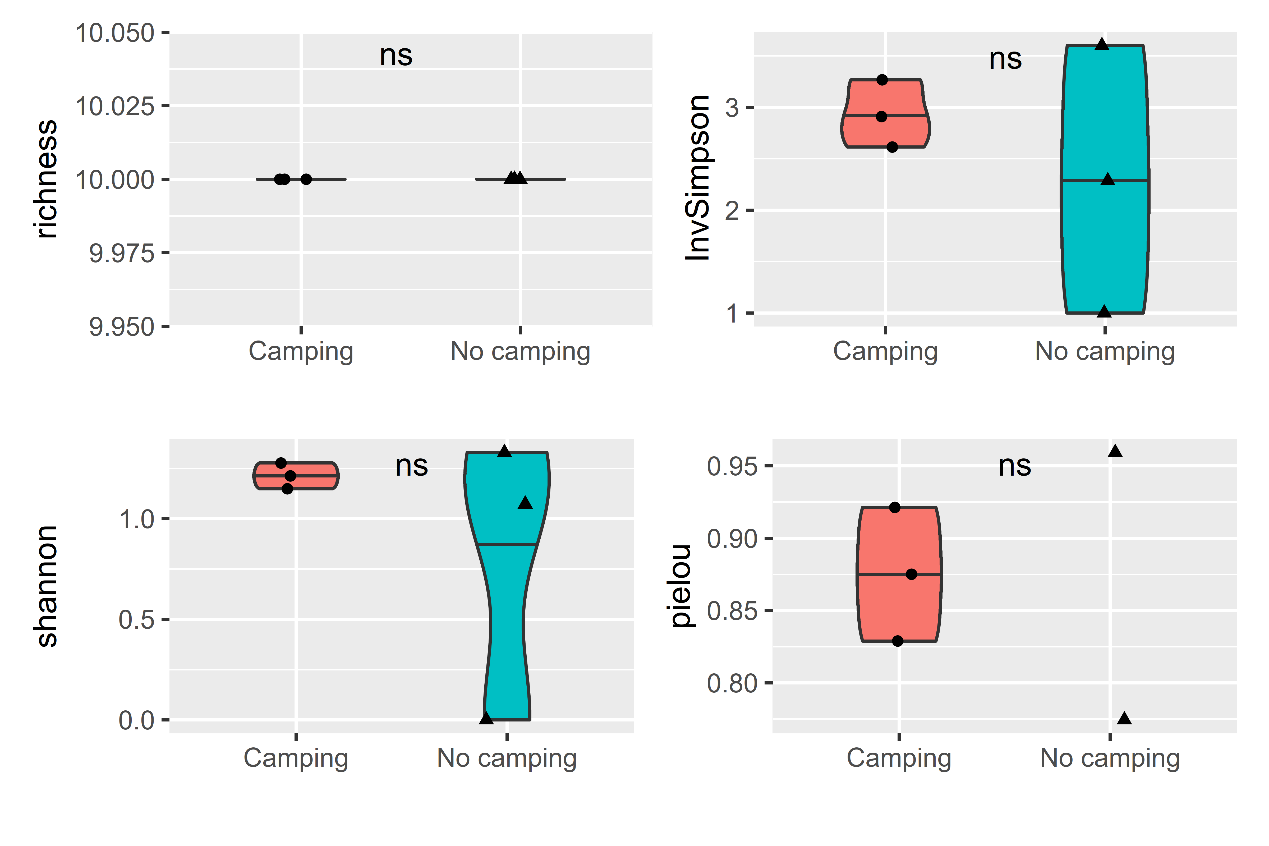


Figure S50. Functional diversities of FAPROTAX1.2.4-based oxygenic photoautotrophy of subsoil bacterial communities under camping and no camping. ns, *p* > 0.05.


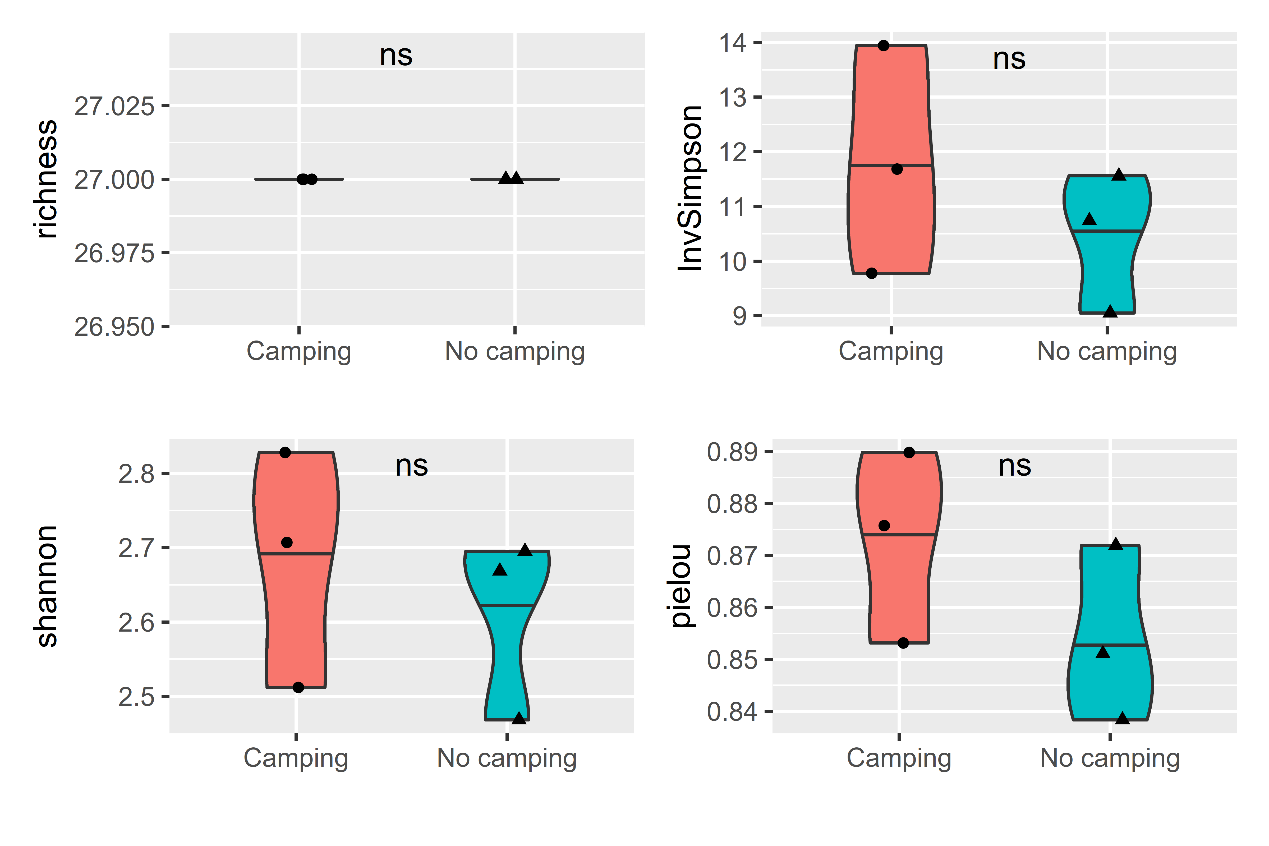


Figure S51. Functional diversities of FAPROTAX1.2.4-based photoautotrophy of subsoil bacterial communities under camping and no camping. ns, *p* > 0.05.


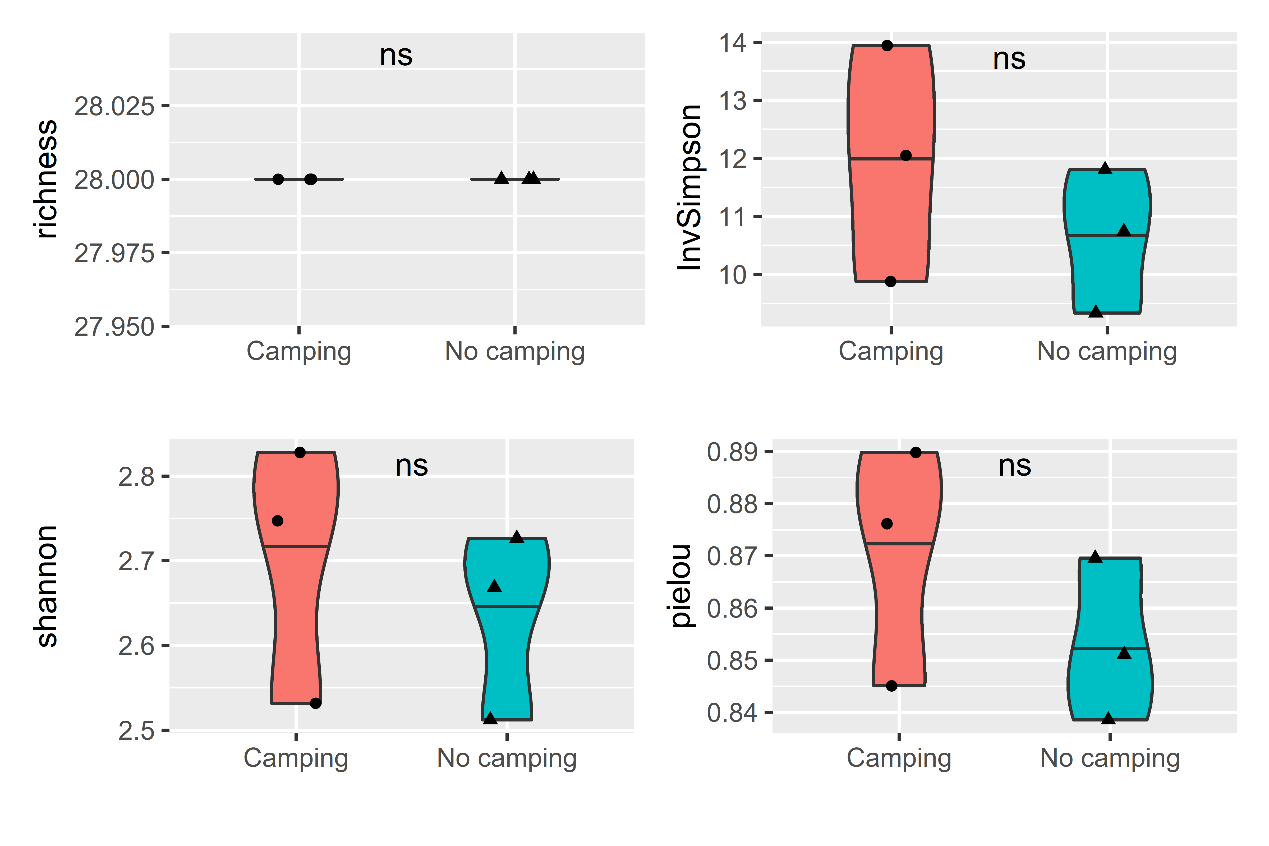


Figure S52. Functional diversities of FAPROTAX1.2.4-based photoheterotrophy of subsoil bacterial communities under camping and no camping. ns, *p* > 0.05.


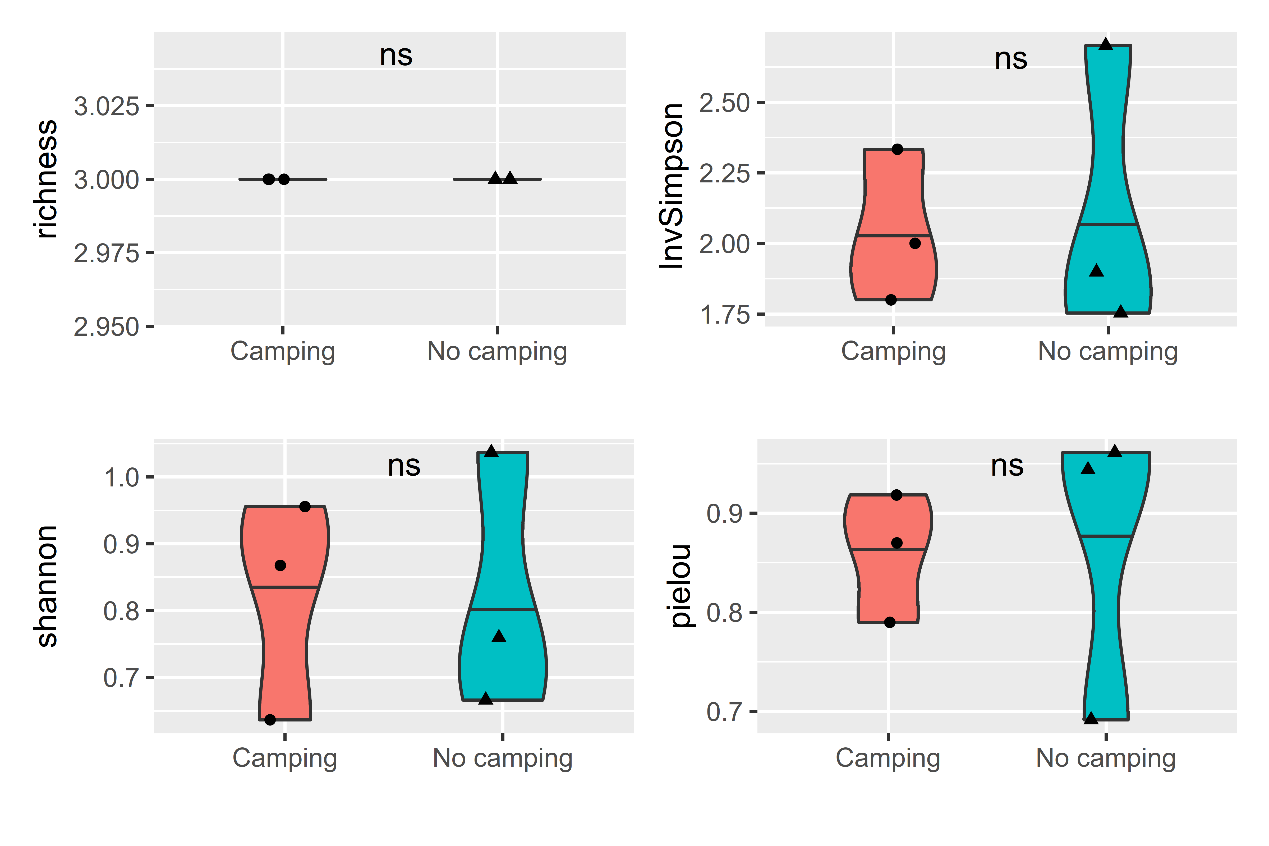


Figure S53. Functional diversities of FAPROTAX1.2.4-based photosynthetic cyanobacteria of subsoil bacterial communities under camping and no camping. ns, *p* > 0.05.


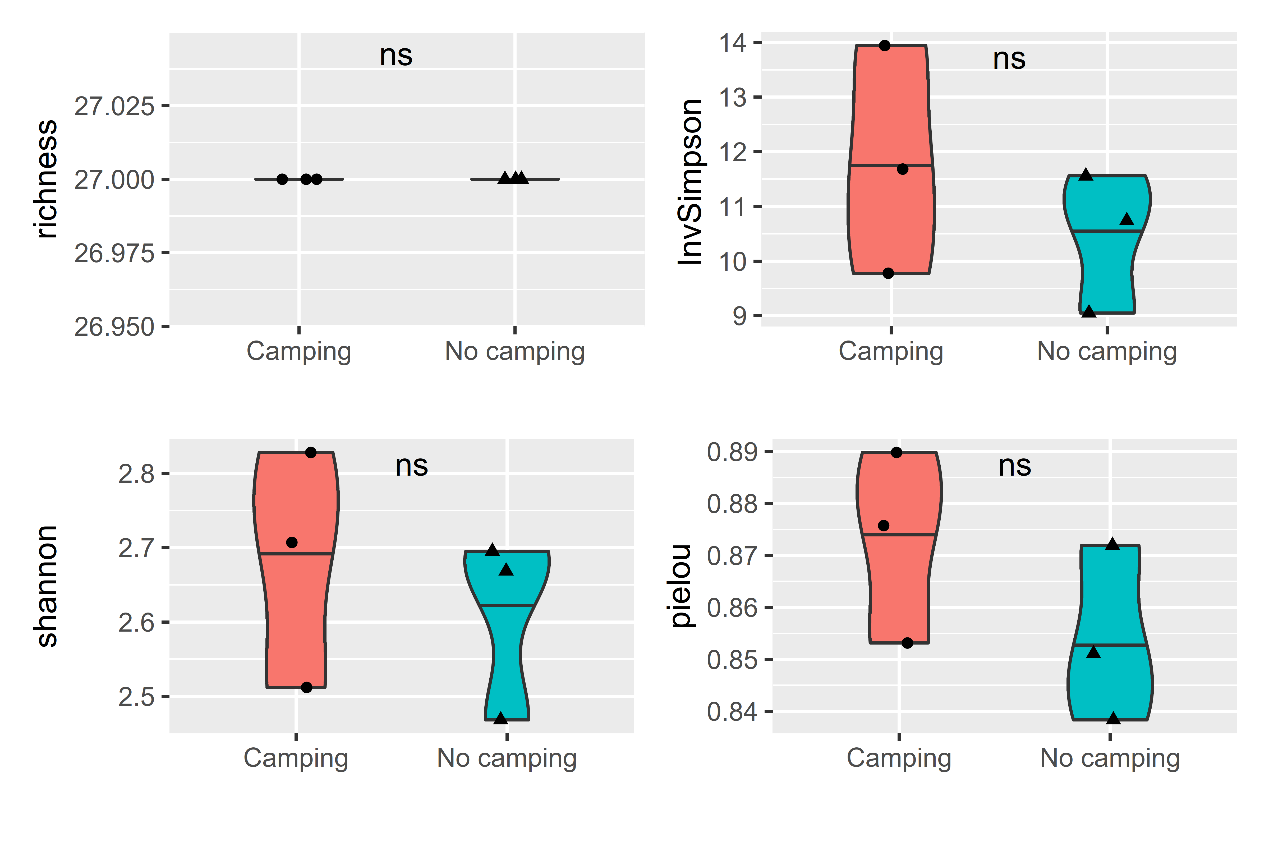


Figure S54. Functional diversities of FAPROTAX1.2.4-based phototrophy of subsoil bacterial communities under camping and no camping. ns, *p* > 0.05.


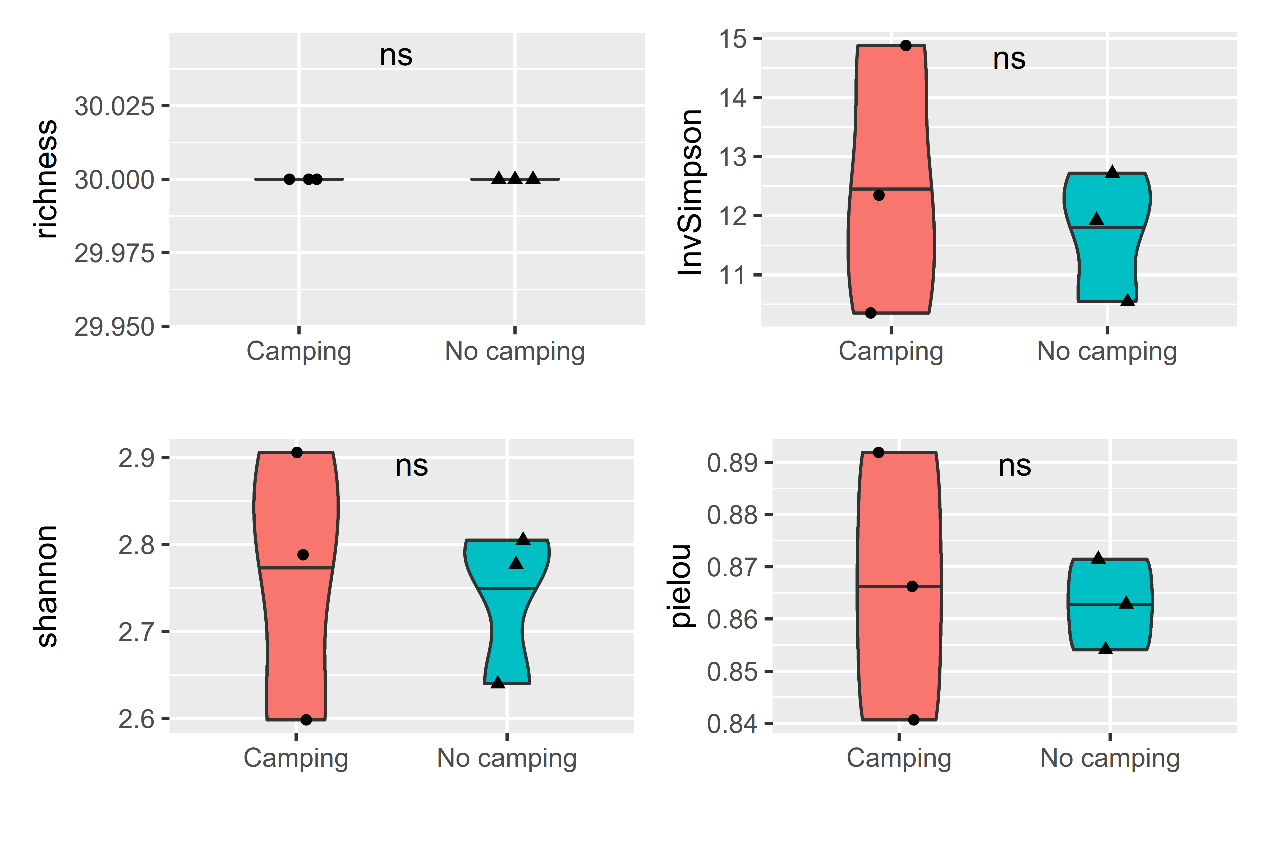


Figure S55. FAPROTAX1.2.4-based functional diversities of subsoil bacterial communities under camping and no camping at the overall level. ns, *p* > 0.05.


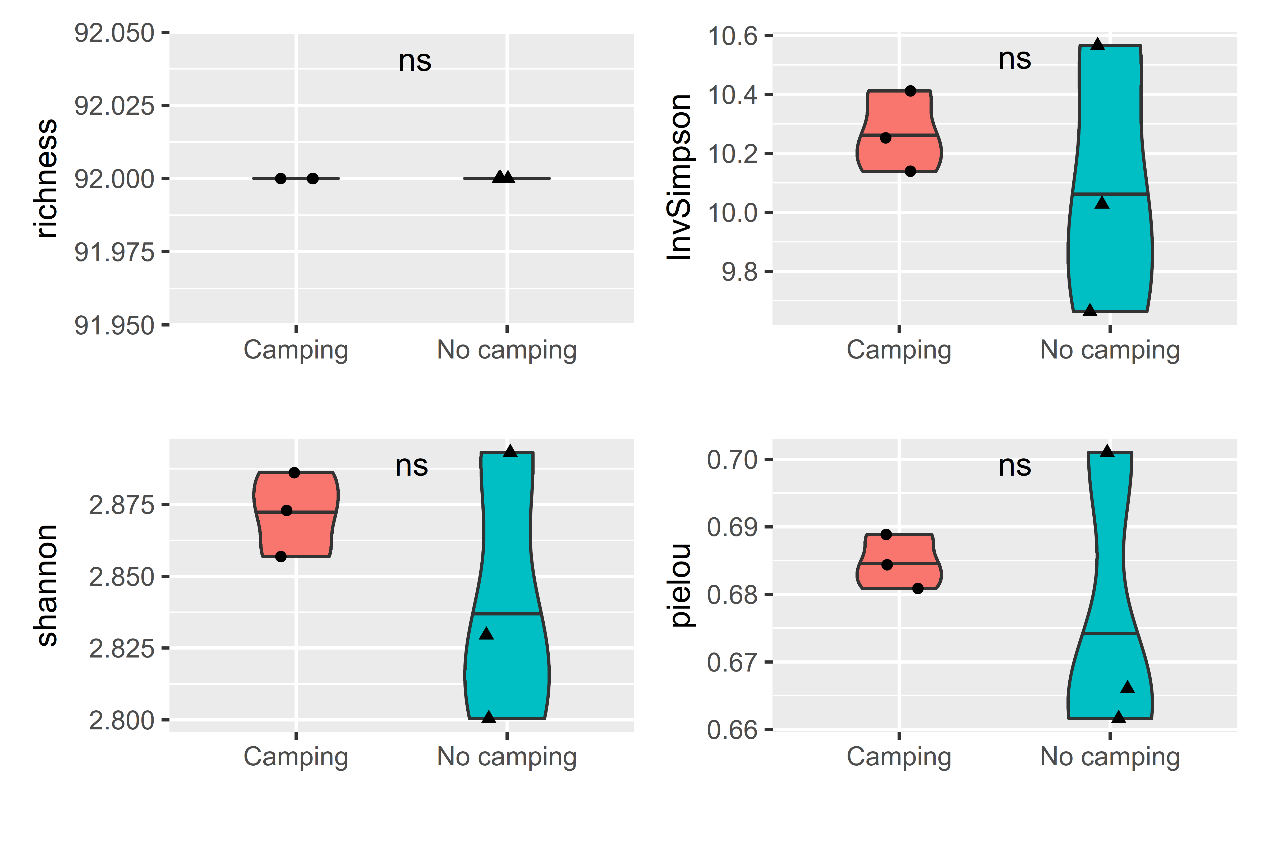


Figure S56. Diversities of Tax4Fun-based functional genes of subsoil bacterial communities under camping and no camping. ns, *p* > 0.05.


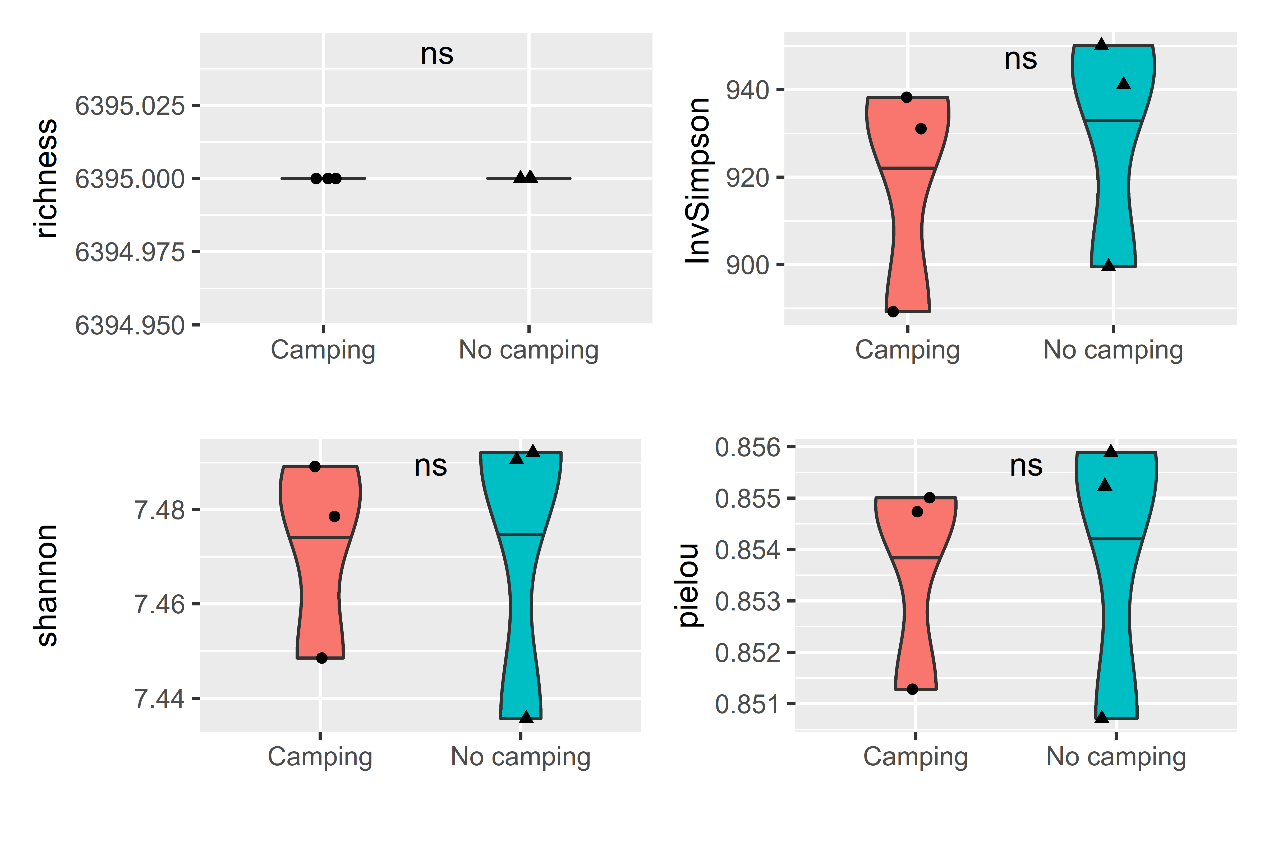


Figure S57. Diversities of Tax4Fun-based C genes of subsoil bacterial communities under camping and no camping. ns, *p* > 0.05.


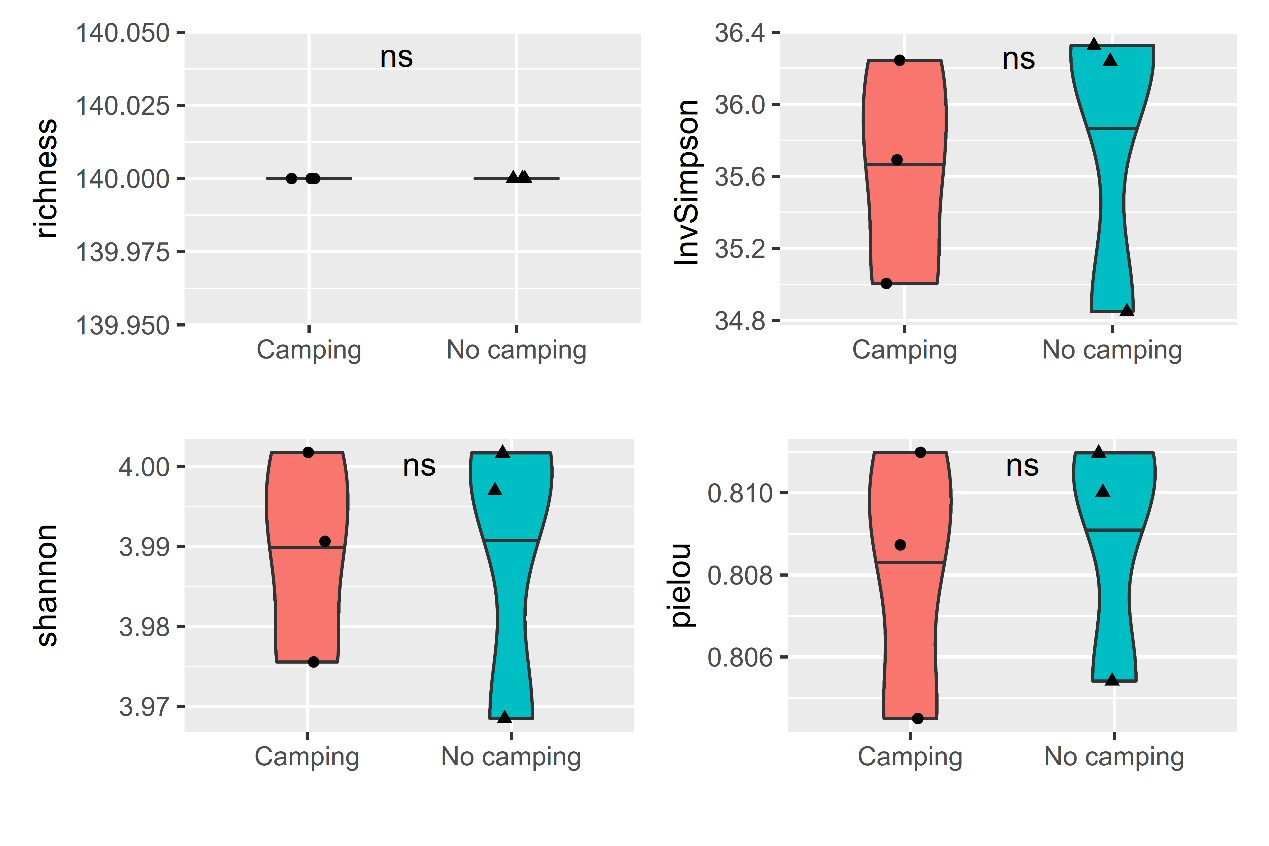


Figure S58. Diversities of Tax4Fun-based C fixation genes of subsoil bacterial communities under camping and no camping. ns, *p* > 0.05.


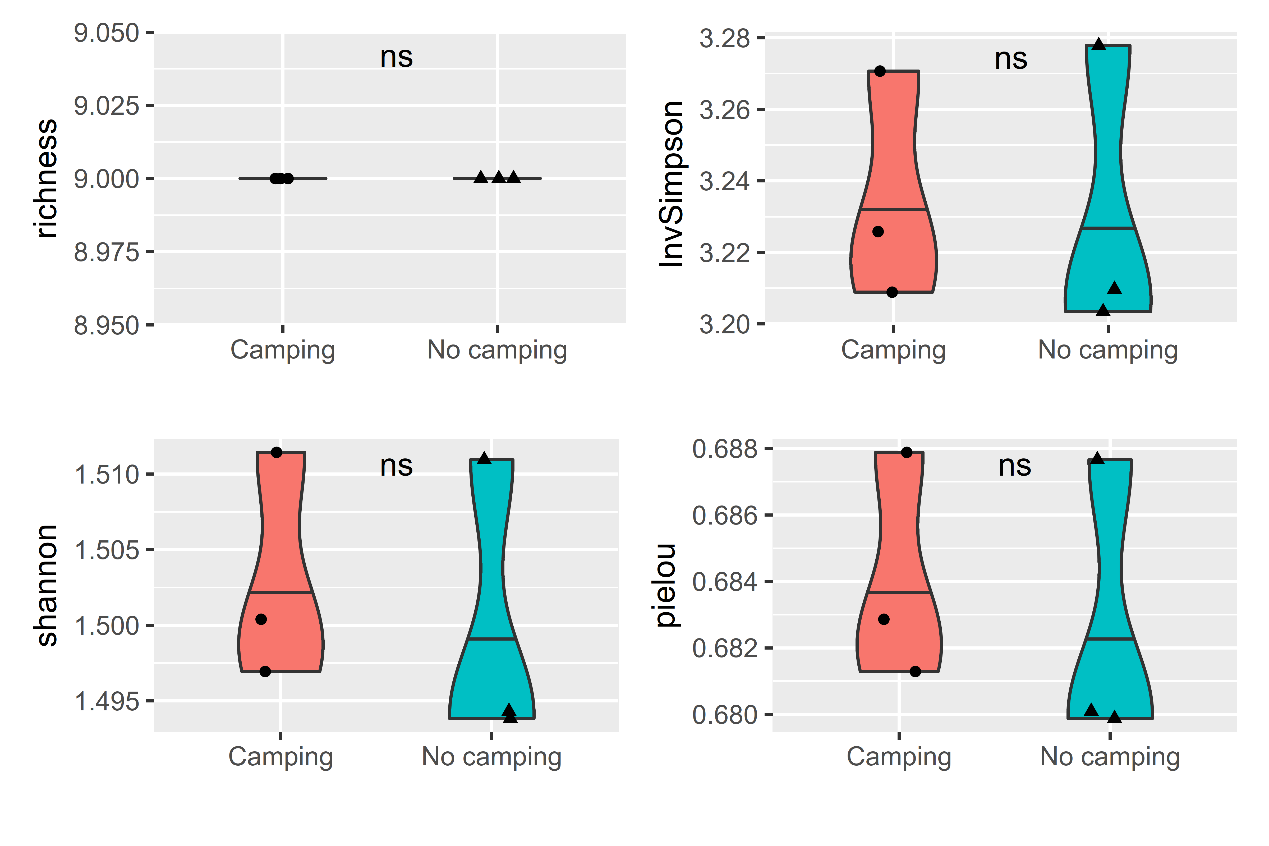


Figure S59. Diversities of Tax4Fun-based C decomposition genes of subsoil bacterial communities under camping and no camping. ns, *p* > 0.05.


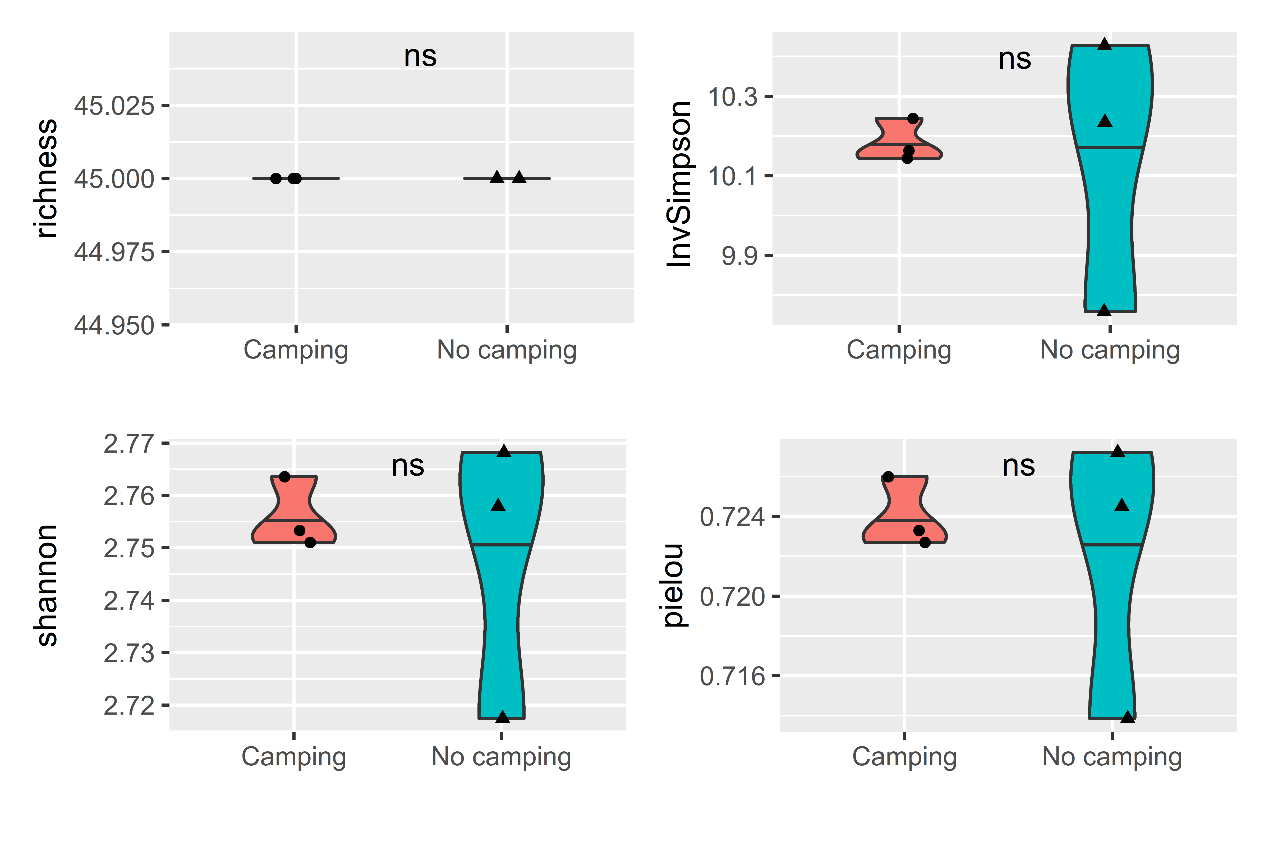


Figure S60. Diversities of Tax4Fun-based N genes of subsoil bacterial communities under camping and no camping. ns, *p* > 0.05.


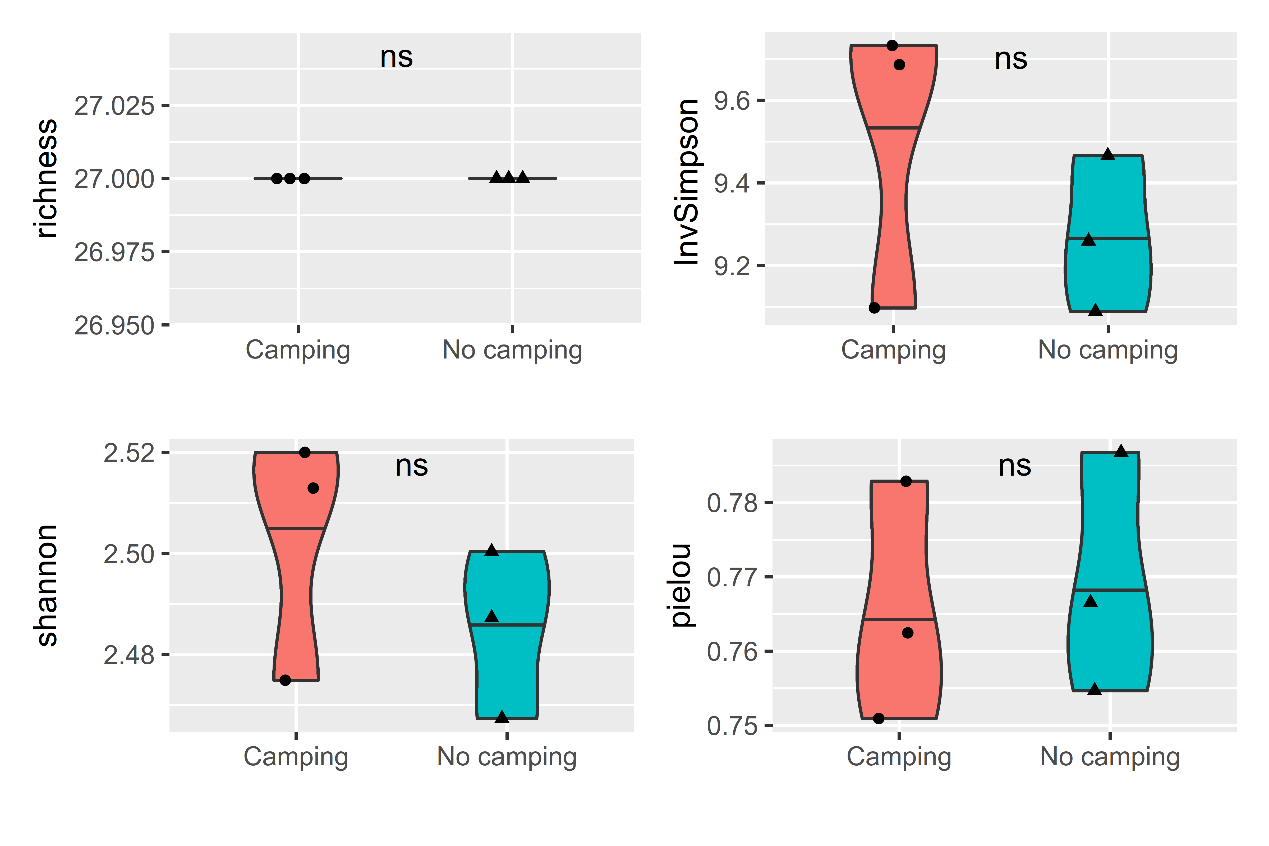


Figure S61. Diversities of Tax4Fun-based N fixation genes of subsoil bacterial communities under camping and no camping. ns, *p* > 0.05.


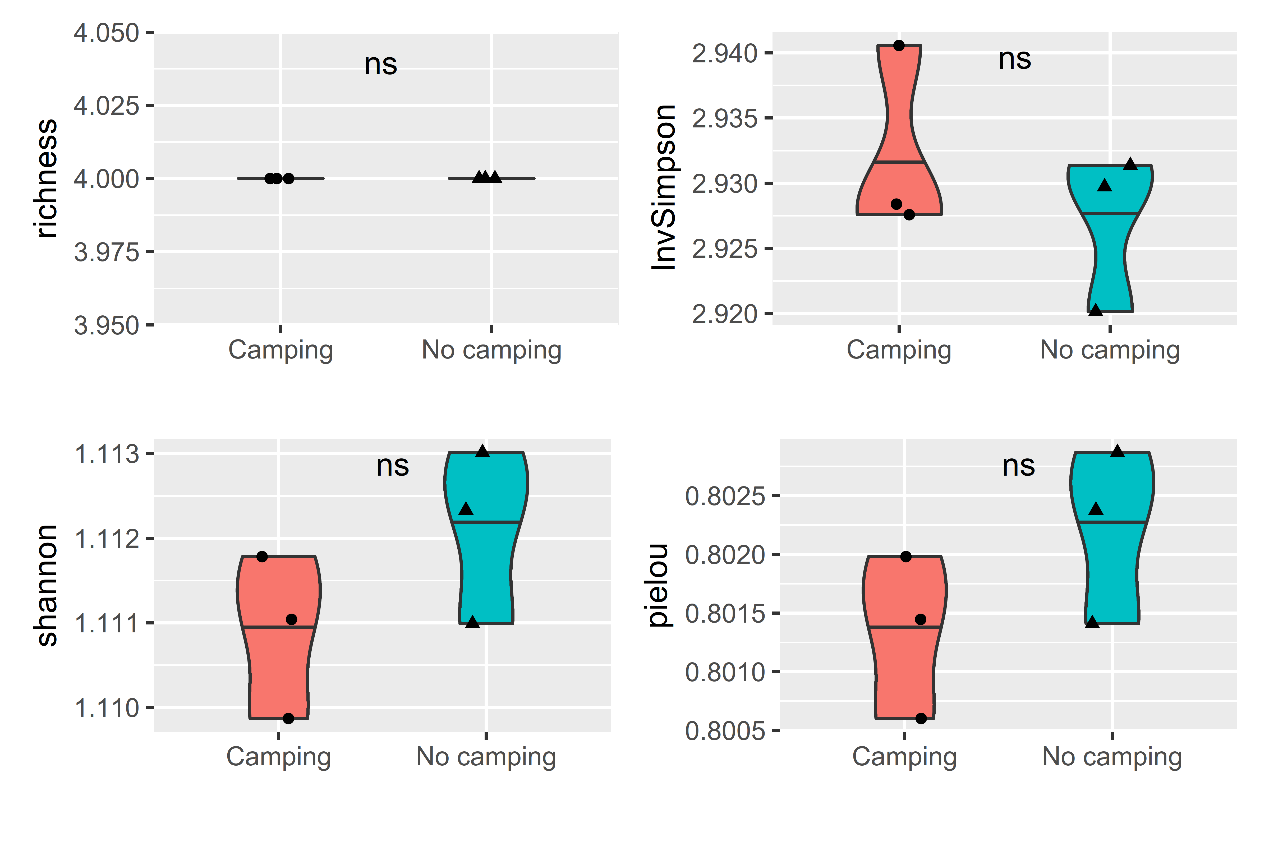


Figure S62 Diversities of Tax4Fun-based N assimilatory nitrate reduction genes of subsoil bacterial communities under camping and no camping. ns, *p* > 0.05.


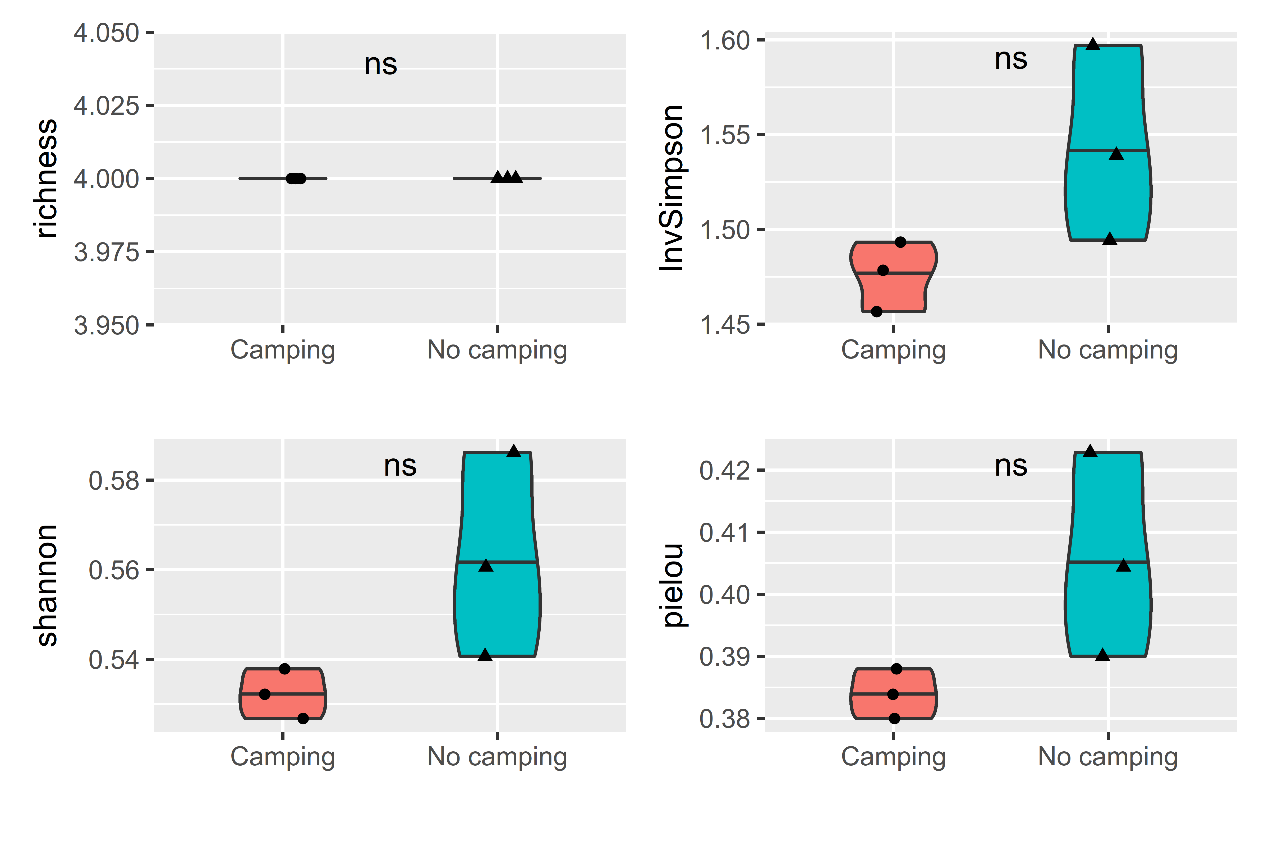


Figure S63 Diversities of Tax4Fun-based complete nitrification genes of subsoil bacterial communities under camping and no camping. ns, *p* > 0.05.


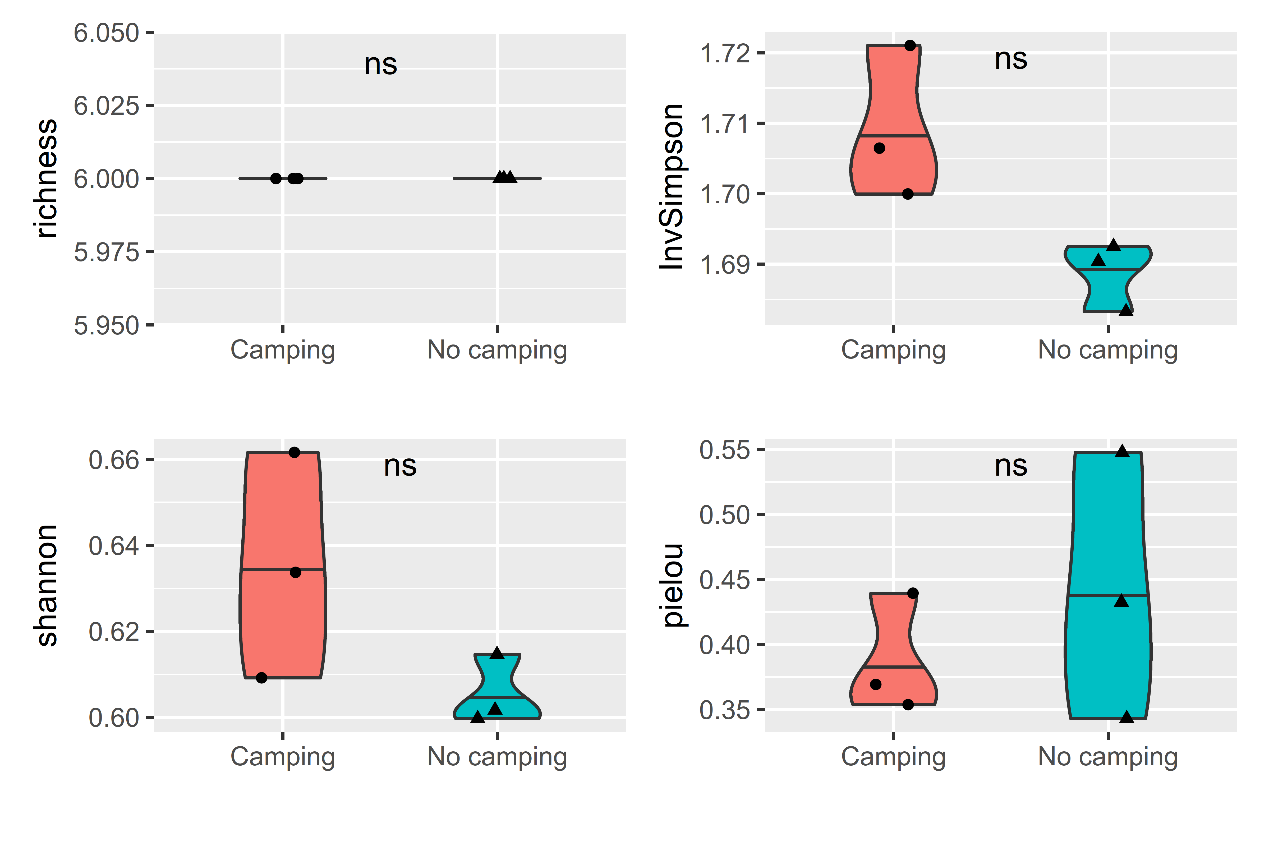


Figure S64. Diversities of Tax4Fun-based denitrification genes of subsoil bacterial communities under camping and no camping. ns, *p* > 0.05.


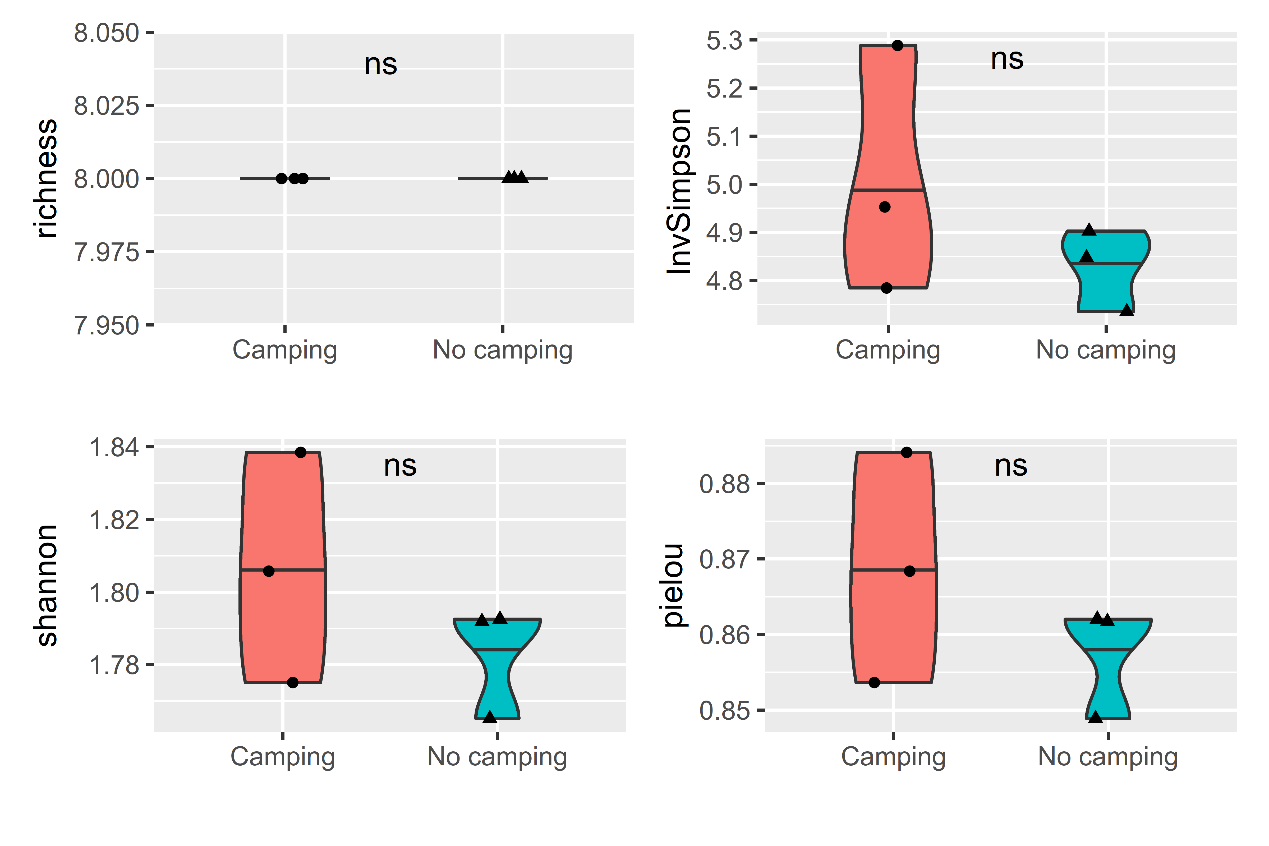


Figure S65. Diversities of Tax4Fun-based dissimilatory nitrate reduction genes of subsoil bacterial communities under camping and no camping. ns, *p* > 0.05.


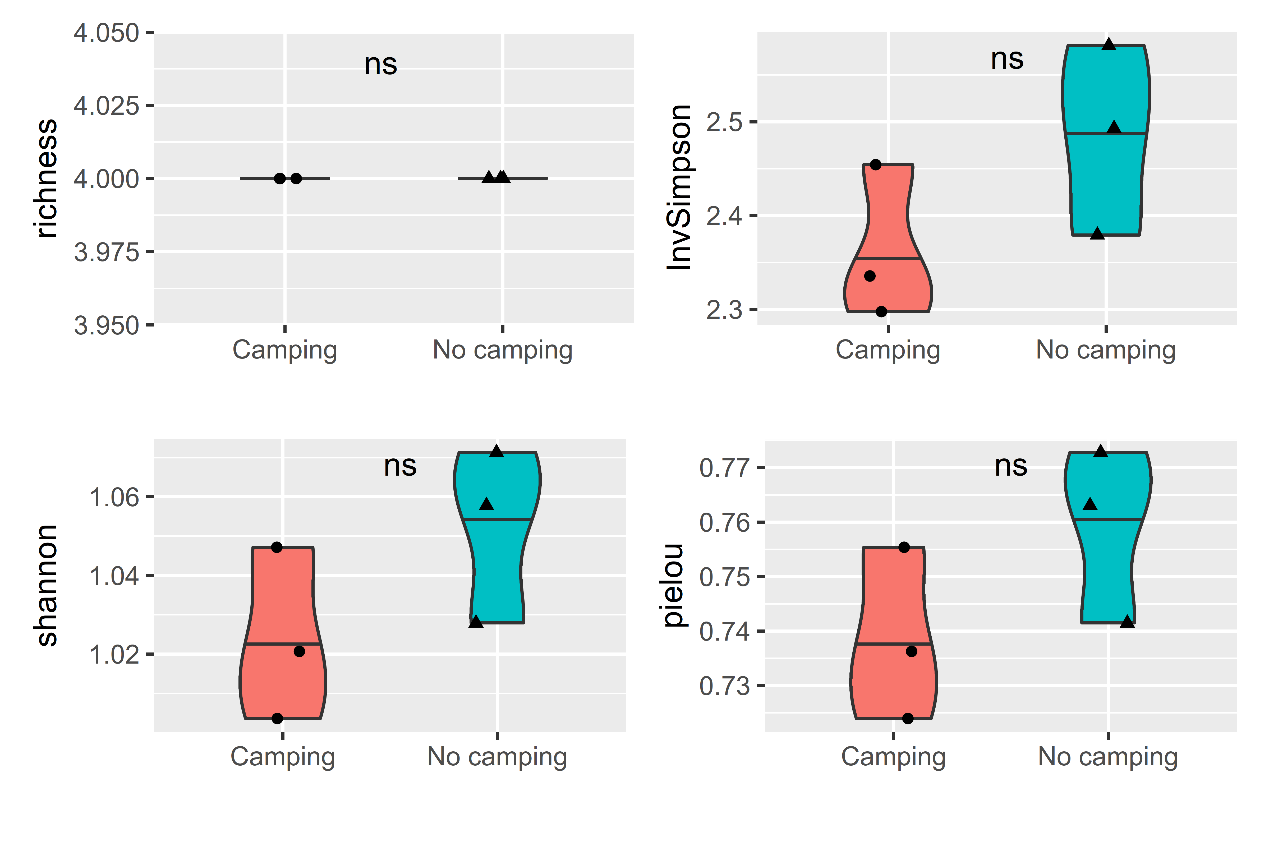


Figure S66. Diversities of Tax4Fun-based P genes of subsoil bacterial communities under camping and no camping. ns, *p* > 0.05.


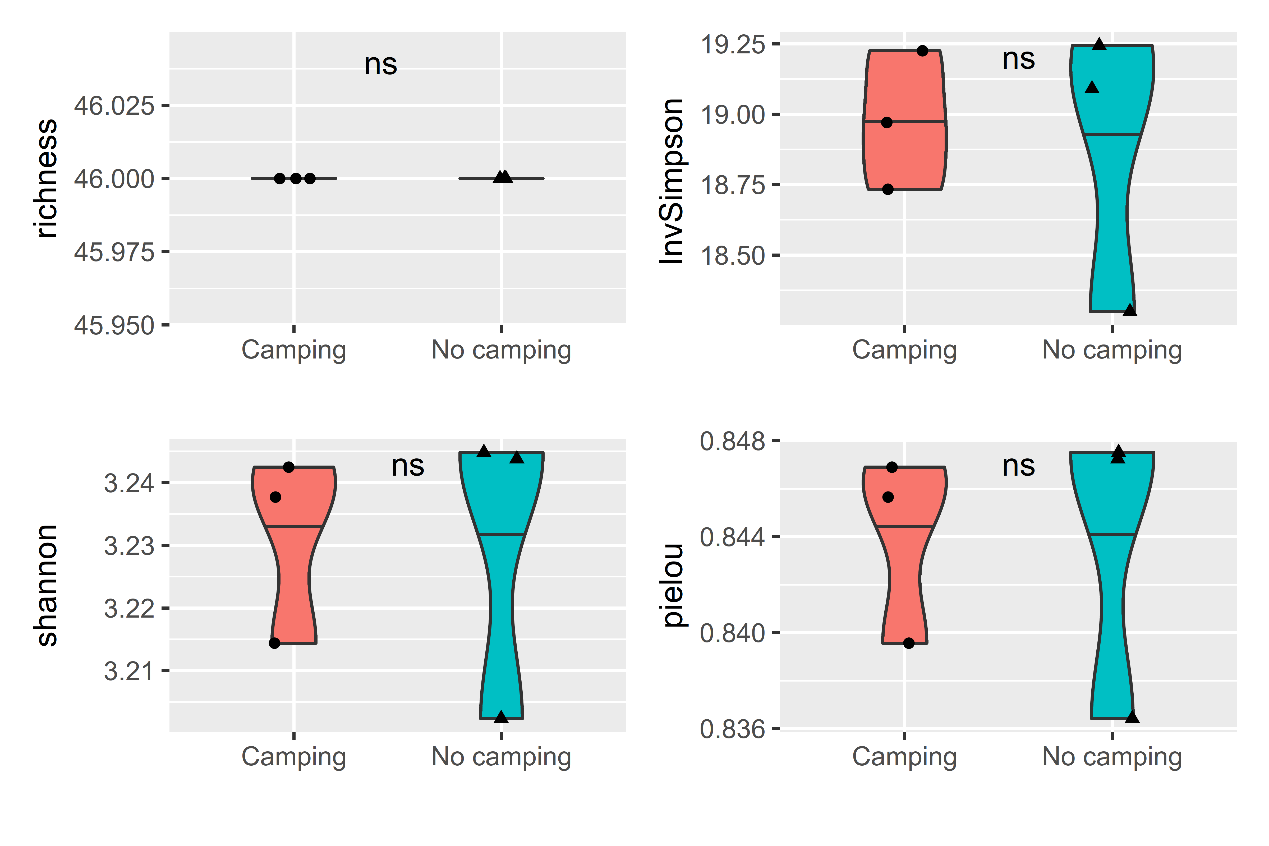


Figure S67. Diversities of Tax4Fun-based P starvation response regulation genes of subsoil bacterial communities under camping and no camping. ns, *p* > 0.05.


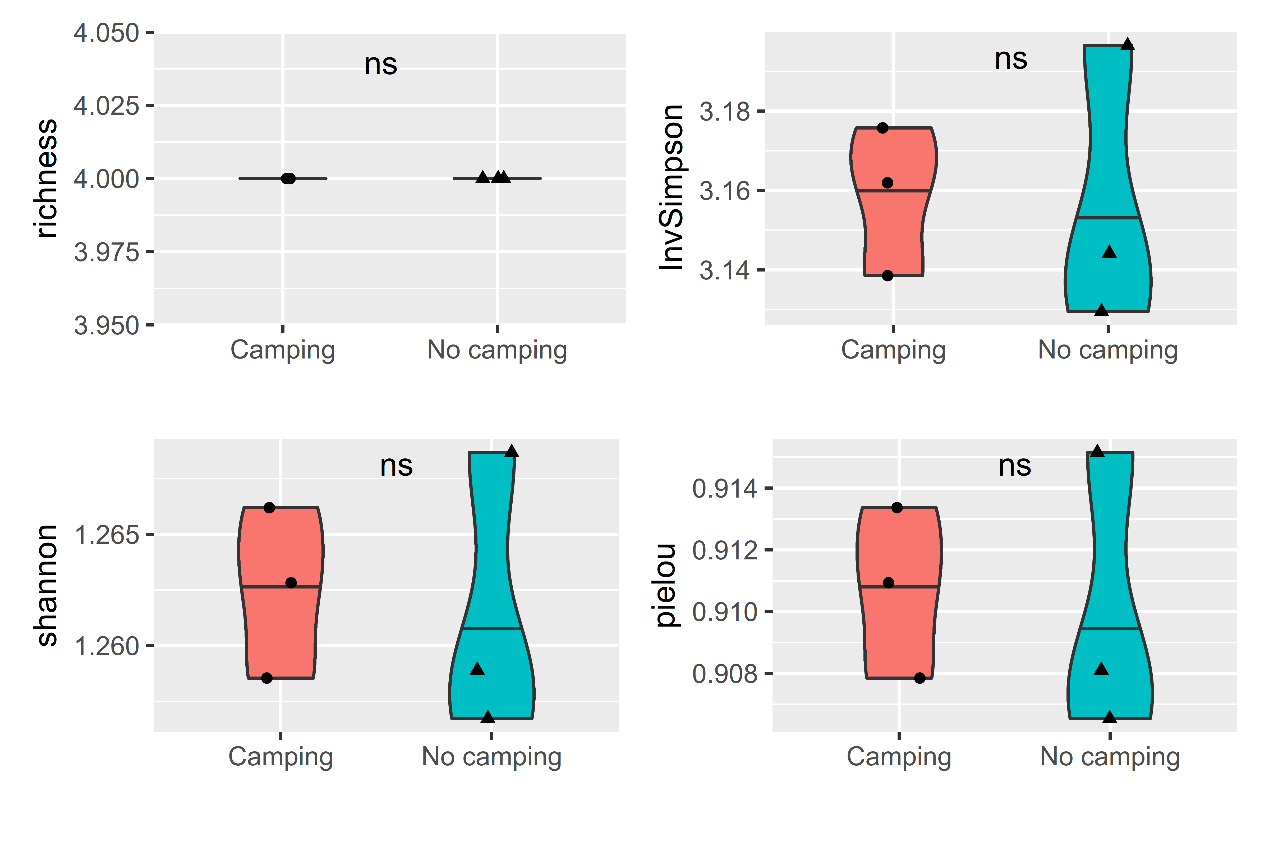


Figure S68. Diversities of Tax4Fun-based inorganic P solubilization genes of subsoil bacterial communities under camping and no camping. ns, *p* > 0.05.


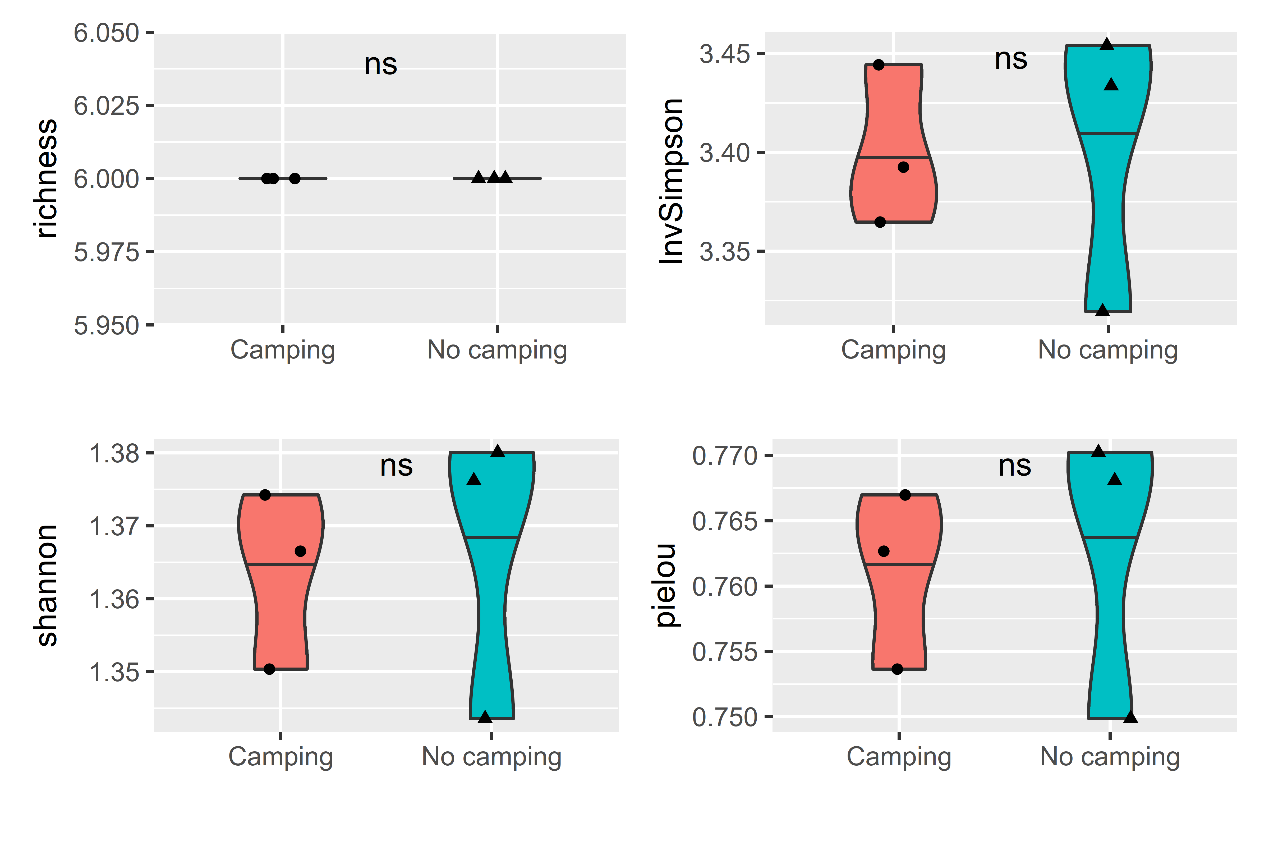


Figure S69. Diversities of Tax4Fun-based Organic P mineralization genes of subsoil bacterial communities under camping and no camping. ns, *p* > 0.05.


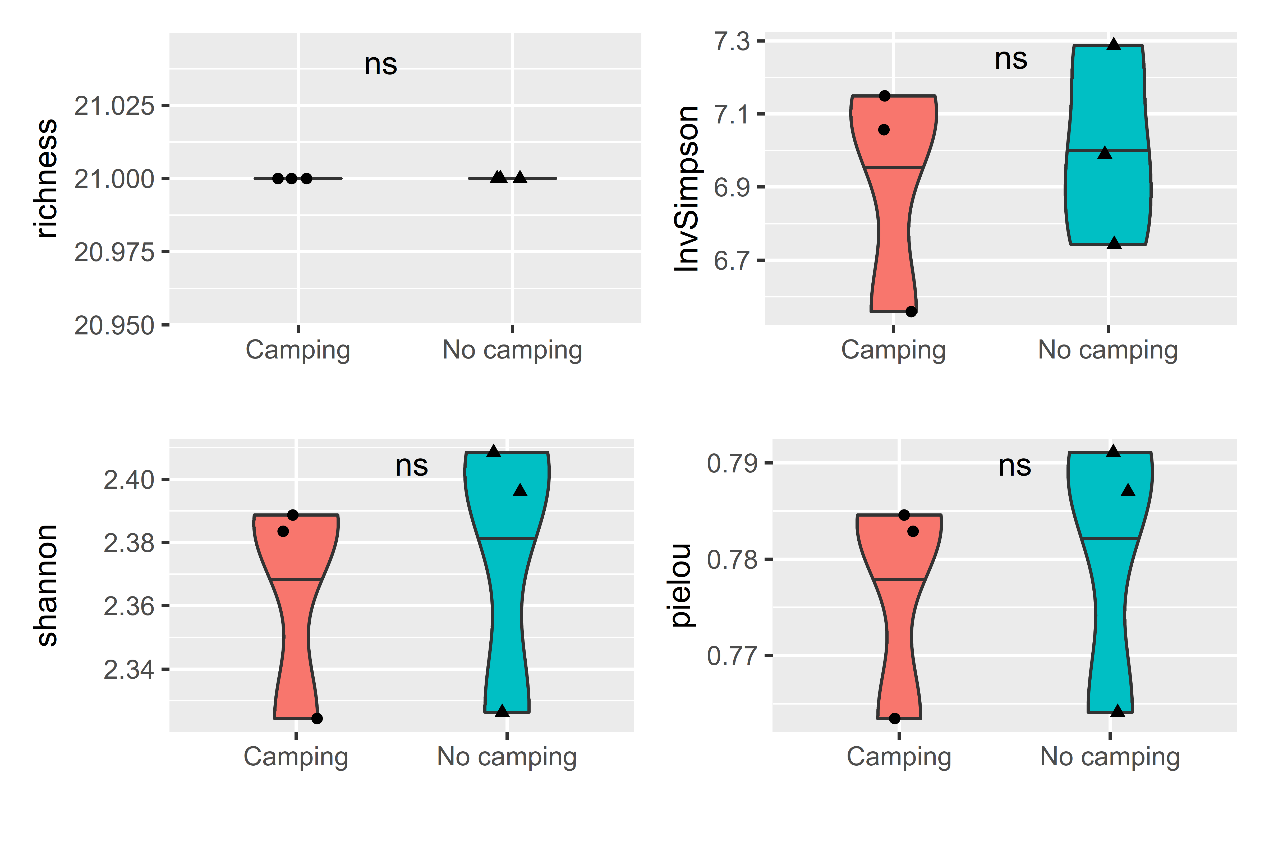


Figure S70. Diversities of Tax4Fun-based P uptake and transport genes of subsoil bacterial communities under camping and no camping. ns, *p* > 0.05.


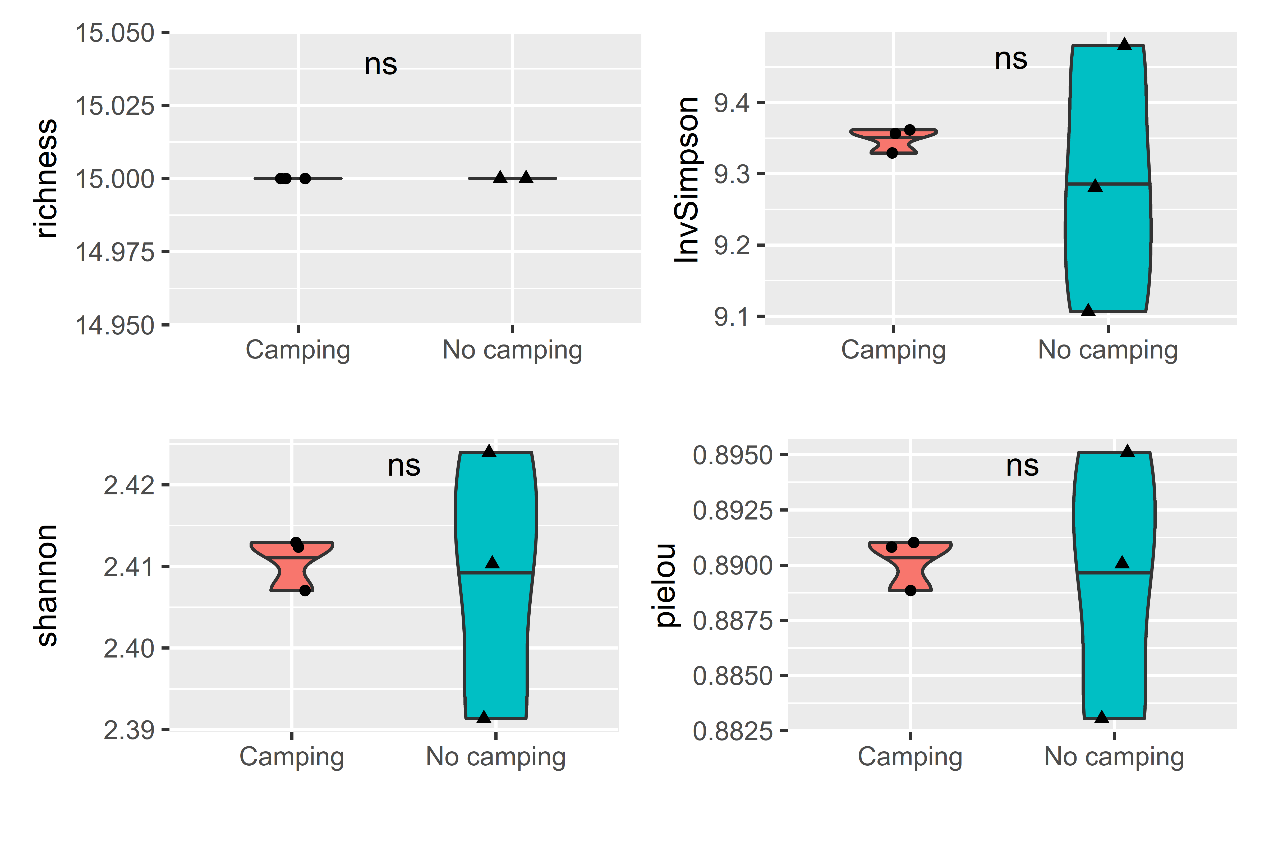


Figure S71. Diversities of Tax4Fun-based resistance gene types of subsoil bacterial communities under camping and no camping. ns, *p* > 0.05.


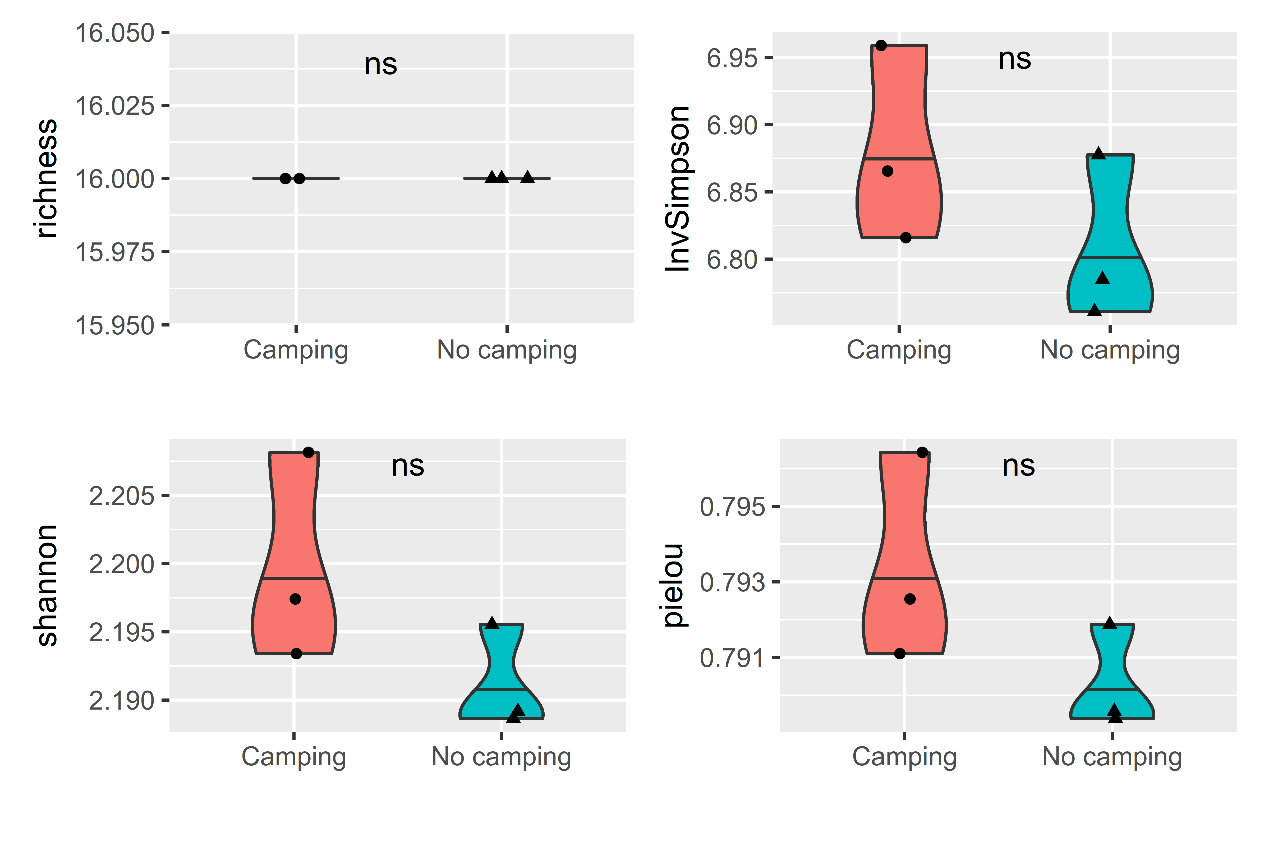


Figure S72. Diversities of Tax4Fun-based resistance gene subtypes of subsoil bacterial communities under camping and no camping. ns, *p* > 0.05.


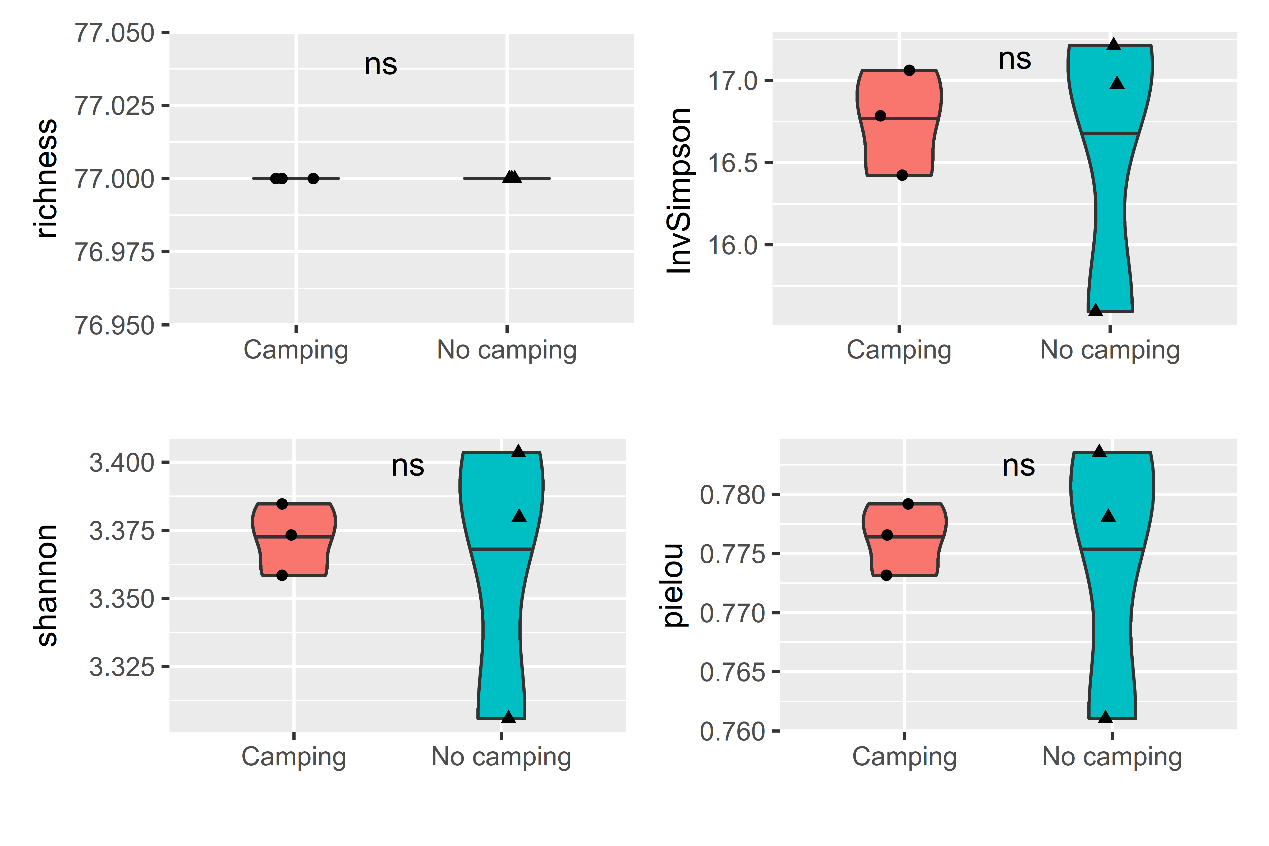


Figure S73. Diversities of Tax4Fun-based vancomycin resistance genes of subsoil bacterial communities under camping and no camping. ns, *p* > 0.05.


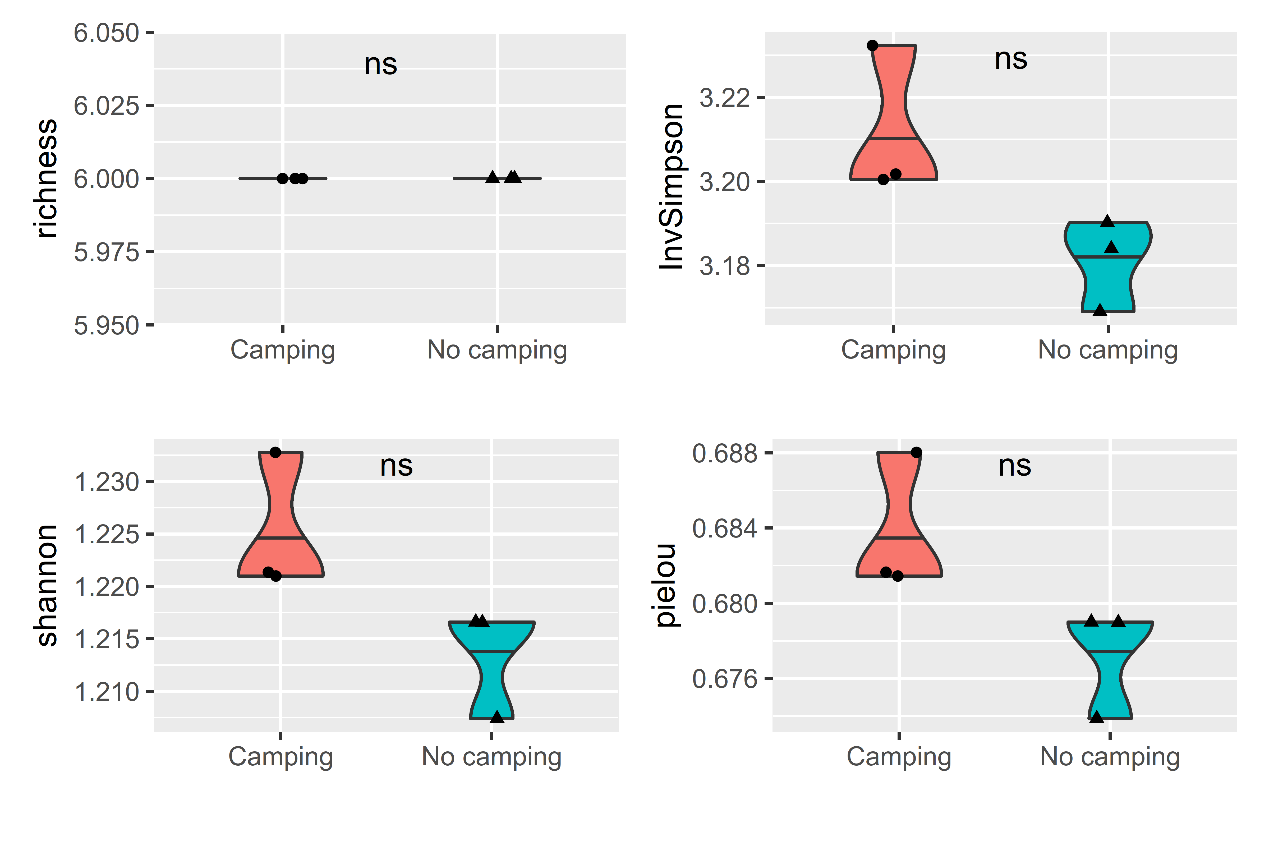


Figure S74. Diversities of Tax4Fun-based tetracycline resistance genes of subsoil bacterial communities under camping and no camping. ns, *p* > 0.05.


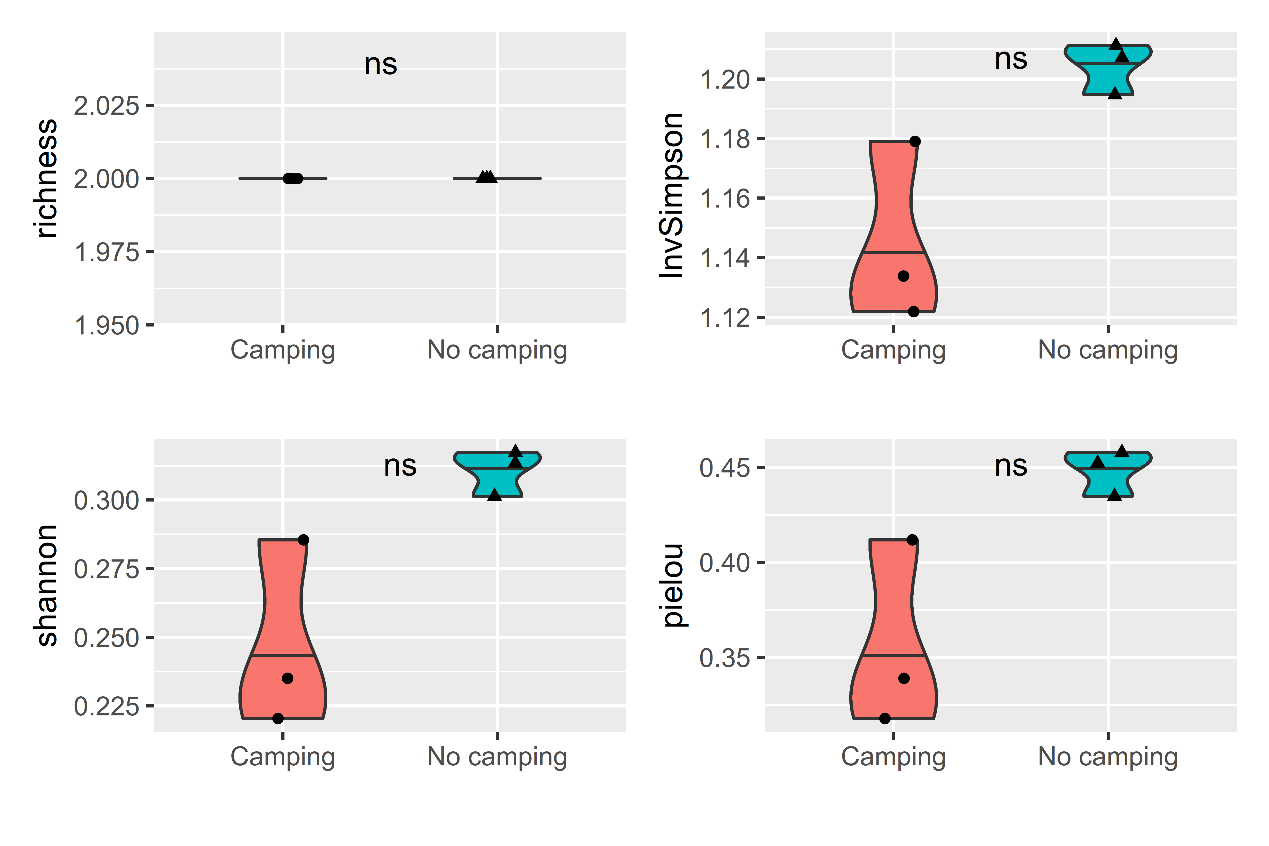


Figure S75. Diversities of Tax4Fun-based aminoglycoside resistance genes of subsoil bacterial communities under camping and no camping. ns, *p* > 0.05.


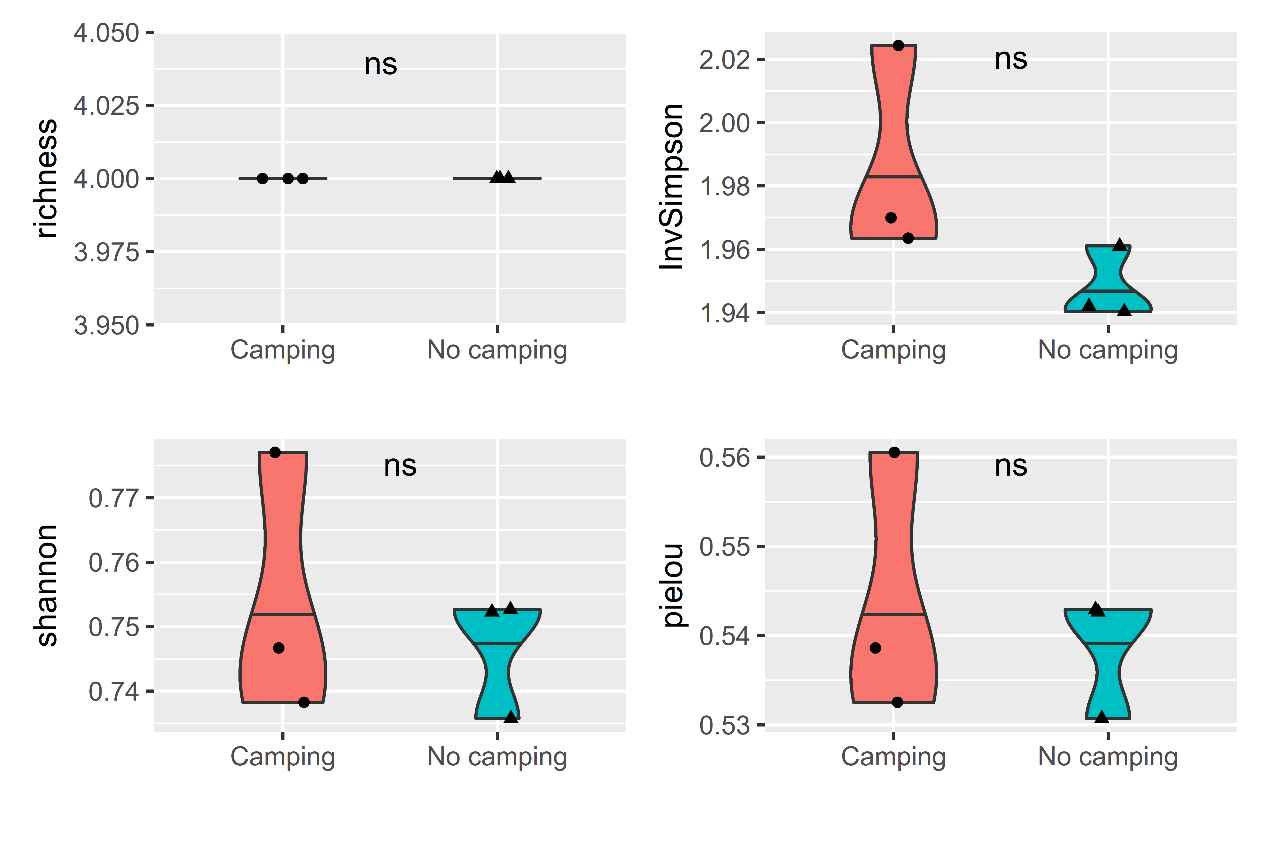


Figure S76. Diversities of Tax4Fun-based bacitracine resistance genes of subsoil bacterial communities under camping and no camping. ns, *p* > 0.05.


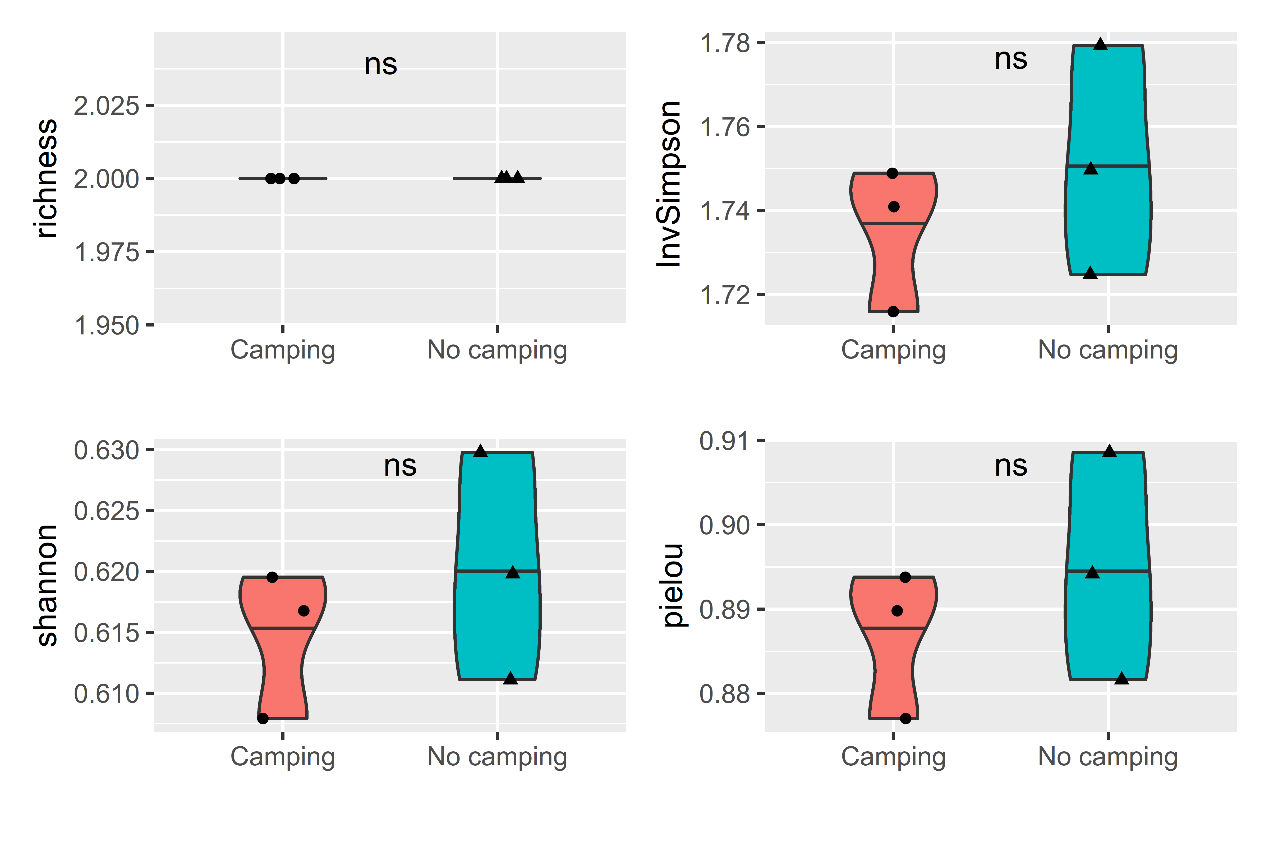


Figure S77. Diversities of Tax4Fun-based beta-lactam resistance genes of subsoil bacterial communities under camping and no camping. ns, *p* > 0.05.


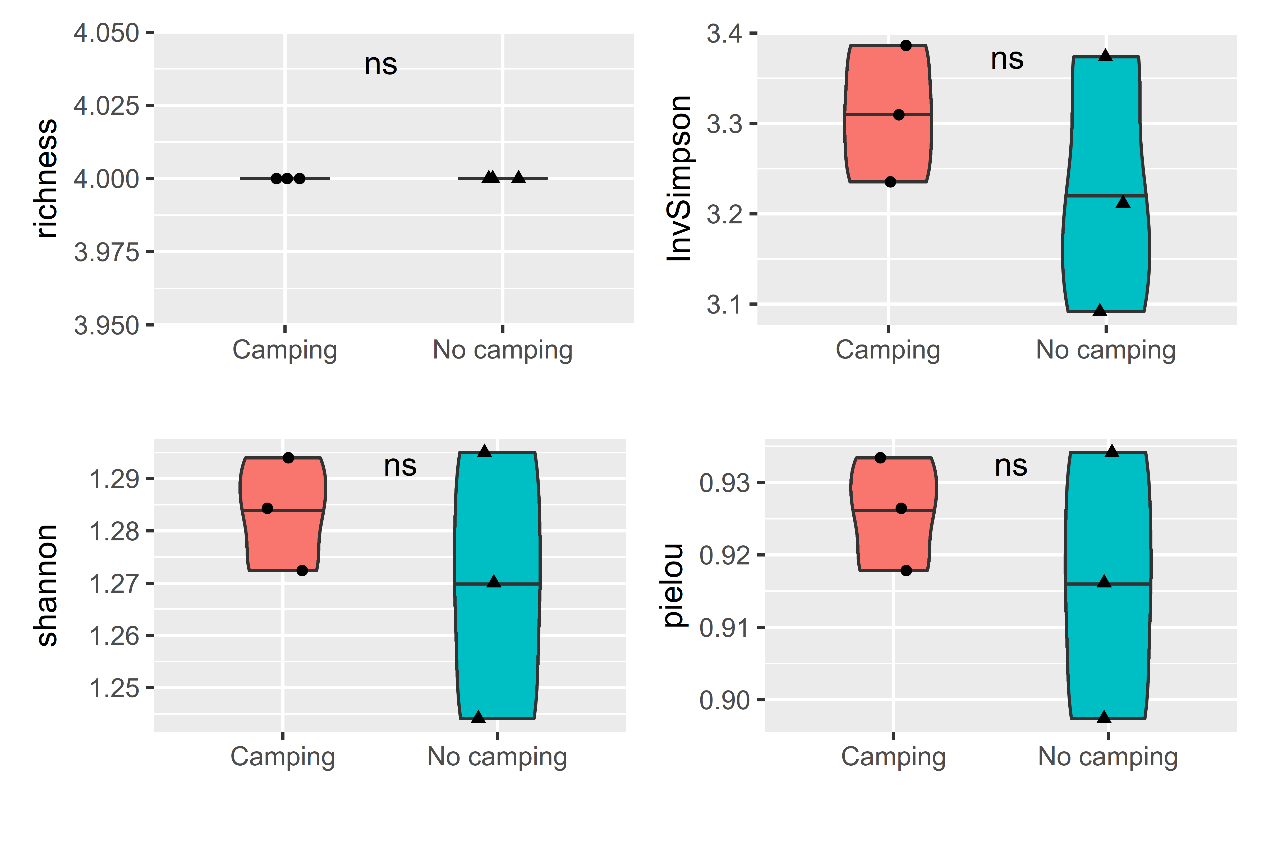


Figure S78. Diversities of Tax4Fun-based macrolide resistance genes of subsoil bacterial communities under camping and no camping. ns, *p* > 0.05.


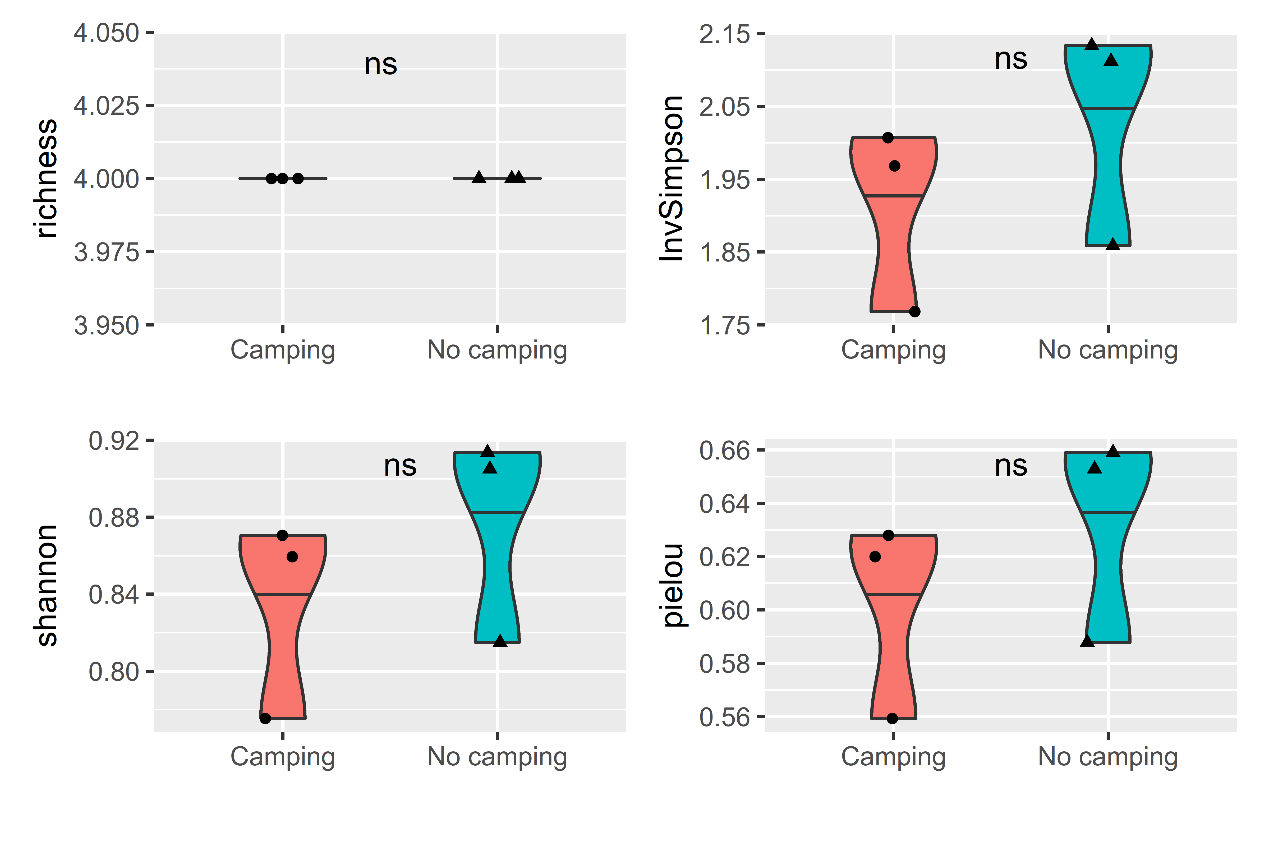


Figure S79. Diversities of Tax4Fun-based multidrug resistance genes of subsoil bacterial communities under camping and no camping. ns, *p* > 0.05.


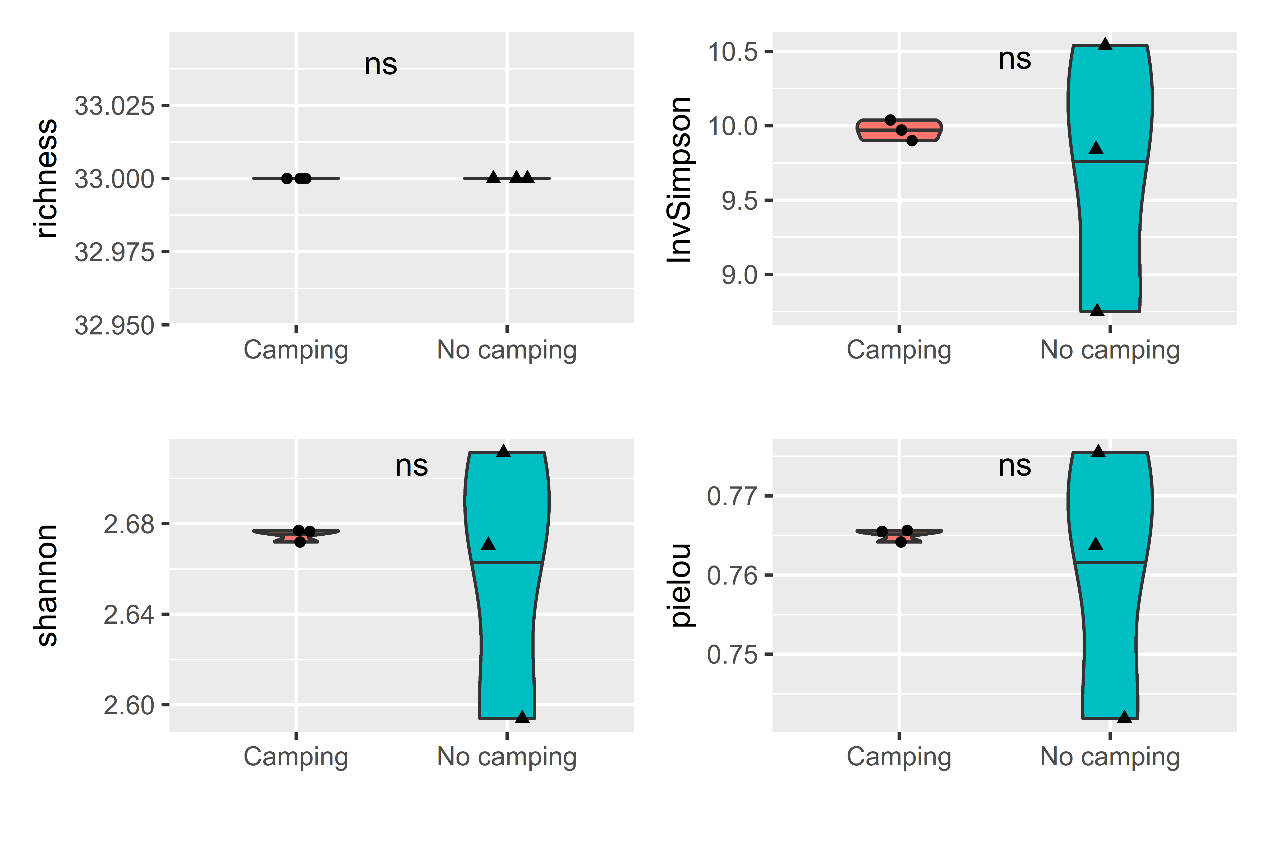


Figure S80. Diversities of Tax4Fun-based cationic antimicrobial peptide (CAMP) resistance genes of subsoil bacterial communities under camping and no camping. ns, *p* > 0.05.


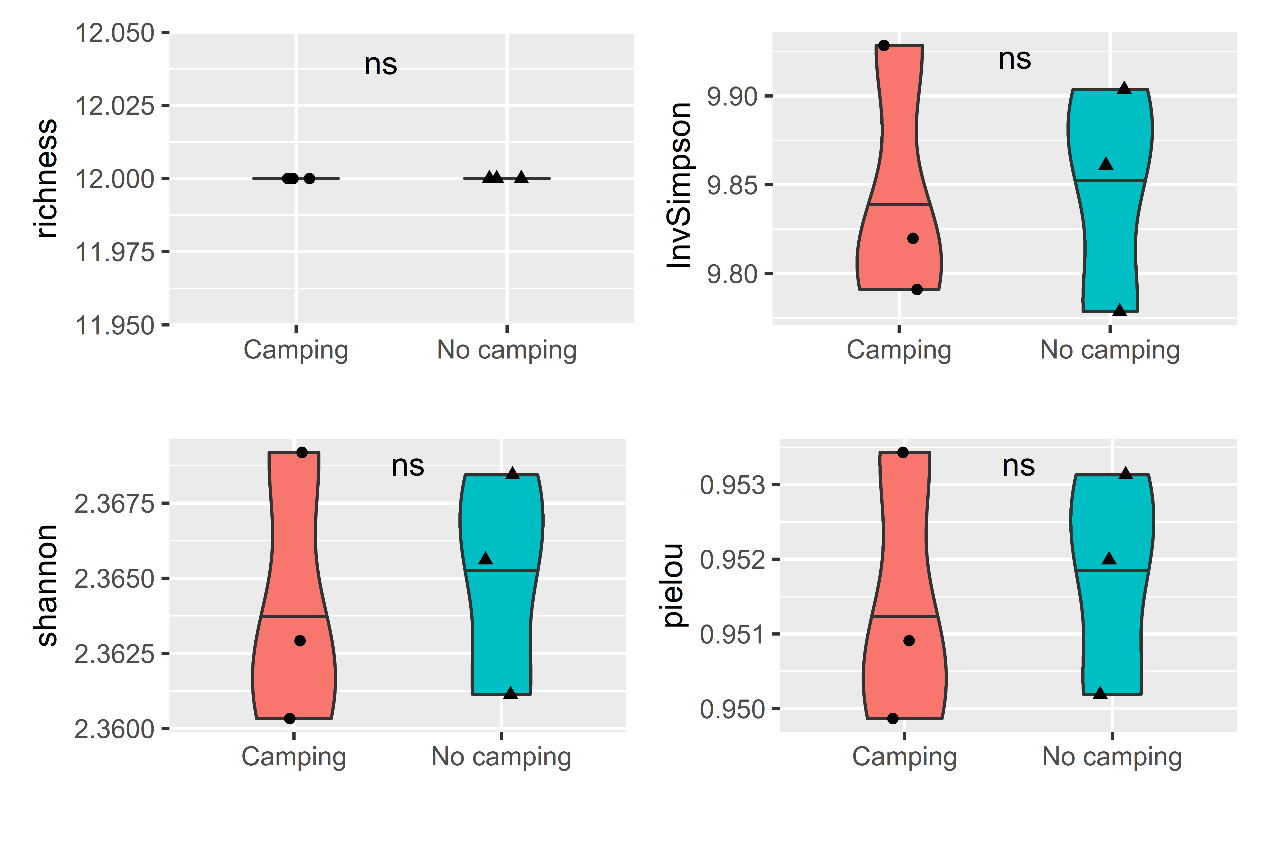


Figure S81. Diversities of Tax4Fun-based peptidases and inhibitors genes of subsoil bacterial communities under camping and no camping. ns, *p* > 0.05.


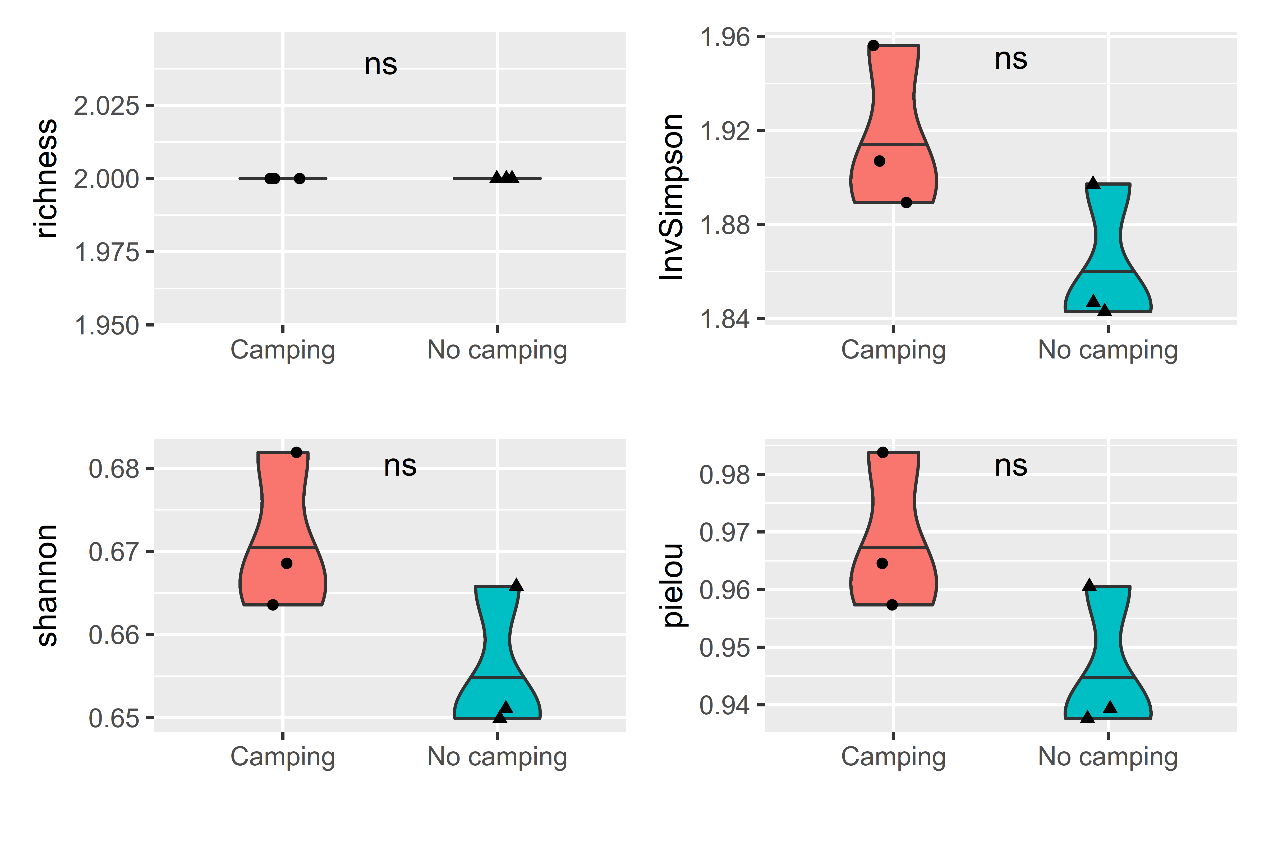


Figure S82. Diversities of Tax4Fun-based ABC transporters genes of subsoil bacterial communities under camping and no camping. ns, *p* > 0.05.


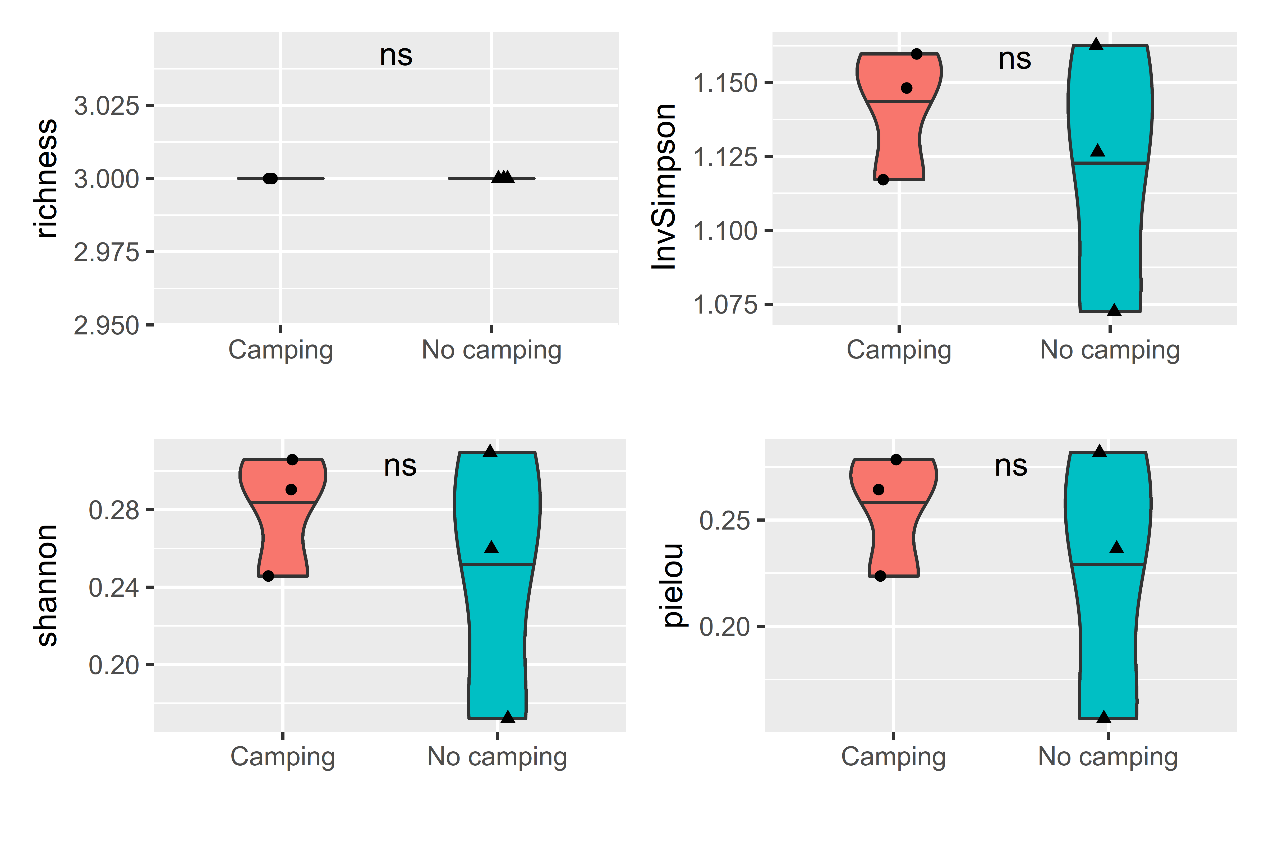


Figure S83. Diversities of FunGuild-based growth form of subsoil fungal communities under camping and no camping. ns, *p* > 0.05.


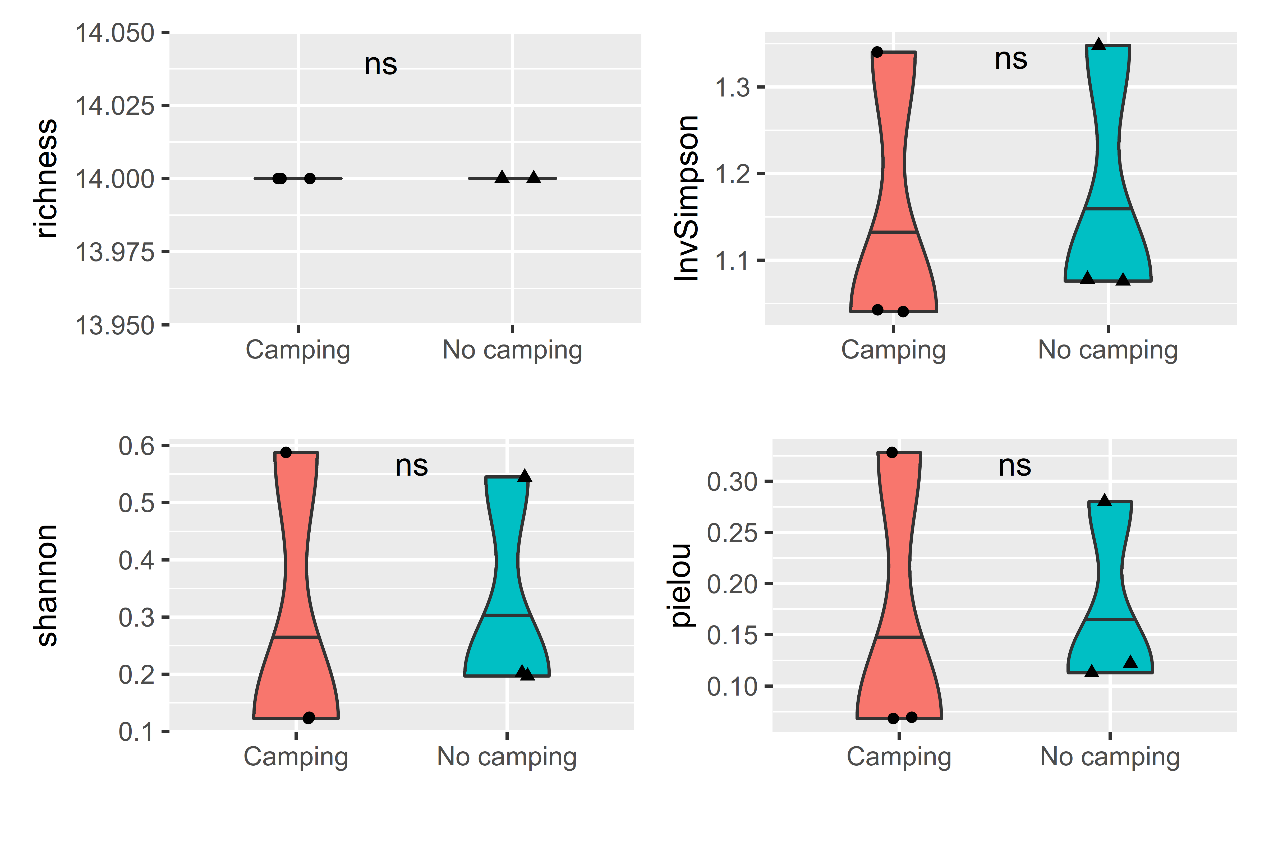


Figure S84. Diversities of FunGuild-based guild of subsoil fungal communities under camping and no camping. ns, *p* > 0.05.


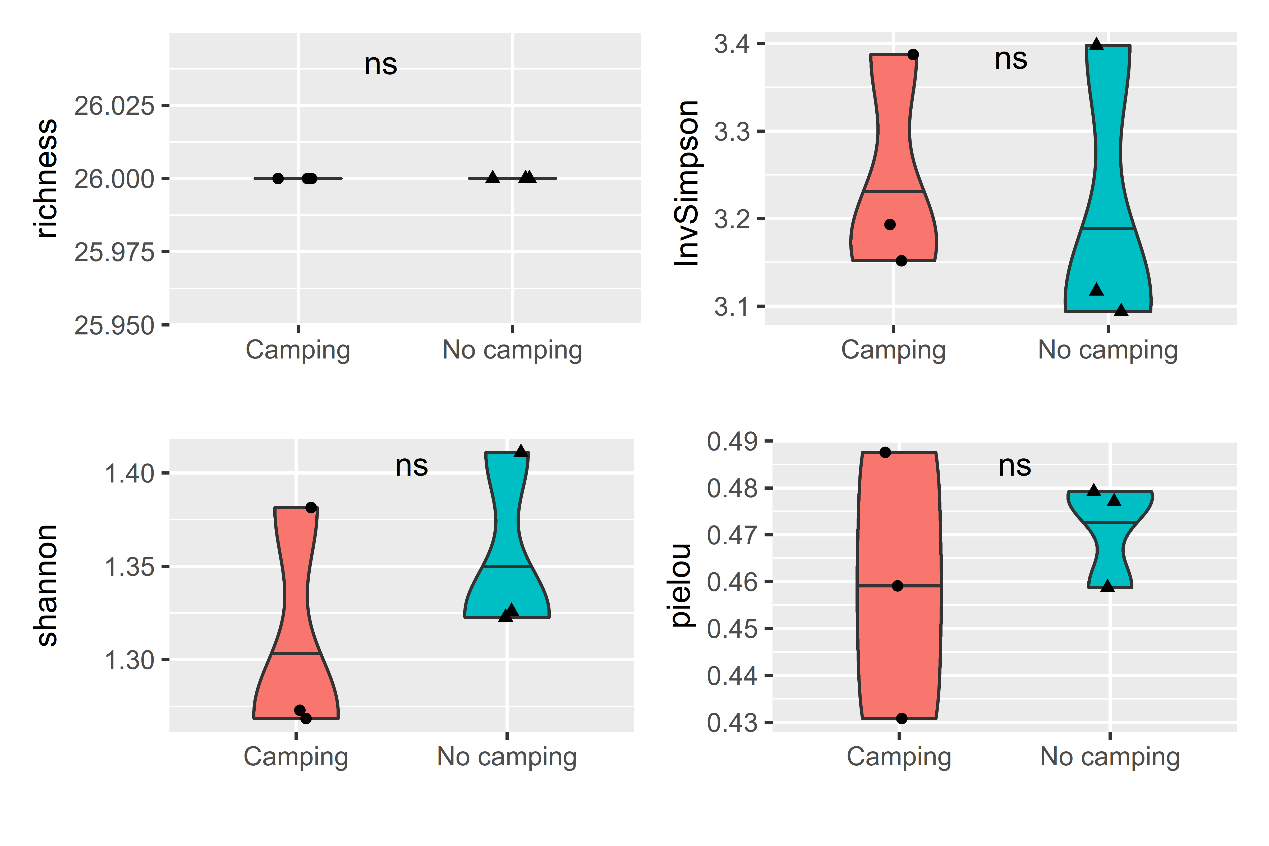


Figure S85. Diversities of FunGuild-based trait of subsoil fungal communities under camping and no camping. ns, *p* > 0.05.


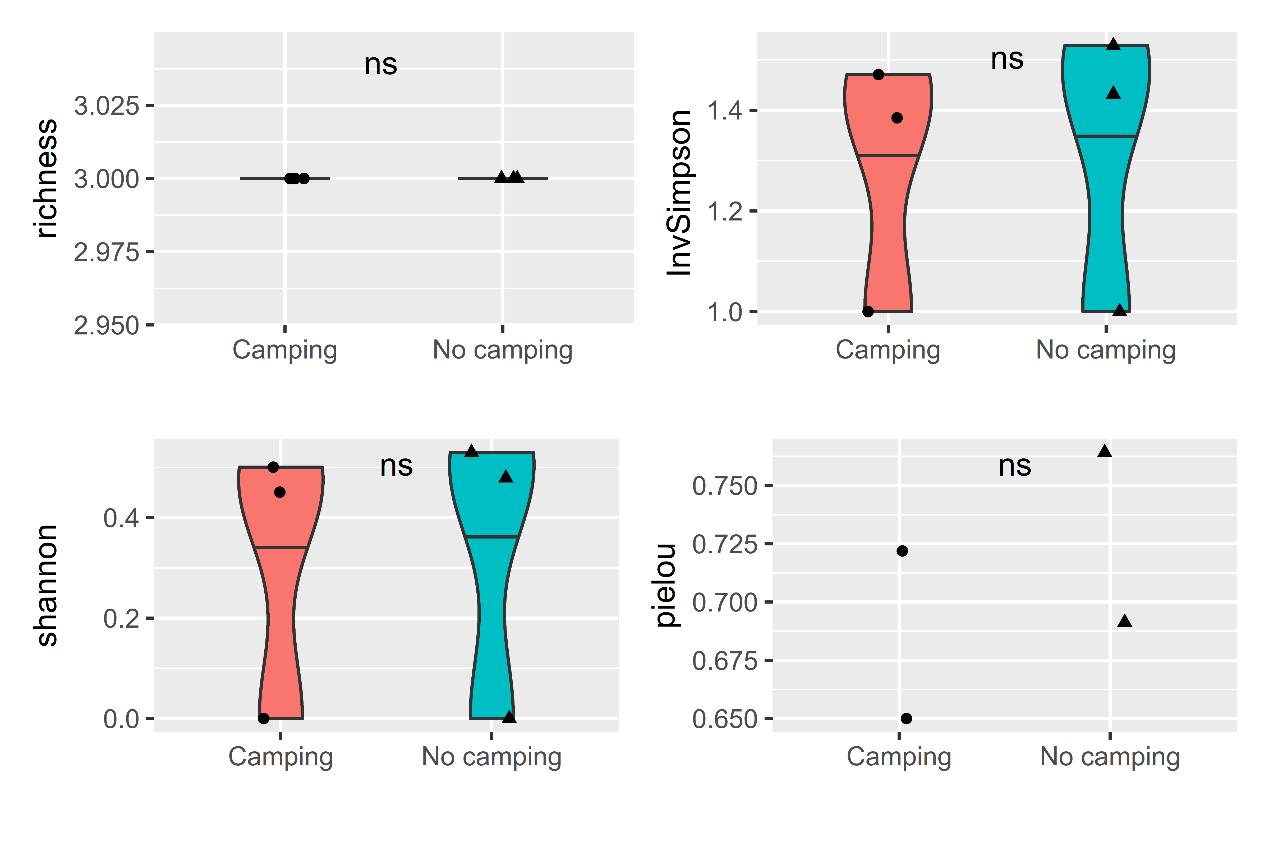


Figure S86. Diversities of FunGuild-based trophic mode of subsoil fungal communities under camping and no camping. ns, *p* > 0.05.


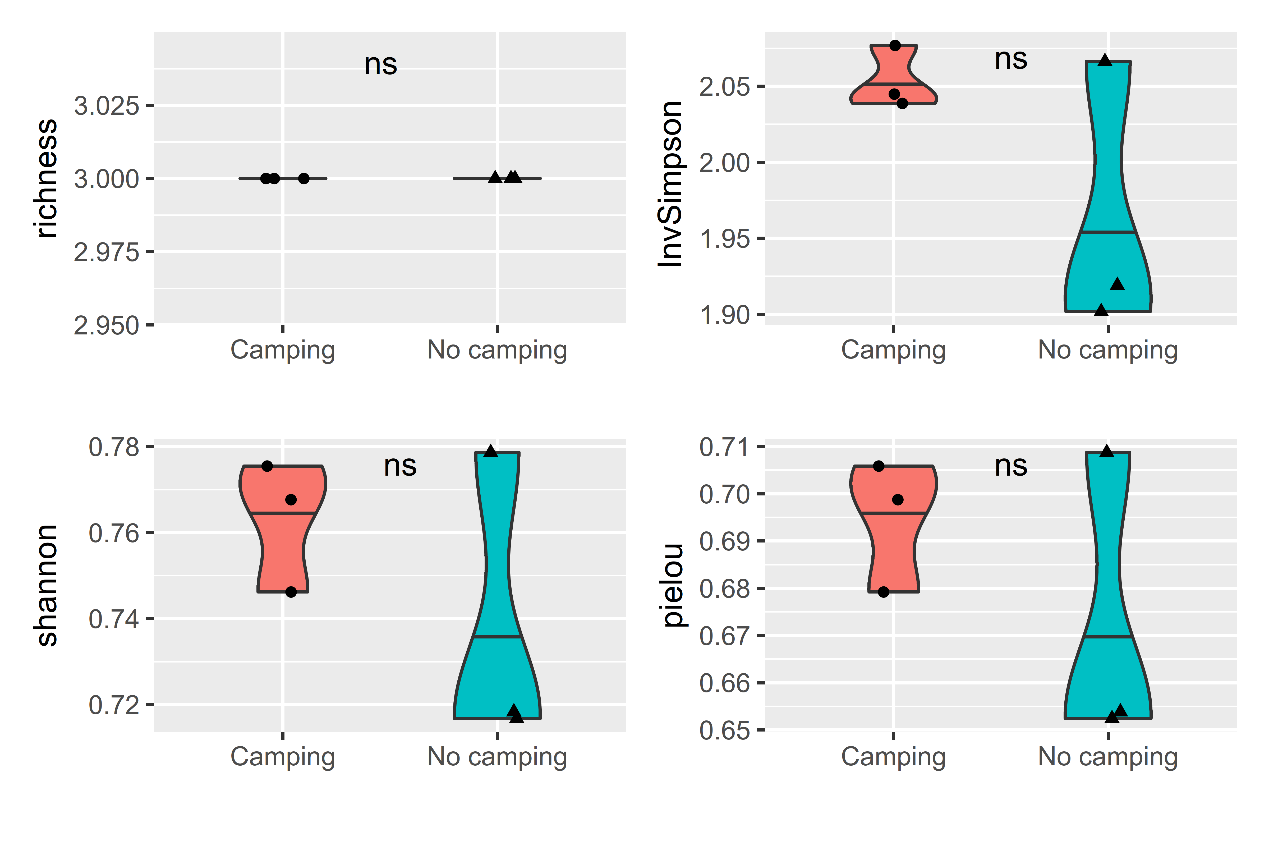

Supplement: Supplemental Information 1 [file peerj-10-14314-s001.zip › Figure S1-86.docx]
