# Supplementary material for: Herbivore camping reshapes the taxonomy, function and network of pasture soil microbial communities
Source: PeerJ. 2022 Nov 9;10:e14314. doi: 10.7717/peerj.14314 (PMC9653066; doi:10.7717/peerj.14314)
Supplement: Supplemental Information 1 [file peerj-10-14314-s001.zip › Legends of Figures S87-112.docx]

Figure S87. Differences in niche overlap，niche width, relative abundance of specialist/generalist species of high degree bacterial OTUs between groups. b, bacteria; hi.degree, high degree OTUs; camping.b.hi.degree, high degree bacterial OTUs under camping; no.camping.b.hi.degree, high degree bacterial OTUs under no camping; camping.specific.b.hi.degree, high degree bacterial OTUs only appeared under camping; no.camping.specific.b.hi.degree, high degree bacterial OTUs only appeared under no camping. The data in Figure 5a is analyzed here.

Figure S88. Differences in niche overlap，niche width, relative abundance of specialist/generalist species of low degree bacterial OTUs between groups. b, bacteria; lo.degree, low degree OTUs; camping.b.lo.degree, low degree bacterial OTUs under camping; no.camping.b.lo.degree, low degree bacterial OTUs under no camping; camping.specific.b.lo.degree, low degree bacterial OTUs only appeared under camping; no.camping.specific.b.lo.degree, low degree bacterial OTUs only appeared under no camping. The data in Figure 5b is analyzed here.

Figure S89. Differences in niche overlap，niche width, relative abundance of specialist/generalist species of 70 and 89 shared bacterial OTUs between groups. b, bacteria; camping.share.70.b.degree, 70 OTUs shared between high degree bacterial OTUs under no camping and low degree bacterial OTUs under camping. Camping.share.89.b.degree, 89 OTUs shared between high degree bacterial OTUs under camping and low degree bacterial OTUs under no camping. The data in Figure 5c, d is analyzed here.

Figure S90. Differences in niche overlap，niche width, relative abundance of specialist/generalist species of high degree fungal OTUs between groups. f, fungi; hi.degree, high degree OTUs; camping.f.hi.degree, high degree fungal OTUs under camping; no.camping.f.hi.degree, high degree fungal OTUs under no camping; camping.specific.f.hi.degree, high degree fungal OTUs only appeared under camping; no.camping.specific.f.hi.degree, high degree fungal OTUs only appeared under no camping. The data in Figure 5e is analyzed here.

Figure S91. Differences in niche overlap，niche width, relative abundance of specialist/generalist species of low degree fungal OTUs between groups. f, fungi; lo.degree, low degree OTUs; camping.f.lo.degree, low degree fungal OTUs under camping; no.camping.f.lo.degree, low degree fungal OTUs under no camping; camping.specific.f.lo.degree, low degree fungal OTUs only appeared under camping; no.camping.specific.f.lo.degree, low degree fungal OTUs only appeared under no camping. The data in Figure 5f is analyzed here.

Figure S92. Differences in niche overlap，niche width, relative abundance of specialist/generalist species of 8 and 9 shared fungal OTUs between groups. f, fungi; camping.share.8.f.degree, 8 OTUs shared between high degree fungal OTUs under no Camping and low degree fungal OTUs under camping; camping.share.9.f.degree, 9 OTUs shared between high degree fungal OTUs under camping and low degree fungal OTUs under no camping. The data in Figure 5g, h is analyzed here.

Figure S93. Faprotax-based functional differences between groups. b, bacteria; lo.degree, low degree OTUs; camping.b.lo.degree, low degree bacterial OTUs under camping; no.camping.b.lo.degree, low degree bacterial OTUs under no camping; camping.specific.b.lo.degree, low degree bacterial OTUs only appeared under camping; no.camping.specific.b.lo.degree, low degree bacterial OTUs only appeared under no camping;

hi.degree, high degree OTUs; camping.b.hi.degree, high degree bacterial OTUs under camping; no.camping.b.hi.degree, high degree bacterial OTUs under no camping; camping.specific.b.hi.degree, high degree bacterial OTUs only appeared under camping; no.camping.specific.b.hi.degree, high degree bacterial OTUs only appeared under no camping; camping.share.70.b.degree, 70 OTUs shared between high degree bacterial OTUs under no Camping and low degree bacterial OTUs under camping.

Figure S94. Tax4Fun-based carbon functional differences of low degree bacterial OTUs between groups. b, bacteria; lo.degree, low degree OTUs; camping.b.lo.degree, low degree bacterial OTUs under camping; no.camping.b.lo.degree, low degree bacterial OTUs under no camping; camping.specific.b.lo.degree, low degree bacterial OTUs only appeared under camping; no.camping.specific.b.lo.degree, low degree bacterial OTUs only appeared under no camping

Figure S95. Tax4Fun-based nitrogen (a-d) and phosphorus (e-h) functional differences of low degree bacterial OTUs between groups. b, bacteria; lo.degree, low degree OTUs; camping.b.lo.degree, low degree bacterial OTUs under camping; no.camping.b.lo.degree, low degree bacterial OTUs under no camping; camping.specific.b.lo.degree, low degree bacterial OTUs only appeared under camping; no.camping.specific.b.lo.degree, low degree bacterial OTUs only appeared under no camping.

Figure S96. Tax4Fun-based antibiotic resistance gene type (a, b) and subtype (c, d) differences of low degree bacterial OTUs between groups. b, bacteria; lo.degree, low degree OTUs; camping.b.lo.degree, low degree bacterial OTUs under camping; no.camping.b.lo.degree, low degree bacterial OTUs under no camping; camping.specific.b.lo.degree, low degree bacterial OTUs only appeared under camping; no.camping.specific.b.lo.degree, low degree bacterial OTUs only appeared under no camping.

Figure S97-98. Tax4Fun-based carbon functional differences of high degree bacterial OTUs between groups. b, bacteria; hi.degree, high degree OTUs; camping.b.hi.degree, high degree bacterial OTUs under camping; no.camping.b.hi.degree, high degree bacterial OTUs under no camping; camping.specific.b.hi.degree, high degree bacterial OTUs only appeared under camping; no.camping.specific.b.hi.degree, high degree bacterial OTUs only appeared under no camping

Figure S99. Tax4Fun-based nitrogen (a-d) and phosphorus (e-h) functional differences of high degree bacterial OTUs between groups. b, bacteria; hi.degree, high degree OTUs; camping.b.hi.degree, high degree bacterial OTUs under camping; no.camping.b.hi.degree, high degree bacterial OTUs under no camping; camping.specific.b.hi.degree, high degree bacterial OTUs only appeared under camping; no.camping.specific.b.hi.degree, high degree bacterial OTUs only appeared under no camping.

Figure S100. Differences in Tax4Fun-based antibiotic resistance gene type (a, b) and subtype (c, d) of high degree bacterial OTUs between groups. b, bacteria; hi.degree, high degree OTUs; camping.b.hi.degree, high degree bacterial OTUs under camping; no.camping.b.hi.degree, high degree bacterial OTUs under no camping; camping.specific.b.hi.degree, high degree bacterial OTUs only appeared under camping; no.camping.specific.b.hi.degree, high degree bacterial OTUs only appeared under no camping.

Figure S101. Differences in Tax4Fun-based carbon (a, b), nitrogen (c, d), phosphorus (e, f), and antibiotic resistance gene type (g) and subtype (h) of 70 shared bacterial OTUs between groups. b, bacteria; camping.share.70.b.degree, 70 OTUs shared between high degree bacterial OTUs under no camping and low degree bacterial OTUs under camping.

Figure S102. Differences in Tax4Fun-based carbon (a-d), phosphorus (e), and antibiotic resistance gene subtype (f) and type (g) of 89 shared bacterial OTUs between groups. b, bacteria; camping.share.89.b.degree, 89 OTUs shared between high degree bacterial OTUs under camping and low degree bacterial OTUs under no camping.

Figure S103-104. Tax4Fun-based carbon functional differences of low and high degree bacterial OTUs between groups. b, bacteria; lo.degree, low degree OTUs; camping.b.lo.degree, low degree bacterial OTUs under camping; hi.degree, high degree OTUs; camping.b.hi.degree, high degree bacterial OTUs under camping; camping.specific.b.lo.degree, low degree bacterial OTUs only appeared under camping; camping.specific.b.hi.degree, high degree bacterial OTUs only appeared under camping.

Figure S105. Tax4Fun-based nitrogen (a-d) and phosphorus (e-h) functional differences of low and high degree bacterial OTUs between groups. b, bacteria; lo.degree, low degree OTUs; camping.b.lo.degree, low degree bacterial OTUs under camping; hi.degree, high degree OTUs; camping.b.hi.degree, high degree bacterial OTUs under camping; camping.specific.b.lo.degree, low degree bacterial OTUs only appeared under camping; camping.specific.b.hi.degree, high degree bacterial OTUs only appeared under camping.

Figure S106. Differences in Tax4Fun-based antibiotic resistance gene type (a, b) and subtype (c, d) of low and high degree bacterial OTUs between groups. b, bacteria; lo.degree, low degree OTUs; camping.b.lo.degree, low degree bacterial OTUs under camping; hi.degree, high degree OTUs; camping.b.hi.degree, high degree bacterial OTUs under camping; camping.specific.b.lo.degree, low degree bacterial OTUs only appeared under camping; camping.specific.b.hi.degree, high degree bacterial OTUs only appeared under camping.

Figure S107-108. Tax4Fun-based carbon functional differences of low and high degree bacterial OTUs between groups. b, bacteria; lo.degree, low degree OTUs; no.camping.b.lo.degree, low degree bacterial OTUs under no camping; hi.degree, high degree OTUs; no.camping.b.hi.degree, high degree bacterial OTUs under no camping; no.camping.specific.b.lo.degree, low degree bacterial OTUs only appeared under no camping; no.camping.specific.b.hi.degree, high degree bacterial OTUs only appeared under no camping.

Figure S109. Tax4Fun-based nitrogen (a-d) and phosphorus (e-h) functional differences of low and high degree bacterial OTUs between groups. b, bacteria; lo.degree, low degree OTUs; no.camping.b.lo.degree, low degree bacterial OTUs under no camping; hi.degree, high degree OTUs; no.camping.b.hi.degree, high degree bacterial OTUs under no camping; no.camping.specific.b.lo.degree, low degree bacterial OTUs only appeared under no camping; no.camping.specific.b.hi.degree, high degree bacterial OTUs only appeared under no camping.

Figure S110. Differences in Tax4Fun-based antibiotic resistance gene type (a, c) and subtype (b, d) of low and high degree bacterial OTUs between groups. b, bacteria; lo.degree, low degree OTUs; no.camping.b.lo.degree, low degree bacterial OTUs under no camping; hi.degree, high degree OTUs; no.camping.b.hi.degree, high degree bacterial OTUs under no camping; no.camping.specific.b.lo.degree, low degree bacterial OTUs only appeared under no camping; no.camping.specific.b.hi.degree, high degree bacterial OTUs only appeared under no camping.

Figure S111-112. Differences in FunGuild-based fungal functions between groups. f, fungi; hi.degree, high degree OTUs; camping.f.hi.degree, high degree fungal OTUs under camping; no.camping.f.hi.degree, high degree fungal OTUs under no camping; camping.specific.f.hi.degree, high degree fungal OTUs only appeared under camping; no.camping.specific.f.hi.degree, high degree fungal OTUs only appeared under no camping.

lo.degree, low degree OTUs; camping.f.lo.degree, low degree fungal OTUs under camping; no.camping.f.lo.degree, low degree fungal OTUs under no camping; camping.specific.f.lo.degree, low degree fungal OTUs only appeared under camping; no.camping.specific.f.lo.degree, low degree fungal OTUs only appeared under no camping.

camping.share.9.f.degree, 9 OTUs shared between high degree fungal OTUs under camping and low degree fungal OTUs under no camping.
