# Supplementary material for: Herbivore camping reshapes the taxonomy, function and network of pasture soil microbial communities
Source: PeerJ. 2022 Nov 9;10:e14314. doi: 10.7717/peerj.14314 (PMC9653066; doi:10.7717/peerj.14314)
Supplement: Supplemental Information 1 [file peerj-10-14314-s001.zip › Table S5.docx]

**Table.S5 Summary of microbial functions described in the literatures**

| **Kingdom** | **Genus** | **Microbial functions** |
| --- | --- | --- |
| Bacteria | Aquamicrobium | Aquamicrobium genus is found in the activated sludge (Chang et al., 2013), sewage(Chang et al., 2015), soil (Wu et al., 2014b; Xu et al., 2017), and wastewater (Peter et al., 2009; Sun et al., 2020a). They are ammonia oxidizing bacteria (Su et al., 2021) and can largely contribute to the nitrogen removal (Sun et al., 2019). They are able to degrade organic pollutants(Xu et al., 2017), recalcitrant organic pollutants (Li et al., 2020b), and also degrade complex organic pollutants under micro-aerobic condition (Sun et al., 2020a). The genus can degrade heterocyclic compounds (Sun et al., 2019), aromatic compounds(Qu et al., 2015), thiophene-2-carboxylate (Peter et al., 2009), biphenyl and polychlorinated biphenyls, thiophene-2-carboxylate, petroleum, polychlorinated degrade biphenyls (PCB), biphenyl, cyhalofop-butyl (Wu et al., 2021), biphenyl and polychlorinated biphenyls(Zhang et al., 2015), polychlorinated Biphenyl (Chang et al., 2015), thiophene-2-carboxylate(Wu et al., 2014b), utilize 2,2’,4,4’,5,5’-hexachlorobiphenyl, 3,3’,4,4’-tetrachlorobiphenyl (Chang et al., 2021) for their growth, can metabolize biphenyl (Chang et al., 2021), and utilize 4-chlorobiphenyl, 2,3-dichlorobiphenyl, 2,3,4trichlorobiphenyl, 2,4,4′-trichlorobiphenyl, 2,3,4,6tetrachlorobiphenyl, 2,2′,4,4′,5-pentachlorobiphenyl, 2,2′,4,4′,5,5′-hexachlorobiphenyl (Chang et al., 2013). This indicates that members of this genus may repair environment (Wu et al., 2021). In addition, they can increase the biomass (Qu et al., 2015). |
| Bacteria | Cellvibrio | Most Cellvibrio species are saprophytic soil bacteria known for their ability to degrade plant cell wall polysaccharides (Yannick and David, 2013; Zhang et al., 2020b). Cellvibrio genus mainly drivers cellulose and hemicellulose fibers hydrolysis in sludge (Li et al., 2019a), exhibits a much wider distribution of lignocellulose degradative ability, can secrete 3-4 types of cellulose-degrading enzymes, a wide variety of polysaccharide hydrolases and laminarinase (Sakellaris et al., 1993), possess xylanase genes (Borjigin et al., 2021), secretes hydrolytic enzyme，such as cellulose hydrolases (Sun et al., 2021), glycoside hydrolases (Zhang et al., 2020b), produces an array of endohydrolytic enzymes involved in the initial phases of β-glycan polysaccharide degradation in the soil (Sakellaris et al., 1997). Nine enzymes were specific to Cellvibrio and were involved in the degradation of glucans (EC:3.2.1.91; EC:3.2.1.39; EC:3.2.1.4; EC:3.2.1.21), and degradation of pectin and further conversion of its metabolites (EC:3.1.1.11; EC:4.2.2.2; EC:4.2.1.7; EC:5.3.1.12; EC:1.1.1.57). In addition to those, genes encoding for mannan (EC:3.2.1.78), arabinan (EC:3.2.1.55) and chitin (EC:3.2.1.14; EC:3.2.1.52), modifying enzymes from Cellvibrio, cell wall polysaccharide degradation (Maya et al., 2014). This genus degrades carboxymethyl cellulose, cellulose, hemicellulose, and chitin (Borjigin et al., 2022), polysaccharides (Akyol et al., 2019), starch, xylan, agar.(Xie et al., 2015), polysaccharides (Zhang et al., 2017c), lignocellulose wastes (Sun et al., 2021) and cellulose (Kolton et al., 2011; Tian et al., 2013), carboxymethyl cellulose, cellulose, hemicellulose and chitin (Borjigin et al., 2021), polysaccharides such as cellulose, xylan, xyloglucan, mannan, arabinan and pectin (Chen et al., 2018), dextran (Wu et al., 2014a) or amylose (Romanenko et al., 2010), manure compost (Gou et al., 2021), xylose, arabinose, and galactose(Xie et al., 2017), yeast cell walls, soluble 0-1,3 glucan laminarin, insoluble 0-1,3 glucans zymosan and pachyman (Sakellaris et al., 1990), ethyl hydroxyethyl cellulose (Björn et al., 1968), polysaccharides (Yannick and David, 2013; Zhang et al., 2020b), chitin (De tender et al., 2019), chitosan (Kazuaki et al., 2010), hemicellulose, cellulose, dextran, xylan, chitin and starch (Wu and He, 2015)，a variety of β-linked polysaccharides such as chitin, cellulose, β-1,3 glucan, and mixed-linkage β-1,3-1,4 glucans (Sakellaris et al., 1997). Cellvibrio genus is involved in the N-cycle (e.g., (De tender et al., 2019)，has nitrate-reducing activity (Nakajima-Kambe et al., 2005). Cellvibrio is also common to plant inhabitants with nitrogen fixation activity (Xiao et al., 2019) that promote the increase of biomass (Akyol et al., 2019; Cristóbal et al., 2022). Cellvibrio genus carries some antibiotic resistance genes (Gou et al., 2021), such as tetracycline resistance gene (Zhang et al., 2017c). Cellvibrio has been widely found in harsh environments, and shows a strong tolerance to various environmental stresses (Zhong et al., 2020a).  Plant matter is a source of cellulose and β-1,3-1,4 glucan (Sakellaris et al., 1997). |
| Bacteria | Chelatococcus | Chelatococcus employs desulfurization through specific cleavage of carbon-sulfur (C-S) bonds (Bordoloi et al., 2016)，degrades PAHs (Avani et al., 2021)， nitrilotriacetate (Margarete and Thomas, 2001), nitrilotriacetic acid (Wilberg et al., 1993), octylphenol polyethoxylates (Yudai et al., 2012)，low-molecular weight polyethylene (Gannes et al., 2013), acesulfame (Huang et al., 2021b)，aminopolycarboxylic acids, poly(3-hydroxybutyrate) (Liu et al., 2013) and crude-oil (Wang et al., 2016)，and produce polyhydroxyalkanoates (Iva et al., 2020) and poly(3-hydroxybutyrate) (Xu et al., 2014).  This genus has the nirK gene(Liu et al., 2021d), is aerobic denitrifier (Liang et al., 2012; Liang and Huang, 2015), employs denitrification for N removal (Yang et al., 2014; Han et al., 2016)，can be as biosorbent for metal removal (Chintalpudi et al., 2021), utilizes the metal-chelating nitrilotriacetate as a sole source of carbon, energy, and nitrogen (Singh, 2011; Thieringer et al., 2021). In addition, Chelatococcus has important role in delignification process(Parmeshwar et al., 2021 ). |
| Bacteria | Christensenellaceae R-7 group | Christensenellaceae R-7 group is ruminal bacteria(Pan et al., 2017; Yan et al., 2018; Bach et al., 2019; Li et al., 2019b; Ma et al., 2020c; Qiu et al., 2020; Yang et al., 2020; Couch et al., 2021; Gui et al., 2021; Huang et al., 2021a; Liu et al., 2021c; Xiang et al., 2021; Zhu et al., 2021; Qiu et al., 2022) and fermentative bacteria (Gao et al., 2022), can degrade complex organics, leaving VFAs (e.g., acetic and butyric acids) (Gao et al., 2022), strongly degrades carbohydrates, amino acids, and carboxylic acids, leaving acetic and butyric acids(Chen et al., 2020), performs propionate and butyrate methanogenic degradation (Cai et al., 2022), and organic matter degradation (Hou et al., 2021). |
| Bacteria | Clostridium sensu stricto 18 | Clostridium sensu stricto 18 is hydrocarbon degrading (Diana et al., 2020) and hydrogen-producing genera (Yang and Wang, 2021). |
| Bacteria | Cohnella | Cohnella can degrade xylan (Yoon et al., 2007; Hatsumi et al., 2010; Luo et al., 2010; Saowapar et al., 2010a; Saowapar et al., 2010b; Fathallh Eida et al., 2012), cellulose (Saowapar et al., 2012; Adriana et al., 2015; Guadalupe et al., 2019; Li et al., 2020c; Yang et al., 2021b), carboxymethyl cellulose, sawdust (Fathallh Eida et al., 2012), and litter (Xu et al., 2019), can use chitin as the sole carbon source (Nasrin et al., 2016) and fix nitrogen (Wang et al., 2012; Wang et al., 2015a; Marie et al., 2017; Xiao et al., 2019). |
| Bacteria | Dyadobacter | Dyadobacter can utilize algal-produced isoprenes (Kimbrel et al., 2019), degrade xenobiotic compounds (Reid et al., 2020), methyl red (Wang et al., 2015b), biodegrade xenobiotics (Pia et al., 2005), and utilise organic residue, cellulolytic (Yuwarad and Savitri, 2019). Dyadobacter is essential in partial nitrification (Zhang et al., 2019b), carries nifH gene, increases soil Nitrate N, ammonical N, and plant growth (Saurabh et al., 2018). Dyadobacter may also be involved in disease suppression (Fu et al., 2017). |
| Bacteria | Family XIII AD3011 group | Family XIII AD3011 group is rumen microbiota (Yu et al., 2020; Park et al., 2021). |
| Bacteria | FukuN18 freshwater group | This genus is found in pasture soils exposed to urea (urine patches) (Ganasamurthy et al., 2021). Higher abundance of this genus in river ecosystems indicate the degradation status of river ecosystems (Yang et al., 2019b). |
| Bacteria | LD29 | LD29 can utilize carbon sources (cellulose, mannan, xylan, chitin, starch), sulfated polysaccharides (Madhusmita et al., 2020). This genus can be found in gut (Liao et al., 2021), and the abundance of LD29 was higher in high oxygen environments than in low oxygen environments (Zhao et al., 2021b). |
| Bacteria | Limnothrix | This genus is non-heterocystous filamentous cyanobacteria (Gaget et al., 2017), and can metabolise a wide range of organic substrates such as amino acids, carbohydrates, and carboxylic acids(Dimitra et al., 2021). |
| Bacteria | Luteimonas | Luteimonas is found in sewage sludges (Lu et al., 2019), crude oil (Onotasamiderhi et al., 2019), waste compost (Li et al., 2020a; Ma et al., 2020a; Wang et al., 2021c), manure (Wang et al., 2015c), chicken litter (Liu et al., 2020b), and rumen (Xiang et al., 2021), has a key role during the composting of humification (Germán et al., 2021), anaerobic digested residue (Wang et al., 2020), manure (Liu et al., 2020a; Zhong et al., 2020b), and the wood (Wang et al., 2021b). This genus can degrade various carbohydrates (Wang et al., 2021b) such as starch(Lee et al., 2010), cellulose (Lin et al., 2020), chitin (Zhou et al., 2021a), lignocellulose (Ma et al., 2020a), aromatic hydrocarbon (Onotasamiderhi et al., 2019), and are capable of mineralizing high molecular weight polycyclic aromatic hydrocarbons such as benzo[a]pyrene and pyrene (Hernando and Chihiro, 2015; Lu et al., 2019; Nagalakshmi et al., 2019; Onotasamiderhi et al., 2019), has catalytic activities related to oxidase, catalase, alkaline phosphatase, esterase, and esterase lipase, and take part in the organic matter biodegradation metabolisms (Guo et al., 2017; Mekdimu et al., 2021). It also degrades bisphenol A(Agnieszka and Magdalena, 2018)， phenanthrene (Elyamine and Hu, 2020)， petroleum hydrocarbon (Eman et al., 2020) and antibiotic sulfamethoxazole (Zheng et al., 2021).  Luteimonas is aerobic and nitrate reducing bacteria (Mekdimu et al., 2021), has ability in denitrifying (David et al., 2018) by reducing nitrite only to nitrous oxide (N_2_O) (Zhang et al., 2017d).  Luteimonas carries some antibiotic resistance genes (Gou et al., 2021). The dissipation of Luteimonas in the composts contributed greatly to the reduction in relative abundance of antibiotic resistance gene (Liu et al., 2020a; Wang et al., 2021c).  Luteimonas shows a slight inhibition of pathogen growth， as part of healthy microbiome of healthy plants (Kristina et al., 2020). They promotes early plant growth and development, increased the acquisition of nitrogen (N) and phosphorous (P) by plants (Claire, 2019). Luteimonas in soils may be an useful indicator for soil amelioration (Guo et al., 2017). |
| Bacteria | OLB13 | The OLB13 genus harbors genes that encode key enzymes required for respiratory ammonification (Cao et al., 2022), that encoding for key enzymes required for respiratory ammonification, N_2_O-detoxification and CO_2_ fixation pathways (Xu et al., 2021), is involved in the nitrification or denitrification process of activated sludge (Han et al., 2020), plays key roles in nitrite accumulation (Zhang et al., 2021a), has anaerobic fermentation function (Zhou et al., 2021b), and degrades sulfamethoxazole (Song et al., 2020). |
| Bacteria | Olivibacter | Olivibacter can be found in soils, waste (Rubén et al., 2020) and gut (Jing et al., 2020), degrade hydrocarbon (István et al., 2011; Rubén et al., 2020) and polymer (Zhang et al., 2021b), complex and toxic compounds (Tian et al., 2017), is also essential in partial nitrification (Zhang et al., 2019b), enable fixing nitrogen to promote plant growth (He et al., 2022). |
| Bacteria | Prevotellaceae UCG-004 | This genus can be found in rumen (Bach et al., 2019; Li et al., 2019b; Qiu et al., 2020; Ribeiro et al., 2020; Christel et al., 2021; Liu et al., 2021c; Zhu et al., 2021; Qiu et al., 2022) and feces of ruminant (Zhang et al., 2019a; Filippo et al., 2020; Jiang et al., 2022). Prevotellaceae UCG-004 has been positively correlated with carbohydrate metabolism (Gaukroger et al., 2021), utilize starch, protein, peptides, hemicellulose, and pectin, play an important role in protein metabolism(Zhu et al., 2021). |
| Bacteria | Ruminococcaceae UCG-005 | Ruminococcaceae UCG-005 can be found in rumen (Bach et al., 2019; Li et al., 2019b; Ren et al., 2020; Yang et al., 2020; Zhu et al., 2021; Zheng et al., 2022), intestinal microbiome (Couch et al., 2021; Domínguez et al., 2022), feces of ruminant (Ezequias et al., 2020; Hao et al., 2020; Ma et al., 2020b; Dai et al., 2021; Khatoon et al., 2022), and cattle houses (Zhang et al., 2022b). It can produce butyrate (Leonardo et al., 2017), digest fiber, and produce short chain fatty acids by fermenting dietary polysaccharides (Chen et al., 2021). |
| Bacteria | Ruminococcus 1 | This genus is rumen bacteria (Henderson et al., 2019; Zhang et al., 2019c; Qiu et al., 2020; Yang et al., 2020; Zheng et al., 2022), as the chief producers of CAZymes in cattle rumen ecosystem (Jose et al., 2017), and produce several types of and most of cellulases and hemicellulases in rumen (Rabee et al., 2022), produce large amounts of cellulolytic enzymes, including exoglucanases, endoglucanases, glucosidases, hemicellulases (Zhang et al., 2017a). It degrades plant polysaccharides, hemicellulose, pectin, and cellulose present in the plant cell wall, fiber (Kohl et al., 2018), xylan and pectin and utilize degraded soluble sugars as substrates (Zhang et al., 2017a), and it can degrade complex carbohydrates and produce short chain fatty acids (Chen et al., 2021), break down fibrous plant material to generate acetate, formate, succinate, and other short chain fatty acids, degrade synergistic cellulose and hemicellulose (Lu et al., 2021), is also associated with thiamine synthesis (Chuang et al., 2021).  It is cellulose-decomposing bacteria (Zhang et al., 2017b; Ma et al., 2021; Song et al., 2021), lactate utilizing bacteria (Xue et al., 2018), and thiamine-synthesis related bacteria (Xue et al., 2019), can ferment complex nondigestible polysaccharides (Zhang et al., 2022a). It is efficient in the breakdown of cellulose and hemicellulose (Iqbal et al., 2018), is the most important cellulose-degrading bacteria in the intestine and rumen of herbivores (Zhang et al., 2017a). Its increases enhance the fiber degradation and ruminal acetate production (Pan et al., 2017; Pan et al., 2018). |
| Bacteria | Pseudanabaena PCC-7429 | This genus is small filamentous cyanobacterium, which is often an epiphyte of Microcystis colonies (Christopher and Jennifer, 2022) and can be found in microbial mats (Nataliia et al., 2020). |
| Bacteria | RB41 | RB41 is involved in soil C (Zachary, 2020) and N cycling (Xi Zhang et al., 2022), accounts for the majority of soil C flux(Bram et al., 2021), play key role in nitrification and N assimilation (Huang et al., 2021c; Meier et al., 2021), has strong adaptability of RB41 to low nutrition (Alexandria and Rachel, 2019; Siddarthan et al., 2021; Zhao et al., 2021a), play an important role in maintaining the metabolic and biogeochemical function (Lin et al., 2022) of soil under long-term low-nutrient stress conditions(Sun et al., 2020b), substantial potential and application value in environmental pollution remediation (Li et al., 2022a), enhance the biodegradation of polyfluoroalkyl substances (Senevirathna et al., 2022). The relative abundance of genera RB41 has negatively correlated trend with soil pH and positively correlated trend with soil NO_3_^−^-N, AP, TN, and TP (Zhao et al., 2020b). RB41 is commonly found in rhizosphere (Huang et al., 2021c), and petroleum contaminated soil (Alexandria and Rachel, 2019). RB41 is the most abundant genera in the healthy soil (Jiao et al., 2022). |
| Bacteria | Rudaea | Rudaea plays key players in nitrification and N assimilation (Meier et al., 2021), can decompose plant residues, degrade organic matter and conversion of solid organic waste into useable nutrients for plants (Nasser et al., 2020). It is involved in denitrification or the biodegradation of some aromatic compounds, cellulose (Nasser et al., 2020; Zhang et al., 2020a), biphenyl, naphthalene and phenol (Qu et al., 2016; Tang et al., 2017) and antibiotics (Zhao et al., 2020a), is resistant to multiple antibiotics(Zhao et al., 2019) and produce antifungal metabolites (Nasser et al., 2020). Rudaea is a pathogenic species(Li et al., 2021), can be found in the polymer synthesis activated sludge (Federico et al., 2013). |
| Bacteria | Rummeliibacillus | Rummeliibacillus is facultative anaerobic (Mohapatra and Duc, 2013), is found in organic farming soils (Chou et al., 2017), and composting processes (Robledo et al., 2020). It carries carbohydrate-utilizing genes, genes involved in the breakdown of protein, involved in cellulose metabolism, including endoglucanase, beta-glucanase, beta-glucosidase, and other glycoside hydrolases with known cellulolytic capabilities, involved in glycogen synthesis, including proteins such as glycogen phosphorylase, glycogen branching protein, glycogen biosynthesis protein, and 4-alphaglucan branching enzyme. A variety of glycoside hydrolases with known cellulolytic capabilities (Lee, 2017), a lot of peptidases/proteases and glutamate synthase were identified from a number of different peptidase families including endopeptidases, amino acid-specific proteases, zinc dependent peptidases/protease, and aminopeptidases (Lee, 2017). It hydrolyses starch during the fermentation process (Yang et al., 2019a) and gelatin (Fu et al., 2019), degrades polybrominated diphenyl ethers (Yang et al., 2017) and fiber (Lee, 2017), produces medium-chain carboxylic acid (Sun et al., 2020c), can effectively enhance the utilization efficiency of carbohydrates in both the fallen leaves and the sewage sludge (Yang et al., 2019a). Additionally, it has the ability to antagonise the growth of soilborne plant pathogens Fusarium sambucinum (Mohamed et al., 2017). |
| Fungi | Candida | Some studies suggest that fungi from the genus Candida are very important human and animal pathogens, inhibit the activity of antifungal drugs and increase the tolerance or resistance to them as well. many Candida species are resistant to drugs(Karpinski et al., 2021) Other members of this genus, previously believed to carry minimal disease risk, are increasingly recognised as important human pathogens, particularly because of variations in susceptibilities to widely used anti-fungal agents (Simon et al., 2016). However, some studies show that only a few species from the genus candida (near 200 species in this genus) are human opportunistic pathogens, such as candida albicans, Candida parapsilosis (Simon et al., 2016), Candida tropicalis and Candida auris, Candida glabrata (Spampinato and Leonardi, 2013; Colabella et al., 2021; Salomat, 2022),, Candida auris is multidrug-resistant (Spatafora et al., 2017). Candida albicans, Candida tropicalis, Candida parapsilosis and Candida lusitaniae can secrete aspartic proteases (Pichová et al., 2001). For instances, a candida species (a yeast) performs itaconic acid fermentation (Takeshi Tabuchi, 1981). Candida etchellsii can synthesize C6–C8 of acetate esters (Yang et al., 2021a), produce volatile flavor compounds (VFCs) in soy sauce fermentation(Feng et al., 2012; Wah et al., 2013), Candida_xylopsoci is used in fuel ethanol fermentation (Wang et al., 2021a), used for bioethanol production(Eloutassi et al., 2014), Candida xylopsoci is detected in Corn Silages (Bai et al., 2021) and traditional Chinese fermented food (Chen et al., 2016). |
| Fungi | Rhizomucor | Rhizomucor is good extracellular β-glucosidase producer (TAKÓ et al., 2010). Rhizomucor miehei in our study contains 110 glycoside hydrolases (GHs), 118 glycosyl transferases (GTs), 2 polysaccharide lyases (PLs), 20 carbohydrate esterases, 155 proteases, 97 Lipases and esterases, 15 cellulases, 16 Chitinases (Zhou et al., 2014). Rhizomucor miehei can produce 3-hydroxy-3-methylglutaryl coenzyme A reductase (Gyöngyi, 2005), highly efficient raw starch hydrolyzing r-Amylase (Georges et al., 2011), glyceraldehyde-3-phosphate dehydrogenase (Mária et al., 2004), aspartic protease (Krisch et al., 2012), lipases (Broadmeadow et al., 1994; Rodrigues and Fernandez-Lafuente, 2010), b-glucosidase (Tako et al., 2010), glyceraldehyde-3-phosphate dehydrogenase (Mária et al., 2004). The Rhizomucor miehei can produce L-asparagine amidohydrolase that catalyses the hydrolysis of L-asparagine to L-aspartic acid and ammonia (Huang et al., 2014), xylanase (Fawzi, 2010), b-1,3-1,4-glucanase(Yang et al., 2015) and utilize various substrates as a single carbon source(MA´RIA et al., 1998). However, the Rhizomucor miehei is an opportunistic pathogen may cause frequently fatal mycotic diseases (Gyöngyi et al., 2004). |
| Fungi | Rhexocercosporidium | Rhexocercosporidium is a phytopathogenic fungus commonly found in soils (Douterelo et al., 2016), can cause ginseng rusty root rot (Reeleder, 2007; Guan et al., 2020; Wei et al., 2020), rusted root of Ginseng (Reeleder et al., 2006), such as Rhexocercosporidium panacis (Punja et al., 2013), but some species of Rhexocercosporidium can control the fungal pathogens Fusarium solani (Humberto et al., 2020). |
| Fungi | Podospora | Podospora species are saprophytic, obligately coprophilous (Chris et al., 2015; Antonelli et al., 2020), cellulose degraders (Yim et al., 2017; Li et al., 2018) inhabiting the dung (Nils et al., 1999; Chris et al., 2015; Francisco et al., 2020; Zhong et al., 2022) of various herbivores (Melo et al., 2015; Zhong et al., 2022) such as rabbits, goats or horses (Mathieu and Sven, 2008) and also isolated as endophytes from trees, grass as well as herbaceous plants and soils (Josphat et al., 2011).It decay recalcitrant lignocellulose with increasing soil nutrient (Tao et al., 2020), degrade recalcitrant fraction of lignocellulose (Eric et al., 2021), degrade plant biomass due to its lignocellolytic enzymes (Yim et al., 2017) , has high litter decomposition activity, consequently result in an increase of soil fertility (He et al., 2019). It could be served as antifungal agent (Ding et al., 2017; Liu et al., 2021b), contributes to antagonism relationship with pathogenic microorganisms (Yim et al., 2017), has significant effects on controlling soil-borne diseases (Tao et al., 2020), and also enhance root growth (Yim et al., 2017). Therefore, Podospore is most abundant in healthy soils(Xu et al., 2012). |
| Fungi | Symbiotaphrina | Members of the genus are obligate gut endosymbionts (Rajib et al., 2020), capable of digesting stored-products, like dried plant and woody substrates (Purahong et al., 2017), provide nitrogen and vitamin to their hosts, to degrade the disaccharide cellobiose, and to produce lipase, α- and β-glucosidase, phosphatase and trypsin, helping the host in digesting the food and detoxifying a variety of plant materials (Irene, 2018; Liu et al., 2021a). For example, Symbiotaphrina kochii, symbiotic yeast-like species, can enzymatically detoxify and utilize mycotoxins as carbon sources (Walter et al., 2021). Symbiotaphrina is a yeast-like genus of endosymbionts that have been reported could provide nitrogen, sterols, vitamins and essential amino acids in insects (Li et al., 2022b), has a symbiotic relationship with host beetles, assisting in the digestion of food by producing enzymes such as lipase, α- and β-glucosidase, phosphatase and trypsin, and detoxifying a variety of plant toxins (e.g. 2-furaldehyde) (Rajib et al., 2020). is a yeast-like genus of endosymbionts of beetles which have been suggested to be implicated in B-vitamin biosynthesis, fatty acid metabolism, and detoxification of noxious plant compounds by insects(Diana et al., 2018). It should be noted that no harmful effect of Symbiotaphrina is reported (Li et al., 2022b). |

**References:**

Adriana, P.M., Nora, I.P., Martínez., M.A., 2015. Cellulose degrading bacteria isolated from industrial samples and the gut of native insects from Northwest of Argentina. Journal of Basic Microbiology 55, 1384-1393.

Agnieszka, C.-K., Magdalena, Z., 2018. Microbial composition of biofilm treating wastewater rich in bisphenol A. Journal of Environmental Science and Health, Part A 53, 385-392.

Akyol, Ç., Ince, O., Ince, B., 2019. Crop-based composting of lignocellulosic digestates: Focus on bacterial and fungal diversity. Bioresource Technology 288, 1-7.

Alexandria, N.I., Rachel, L.V., 2019. Bacterial communities differ between plant species and soil type, and differentially influence seedling establishment on serpentine soils. Plant Soil 441, 423-437.

Antonelli, F., Esposito, A., Galotta, G., Davidde Petriaggi, B., Piazza, S., Romagnoli, M., Guerrieri, F., 2020. Microbiota in Waterlogged Archaeological Wood: Use of Next-Generation Sequencing to Evaluate the Risk of Biodegradation. Applied Sciences 10.

Avani, B.P., Toral, M., Kunal, R.J., Chirayu, D., Datta, M., 2021. Metagenomic insights into bacterial communities’ structures in polycyclic aromatic hydrocarbons degrading consortia. Journal of Environmental Chemical Engineering 9.

Bach, A., López-García, A., González-Recio, O., Elcoso, G., Fàbregas, F., Chaucheyras-Durand, F., Castex, M., 2019. Changes in the rumen and colon microbiota and effects of live yeast dietary supplementation during the transition from the dry period to lactation of dairy cows. Journal of Dairy Science 102, 6180-6198.

Bai, C., Wang, C., Sun, L., Xu, H., Jiang, Y., Na, N., Yin, G., Liu, S., Xue, Y., 2021. Dynamics of Bacterial and Fungal Communities and Metabolites During Aerobic Exposure in Whole-Plant Corn Silages With Two Different Moisture Levels. Front Microbiol 12, 663895.

Björn, b., Bengt, v.h., Bo, g., 1968. Some Properties of Cellulolytic Cellvibrio Strains from Polluted Water. Applied Microbiology 16, 1424-1425.

Bordoloi, N., Bhagowati, P., Chaudhuri, M., Mukherjee, A., 2016. Proteomics and Metabolomics Analyses to Elucidate the Desulfurization Pathway of Chelatococcus sp. PLOS ONE 11, e0153547.

Borjigin, Q., Yu, X., Gao, J., Zhang, B., Wang, Z., Hu, S., Han, S., Sun, J., Wanji., H., 2021. Taxonomic structure and function of the corn stover degradative microbial consortium GF-20 following growth on different sources of nitrogen. Annals of Applied Biology 180, 236-246.

Borjigin, Q., Zhang, B., Yu, X., Gao, J., Zhang, X., Qu, J., Ma, D., Hu, S., Han, S., 2022. Metagenomics Study To Compare The Taxonomy And Metabolism of a Lignocellulolytic Microbial Consortium Cultured in Different Carbon Conditions. World Journal of Microbiology and Biotechnology 38, 78

Bram, W.S., Junhui, L., Benjamin, J.K., Steven, J.B., Paul, D., Michaela, H., Kirsten, S.H., Xiao-Jun, A.L., Rebecca, L.M., Ember, M.M., Jennifer, P.-R., Egbert, S., Bruce, A.H., 2021. Nutrients cause consolidation of soil carbon flux to small proportion of bacterial community. naturecommunications 12, 1-9.

Broadmeadow, A., Clare, C., De Boer, A.S., 1994. An overview of the safety evaluation of the Rhizomucor miehei lipase enzyme. Food Addit Contam 11, 105-119.

Cai, M., Zhang, H., Zhang, Y., Wu, H., 2022. Bioelectrochemical assisted landfill technology for the stabilization and valorization of food waste anaerobic digestate. Bioresource Technology 351, 1-9.

Cao, J., Li, N., Jiang, J., Xu, Y., Zhang, B., Luo, X., Hu, Y., 2022. Activated carbon as an insoluble electron shuttle to enhance the anaerobic ammonium oxidation coupled with Fe(III) reduction process. Environmental Research 204 1-9.

Chang, Y.-C., Sawada, K., Kim, E.-S., Jung, K., Kikuchi, S., 2015. Whole-Genome Sequence of Aquamicrobium sp. Strain SK-2, a Polychlorinated Biphenyl-Utilizing Bacterium Isolated from Sewage Sludge. Genome Announcements 3, e00439-00415.

Chang, Y.-C., Sugawara, H., Reddy, M.V., 2021. Validation of biphenyl degradation pathway by polymerase chain reaction, peptide mass fingerprinting and enzyme analysis. Water-Energy Nexus 4, 69-75.

Chang, Y.-C., Takada, K., Choi, D., Toyama, T., Sawada, K., Kikuchi, S., 2013. Isolation ofBiphenyl and PolychlorinatedBiphenyl-Degrading Bacteria and Their Degradation Pathway. Appl Biochem Biotechnol 170, 381-398.

Chen, H.L., Xing, X., Zhang, B., Huang, H.B., Shi, C.W., Yang, G.L., Wang, C.F., 2021. Higher mucosal type II immunity is associated with increased gut microbiota diversity in BALB/c mice after Trichinella spiralis infection. Mol Immunol 138, 87-98.

Chen, P., Zhao, Y., Wu, Z., Liu, R., Xu, R., Yan, L., Li, H., 2016. Metagenomic data of fungal internal transcribed spacer from serofluid dish, a traditional Chinese fermented food. Genom Data 7, 134-136.

Chen, R., Li, Z., Feng, J., Zhao, L., Yu, J., 2020. Effects of digestate recirculation ratios on biogas production and methane yield of continuous dry anaerobic digestion. Bioresource Technology 316, 1-8.

Chen, Y., Chang, S.K.C., Chen, J., Zhang, Q., Yu, H., 2018. Characterization of microbial community succession during vermicomposting of medicinal herbal residues. Bioresource Technology 249, 542-549.

Chintalpudi, V.K., Kanamarlapudi, R.K.S.L., Mallu, U.R., Muddada, S., 2021. Isolation, identification, biosorption optimization, characterization, isotherm, kinetic and application of novel bacterium Chelatococcus sp. biomass for removal of Pb (II) ions from aqueous solutions. International Journal of Environmental Science and Technology 19, 1531-1544.

Chou, Y.-M., Shen, F.-T., Chiang, S.-C., Chang, C.-M., 2017. Functional diversity and dominant populations of bacteria in banana plantation soils as influenced by long-term organic and conventional farming. Applied Soil Ecology 110 21-33.

Chris, N.J., Susan, R., Simon, G.H., Chris, S.M.T., A. Peter, K., Barry, W.B., 2015. Using dung fungi to interpret decline and extinction of megaherbivores: problems and solutions. Quaternary Science Reviews 110, 107-113.

Christel, M.-E., Flavie, T., Beatrice, G., Guillermo, M.B., Quentin, L.G., Didier, M., Marie-Lea, D.A., Géraldine, P., Jean-Louis, W., Annabelle, M., 2021. Apart From the Diet, the Ruminal Microbiota of Lambs Is Modified in Relation to Their Genetic Potential for Feed Efficiency or Feeding Behavior. Front Microbiol 12, 1-15.

Christopher, J.G., Jennifer, G.J., 2022. Dynamic Responses of Endosymbiotic Microbial Communities Within Microcystis Colonies in North American Lakes to Altered Nitrogen, Phosphorus, and Temperature Levels. Front Microbiol 12, 1-23.

Chuang, S.T., Li, K.Y., Tu, P.W., Ho, S.T., Hsu, C.C., Hsieh, J.C., Chen, M.J., 2021. Investigating the Reciprocal Interrelationships among the Ruminal Microbiota, Metabolome, and Mastitis in Early Lactating Holstein Dairy Cows. Animals (Basel) 11.

Claire, E.K., 2019. The Plant Growth-Promoting Potential of Root-Associated Bacteria from Plants Growing in Stressed Environments, Food and Bioproduct Sciences. University of Saskatchewan, Saskatoon, p. 140.

Colabella, C., Casagrande Pierantoni, D., Corte, L., Roscini, L., Conti, A., Bassetti, M., Tascini, C., Robert, V., Cardinali, G., 2021. Single Strain High-Depth NGS Reveals High rDNA (ITS-LSU) Variability in the Four Prevalent Pathogenic Species of the Genus Candida. Microorganisms 9, 1-19.

Couch, C.E., Stagaman, K., Spaan, R.S., Combrink, H.J., Sharpton, T.J., Beechler, B.R., Jolles, A.E., 2021. Diet and gut microbiome enterotype are associated at the population level in African buffalo. Nat Commun 12, 1-11.

Cristóbal, H.-Á., Felipe, G.-O., Rocío, C.-O., Miguel, F.R., Hugo, R.B., Daniel, P., Luis, D.A., 2022. Squash root microbiome transplants and metagenomic inspection for in situ arid adaptations. Science of The Total Environment 805, 150136.

Dai, Q., Ma, J., Cao, G., Hu, R., Zhu, Y., Li, G., Zou, H., Wang, Z., Peng, Q., Xue, B., Wang, L., 2021. Comparative study of growth performance, nutrient digestibility, and ruminal and fecal bacterial community between yaks and cattle-yaks raised by stall-feeding. AMB Express 11, 1-11.

David, W., Daniel, K., Andrea, s.D., Ann-Kathrin, G., Arne, W., Thomas, A.T., 2018. Insights into the variability of microbial community composition and micropollutant degradation in diverse biological wastewater treatment systems. Water Research 143, 313-324.

De tender, C., Mesuere, B., Van der Jeugt, F., Haegeman, A., Ruttink, t., Vandecasteele, B., Dawyndt, p., Debode, J., Kuramae, e.e., 2019. peat substrate amended with chitin modulates the N-cycle, siderophore and chitinase responses in the lettuce rhizobiome. 9.

Diana, R., Leticia, V.-A., Blanca, T., Alejandro, E.-R., Luis, S., Katy, J., 2020. Bacterial diversity in surface sediments from the continental shelf and slope of the North West gulf of Mexico and the presence of hydrocarbon degrading bacteria. Marine Pollution Bulletin 150, 1-9.

Diana, S.-R., Eric-Edmundo, H.-D., Claudia-Anahí, P.-T., Randy, O.-C., Emanuel, V., Benjamín, R.-H., Alexandro, A.-S., Abel, L.-B., Nayeli, C.-O., Lervin, H.-R., Enrique, I.-L., 2018. Environmental pH modulates transcriptomic responses in the fungus Fusarium sp. associated with KSHB Euwallacea sp. near fornicatus. BMC Genomics () : 19, 1-21.

Dimitra, K., Ioannis, S., Anastasia, T., Κaterina, V., Haralambos, S., Petros, K., 2021. Production of biomass and β-glucosidase by a novel cyanobacterium Pseudanabaena/Limnothrix sp under heterotrophic conditions. Available at SSRN: <https://ssrn.com/abstract=3954808> or <http://dx.doi.org/10.2139/ssrn.3954808>.

Ding, J., Jiang, X., Guan, D., Zhao, B., Ma, M., Zhou, B., Cao, F., Yang, X., Li, L., Li, J., 2017. Influence of inorganic fertilizer and organic manure application on fungal communities in a long-term field experiment of Chinese Mollisols. Applied Soil Ecology 111, 114-122.

Domínguez, F.F., Crisanto, M.E.V., Castro, R.L.S., Rojas, L.V., Cuba, V.M.B., Santos, G.R.S., Salazar, M.W.S., Ramos, C.A.L., Mialhe, E., 2022. Metagenomic analysis of the intestinal microbiome in goats on cactus and Salicornia-based diets. Open Veterinary Journal 12, 61-68.

Douterelo, I., Jackson, M., Solomon, C., Boxall, J., 2016. Microbial analysis of in situ biofilm formation in drinking water distribution systems: implications for monitoring and control of drinking water quality. Applied Microbiology & Biotechnology 100, 3301-3311.

Eloutassi, N., Louasté, B., Boudine, L., Chaouch, M., 2014. Valorisation of whey: Bioethanol production by free and immobilized yeasts. International Journal of Innovation and Applied Studies 6, 493-498.

Elyamine, A.M., Hu, C., 2020. Earthworms and rice straw enhanced soil bacterial diversity and promoted the degradation of phenanthrene. 32, 1-12.

Eman, K., Esmaeil, S., Nagalakshmi, H., Andrew, M.O., Andrew, S.B., 2020. Impact of necrophytoremediation on petroleum hydrocarbon degradation, ecotoxicity and soil bacterial community composition in diesel-contaminated soil. Environmental Science and Pollution Research 27, 31171-31183.

Eric, M., Noura, Z., Chantal, H., Vicky, L., Hani, A., Antoine, K., 2021. Soil microbial community dynamics after co-application of biochar and paper mill biosolids. Applied Soil Ecology 165, 1-10.

Ezequias, C.-L., Andreas, H., Renee, M.P., Wilhelm, K., Qendrim, Z., 2020. Evaluation of fecal fermentation profile and bacterial community in organically fed dairy cows consuming forage-rich diets with different particle sizes. Journal of Dairy Science 103, 8020-8033.

Fathallh Eida, M., Nagaoka, T., Wasaki, J., Kouno, K., 2012. Isolation and characterization of cellulose-decomposing bacteria inhabiting sawdust and coffee residue composts. Microbes & Environments 27, 226-233.

Fawzi, E.M., 2010. Highly thermostable purified xylanase from Rhizomucor miehei NRRL 3169. Annals of Microbiology 60, 363-368.

Federico, M.I., Eva, L.M.F., Leonardo, E., 2013. Industrial activated sludge exhibit unique bacterial community composition at high taxonomic ranks. Water Research 47, 3854-3864.

Feng, J., Zhan, X.-B., Zheng, Z.-Y., Wang, D., Zhang, L.-M., Lin, C.-C., 2012. A two-step inoculation of Candida etchellsii to enhance soy sauce flavour and quality. International Journal of Food Science & Technology 47, 2072-2078.

Filippo, C., Giovanni, N., Gabriele, C., Mauro, P., Martino, C., 2020. Characterizing the fecal bacteria and archaea community of heifers and lactating cows through 16S rRNA next-generation sequencing. Journal of Applied Genetics 61, 593-605.

Francisco, C., Vanessa, B.T., Solange, X.S., 2020. Additions to a checklist of coprophilous fungi and other fungi recorded on dung from Brazil: an overview of a century of research. Mycotaxon 135, 901.

Fu, L., Penton, C.R., Ruan, Y., Shen, Z., Xue, C., Li, R., Shen, Q., 2017. Inducing the rhizosphere microbiome by biofertilizer application to suppress banana Fusarium wilt disease. Soil Biology & Biochemistry 104, 39-48.

Fu, P.P., Xiong, F., Feng, W.W., Zou, H., Wu, S.G., Li, M., Wang, G.T., Li, W.X., 2019. Effect of intestinal tapeworms on the gut microbiota of the common carp, Cyprinus carpio. Parasites & Vectors 12.

Gaget, V., Humpage, A.R., Huang, Q., Monis, P., Brookes, J.D., 2017. Benthic cyanobacteria: A source of cylindrospermopsin and microcystin in Australian drinking water reservoirs. Water Research 124 454-464.

Ganasamurthy, S., David, R., Samad, M.S., Richards, K.G., Lanigan, G., Grelet, G.-A., Timothy J. Clough, Morales, S.E., 2021. Competition and community succession link N transformation and greenhouse gas emissions in urine patches. Science of The Total Environment 779, 1-13.

Gannes, V.d., Eudoxie, G., Hickey, W.J., 2013. Prokaryotic successions and diversity in composts as revealed by 454-pyrosequencing. Bioresource Technology 133, 573-580.

Gao, Y., Guo, L., Jin, C., Zhao, Y., Gao, M., She, Z., Wang, G., 2022. Metagenomics and network analysis elucidating the coordination between fermentative bacteria and microalgae in a novel bacterial-algal coupling reactor (BACR) for mariculture wastewater treatment. Water Research 215, 1-10.

Gaukroger, C.H., Edwards, S.A., Walshaw, J., Nelson, A., Adams, I.P., Stewart, C.J., Kyriazakis, I., 2021. Shifting sows: longitudinal changes in the periparturient faecal microbiota of primiparous and multiparous sows. Animal 15, 1-8.

Georges, T., Anders, V.-N., Agne`s, R.-S., Paul, C., Alain, B.o., 2011. In Depth Study of a New Highly Efficient Raw Starch Hydrolyzing r-Amylase from Rhizomucor sp. Biomacromolecules 12, 34-42.

Germán, T., Antonio, J.F.-G., Ana, V.L., Elisabet, A., Fernando, T., Carmen, G.-M., Manuel, F.-L., Emilio, B., Eulogio, J.B., 2021. Involvement of the metabolically active bacteria in the organic matter degradation during olive mill waste composting. Science of The Total Environment 789, 1-10.

Gou, C., Wang, Y., Zhang, X., Zhong, R., Gao, Y., 2021. Effects of chlorotetracycline on antibiotic resistance genes and the bacterial community during cattle manure composting. Bioresource Technology 323, 1-8.

Guadalupe, P., Caroline, P., Hakim, T., Katja, S., 2019. A time travel story: metagenomic analyses decipher the unknown geographical shift and the storage history of possibly smuggled antique marble statues. Annals of Microbiology 69.

Guan, Y.M., Ma, Y.Y., Jin, Q., Wang, Q.X., Liu, N., Fu, Y.P., Zhang, Y.Y., Li, Y., 2020. Multi-Locus Phylogeny and Taxonomy of the Fungal Complex Associated With Rusty Root Rot of Panax ginseng in China. Front Microbiol 11, 1-21.

Gui, L.-S., Raza, S.H.A., Allam, F.A.E.A., Zhou, L., Hou, S., Khan, I., Kakar, I.U., El-Aziz, A.H.A., Jia, J., Sun, Y., Wang, Z., 2021. Altered milk yield and rumen microbial abundance in response to concentrate supplementation during the cold season in Tibetan sheep. Electronic Journal of Biotechnology 53, 80-86.

Guo, J., Liu, W., Zhu, C., Luo, G., Kong, Y., Ling, N., Wang, M., Dai, J., Shen, Q., Guo, S., 2017. Bacterial rather than fungal community composition is associated with microbial activities and nutrient-use efficiencies in a paddy soil with short-term organic amendments. Plant Soil 424, 335–349.

Gyöngyi, L., 2005. Isolation and characterization of the gene encoding 3hydroxy-3-methylglutaryl coenzyme A reductase (HMGCoA reductase) of Rhizomucor miehei. Acta Biologica Szegediensis 49, 51.

Gyöngyi, L., Tamás, P., Ildikó, N., Erzsébet, N., Csaba, V., 2004. Differentiation of rhizomucor species on the basis of their different sensitivities to lovastatin. Journal of clinical microbiology 42, 5400-5402.

Han, F., Zhang, M., Shang, H., Liu, Z., Zhou, W., 2020. Microbial community succession, species interactions and metabolic pathways of sulfur-based autotrophic denitrification system in organiclimited nitrate wastewater. Bioresource Technology 315, 1-9.

Han, L., Shaobin, H., Zhendong, W., Pengfei, C., Yongqing, Z., 2016. Performance of a new suspended filler biofilter for removal of nitrogen oxides under thermophilic conditions and microbial community analysis. Sci Total Environ 562, 533-541.

Hao, Y., Huang, S., Si, J., Zhang, J., Gaowa, N., Sun, X., Lv, J., Liu, G., He, Y., Wang, W., Wang, Y., Li, S., 2020. Effects of Paper Mulberry Silage on the Milk Production, Apparent Digestibility, Antioxidant Capacity, and Fecal Bacteria Composition in Holstein Dairy Cows. Animals 10, 1-13.

Hatsumi, S., Yudai, T., Teruhiko, B., Kenji, U., 2010. Cohnella fontinalis sp. nov., a xylanolytic bacterium isolated from fresh water. International Journal of Systematic and Evolutionary Microbiology 60, 1344-1348.

He, C., Zheng, L., Gao, W., Ding, J., Li, C., Xu, X., Han, B., Li, Q., Wang, S., 2022. Diversity and functions of quorum sensing bacteria in the root environment of the Suaeda glauca and Phragmites australis coastal wetlands. Environmental Science and Pollution Research, 1-13.

He, Z., Chen, H., Liang, L., Dong, J., Liang, Z., Zhao, L., 2019. Alteration of crop rotation in continuous Pinellia ternate cropping soils profiled via fungal ITS amplicon sequencing. Letters in Applied Microbiology 68, 522-529.

Henderson, G., Forster, R.J., Kumar, S., Forster, R.J., Kelly, W.J., Leahy, S.C., Guan, L.L., Janssen, P.H., 2019. Improved taxonomic assignment of rumen bacterial 16S rRNA sequences using a revised SILVA taxonomic framework. PeerJ 7, 1-18.

Hernando, P.B., Chihiro, I., 2015. Polycyclic aromatic hydrocarbons (PAHs) biodegradation potential and diversity of microbial consortia enriched from tsunami sediments in Miyagi, Japan. Journal of Hazardous Materials 283, 689-697.

Hou, Y., Li, B., Feng, G., Zhang, C., He, J., Li, H., Zhu, J., 2021. Responses of bacterial communities and organic matter degradation in surface sediment to Macrobrachium nipponense bioturbation. Science of The Total Environment 759, 1-16.

Huang, C., Ge, F., Yao, X., Guo, X., Bao, P., Ma, X., Wu, X., Chu, M., Yan, P., Liang, C., 2021a. Microbiome and Metabolomics Reveal the Effects of Different Feeding Systems on the Growth and Ruminal Development of Yaks. Front Microbiol 12, 1-16.

Huang, L., Liu, Y., Sun, Y., Yan, Q., Jiang, Z., 2014. Biochemical characterization of a novel L-Asparaginase with low glutaminase activity from Rhizomucor miehei and its application in food safety and leukemia treatment. Appl Environ Microbiol 80, 1561-1569.

Huang, Y., Deng, Y., JLaw, a.C.-F., Yang, Y., Ding, J., Leung, K.S.-Y., Zhang, T., 2021b. Acesulfame aerobic biodegradation by enriched consortia and Chelatococcus spp.: Kinetics, transformation products, and genomic characterization. Water Research 202, 117454.

Huang, Z., Liu, B., Yin, Y., Liang, F., Xie, D., Han, T., Liu, Y., Yan, B., Li, Q., Huang, Y., Li, Q., 2021c. Impact of biocontrol microbes on soil microbial diversity in ginger (Zingiber officinale Roscoe). Pest Management Science 77, 5537-5546.

Humberto, E.O., Daniel, T.-M., Luis, C.-R., 2020. Patents on Endophytic Fungi for Agriculture and Bioand Phytoremediation Applications. Microorganisms 8, 1-26.

Iqbal, M.W., Zhang, Q., Yang, Y., Li, L., Zou, C., Huang, C., Lin, B., 2018. Comparative study of rumen fermentation and microbial community differences between water buffalo and Jersey cows under similar feeding conditions, , . Journal of Applied Animal Research 46, 740-748.

Irene, S., 2018. Yeast‐insect associations: It takes guts. Yeast 35, 315-330.

István, S., Sándor, S., Balázs, K., Judit, H., Péter, H., Erzsébet, B., András, T., Edit, K., Zoltán, P., József, K., 2011. Olivibacter oleidegradans sp. nov., a hydrocarbondegrading bacterium isolated from a biofilter cleanup facility on a hydrocarbon-contaminated site. International Journal of Systematic & Evolutionary Microbiology 61, 2861-2865.

Iva, P., Ivana, N., Petr, S., Xenie, K., Martin, K., Stanislav, O., 2020. Application ofosmotic challenge for enrichment ofmicrobial consortia in polyhydroxyalkanoates producing thermophilic and thermotolerant bacteria and their subsequent isolation. International Journal of Biological Macromolecules 144 698-704.

Jiang, F., Song, P., Wang, H., Zhang, J., Liu, D., Cai, Z., Gao, H., Chi, X., Zhang, T., 2022. Comparative analysis of gut microbial composition and potential functions in captive forest and alpine musk deer. Appl Microbiol Biotechnol 106, 1325-1339.

Jiao, N., Song, X., Song, R., 2022. Diversity and structure of the microbial community in rhizosphere soil of Fritillaria ussuriensis at different health levels. PeerJ 10, 1-30.

Jing, T.Z., Qi, F.H., Wang, Z.Y., 2020. Most dominant roles of insect gut bacteria: digestion, detoxification, or essential nutrient provision? Microbiome 8, 38.

Jose, V.L., Appoothy, T., More, R.P., Arun, A.S., 2017. Metagenomic insights into the rumen microbial fibrolytic enzymes in Indian crossbred cattle fed finger millet straw. AMB Express 7, 1-11.

Josphat, C.M., Birger, D., Anja, S., Hartmut, L., 2011. Larvicidal activity of metabolites from the endophytic Podospora sp. against the malaria vector Anopheles gambiae. Parasitology Research 108, 561-566.

Karpinski, T.M., Ozarowski, M., Seremak-Mrozikiewicz, A., Wolski, H., Adamczak, A., 2021. Plant Preparations and Compounds with Activities against Biofilms Formed by Candida spp. Journal of Fungi 7, 1-13.

Kazuaki, S., Yasuhito, A., Masahiro, N., Goro, T., Makoto, S., 2010. Analysis of a change in bacterial community in different environments with addition of chitin or chitosan. Journal of Bioscience and Bioengineering 109, 472-478.

Khatoon, M., Patel, S.H., Pandit, R.J., Jakhesara, S.J., Rank, D.N., Joshi, C.G., Kunjadiya, A.P., 2022. Rumen and fecal microbial profiles in cattle fed high lignin diets using metagenome analysis. Anaerobe 73 1-10.

Kimbrel, J.A., Samo, T.J., Ward, C., Nilson, D., Mayali, X., 2019. Host selection and stochastic effects influence bacterial community assembly on the microalgal phycosphere. Algal Research 40, 1-10.

Kohl, K.D., Dearing, D.M., Bordenstein, S.R., 2018. Microbial communities exhibit host species distinguishability and phylosymbiosis along the length of the gastrointestinal tract. Mol Ecol 27, 1874-1883.

Kolton, M., Harel, Y.M., Pasternak, Z., Graber, E.R., Elad, Y., Cytryn, E., 2011. Impact of Biochar Application to Soil on the Root-Associated Bacterial Community Structure of Fully Developed Greenhouse Pepper Plants. Appl Environ Microbiol, 4924–4930.

Krisch, J., Bencsik, O., Papp, T., Vagvolgyi, C., Tako, M., 2012. Characterization of a beta-glucosidase with transgalactosylation capacity from the zygomycete Rhizomucor miehei. Bioresour Technol 114, 555-560.

Kristina, U., Regina, B., Undine, B., Michael, K., Andreas, U., 2020. A Comparative Analysis of Ash Leaf-Colonizing Bacterial Communities Identifies Putative Antagonists of Hymenoscyphus fraxineus. Front Microbiol 11, 1-17.

Lee, M., Woo, S.-G., Park, J., Yoo, S.-A., 2010. Dyadobacter soli sp. nov., a starch-degrading bacterium isolated from farm soil. International Journal of Systematic and Evolutionary Microbiology 60, 2577-2582.

Lee, O., 2017. Identification of Candidate Cellulose Utilizing Bacteria from the Rumen of Beef Cattle, Using Bacterial Community Profiling and Metagenomics. Electronic Theses and Dissertations 1666, <https://openprairie.sdstate.edu/etd/1666>.

Leonardo, M., Christian, M., Gabriele, A., Francesca, T., Deborah, C., Douwe, v.S., Marco, V., 2017. Identification of universal gut microbial biomarkers of common human intestinal diseases by meta-analysis. FEMS Microbiol Ecol 93, 1-10.

Li, G., Zhu, Q., Jiang, Z., Li, M., Ma, C., Li, X., Liu, H., Liu, Y., Li, Q., 2020a. Roles of non-ionic surfactant sucrose ester on the conversion of organic matters and bacterial community structure during composting. Bioresource Technology 308, 1-9.

Li, K., Mehmood, K., Zhang, H., Jiang, X., Shahzad, M., Dong, X., Li, J., 2018. Characterization of fungus microbial diversity in healthy and diarrheal yaks in Gannan region of Tibet Autonomous Prefecture. Acta Tropica 182, 14-26.

Li, P., Liu, M., Li, G., Liu, K., Liu, T., Wu, M., Saleem, M., Li, Z., 2021. Phosphorus availability increases pathobiome abundance and invasion of rhizosphere microbial networks by Ralstonia. Environ Microbiol 23, 5992-6003.

Li, Q., Xiang, P., Zhang, T., Wu, Q., Bao, Z., Tu, W., Li, L., Zhao, C., 2022a. The effect of phosphate mining activities on rhizosphere bacterial communities of surrounding vegetables and crops. Science of The Total Environment 821, 1-13.

Li, S.-n., Hua, T., Yuan, C.S., Li, B.k., Zhu, X.y., Li, F.x., 2020b. Degradation pathways, microbial community and electricity properties analysis of antibiotic sulfamethoxazole by bio-electro-Fenton system. Bioresource Technology 298, 1-9.

Li, S., Wu, Z., Liu, G., 2019a. Degradation kinetics of toilet paper fiber during wastewater treatment: Effects of solid retention time and microbial community. Chemosphere 225, 915-926.

Li, X., Zhang, Y., Yi, Y., Shan, Y., Liu, B., Zhou, Y., Wang, X., Lü, X., 2022b. Revealing the effects of Moringa oleifera Lam. leaves addition on Fuzhuan Brick Tea by metabolomic and microbiota analysis. LWT - Food Science and Technology 156, 1-9.

Li, Y., Chen, Z., Peng, Y., Zheng, K., Ye, C., Wan, K., Zhang, S., 2020c. Changes in aerobic fermentation and microbial community structure in food waste derived from different dietary regimes. Bioresource Technology 317, 1-12.

Li, Z., Si, H., Nan, W., Wang, X., Zhang, T., Li, G., 2019b. Bacterial community and metabolome shifts in the cecum and colon of captive sika deer (Cervus nippon) from birth to post weaning. FEMS Microbiology Letters 366, 1-13.

Liang, W., Huang, S., 2015. Isolation of a thermophilic aerobic denitrifier and characterization for its denitrification performance. Desalination and Water Treatment 10, 1-4.

Liang, W., Huang, S., Liu, J., Zhang, R., Yan, F., 2012. Removal of nitric oxide in a biotrickling filter under thermophilic condition using Chelatococcus daeguensis. Journal of the Air & Waste Management Association 62, 509-516.

Liao, Q., Huang, L., Zhen, Y., Qin, Y., Jiang, Q., Lan, T., Shen, P., 2021. Effects of dietary Metschnikowia sp. GXUS03 on growth, immunity, gut microbiota and Streptococcus agalactiae resistance of Nile tilapia (Oreochromis niloticus). Aquaculture Research 53, 1918-1927.

Lin, P., Yan, Z.F., Li, C.T., 2020. Luteimonas cellulosilyticus sp. nov., Cellulose‑Degrading Bacterium Isolated from Soil in Changguangxi National Wetland Park, China. Current Microbiology 77, 1341-1347.

Lin, Y., Zhang, Y., Liang, X., Duan, R., Yang, L., Du, Y., Wu, L., Huang, J., Xiang, G., Bai, J., Zhen, Y., 2022. Assessment of rhizosphere bacterial diversity and composition in a metal hyperaccumulator (Boehmeria nivea) and a nonaccumulator (Artemisia annua) in an antimony mine. Journal of Applied Microbiology 132, 3432-3443.

Liu, H., Hall, M.A., Brettell, L.E., Halcroft, M., Wang, J., Nacko, S., Spooner-Hart, R., Cook, J.M., Riegler, M., Singh, B., 2021a.

Liu, H., Qi, Y., Wang, J., Jiang, Y., Geng, M., 2021b. Synergistic effects of crop residue and microbial inoculant on soil properties and soil disease resistance in a Chinese Mollisol. Sci Rep 11, 1-14.

Liu, S., Shah, A.M., Yuan, M., Kang, K., Wang, Z., Wang, L., Xue, B., Zou, H., Zhang, X., Yu, P., Wang, H., Tian, G., Peng, Q., 2021c. Effects of dry yeast supplementation on growth performance, rumen fermentation characteristics, slaughter performance and microbial communities in beef cattle. Animal Biotechnology 31.

Liu, X., Tang, Z., Zhang, Q., Kong, W., 2021d. The contrasting effects of biochar and straw on N2O emissions in the maize season in intensively farmed soil. Environmental Science and Pollution Research 28, 29806-29819.

Liu, X., Wang, C., Ji, M., 2013. Metabolic Characteristic Analysis of an Oil Field Wastewater Degrading Strain Chelatococcus G5. Applied Mechanics and Materials 260-261, 684-689.

Liu, Y., Cheng, D., Xue, J., Weaver, L., Wakelin, S.A., Feng, Y., Li, Z., 2020a. Changes in microbial community structure during pig manure composting and its relationship to the fate of antibiotics and antibiotic resistance genes. Journal of Hazardous Materials 389, 1-9.

Liu, Y., Michael, D.-S., Marc, M., Hu, H.-W., Glenn, B., Helen, B.-J., 2020b. Antibiotic Resistance Genes in Antibiotic-Free Chicken Farms. Antibiotics 9, 1-12.

Lu, S., Mikkelsen, D., Yao, H., Williams, B.A., Flanagan, B.M., Gidley, M.J., 2021. Wheat cell walls and constituent polysaccharides induce similar microbiota profiles upon in vitro fermentation despite different short chain fatty acid end-product levels. Food Funct 12, 1135-1146.

Lu, Y., Zheng, G., Zhou, W., Wang, J., Zhou., L., 2019. Bioleaching conditioning increased the bioavailability of polycyclic aromatic hydrocarbons to promote their removal during co-composting of industrial and municipal sewage sludges. Science of The Total Environment 665, 1073-1082.

Luo, X., Zhang, W., Dai, J., Lei, Z., Fang, C., 2010. Cohnella damensis sp nov. a motile xylanolytic bacteria isolated from a low altitude area in tibet. , . Journal of Microbiology and Biotechnology 20, 410-414.

Ma, C., Lo, P.K., Xu, J., Li, M., Jiang, Z., Li, G., Zhu, Q., Li, X., Leong, S.Y., Li, Q., 2020a. Molecular mechanisms underlying lignocellulose degradation and antibiotic resistance genes removal revealed via metagenomics analysis during different agricultural wastes composting. Bioresource Technology 314, 1-10.

Ma, J., Shah, A.M., Shao, Y., Wang, Z., Zou, H., Hu, R., Peng, Q., Kang, K., Wanapat, M., 2020b. Effects of yeast cell wall on the growth performance, ruminal fermentation, and microbial community of weaned calves. Livestock Science 239, 1-8.

Ma, J., Shah, A.M., Wang, Z., Fan, X., 2021. Potential protective effects of thiamine supplementation on the ruminal epithelium damage during subacute ruminal acidosis. Anim Sci J 92, e13579.

Ma, J., Zhu, Y., Wang, Z., Yu, X., Hu, R., Wang, X., Cao, G., Zou, H., Shah, A., Peng, Q., Xue, B., Wang, L., Zhao, S., Kong, X., 2020c. Comparing the Bacterial Community in the Gastrointestinal Tracts Between Growth-Retarded and Normal Yaks on the Qinghai–Tibetan Plateau. Front Microbiol 11, 1-15.

MA´RIA, V., TAMA´S, P., ZSOLT, K., CSABA, V.G.L., 1998. Differentiation of Rhizomucor Species by Carbon Source Utilization and Isoenzyme Analysis. Journal of clinical microbiology 36, 2153-2156.

Madhusmita, M., Pratiksha, B., Kim, J.Y., Rastogi, G., 2020. Seasonal and spatial dynamics of bacterioplankton communities in a brackish water coastal lagoon. Science of The Total Environment 705, 1-13.

Margarete, B.-W., Thomas, E., 2001. Environmental fate and microbial degradation of aminopolycarboxylic acids. FEMS Microbiology Reviews () 25, 69-106.

Mária, V., Zsolt, K., Klára, Á., Tamás, P., Helmut, S., Csaba, V., 2004. Cloning and sequence analysis of the glyceraldehyde-3-phosphate dehydrogenase gene from the zygomycetes fungus Rhizomucor miehei. Antonie Van Leeuwenhoek 86, 111-119.

Marie, U., Franz, B., Silvia, G., Tillmann, L., Susanne, K., Timo, K., Miriam, A., Ulrich, K., Jean, C.M., Michael, S., Doreen, F., 2017. Bacteria utilizing plant-derived carbon in the rhizosphere of triticum aestivum change in different depths of an arable soil. Environmental Microbiology Reports 9, 729-741.

Mathieu, P., Sven, J.S., 2008. The genome sequence of Podospora anserina, a classic model fungus. Genome biology 9, 1-4.

Maya, O.-L., Noa, S., Milana, G.-V., Stefan, J.G., Yitzhak, H., Dror, M., 2014. Niche and host-associated functional signatures of the root surface microbiome. Nat Commun 5, 1-9.

Meier, M.A., Lopez-Guerrero, M.G., Guo, M., Schmer, M.R., Herr, J.R., Schnable, J.C., Alfano, J.R., Yang, J., 2021. Rhizosphere Microbiomes in a Historical Maize-Soybean Rotation System Respond to Host Species and Nitrogen Fertilization at the Genus and Subgenus Levels. Appl Environ Microbiol 87, e03132-03120.

Mekdimu, M.D., Jingyeong, S., Hyun, M.J., Hyun, U.C., Jinhua, W., Young, M.K., 2021. Effects of biological pretreatments of microalgae on hydrolysis, biomethane potential and microbial community. Bioresource Technology 329, 1-10.

Melo, R., Miller, A., Maia, L., 2015. The genus Podospora (Lasiosphaeriaceae, Sordariales) in Brazil. Mycosphere 6, 201-215.

Mohamed , R., Groulx, E., Defilippi, S., Erak, T., Tambong, J.T., Tweddell, R.J., Tsopmo, A., Avis, T.J., 2017. Physiological and molecular characterization of compost bacteria antagonistic to soilborne plant pathogens. Canadian Journal of Microbiology 63, 1-47.

Mohapatra, B.R., Duc, M.T.L., 2013. Detecting the dormant: a review of recent advances in molecular techniques for assessing the viability of bacterial endospores. Applied Microbiology & Biotechnology 97, 7963-7975.

Nagalakshmi, H., Esmaeil, S., Sohni, S.J., Eman, K., Vivek, B.R., Paul D., M., A. Mark, O., Andrew, S.B., 2019. Influence of bioaugmentation and biostimulation on PAH degradation in aged contaminated soils: Response and dynamics of the bacterial community. J Environ Manage 238.

Nakajima-Kambe, T., Okada, N., Takeda, M., Akutsu-Shigeno, Y., Matsumura, M., Nomura, N., Uchiyama, H., 2005. Screening of Novel Cellulose-Degrading Bacterium and Its Application to Denitrification of Groundwater. Journal of Bioscience & Bioengineering 99, 429-433.

Nasrin, A., Saeed, A., Ali, A.K., Kamahldin, H., 2016. Thermostable chitinase from Cohnella sp. A01: isolation and product optimization. Brazilian journal of microbiology 47, 931-940.

Nasser, K., Horst, K., Brendan, W., 2020. Metabarcoding Analysis of Bacterial Communities Associated with Media Grow Bed Zones in an Aquaponic System. International Journal of Microbiology 2020, 1-10.

Nataliia, K., Małgorzata, S., Łukasz, Ł., Małgorzata, S.-M., Monika, C., Hanna, M.-M., Marta, C., Toirbek, N., Susanna, A.W., Jonathan, P., Jan, K., Iwona, J., 2020. Limited Microcystin, Anatoxin and Cylindrospermopsin Production by Cyanobacteria from Microbial Mats in Cold Deserts. Toxins 12, 1-19.

Nils, L., Daniel, P.M., Ann, B., Laura, E.L., 1999. Podospora austrohemisphaerica, a new heterothallic ascomycete from dung. Mycologia 91, 405-415.

Onotasamiderhi, T.I., Paola, M., Russell, J.D., David, W., 2019. Impacts of activated carbon amendments, added from the start or after five months, on the microbiology and outcomes of crude oil bioremediation in soil. International Biodeterioration & Biodegradation 142, 1–10.

Pan, X., Nan, X., Yang, L., Jiang, L., Xiong, B., 2018. Thiamine status, metabolism and application in dairy cows: a review. Br J Nutr 120, 491-499.

Pan, X., Xue, F., Nan, X., Tang, Z., Wang, K., Beckers, Y., Jiang, L., Xiong, B., 2017. Illumina Sequencing Approach to Characterize Thiamine Metabolism Related Bacteria and the Impacts of Thiamine Supplementation on Ruminal Microbiota in Dairy Cows Fed High-Grain Diets. Front Microbiol 8, 1-10.

Park, T., Cersosimo, L.M., Li, W., Radloff, W., Zanton, G.I., 2021. Pre-weaning Ruminal Administration of Differentially-Enriched, Rumen-Derived Inocula Shaped Rumen Bacterial Communities and Co-occurrence Networks of Post-weaned Dairy Calves. Front Microbiol 12, 1-18.

Parmeshwar, V.G., Arijita, B., Subhajit, S., Khusboo, L., Nensina, M., Vijeta, R., Deepika, M., Shyama, P.S., Vaskar, D., Shilpi, G., 2021 Functional characterization of thermotolerant microbial consortium for lignocellulolytic enzymes with central role of Firmicutes in rice straw depolymerization. Sci Rep 11, 1-13.

Peter, K., Elena, M., Nicole, L., Udo, J., 2009. Transfer of Defluvibacter lusatiensis to the genus Aquamicrobium as Aquamicrobium lusatiense comb. nov. and description of Aquamicrobium aerolatum sp. nov. International Journal of Systematic and Evolutionary Microbiology 59, 2468–2470.

Pia, A.W., Jens, E.J., Ulrich, K., Bjarne, M.H., 2005. Isolation and taxonomic affiliation of N-heterocyclic aromatic hydrocarbon-transforming bacteria. Applied Microbiology & Biotechnology 67, 420–428.

Pichová, I., Libuše Pavlíčkov, J Dostál, Elena Dolejš, Olga Hrušková-Heidingsfeldov, Weber, J., Ruml, T., Souček, M., 2001. Secreted aspartic proteases of Candida albicans, Candida tropicalis, Candida parapsilosis and Candida lusitaniae. European Journal of Biochemistry 268, 2669-2677.

Punja, Z.K., Wan, A., Leippi, L., Goswami, R.S., Jayaraj, J., 2013. Growth, pathogenicity and infection behaviour, and genetic diversity of Rhexocercosporidium panicis isolates from ginseng roots in British Columbia. Canadian Journal of Plant Pathology 35, 503-513.

Purahong, W., Pietsch, K.A., Lentendu, G., Schöps, R., Bruelheide, H., Wirth, C., Buscot, F., Wubet, T., 2017. Characterization of Unexplored Deadwood Mycobiome in Highly Diverse Subtropical Forests Using Culture-independent Molecular Technique. 8, 1-17.

Qiu, Q., Gao, C., Rahman, M.A.u., Cao, B., Su, H., 2020. Digestive Ability, Physiological Characteristics, and Rumen Bacterial Community of Holstein Finishing Steers in Response to Three Nutrient Density Diets as Fattening Phases Advanced. Microorganisms 8, 1-17.

Qiu, X., Qin, X., Chen, L., Chen, Z., Hao, R., Zhang, S., Yang, S., Wang, L., Cui, Y., Li, Y., Ma, Y., Cao, B., Su, H., 2022. Serum Biochemical Parameters, Rumen Fermentation, and Rumen Bacterial Communities Are Partly Driven by the Breed and Sex of Cattle When Fed High-Grain Diet. Microorganisms 10, 1-13.

Qu, Y., Zhang, X., Ma, Q., Deng, J., Deng, Y., Nostrand, J.D.V., Wu, L., He, Z., Qin, Y., Zhou, J., Zhou, J., 2015. Microbial Community Dynamics and Activity Link to Indigo Production from Indole in Bioaugmented Activated Sludge Systems. PLOS ONE 10, e013845.

Qu, Y., Zhang, X., Shen, W., Ma, Q., You, S., Pei, X., Li, S., Ma, F., Zhou, J., 2016. Illumina MiSeq sequencing reveals long-term impacts of single-walled carbon nanotubes on microbial communities of wastewater treatment systems. Bioresource Technology 211, 209-215.

Rabee, A.E., Alahl, A.A.S., Lamara, M., Ishaq, S.L., 2022. Fibrolytic rumen bacteria of camel and sheep and their applications in the bioconversion of barley straw to soluble sugars for biofuel production. PLOS ONE 17, e0262304.

Rajib, M., Brodie, S., Phillip, W.T., Toni, A.c., 2020. fruit host‑dependent fungal communities in the microbiome of wild Queensland fruit fly larvae. Sci Rep 10, 1-12.

Reeleder, R.D., 2007. Rhexocercosporidium panacis sp. nov., a new anamorphic species causing rusted root of ginseng (Panax quinquefolius). Mycologia 99, 91-98.

Reeleder, R.D., Hoke, S.M.T., Zhang, Y., 2006. Rusted Root of Ginseng (Panax quinquefolius) Is Caused by a Species of Rhexocercosporidium. 96, 1243-1254.

Reid, T., Droppo, I.G., Weisener, C.G., 2020. Tracking functional bacterial biomarkers in response to a gradient of contaminant exposure within a river continuum. Water Research 168, 1-12.

Ren, Q., Si, H., Yan, X., Liu, C., Ding, L., Long, R., Li, Z., Qiu, Q., 2020. Bacterial communities in the solid, liquid, dorsal, and ventral epithelium fractions of yak (Bos grunniens) rumen. MicrobiologyOpen 9, 1-16.

Ribeiro, G.O., Gruninger, R.J., Jones, D.R., Beauchemin, K.A., Yang, W.Z., Wang, Y., Tsang, A., McAllister., T.A., 2020. Effect of ammonia fiber expansion-treated wheat straw and a recombinant fibrolytic enzyme on rumen microbiota and fermentation parameters, total tract digestibility, and performance of lambs. Journal of Animal Science, 1-19.

Robledo, M., Gomez-Silvan, C., Andersen, G.L., Calvo, C., Aranda, E., 2020. Assessment of bacterial and fungal communities in a full-scale thermophilic sewage sludge composting pile under a semipermeable cover. Bioresour Technol 298, 122550.

Rodrigues, R.C., Fernandez-Lafuente, R., 2010. Lipase from Rhizomucor miehei as a biocatalyst in fats and oils modification. Journal of Molecular Catalysis B: Enzymatic 66, 15-32.

Romanenko, L.A., Tanaka, N., Frolova, G.M., 2010. Umboniibacter marinipuniceus gen. nov., sp. nov., a marine gammaproteobacterium isolated from the mollusc Umbonium costatum from the Sea of Japan. International Journal of Systematic and Evolutionary Microbiology 60, 603-609.

Rubén, F., Iván, L., Carlos, S., Diego, B., José, L.R.G., Ana, I.P., 2020. Bioaugmentation Treatment of a PAH-Polluted Soil in a Slurry Bioreactor. Applied Sciences 10, 1-17.

Sakellaris, H., Manners, J.M., Pemberton, J.M., 1997. A Gene Encoding an Exo-␤-Glucosidase from Cellvibrio mixtus. Current Microbiology 35, 228-232.

Sakellaris, H., Pemberton, J.M., Manners, J.M., 1990. Genes from Cellvibrio mixtus Encoding 1-1,3 Endoglucanase. Appl Environ Microbiol, 3204-3208.

Sakellaris, H., Pemberton, J.M., Manners, J.M., 1993. Characterization of an endo-1,3(4)-β-D-glucanase gene from Cellvibrio mixtus. FEMS Microbiology Letters 109, 269-272.

Salomat, M.A., 2022. Determination of the disribution of candida fungi in patients with chronic generalized periodontitis. Academic Research in Educational Sciences 3, 292-295.

Saowapar, K., Somboon, T., Ancharida, A., Kwang, K.K., Keun, C.L., Jung-Sook, L., 2010a. Cohnella thailandensis sp. nov., a xylanolytic bacterium from Thai soil. International Journal of Systematic and Evolutionary Microbiology 60, 2284-2287.

Saowapar, K., Somboon, T., Ancharida, A., Kwang, K.K., Keun, C.L., Jung-Sook, L., 2010b. Cohnella xylanilytica sp. nov. and Cohnella terrae sp. nov., xylanolytic bacteria from soil. International Journal of Systematic and Evolutionary Microbiology 60, 2913-2917.

Saowapar, K., Somboon, T., Ancharida, A., Kwang, K.K., Keun, C.L., Jung-Sook, L., 2012. Cohnella cellulosilytica sp. nov., isolated from buffalo faeces. International Journal of Systematic and Evolutionary Microbiology 62, 1921-1925.

Saurabh, K., Deep, C.S., Mamta, B., Reeta, G., 2018. Plant growth promoting potential of psychrotolerant Dyadobacter sp. for pulses and finger millet and impact of inoculation on soil chemical properties and diazotrophic abundance. Journal of Plant Nutrition 41, 1-13.

Senevirathna, S.T.M.L.D., Krishna, K.C.B., Mahinroosta, R., Sathasivan, A., 2022. Comparative characterization of microbial communities that inhabit PFAS-rich contaminated sites: A case-control study. Journal of Hazardous Materials 423, 1-11.

Siddarthan, V., Vatharamattathil, M.K., Vadakke, N.S., Dinesh, S.L., Mahesh, M., Kottekkatu, P.K., 2021. Bacterial diversity and community structure along the glacier foreland of Midtre Lov´enbreen, Svalbard, Arctic. Ecological Indicators 126, 1-9.

Simon, J.S.C., Frances, B., Alvaro, P.-M., Tony, R., Kate, H., Alireza, A., Adam, B., Zsolt, B., Tamas, K., Daniel, S., Richard, S., Monica, R., Julia, B., Zoltan, T., 2016. Rapid Evaporative Ionisation Mass Spectrometry (REIMS) Provides Accurate Direct from Culture Species Identification within the Genus Candida. Sci Rep 6, 1-10.

Singh, D.N.a.A.K.T., 2011. Evaluation of the Coal-Degrading Ability of Rhizobium and Chelatococcus Strains Isolated from the Formation Water of an Indian Coal Bed. Journal of Microbiology & Biotechnology 21, 1101–1108.

Song, B., Wu, T., You, P., Wang, H., Burke, J.L., Kang, K., Yu, W., Wang, M., Li, B., He, Y., Huo, Q., Li, C., Tian, W., Li, R., Li, J., Wang, C., Sun, X., 2021. Dietary Supplementation of Yeast Culture Into Pelleted Total Mixed Rations Improves the Growth Performance of Fattening Lambs. Front Vet Sci 8, 657816.

Song, H.-L., Lu, Y.-X., Yang, X.-L., Xu, H., Singh, R.P., Du, K.-X., Yang, Y.-L., 2020. Degradation of sulfamethoxazole in low-C/N ratio wastewater by a novel membrane bioelectrochemical reactor. Bioresource Technology 305, 1-8.

Spampinato, C., Leonardi, D., 2013. Candida infections, causes, targets, and resistance mechanisms: traditional and alternative antifungal agents. Biomed Res Int 2013, 204237.

Spatafora, J.W., Aime, M.C., Grigoriev, I.V., Martin, F., Stajich, J.E., Blackwell, M., 2017. The Fungal Tree of Life: from Molecular Systematics to Genome-Scale Phylogenies. Microbiol Spectr 5.

Su, H., Zhang, D., Antwi, P., Xiao, L., Luo, W., Deng, X., Lai, C., Liu, Z., Shi, M., Manefield, M.J., 2021. Unraveling the effects of light rare-earth element (Lanthanum (III)) on the efficacy of partial-nitritation process and its responsible functional genera. Chemical Engineering Journal 408.

Sun, C., Wei, Y., Kou, J., Han, Z., Shi, Q., Liu, L., Sun, Z., 2021. Improve spent mushroom substrate decomposition, bacterial community and mature compost quality by adding cellulase during composting. Journal of Cleaner Production 299, 1-11.

Sun, J., Li, N., Yang, P., Zhang, Y., Yuan, Y., Lu, X., Zhang, H., 2020a. Simultaneous antibiotic degradation, nitrogen removal and power generation in a microalgaebacteria powered biofuel cell designed for aquaculture wastewater treatment and energy recovery. 45, 10871-10881.

Sun, J., Xu, W., Yuan, Y., Lu, X., Kjellerup, B.V., Xu, Z., Zhang, H., Zhang, Y., 2019. Bioelectrical power generation coupled with high-strength nitrogen removal using a photo-bioelectrochemical fuel cell under oxytetracycline stress. Electrochimica Acta 299, 500-508.

Sun, J., Yang, L., Wei, J., Quan, J., Yang, X., 2020b. The responses of soil bacterial communities and enzyme activities to the edaphic properties of coal mining areas in Central China. PLOS ONE 15, e0231198.

Sun, L., Zhang, Y., Chen, W., Lan, T., Wang, Y., Wu, Y., Liao, X., Mi, J., 2020c. The Dynamic Changes of Gut Microbiota during the Perinatal Period in Sows. Animals 10, 1-14.

Takeshi Tabuchi, T.S., Tsuguo Ishidori, Tadaatsu Nakahara & Junta Sugiyama 1981. Itaconic Acid Fermentation by a Yeast Belonging to the Genus Candida. Agricultural and Biological Chemistry 45, 475-479.

TAKÓ, M., FARKAS, E., LUNG, S., KRISCH, J., VÁGVÖLGYI, C., PAPP, T., 2010. Identification of acid- and thermotolerant extracellular β-glucosidase activities in Zygomycetes fungi. Acta Biologica Hungarica 61, 101-110.

Tako, M., Toth, A., L, G.N., Krisch, J., Vagvolgyi, C., Papp, T., 2010. A new beta-glucosidase gene from the zygomycete fungus Rhizomucor miehei. Antonie Van Leeuwenhoek 97, 1-10.

Tang, X., Yang, Y., Huang, Wenda,, McBride, Murray B.,, Guo, J., Tao, R., Dai, Y., 2017. Transformation of chlorpyrifos in integrated recirculating constructed wetlands (IRCWs) as revealed by compound-specific stable isotope (CSIA) and microbial community structure analysis. Bioresource Technology 233 264-270.

Tao, R., Hu, B., Chu, G., 2020. Impacts of organic fertilization with a drip irrigation system on bacterial and fungal communities in cotton field. Agricultural Systems 182, 1-11.

Thieringer, P.H., Honeyman, A.S., Spear, J.R., 2021. Spatial and Temporal Constraints on the Composition of Microbial Communities in Subsurface Boreholes of the Edgar Experimental Mine. Microbiol Spectrum 9, e00631-00621.

Tian, L., Guan, T.W., Yang, L.L., Lu, K.Y., Liu, B.B., ChunYu, W.X., Yin, M., Li, E.Y., Ji, Y., Zhang, X.P., Tang, S.K., 2017. Olivibacter flavus sp. nov., a novel endophytic bacterium isolated from the root of Camellia sinensis. Archives of Microbiology 199, 1237-1242.

Tian, W., Sun, Q., Xu, D., Zhang, Z., Chen, D., Li, C., Shen, Q., Shen, B., 2013. Succession of bacterial communities during composting process as detected by 16S rRNA clone libraries analysis. International Biodeterioration & Biodegradation 78, 58-66.

Wah, T.T., Walaisri, S., Assavanig, A., Niamsiri, N., Lertsiri, S., 2013. Co-culturing of Pichia guilliermondii enhanced volatile flavor compound formation by Zygosaccharomyces rouxii in the model system of Thai soy sauce fermentation. Int J Food Microbiol 160, 282-289.

Walter, P.P., István, P., Zoltán, G., Tünde, P., 2021. The Aspergilli and Their Mycotoxins: Metabolic Interactions With Plants and the Soil Biota. Front Microbiol 10, 1-21.

Wang, C., Sun, L., Xu, H., Na, N., Yin, G., Liu, S., Jiang, Y., Xue, Y., 2021a. Microbial Communities, Metabolites, Fermentation Quality and Aerobic Stability of Whole-Plant Corn Silage Collected from Family Farms in Desert Steppe of North China. Processes 9.

Wang, J.m., Gan, X.m., Pu, F.j., Wang, W.x., Ma, M., Sun, L.l., Hu, J.w., Hu, B., Zhang, R.p., Bai, L.l., Li, L., Liu, H.h., 2021b. Effect of fermentation bed on bacterial growth in the fermentation mattress material and cecum of ducks. Archives of Microbiology 203, 1489-1497.

Wang, L.-Y., Chen, S.-F., Wang, L., Zhou, Y.-G., Liu, H.-C., 2012. Cohnella plantaginis sp. nov., a novel nitrogen-fixing species isolated from plantain rhizosphere soil. Antonie Van Leeuwenhoek 102, 83-89.

Wang, L.-Y., Wang, T.-S., Chen, S.-F., 2015a. Cohnella capsici sp. nov., a novel nitrogen-fixing species isolated from Capsicum annuum rhizosphere soil, and emended description of Cohnella plantaginis. Antonie Van Leeuwenhoek 107, 133-139.

Wang, L., Chen, L., Ling, Q., Li, C.-c., Tao, Y., Wang, M., 2015b. Dyadobacter jiangsuensis sp. nov., a methyl red degrading bacterium isolated from a dye-manufacturing factory. International Journal of Systematic and Evolutionary Microbiology 65, 1138-1143.

Wang, L., Zheng, J., Huang, X., 2021c. Co-composting materials can further affect the attenuation of antibiotic resistome in soil application. Waste Management 135, 329-337.

Wang, T.T., Sun, Z.Y., Wang, S.P., Tang, Y.Q., Kida, K., 2020. Succession of Total and Active Microbial Community During the Composting of Anaerobic Digested Residue. Waste and Biomass Valorization 11, 4677-4689

Wang, X., Yang, H.-X., Zhang, Y.-K., Zhu, S.-J., Liu, X.-W., Zhang, H., Zhang, C.-F., Zhao, C.-R., Hu, G., Hong, Q., 2015c. Luteimonas soli sp. nov., isolated from farmland soil. International Journal of Systematic and Evolutionary Microbiology 65, 4809-4815.

Wang, Y., Cui, D., Li, A., Yang, J., Ma, F., 2016. Complete genome sequence of Chelatococcus sp. CO-6, a crude-oil-degrading bacterium. Journal of Biotechnology 219, 20-21.

Wei, X., Wang, X., Cao, P., Gao, Z., Chen, A.J., Han, J., 2020. Microbial community changes in the rhizosphere soil of healthy and rusty Panax ginseng and discovery of pivotal fungal genera associated with rusty roots. Biomed Res Int 2020, 8018525.

Wilberg, E., El-Banna, T., Auling, G., Egli, T., 1993. Serological studies on nitrilotriacetic acid (nta)-utilizing bacteria: distribution of chelatobacter heintzii and chelatococcus asaccharovorans in sewage treatment plants and aquatic ecosystems. Systematic & Applied Microbiology 16, 147-152.

Wu, C., le, C., Gao, T., Zheng, Y., Ji, J., 2021. Isolation and Identification of Aquamicrobium Strains from a Fecal Contaminated Sludge Sample. MEDS Public Health and Preventive Medicine 1, 18-22.

Wu, Y.-R., He, J., 2015. Characterization of a xylanaseproducing Cellvibrio mixtus strain J3-8 and its genome analysis. Sci Rep 5, 1-11.

Wu, Y.-R., Lin, B., Yu, Y., 2014a. Draft Genome Sequence of a Xylanase-Producing Bacterial Strain, Cellvibrio mixtus J3-8. Genome Announcements 2, e01281-01214.

Wu, Z.-G., Zhang, Y.-P., Wu, X.-W., Wang, F., Gu, C.-G., Yang, Z.-Z., Jiang, X., 2014b. Aquamicrobium terrae sp. nov., isolated from the polluted soil near a chemical factory. Antonie Van Leeuwenhoek 105, 1131-1137.

Xi Zhang, Qianqian Zhang, Liping Zhan, Xintong Xu, Ruiyu Bi, Zhengqin Xiong, 2022. Biochar addition stabilized soil carbon sequestration by reducing temperature sensitivity of mineralization and altering the microbial community in a greenhouse vegetable field. J Environ Manage 313, 1-11.

Xiang, K., Hu, X., Mu, R., Li, S., Wang, Y., Zhao, C., Zhang, N., Fu, Y., 2021. Rumen Microbiota Alterations During Ketosis is Associated with the Development of Mastitis in Dairy Cows. Research square, 1-25.

Xiao, E., Ning, Z., Xiao, T., Sun, W., Qiu, Y., Zhang, Y., Chen, J., Gou, Z., Chen, Y., 2019. Variation in rhizosphere microbiota correlates with edaphic factor in an abandoned antimony tailing dump. Environmental Pollution 253, 141-151.

Xie, Z., Lin, W., Luo, J., 2015. Genome sequence of Cellvibrio pealriver PR1, a xylanolytic and agarolytic bacterium isolated from freshwater. Journal of Biotechnology 214, 57-58.

Xie, Z., Lin, W., Luo, J., 2017. Comparative Phenotype and Genome Analysis of Cellvibrio sp. PR1, a Xylanolytic and Agarolytic Bacterium from the Pearl River. Biomed Research International, 1-10.

Xu, C.-F., Zhang, L., Huang, J.-W., Chen, K., Li, S.-P., Jiang, J.-D., 2017. Aquamicrobium soli sp. nov., a bacterium isolated from a chlorobenzoate-contaminated soil. Antonie Van Leeuwenhoek 110, 305-312.

Xu, F., Huang, S., Liu, Y., Zhang, Y., Chen., S., 2014. Comparative study on the production of poly(3-hydroxybutyrate) by thermophilic Chelatococcus daeguensis TAD1: a good candidate for large-scale production. Appllied Microbiology & Biotechnology 98, 1-10.

Xu, J., Li, C., Zhu, N., Shen, Y., Yuan, H., 2021. Particle size-dependent behavior of redox-active biochar to promote anaerobic ammonium oxidation (anammox). Chemical Engineering Journal 410, 1-9.

Xu, L., Ravnskov, S., Larsen, J., Nilsson, R.H., Nicolaisen, M., 2012. Soil fungal community structure along a soil health gradient in pea fields examined using deep amplicon sequencing. Soil Biology & Biochemistry 46, 26-32.

Xu, S., Tian, L., Chang, C., Li, X., Tian, C., 2019. Plants exhibit significant effects on the rhizospheric microbiome across contrasting soils in tropical and subtropical China. FEMS Microbiol Ecol 95, 1-9.

Xue, F., Nan, X., Li, Y., Pan, X., Guo, Y., Jiang, L., Xiong, B., 2019. Metagenomic insights into effects of thiamine supplementation on ruminal non-methanogen archaea in high-concentrate diets feeding dairy cows. BMC Vet Res 15, 7.

Xue, F., Nan, X., Sun, F., Pan, X., Guo, Y., Jiang, L., Xiong, B., 2018. Metagenome sequencing to analyze the impacts of thiamine supplementation on ruminal fungi in dairy cows fed high-concentrate diets. AMB Express 8, 159.

Yan, X.T., Yan, B.Y., Ren, Q.M., Dou, J.J., Wang, W.W., Zhang, J.J., Zhou, J.W., Long, R.J., Ding, L.M., Han, J., Li, Z.P., Qiu, Q., 2018. Effect of slow-release urea on the composition of ruminal bacteria and fungi communities in yak. Animal Feed Science and Technology 244, 18-27.

Yang, C.-W., Huang, H.-W., Chang, B.-V., 2017. Microbial communities associated with anaerobic degradation of polybrominated diphenyl ethers in river sediment. Journal of Microbiology, Immunology and Infection 50, 32-39.

Yang, C., Tsedan, G., Liu, Y., Hou, F., 2020. Shrub coverage alters the rumen bacterial community of yaks (Bos grunniens) grazing in alpine meadows. Journal of Animal Science and Technology 62, 504-520.

Yang, G., Hu, Y., Wang, J., 2019a. Biohydrogen production from co-fermentation of fallen leaves and sewage sludge. Bioresource Technology 285, 1-8.

Yang, G., Wang, J., 2021. Biohydrogen production by co-fermentation of antibiotic fermentation residue and fallen leaves: Insights into the microbial community and functional genes. Bioresource Technology 337, 1-8.

Yang, L., Li, X., Lu, Y., Lv, Y., Chi, Y., He, Q., 2021a. Ester synthesis mechanism and activity by Bacillus licheniformis, Candida etchellsii, and Zygosaccharomyces rouxii isolated from Chinese horse bean chili paste. J Sci Food Agric 101, 5645-5651.

Yang, M., Zhao, J., Yuan, Y., Chen, X., Yang, F., Li, X., 2021b. Comparative metagenomic discovery of the dynamic cellulose-degrading process from a synergistic cellulolytic microbiota. Cellulose 28, 2105-2123.

Yang, S.Q., Xiong, H., Yang, H.Y., Yan, Q.J., Jiang, Z.Q., 2015. High-level production of beta-1,3-1,4-glucanase by Rhizomucor miehei under solid-state fermentation and its potential application in the brewing industry. J Appl Microbiol 118, 84-91.

Yang, Y., Huang, S., Zhang, Y., Xu, F., 2014. Nitrogen Removal by Chelatococcus daeguensis TAD1 and Its Denitrification Gene Identification. 172, 829-839.

Yang, Y., Li, S., Gao, Y., Chen, Y., Zhan, A., 2019b. Environment-driven geographical distribution of bacterial communities and identification of indicator taxa in Songhua River. Ecological Indicators 101, 62-70.

Yannick, P., David, G.H., 2013. The genome of the mustard leaf beetle encodes two active xylanases originally acquired from bacteria through horizontal gene transfer. Proceedings of the Royal Society B 280, 1-7.

Yim, B., Nitt, H., Wrede, A., Jacquiod, S., Sørensen, S.J., Winkelmann, T., Smalla, K., 2017. Effects of Soil Pre-Treatment with Basamid® Granules, Brassica juncea, Raphanus sativus, and Tagetes patula on Bacterial and Fungal Communities at Two Apple Replant Disease Sites. Front Microbiol 8.

Yoon, M.H., Ten, L.N., Im, W.T., 2007. Cohnella panacarvi sp. nov. a xylanolytic bacterium isolated from ginseng cultivating soil. J Microbiol Biotechnol, . 17, 913-918.

Yu, S., Zhang, G., Liu, v., Wu, P., Yu, Z., Wang, J., 2020. Repeated inoculation with fresh rumen fluid before or during weaning modulates the microbiota composition and cooccurrence of the rumen and colon of lambs. BMC Microbiology 20.

Yudai, H., Manabu, W., Kaduki, N., Akifumi, H., Hiroto, T., 2012. Application of matrix-assisted laser-desorption/ionization time-of-flight mass spectrometry for the identification of alkylphenol polyethoxylate-degrading bacteria in the environment. Journal of Pesticide Science, 305-311.

Yuwarad, P., Savitri, V., 2019. Dyadobacter and Sphingobacterium isolated from herbivore manure in Thailand and their cellulolytic activity in various organic waste substrates. Agriculture & Natural Resources 53, 89-98.

Zachary, M., 2020. The effects of captivity on the endangered comal spring riffle beetle, heterrlmis comalensis. Texas State University

Zhang, B., Qin, S., Wu, Y., Zhang, R., Xu, Y., Yang, C., 2022a. Rhamnolipids Regulate Lipid Metabolism, Immune Response, and Gut Microbiota in Rats. Frontiers in Nutrition 9.

Zhang, G., Hu, L., Wang, Y., Dong, G., Wang, Y., Luo, H., Qiu, W., Guo, G., 2019a. The Association Between Inflammaging and Age-Related Changes in the Ruminal and Fecal Microbiota Among Lactating Holstein Cows. Front Microbiol 10, 1-17.

Zhang, J., Xu, C., Huo, D., Hu, Q., Peng, Q., 2017a. Comparative study of the gut microbiome potentially related to milk protein in Murrah buffaloes (Bubalus bubalis) and Chinese Holstein cattle. Scientific report 7, 1-11.

Zhang, L., Chung, J., Jiang, Q., Sun, R., Zhang, J., Zhong, Y., Ren, N., 2017b. Characteristics of rumen microorganisms involved in anaerobic degradation of cellulose at various pH values. RSC Advances 7, 40303-40310.

Zhang, L., Fan, J., Nguyen, H.N., Li, S., Rodrigues, D.F., 2019b. Effect of cadmium on the performance of partial nitrification using sequencing batch reactor. Chemosphere 222, 913-922.

Zhang, L., Gu, J., Wang, X., Sun, W., Yin, Y., Sun, Y., Guo, A., Tuo, X., 2017c. Behavior of antibiotic resistance genes during co-composting of swine manure with Chinese medicinal herbal residues. Bioresource Technology 244, 252-260.

Zhang, L., Hao, S., Wang, Y., Lan, S., Dou, Q., Peng, Y., 2021a. Rapid start-up strategy of partial denitrification and microbially driven mechanism of nitrite accumulation mediated by dissolved organic matter. Bioresource Technology 340, 1-10.

Zhang, L., Lai, J.l., Zhang, Y., Luo, X.g., Li, Z.g., 2021b. Degradation of Uranium‑Contaminated Decontamination Film by UV Irradiation and Microbial Biodegradation. Microb Ecol, 1-12.

Zhang, Q., Li, C., Niu, X., Zhang, Z., Li, F., Li, F., 2019c. The effects of milk replacer allowance and weaning age on the performance, nutrients digestibility, and ruminal microbiota communities of lambs. Animal Feed Science and Technology 257.

Zhang, W., Zeng, Z., Liu, Z., Huang, J., Xiao, R., Shao, B., Liu, Y., Liu, Y., Tang, W., Zeng, G., Gong, J., He, Q., 2020a. Effects of carbon nanotubes on biodegradation of pollutants: Positive or negative? Ecotoxicology and Environmental Safety 189, 1-14.

Zhang, X., Ma, Z., Hao, P., Ji, S., Gao, Y., 2022b. Revealing the characteristics, bacterial communities and antibiotic-resistance genes in bioaerosols inside different animal houses: From one-health perspective. Available at SSRN: <https://ssrn.com/abstract=4084669> or <http://dx.doi.org/10.2139/ssrn.4084669>.

Zhang, X., Qu, Y., Ma, Q., Zhang, Z., Li, D., Wang, J., Shen, W., Shen, E., Zhou, J., 2015. Illumina MiSeq Sequencing Reveals Diverse Microbial Communities of Activated Sludge Systems Stimulated by Different Aromatics for Indigo Biosynthesis from Indole. PLOS ONE 10, e0125732.

Zhang, X., Zhang, Q., Liang, B., Li, J., 2017d. Changes in the abundance and structure of bacterial communities in the greenhouse tomato cultivation system under long-term fertilization treatments. Applied Soil Ecology () 121 82-89.

Zhang, Y., Xu, J., Wang, E., Wang, N., 2020b. Mechanisms Underlying the Rhizosphere-To-Rhizoplane Enrichment of Cellvibrio Unveiled by Genome-Centric Metagenomics and Metatranscriptomics. Microorganisms 8, 1-14.

Zhao, C., Liu, B., Meng, S., Wang, Y., Yan, L., Zhang, X., Wei, D., 2021a. Microbial fuel cell enhanced pollutants removal in a solid-phase biological denitrification reactor: System performance, bioelectricity generation and microbial community analysis. Bioresource Technology 341, 1-8.

Zhao, D., Gao, P., Xu, L., Qu, L., Han, Y., Zheng, L., Gong, X., 2021b. Disproportionate responses between free-living and particle-attached bacteria during the transition to oxygen-deficient zones in the Bohai Seawater. Science of The Total Environment 791, 1-13.

Zhao, R., Feng, J., Liu, J., Fu, W., Li, X., Li, B., 2019. Deciphering of microbial community and antibiotic resistance genes in activated sludge reactors under high selective pressure of different antibiotics. Water Research 151 388-402.

Zhao, R., Yu, K., Zhang, J., Zhang, G., Huang, J., Ma, L., Deng, C., Li, X., Li, B., 2020a. Deciphering the mobility and bacterial hosts of antibiotic resistance genes under antibiotic selection pressure by metagenomic assembly and binning approaches. Water Research 186, 1-15.

Zhao, Y., Mao, X., Zhang, M., Yang, W., Di, H.J., Ma, L., Liu, W., Li, B., 2020b. Response of soil microbial communities to continuously mono-cropped cucumber under greenhouse conditions in a calcareous soil of north China. Journal of Soils and Sediments 20, 2446-2459.

Zheng, Y., He, T., Xie, T., Wang, J., Yang, Z., Sun, X., Wang, W., Li, S., 2022. Hydroxy-selenomethionine supplementation promotes the in vitro rumen fermentation of dairy cows by altering the relative abundance of rumen microorganisms. J Appl Microbiol 132, 2583-2593.

Zheng, Y., Liu, Y., Qu, M., Hao, M., Yang, D., Yang, Q., Wang, X.C., Dzakpasu, M., 2021. Fate of an antibiotic and its effects on nitrogen transformation functional bacteria in integrated vertical flow constructed wetlands. Chemical Engineering Journal 417, 1-10.

Zhong, F., Fan, X., Ji, W., Hai, Z., Hu, N., Li, X., Liu, G., Yu, C., Chen, Y., Lian, B., Wei, H., Zhang, J., 2022. Soil Fungal Community Composition and Diversity of Culturable Endophytic Fungi from Plant Roots in the Reclaimed Area of the Eastern Coast of China. Journal of Fungi 8, 1-17.

Zhong, S., Chen, Q., Hu, J., Liu, S., Qiao, S., Ni, J., Sun, W., 2020a. Vertical distribution of microbial communities and their response to metal(loid)s along the vadose zone–aquifer sediments. Journal of Applied Microbiology 129, 1657-1673.

Zhong, X.-Z., Li, X.-X., Zeng, Y., Wang, S.-P., Sun, Z.-Y., Tang, Y.-Q., 2020b. Dynamic change of bacterial community during dairy manure composting process revealed by high-throughput sequencing and advanced bioinformatics tools. Bioresource Technology 306, 1-10.

Zhou, J., Chen, J., Ma, J., Xu, N., Xin, F., Zhang, W., Zhang, H., Dong, W., Jiang, M., 2021a. Luteimonas wenzhouensis Sp. Nov., A Chitinolytic Bacterium Isolated from a Landfill Soil. Current Microbiology 78, 383 - 388.

Zhou, P., Zhang, G., Chen, S., Jiang, Z., Tang, Y., Henrissat, B., Yan, Q., Yang, S., Chen, C.-F., Zhang, B., Du, Z., 2014. Genome sequence and transcriptome analyses of the thermophilic zygomycete fungus Rhizomucor miehei. BMC Genomics 15, 1-13.

Zhou, S., Song, Z., Sun, Z., Shi, X., Zhang, Z., 2021b. The effects of undulating seasonal temperature on the performance and microbial community characteristics of simultaneous anammox and denitrification (SAD) process. Bioresource Technology 321, 1-9.

Zhu, Y., Wang, Z., Hu, R., Wang, X., Wang, L., 2021. Comparative study of the bacterial communities throughout the gastrointestinal tract in two beef cattle breeds. Appl Microbiol Biotechnol 105, 313-325.
