# Supplementary figures and images for: Herbivore camping reshapes the taxonomy, function and network of pasture soil microbial communities
Source: PeerJ. 2022 Nov 9;10:e14314. doi: 10.7717/peerj.14314 (PMC9653066; doi:10.7717/peerj.14314)

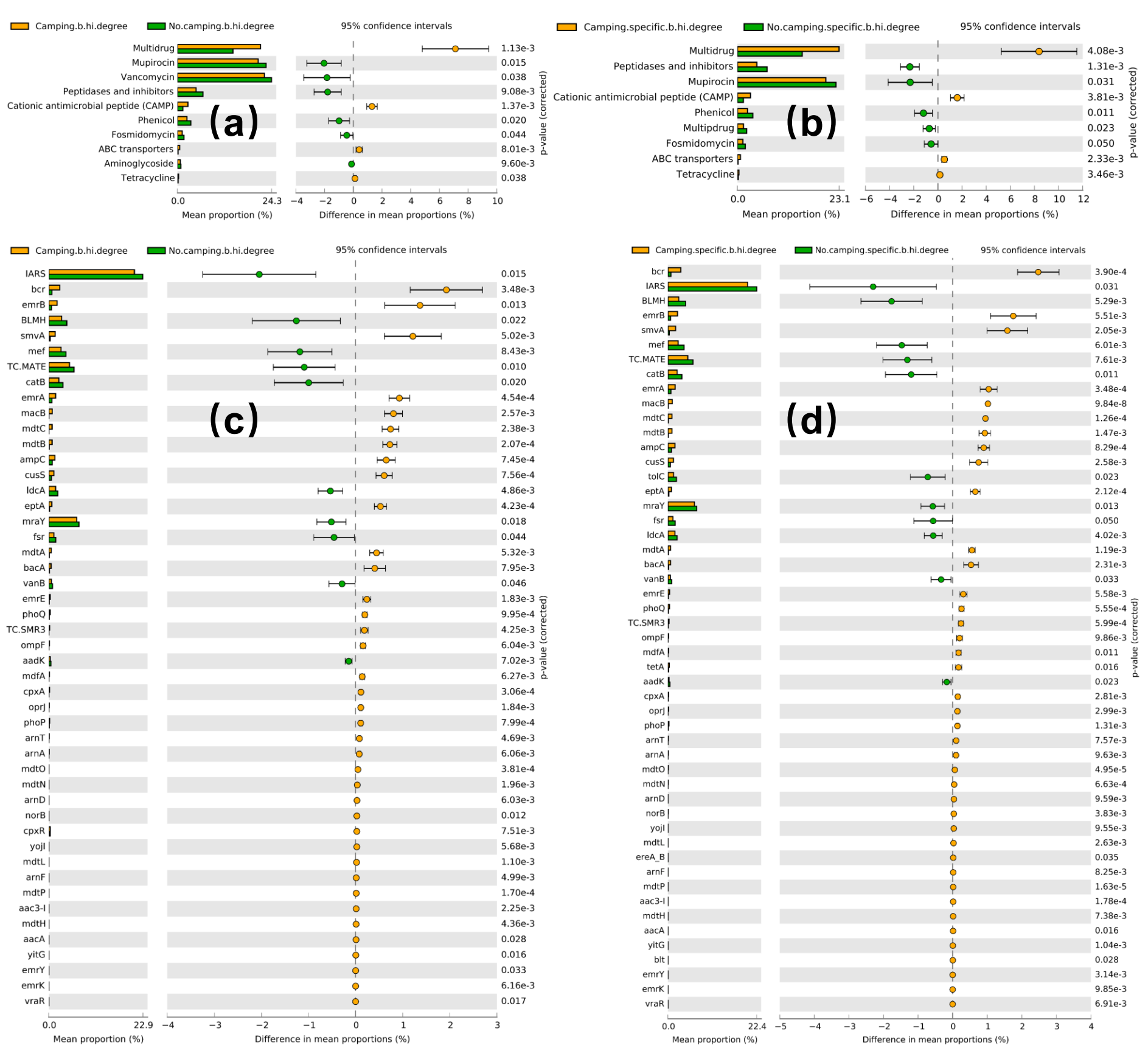

Supplement: Supplemental Information 1 [file peerj-10-14314-s001.zip › Figure S100.pdf]

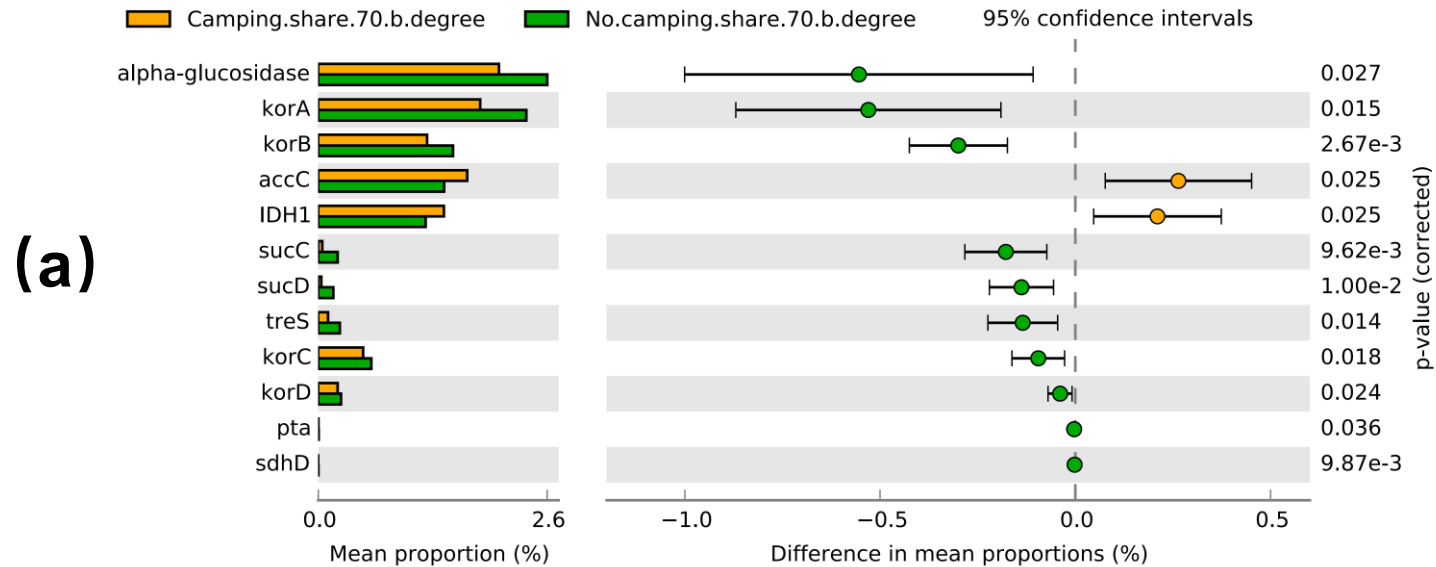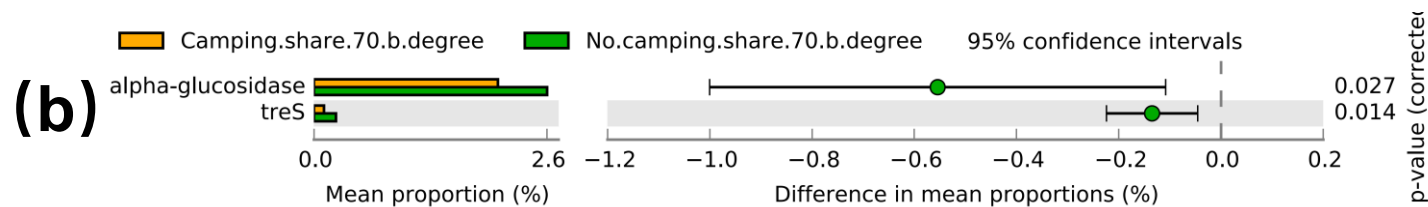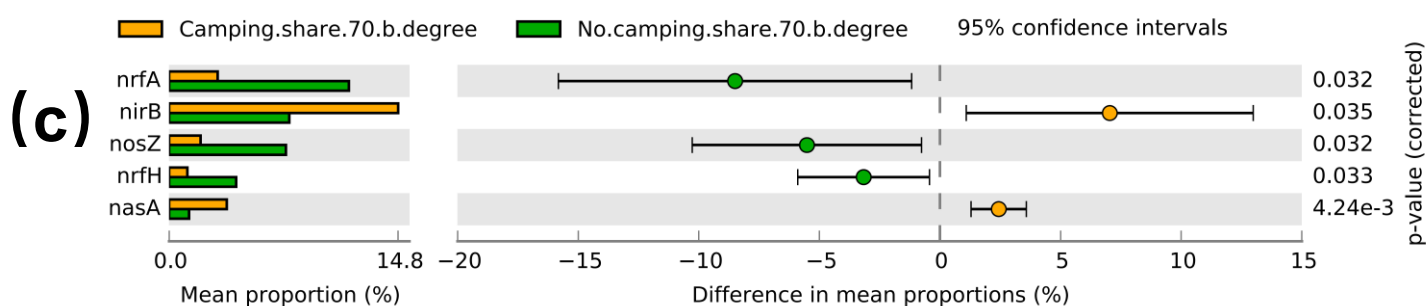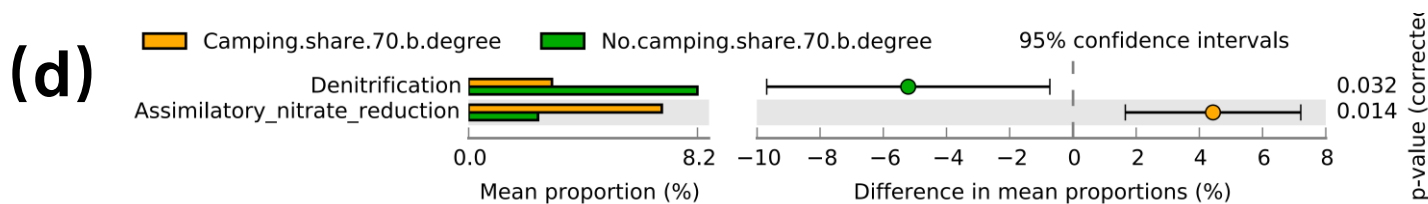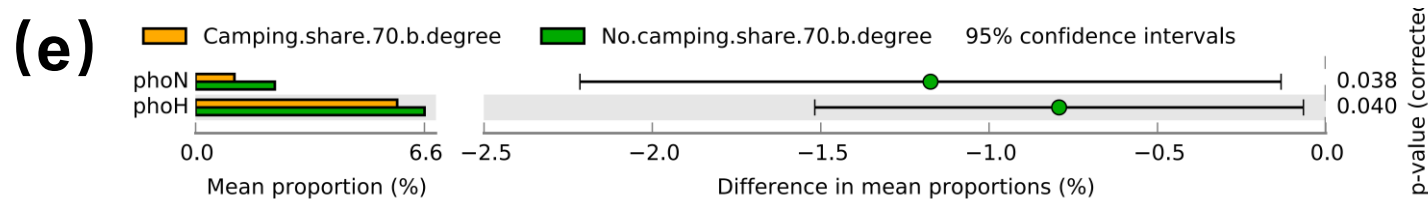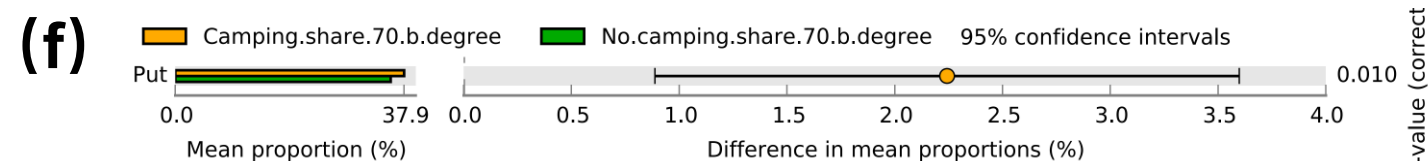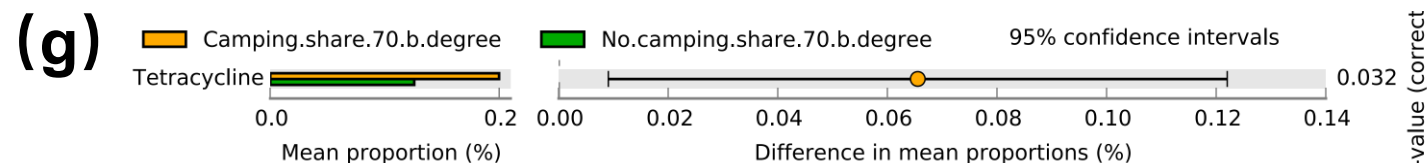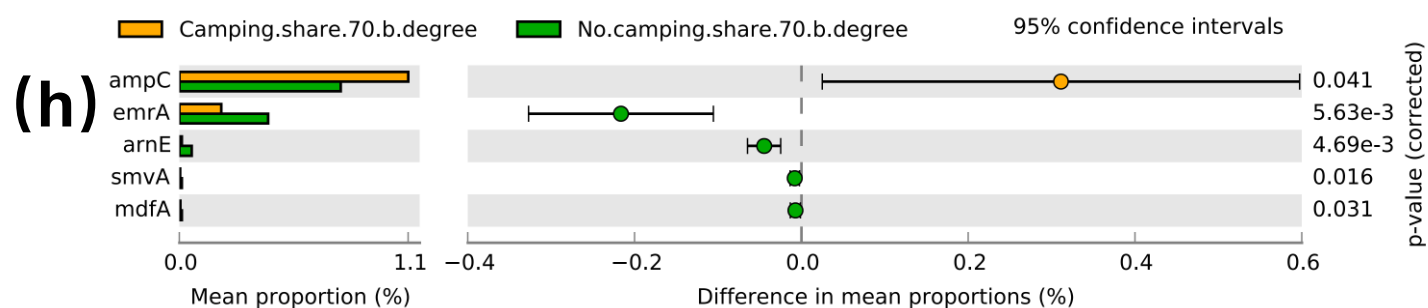

Supplement: Supplemental Information 1 [file peerj-10-14314-s001.zip › Figure S101.pdf]

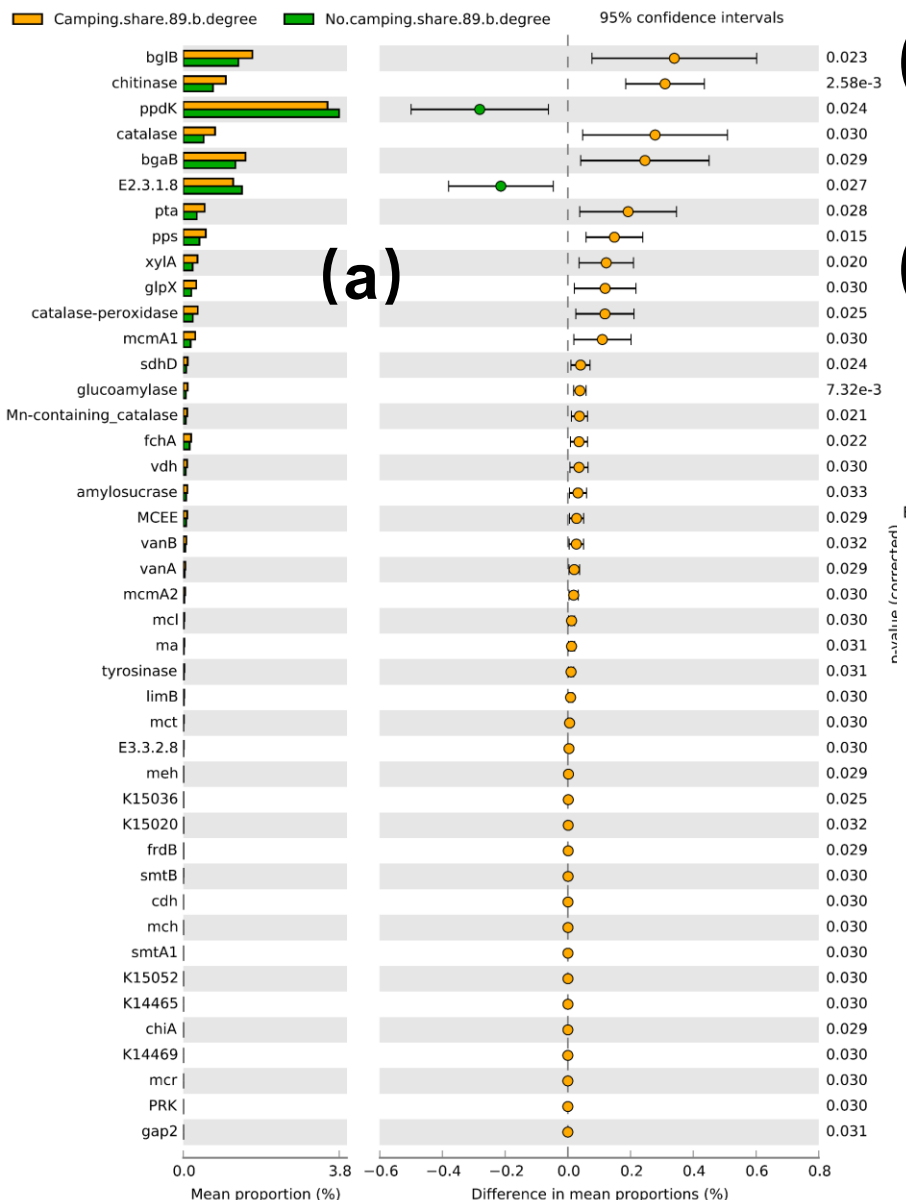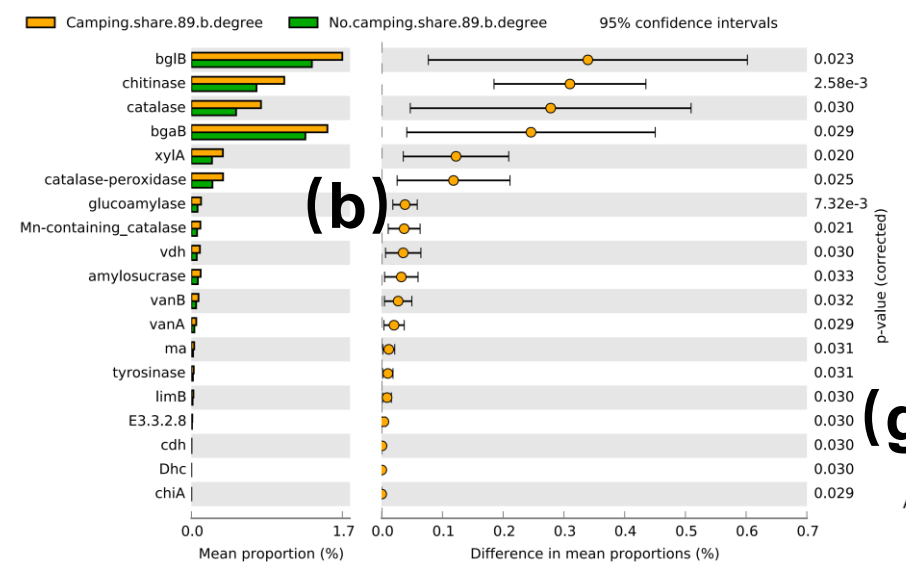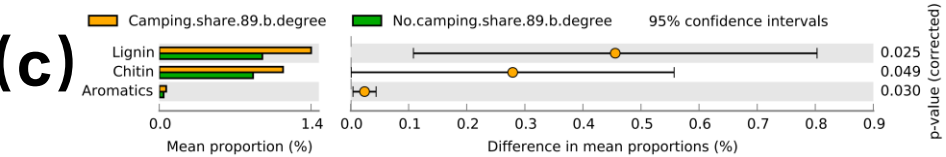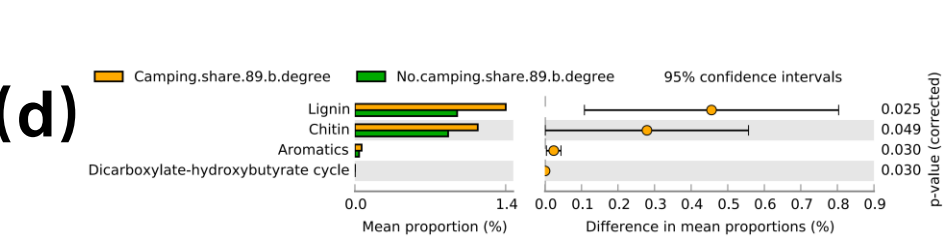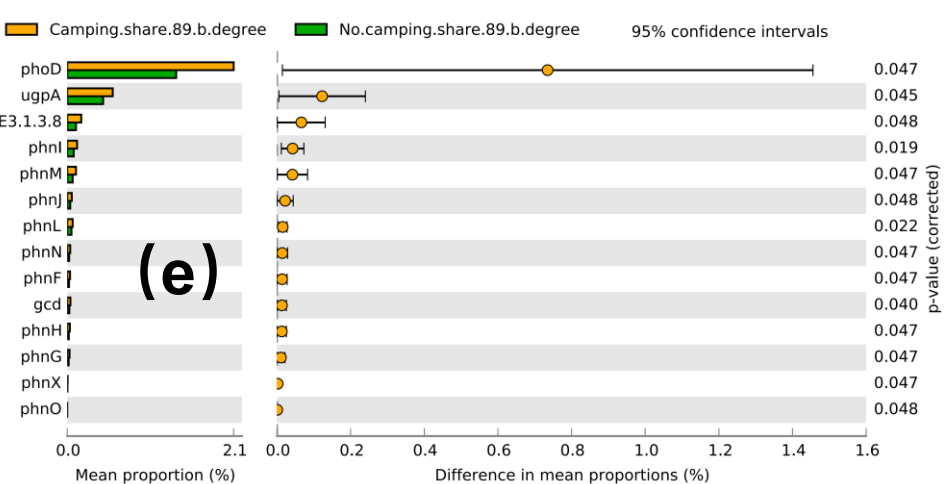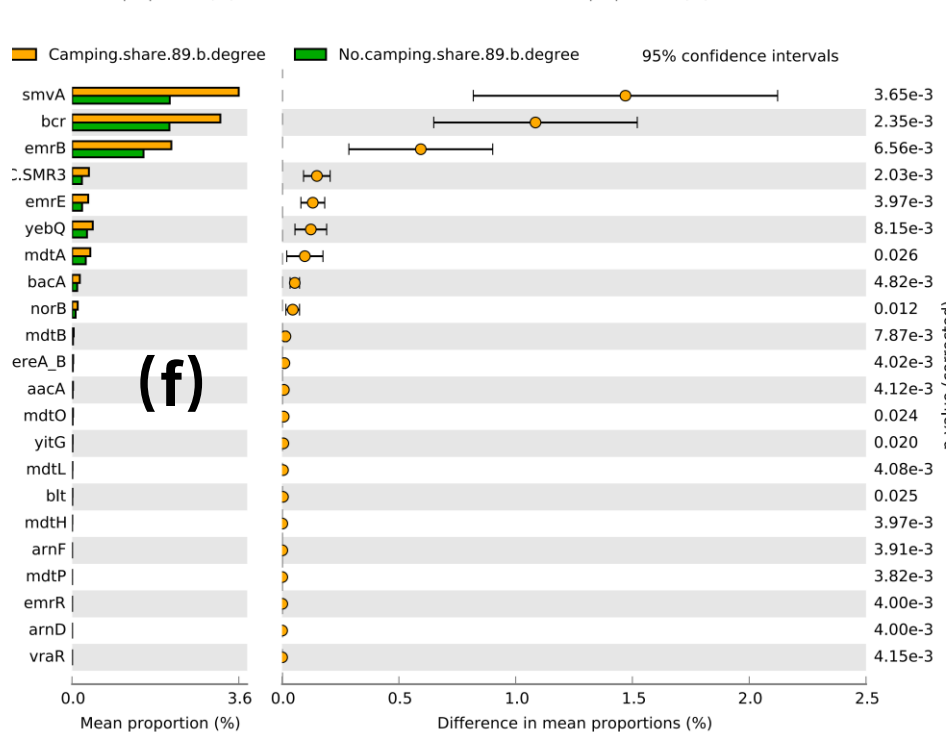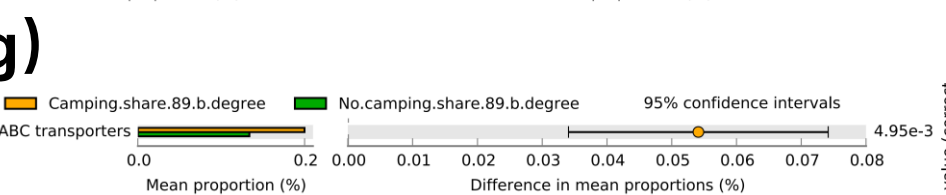

Supplement: Supplemental Information 1 [file peerj-10-14314-s001.zip › Figure S102.pdf]

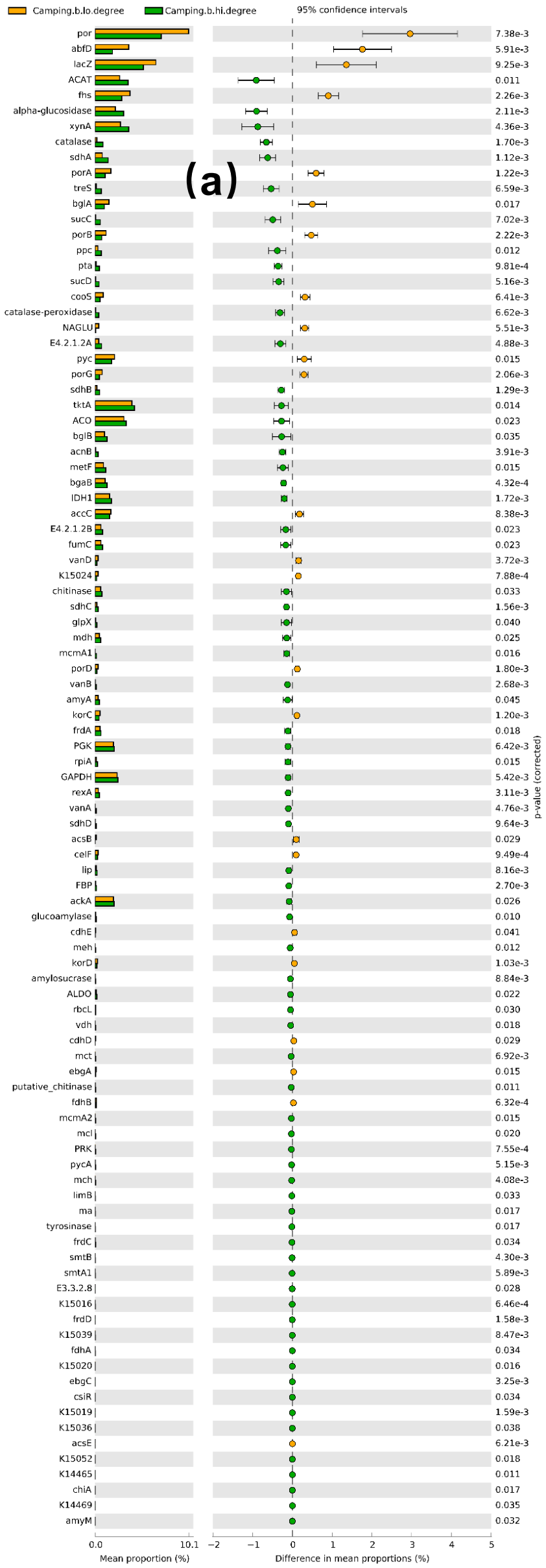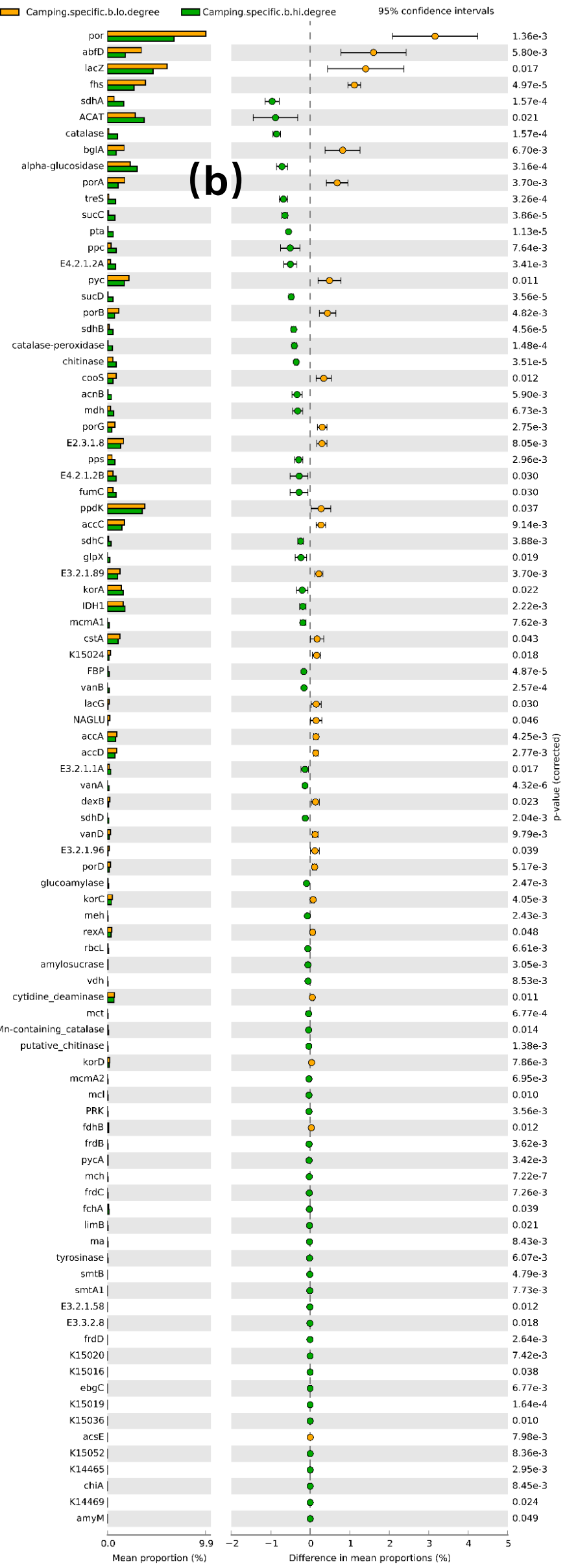

Supplement: Supplemental Information 1 [file peerj-10-14314-s001.zip › Figure S103.pdf]

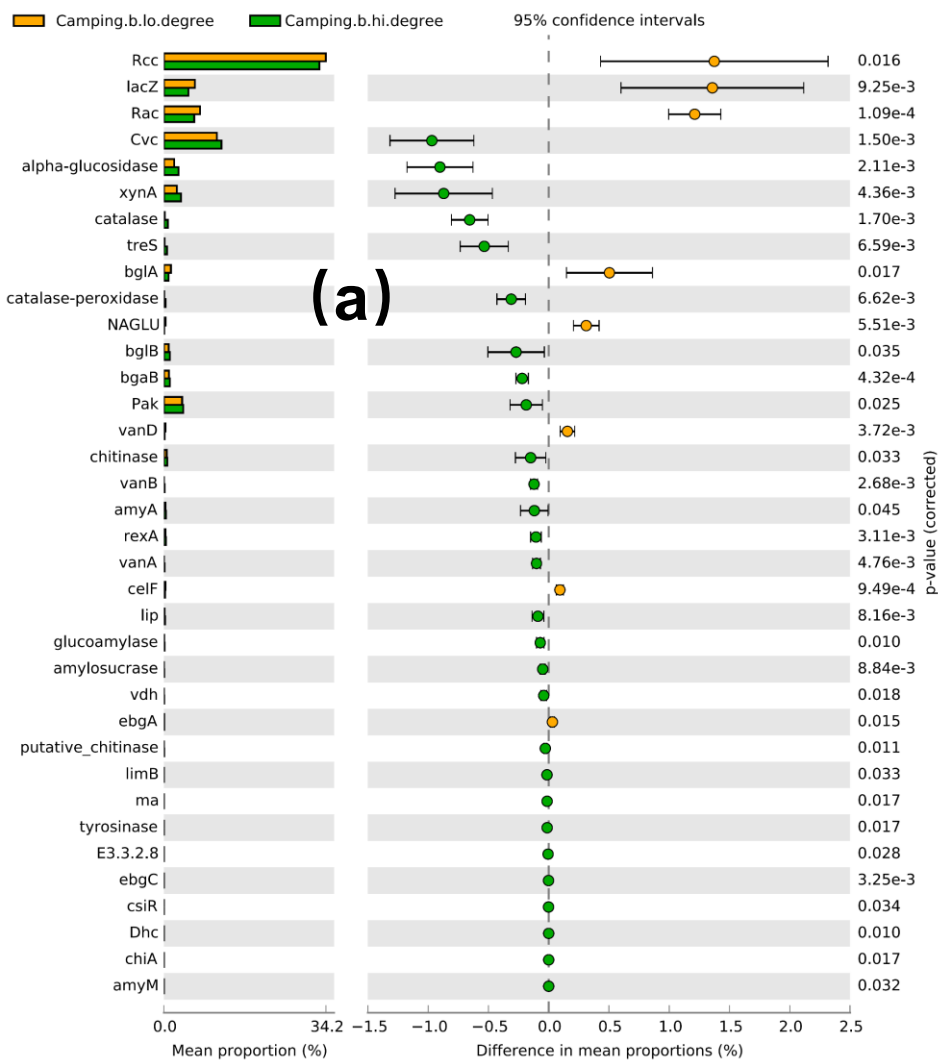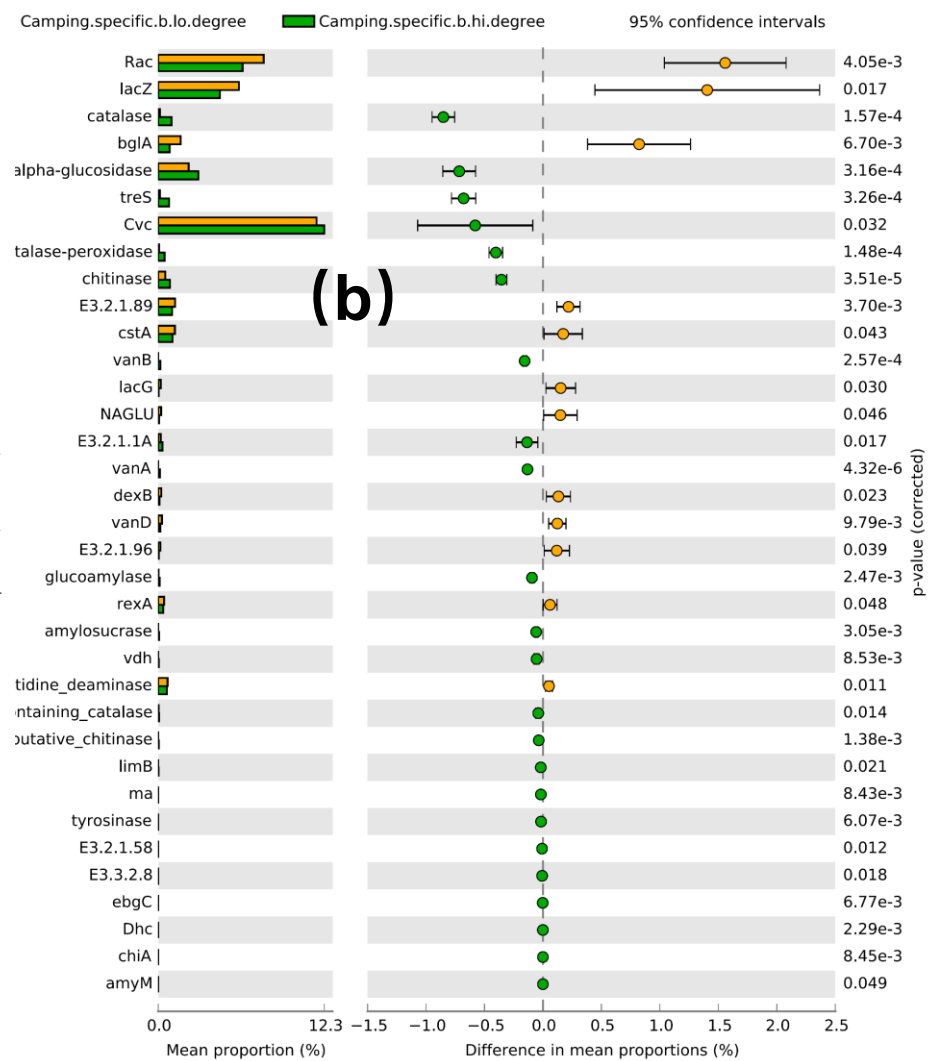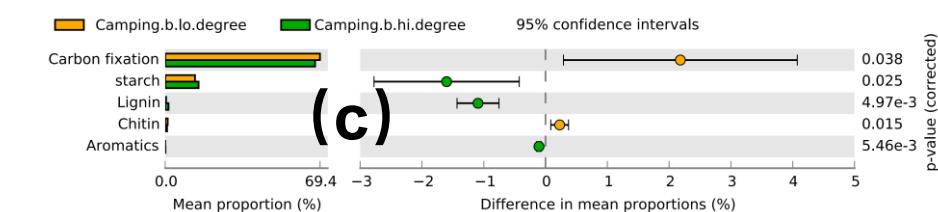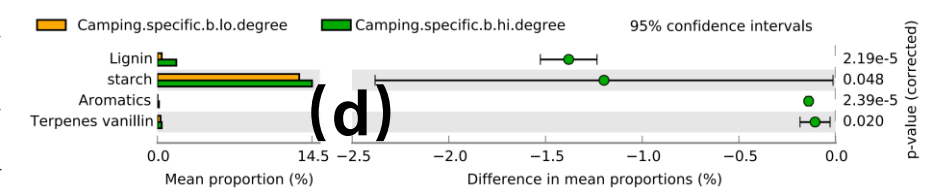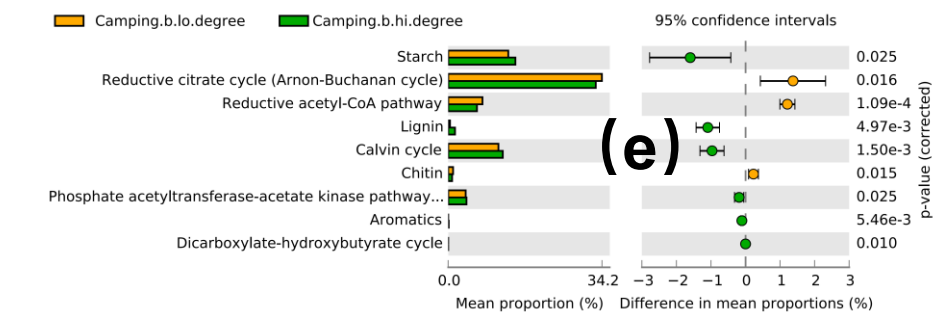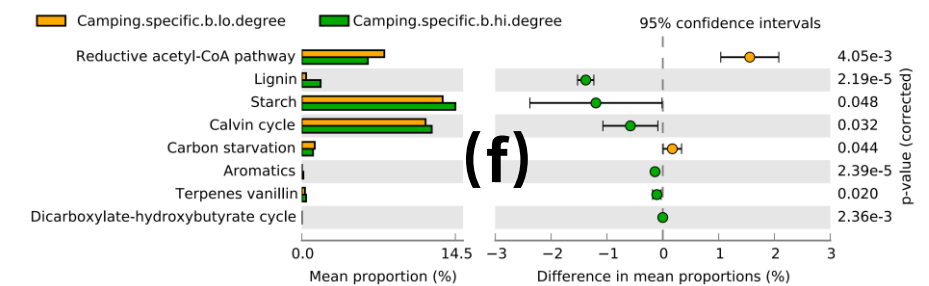

Supplement: Supplemental Information 1 [file peerj-10-14314-s001.zip › Figure S104.pdf]

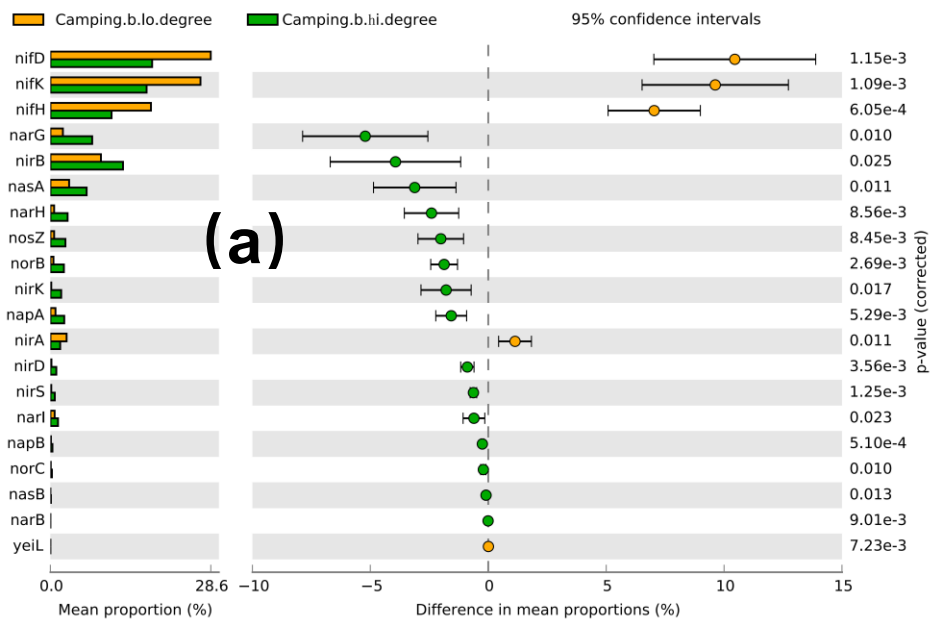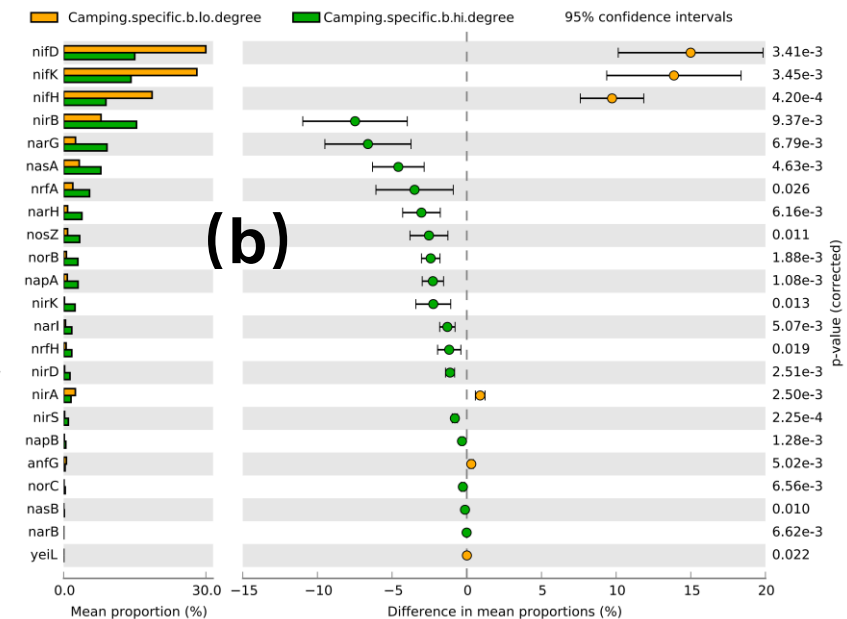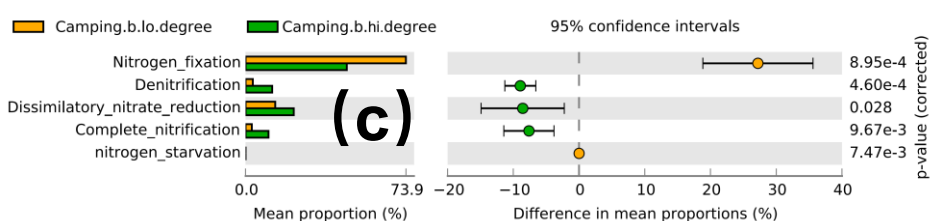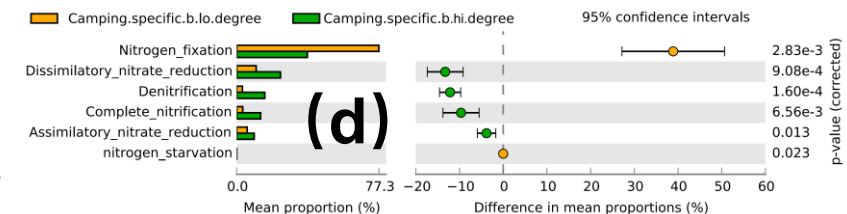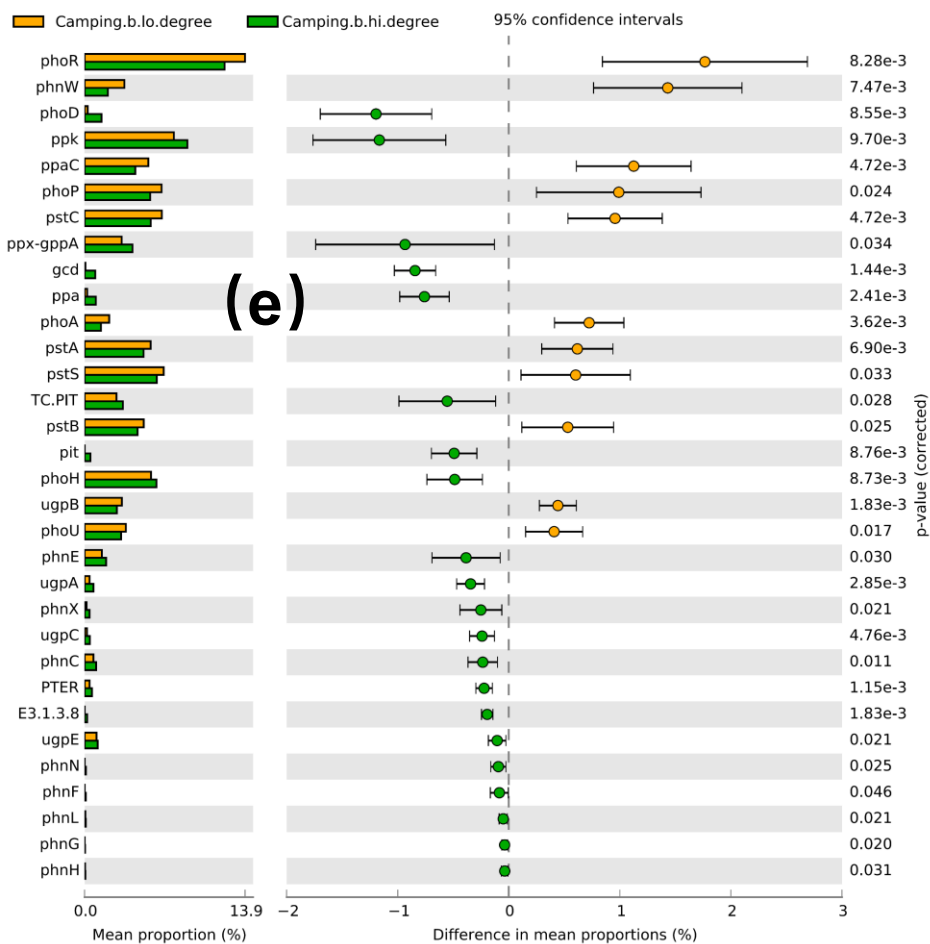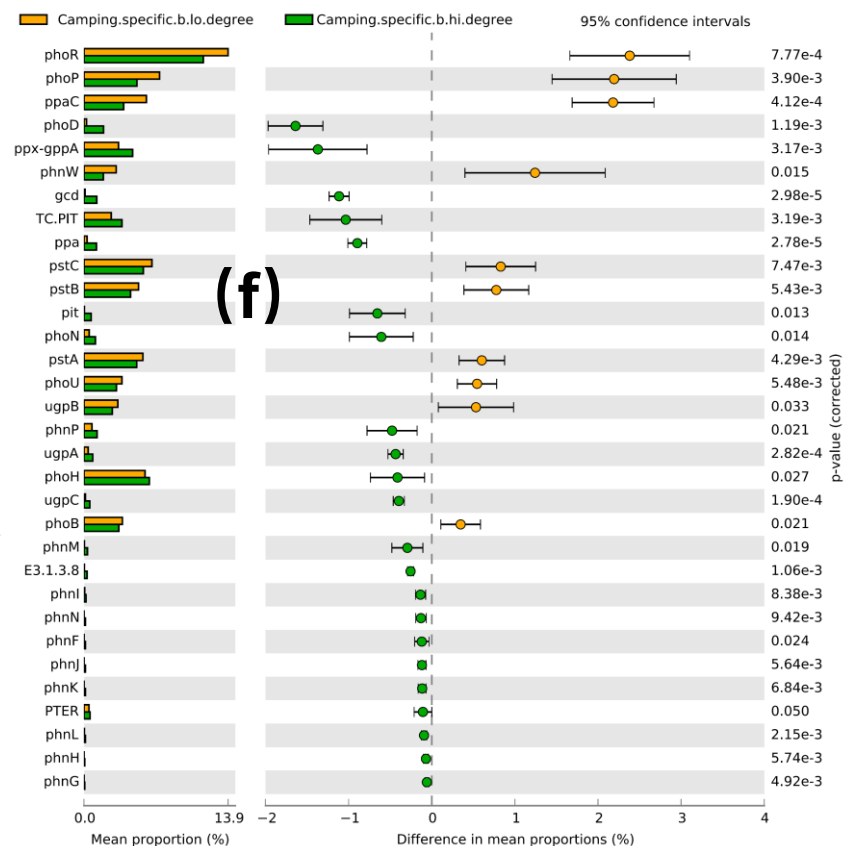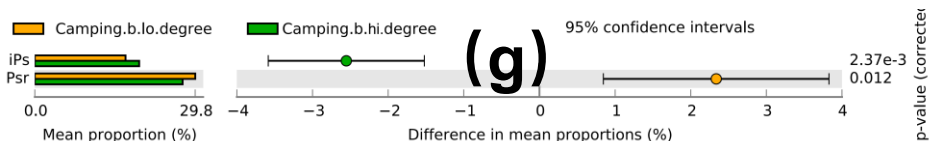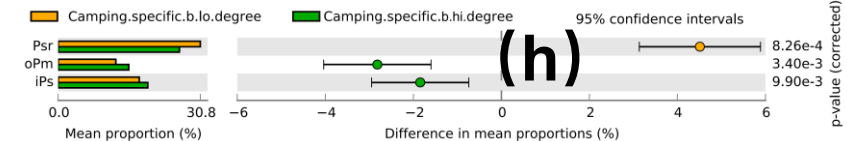

Supplement: Supplemental Information 1 [file peerj-10-14314-s001.zip › Figure S105.pdf]

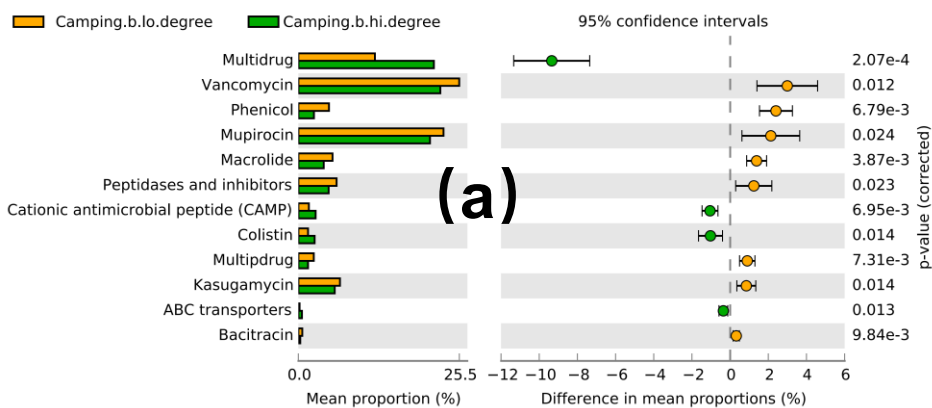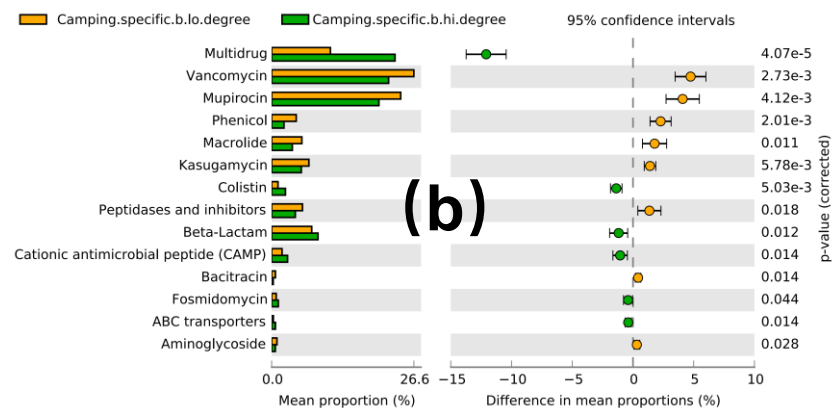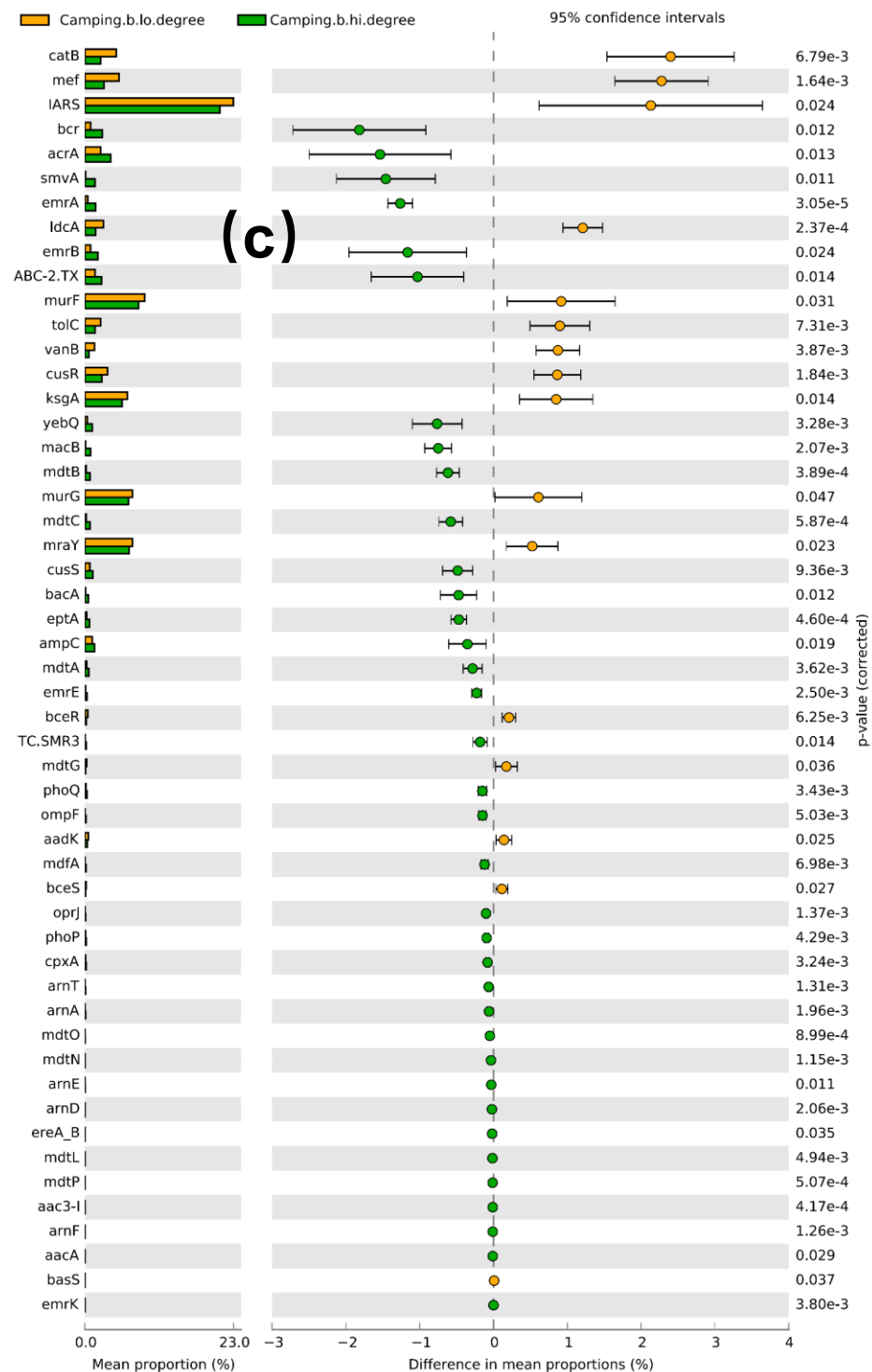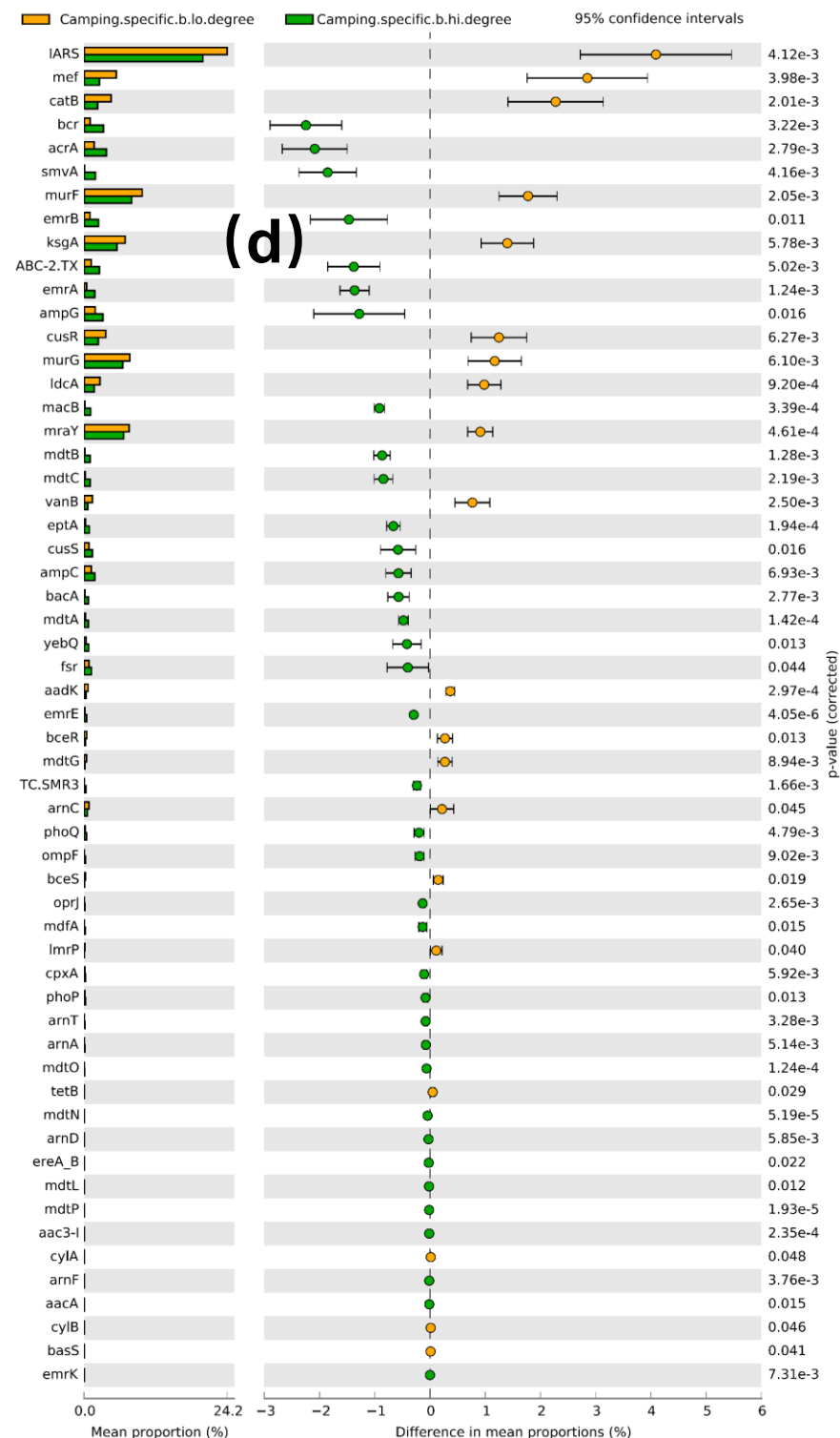

Supplement: Supplemental Information 1 [file peerj-10-14314-s001.zip › Figure S106.pdf]

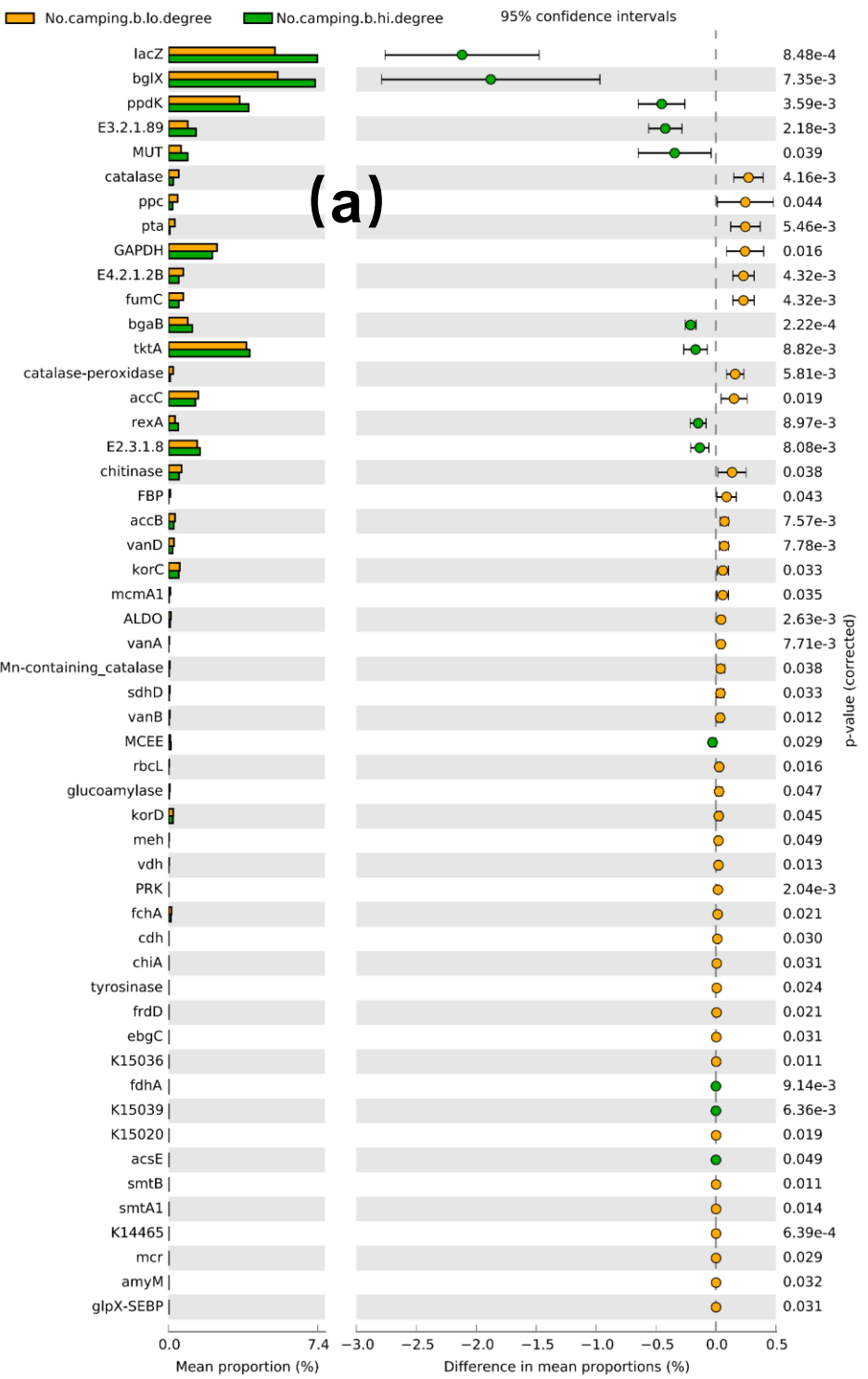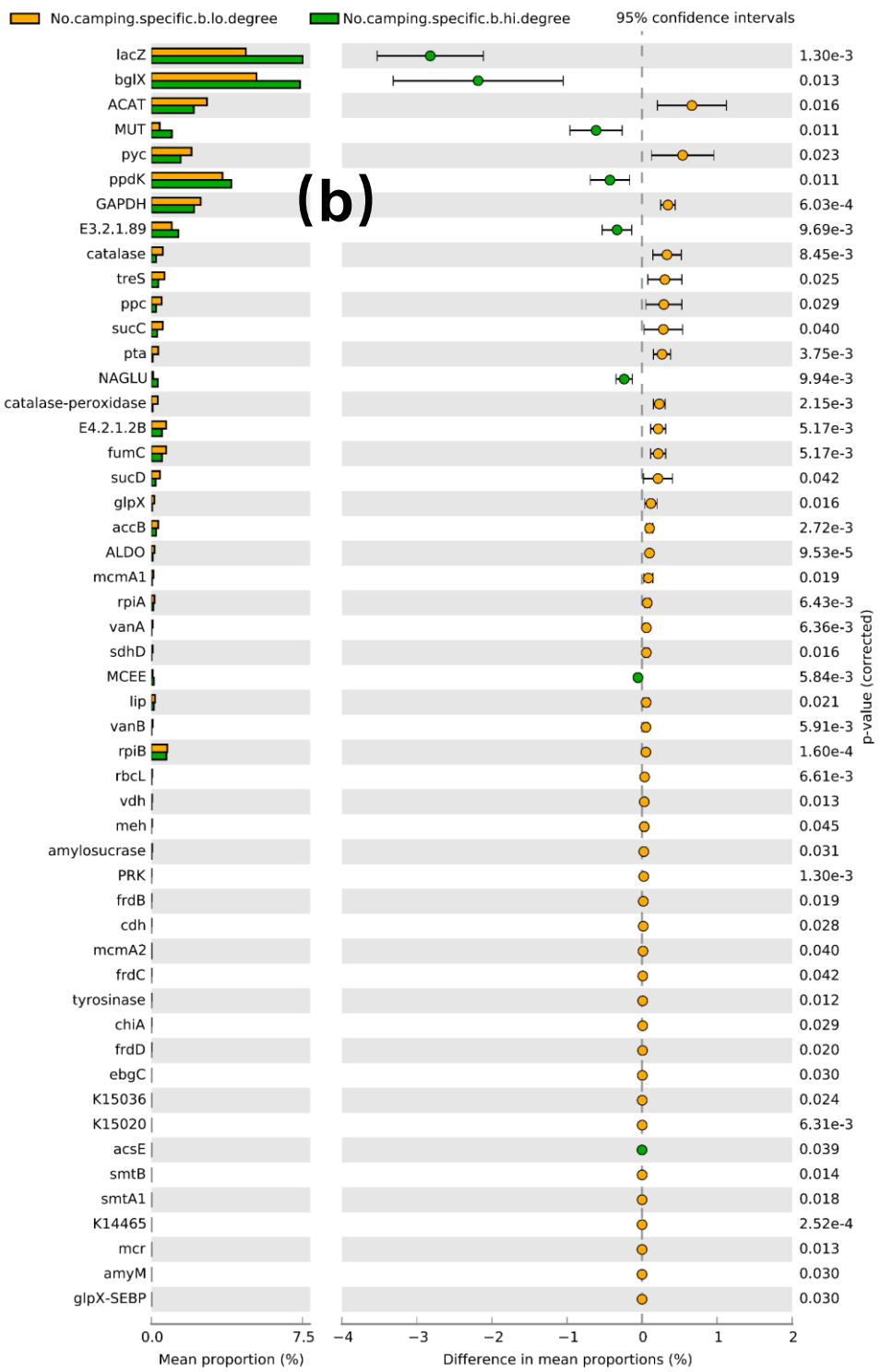

Supplement: Supplemental Information 1 [file peerj-10-14314-s001.zip › Figure S107.pdf]

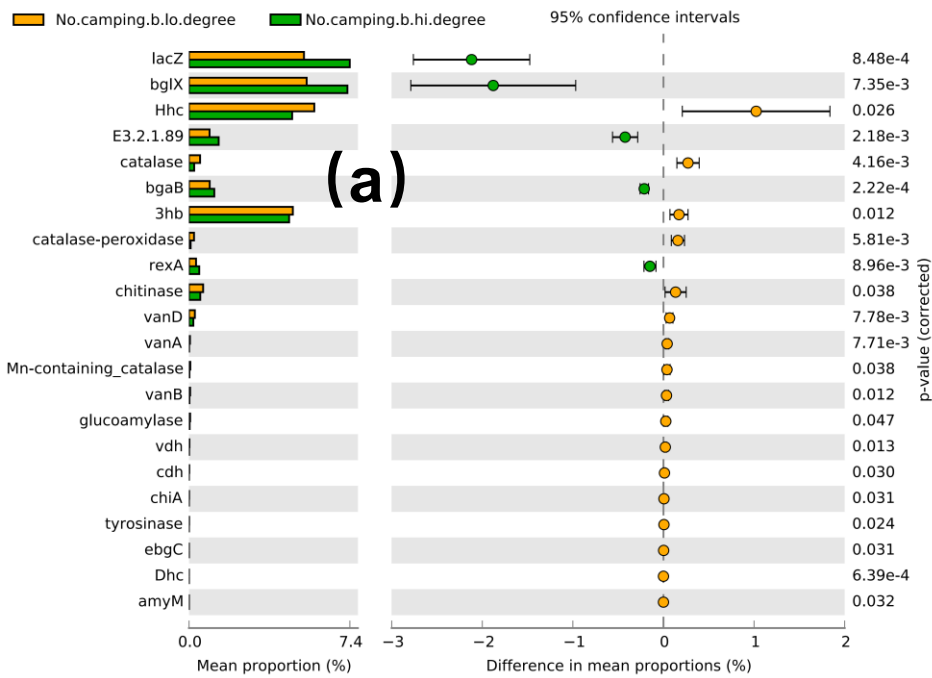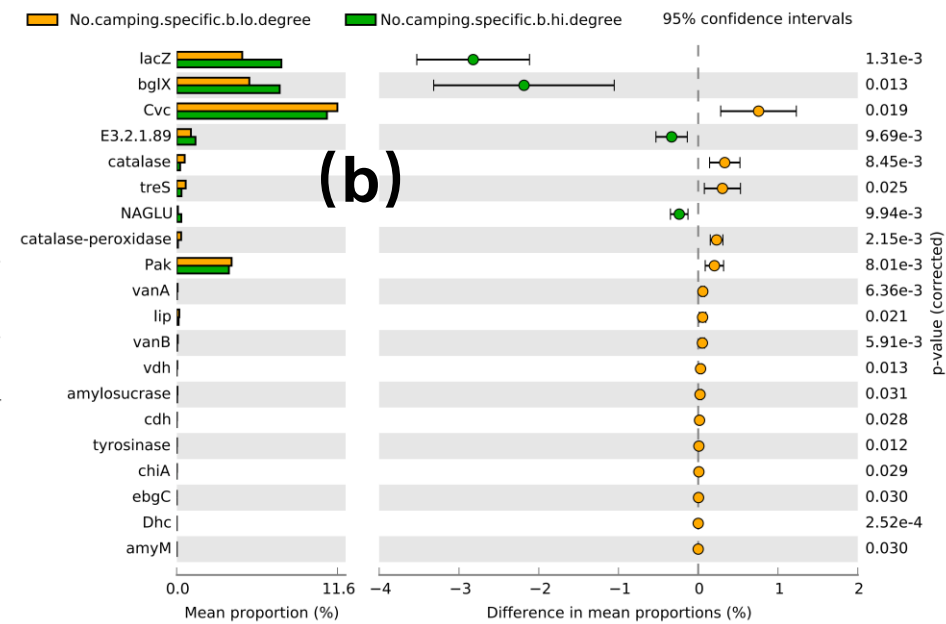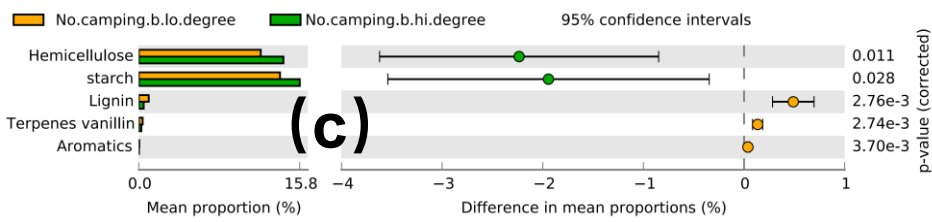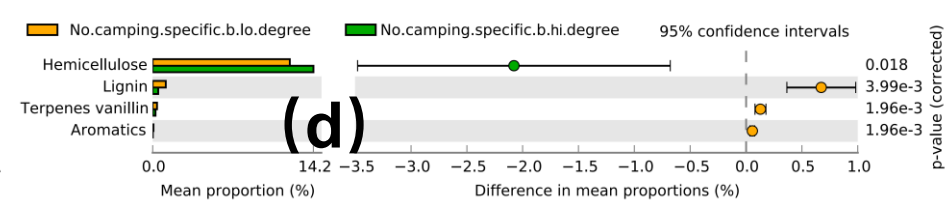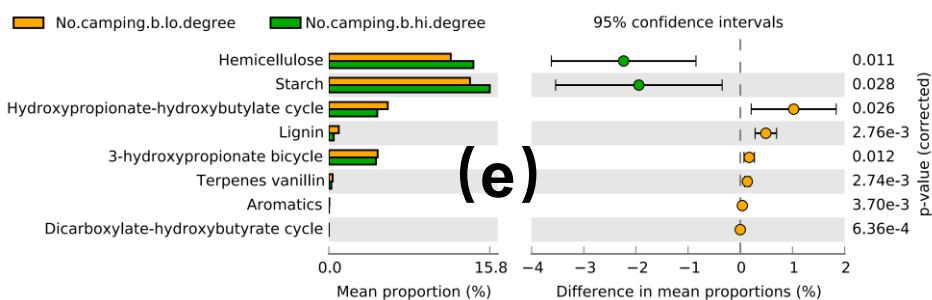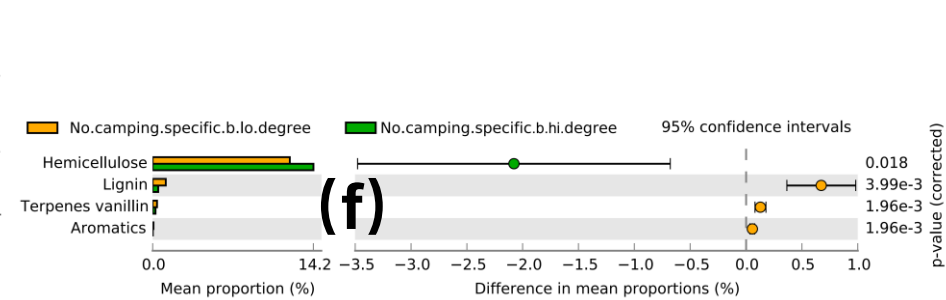

Supplement: Supplemental Information 1 [file peerj-10-14314-s001.zip › Figure S108.pdf]

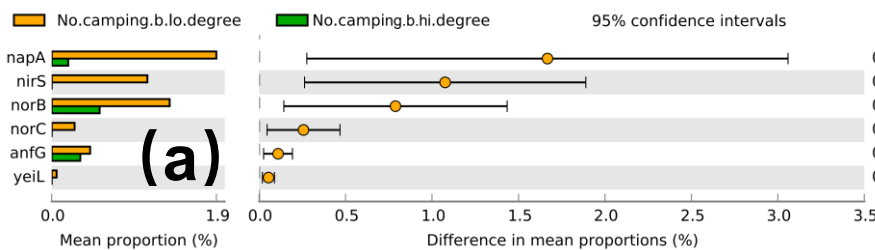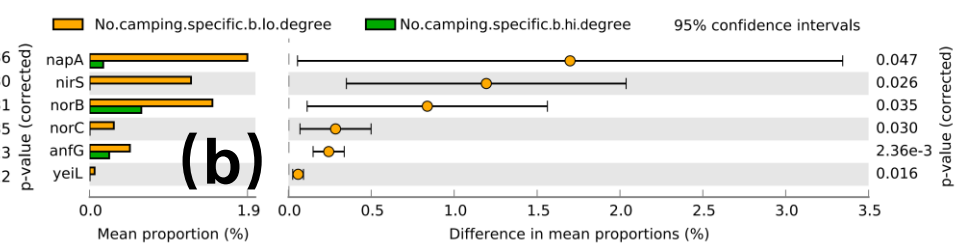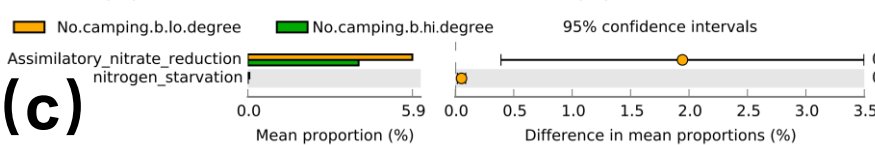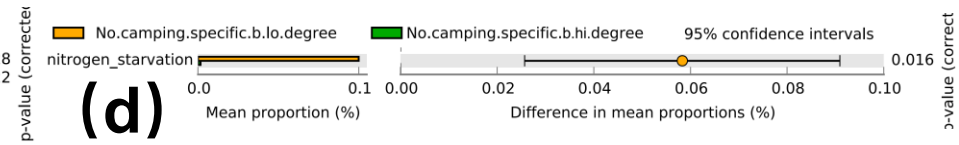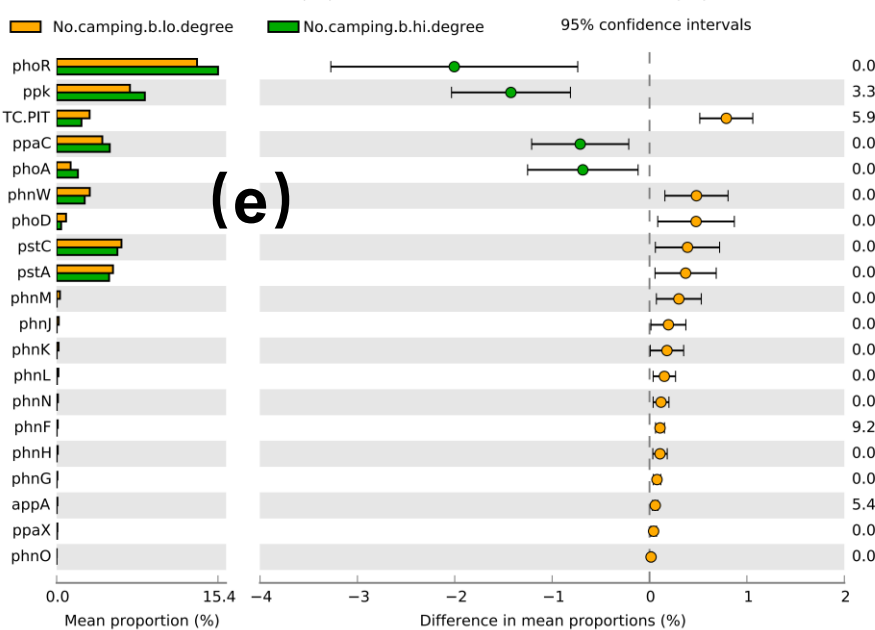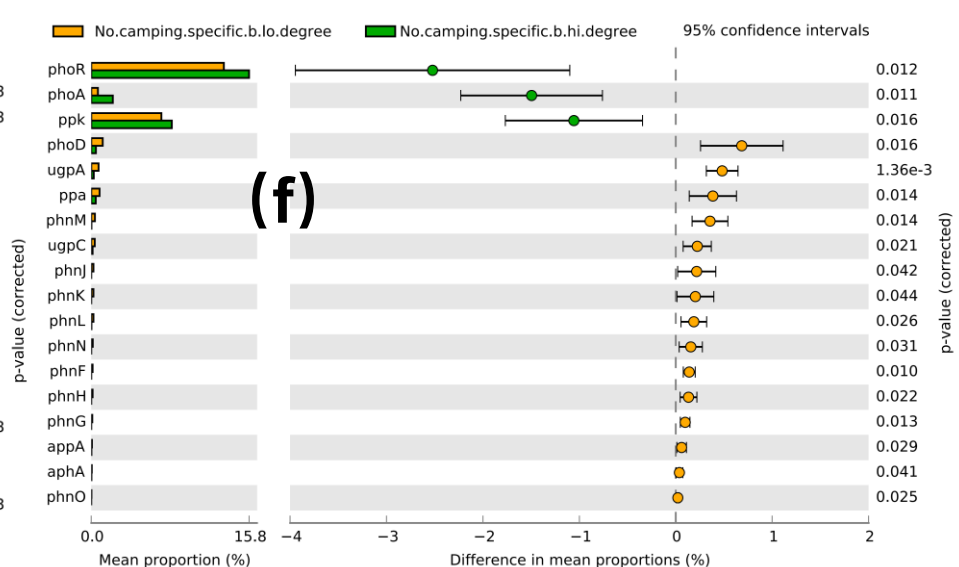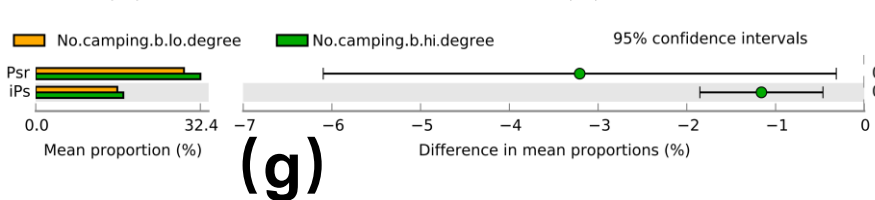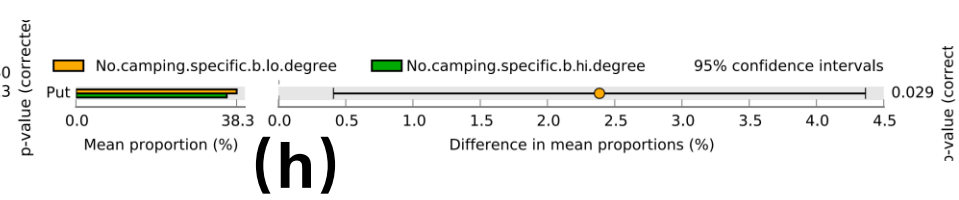

Supplement: Supplemental Information 1 [file peerj-10-14314-s001.zip › Figure S109.pdf]

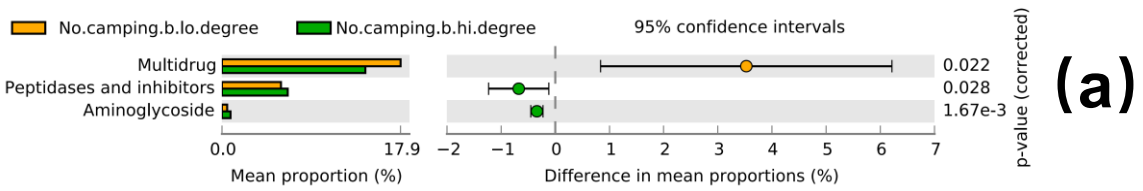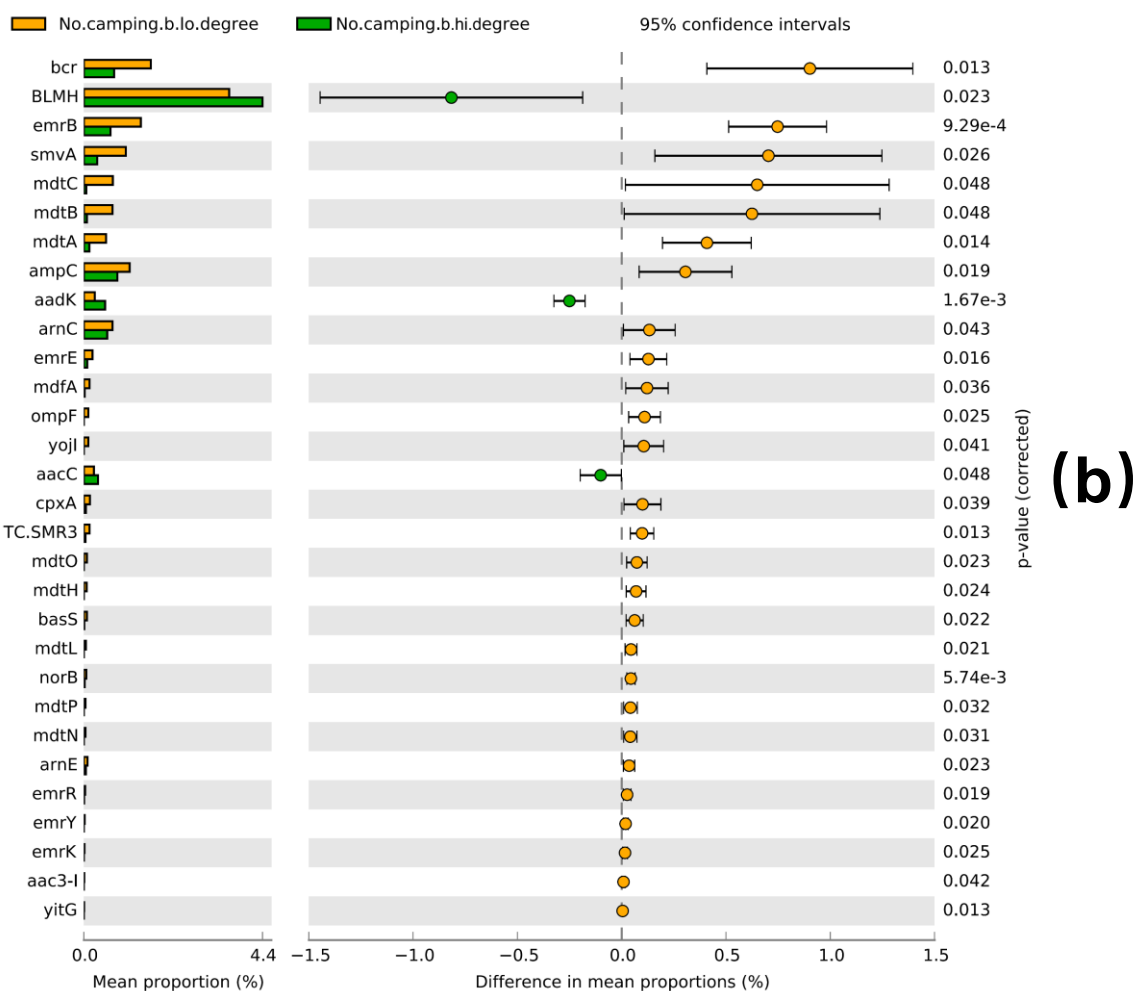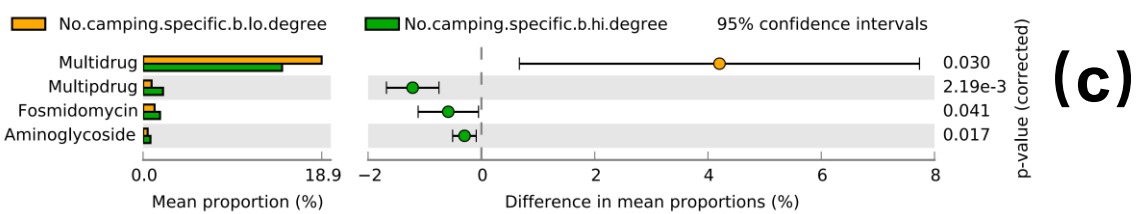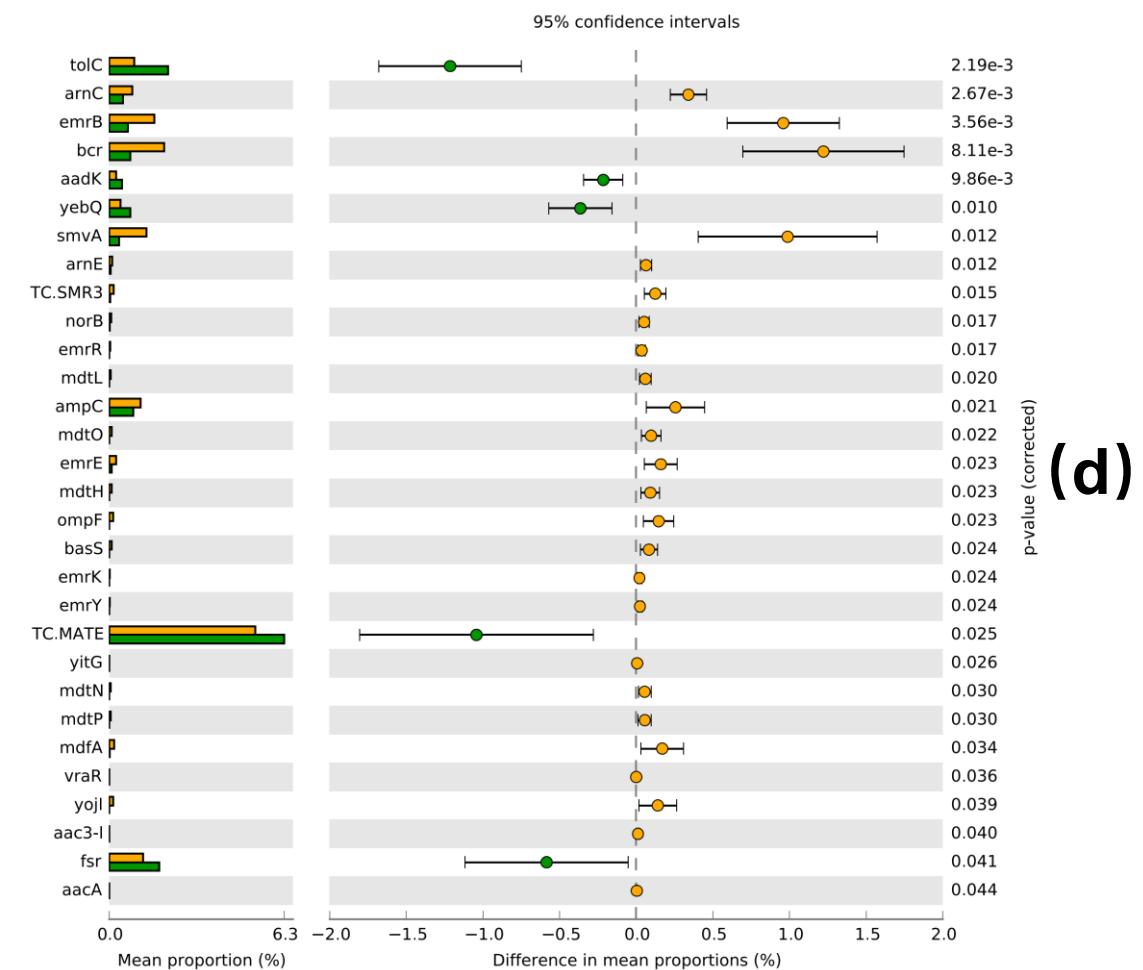

Supplement: Supplemental Information 1 [file peerj-10-14314-s001.zip › Figure S110.pdf]

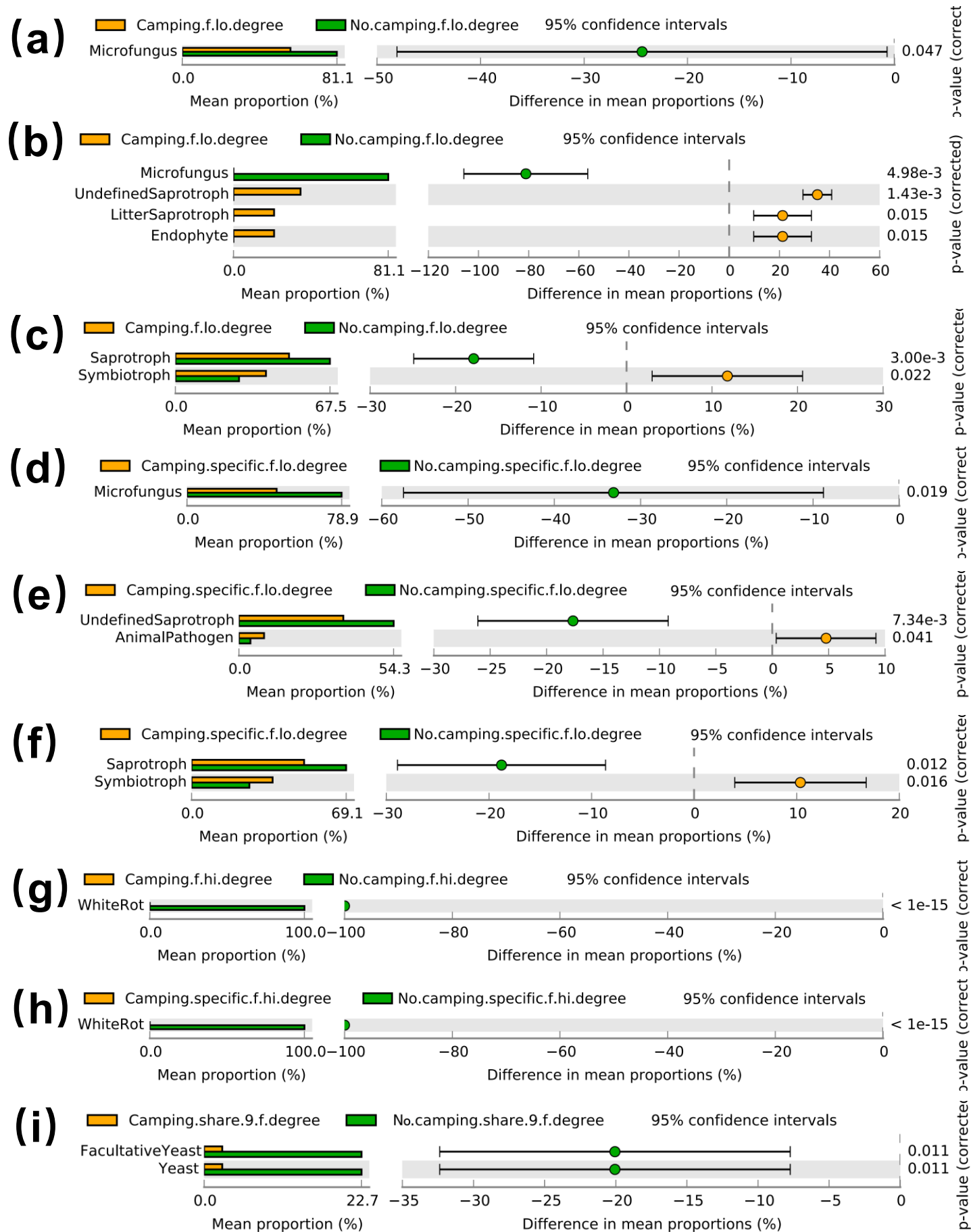

Supplement: Supplemental Information 1 [file peerj-10-14314-s001.zip › Figure S111.pdf]

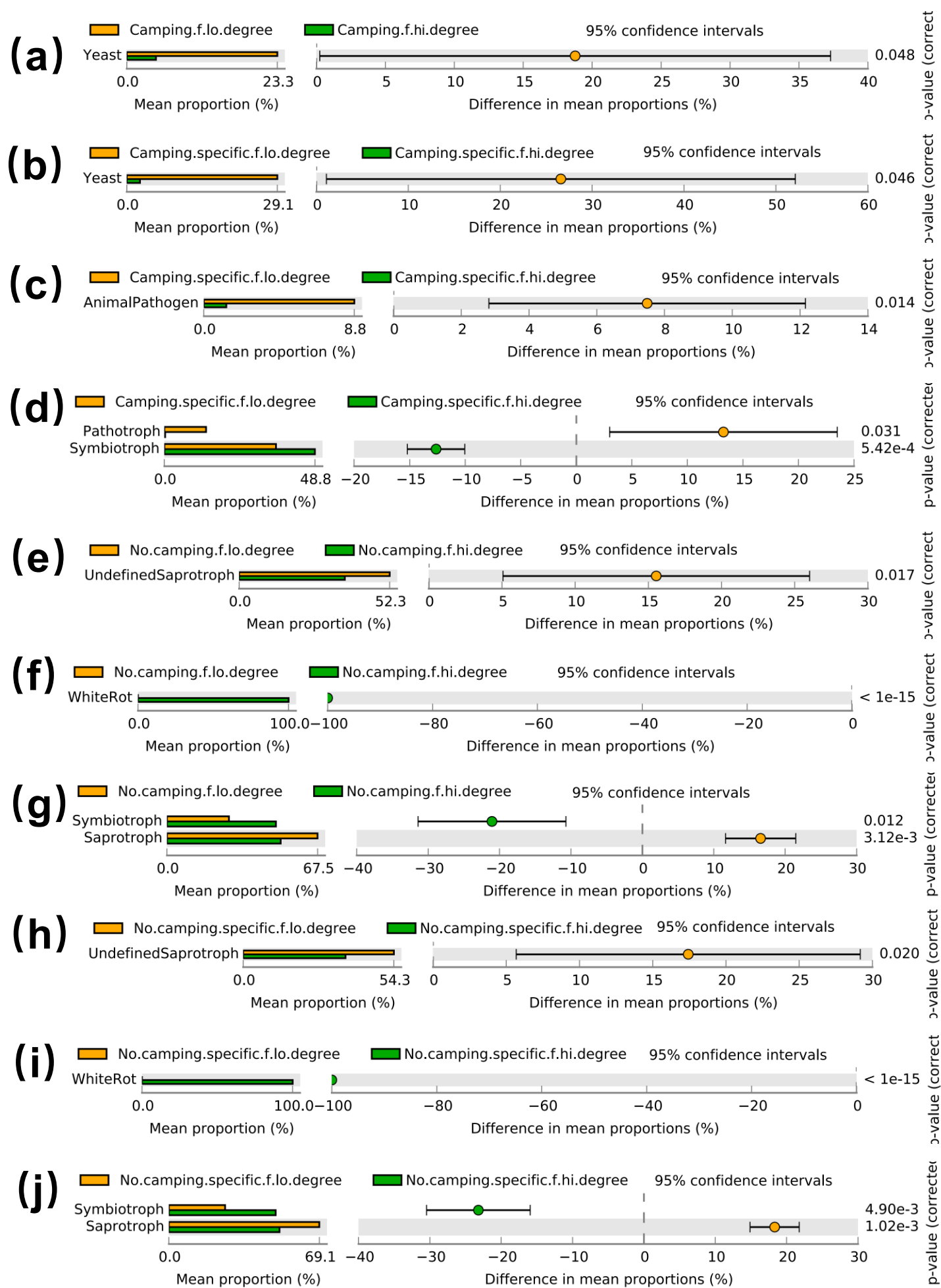

Supplement: Supplemental Information 1 [file peerj-10-14314-s001.zip › Figure S112.pdf]

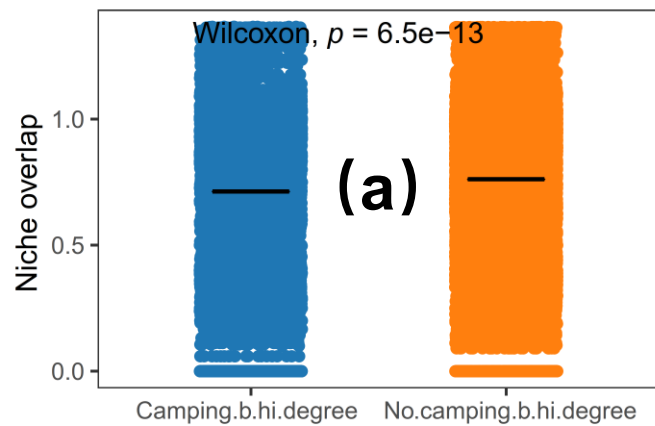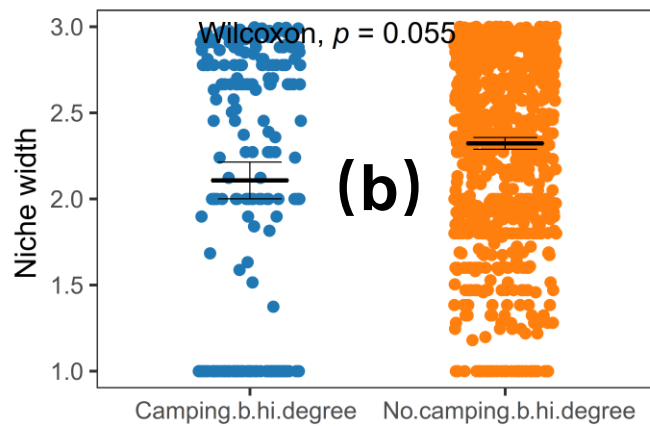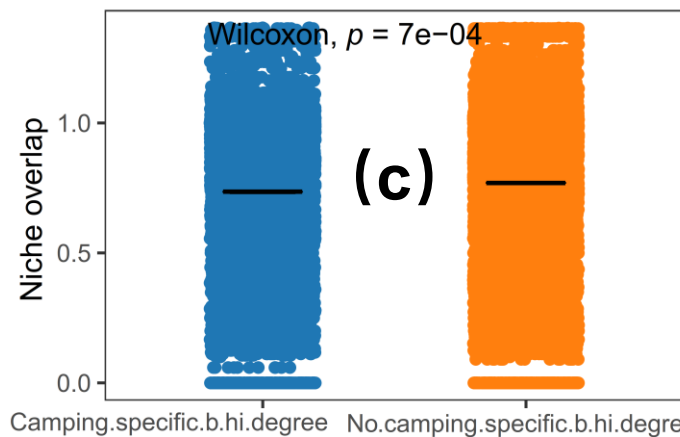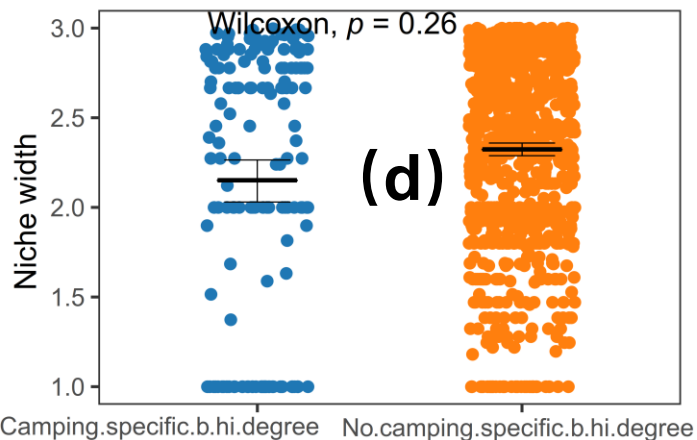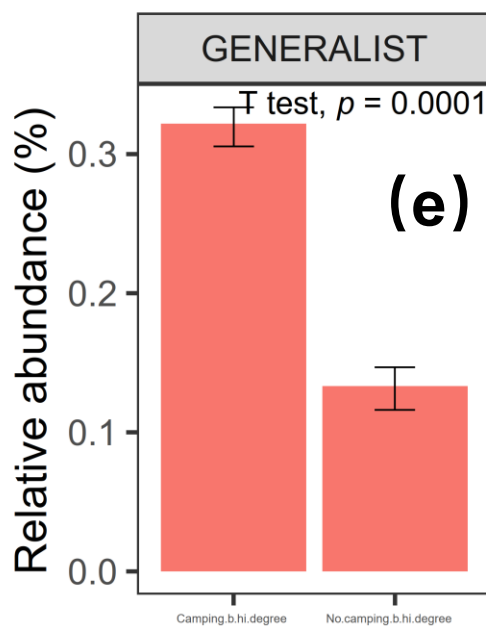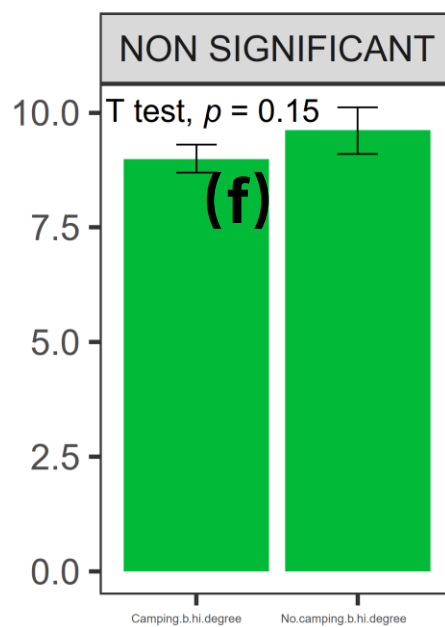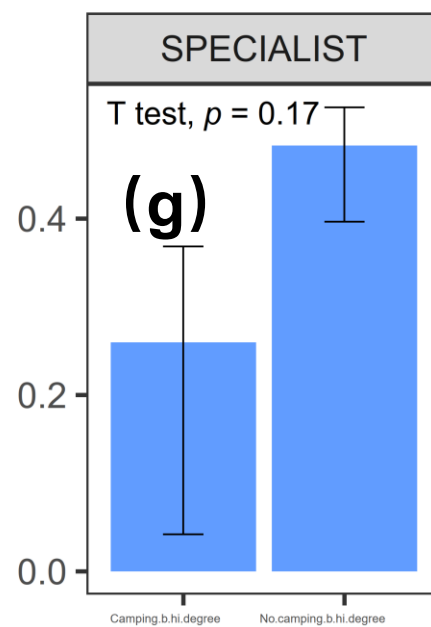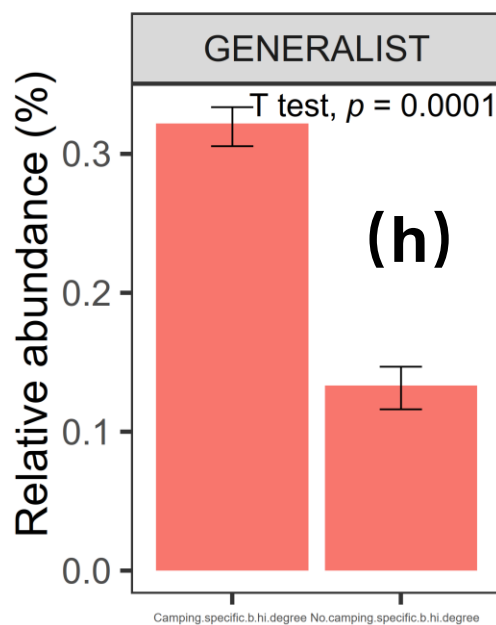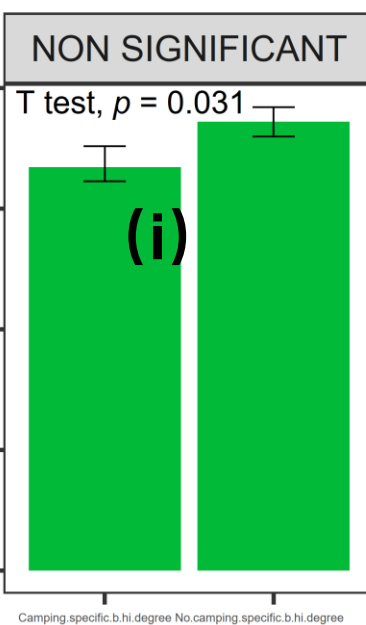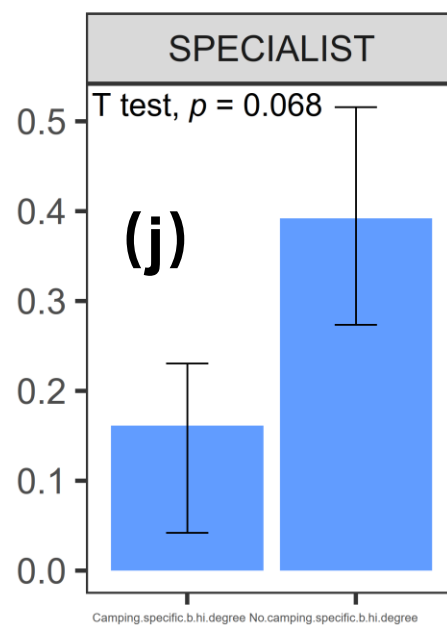

Group

Supplement: Supplemental Information 1 [file peerj-10-14314-s001.zip › Figure S87.pdf]

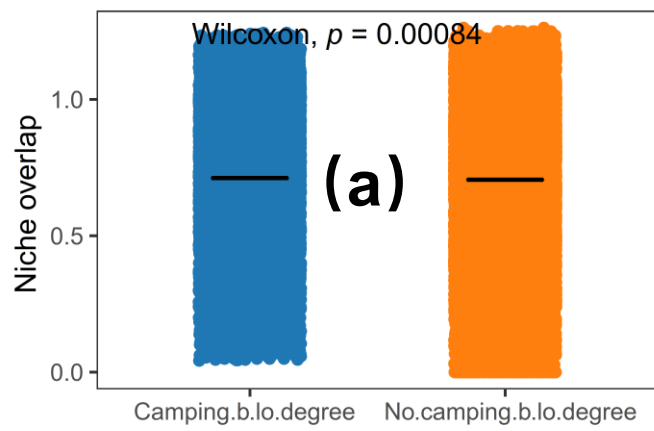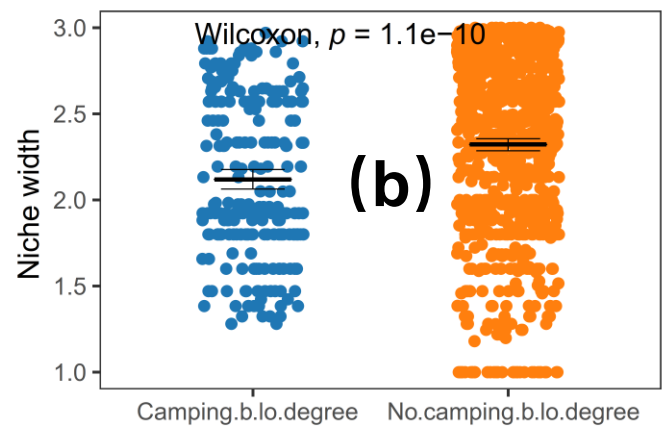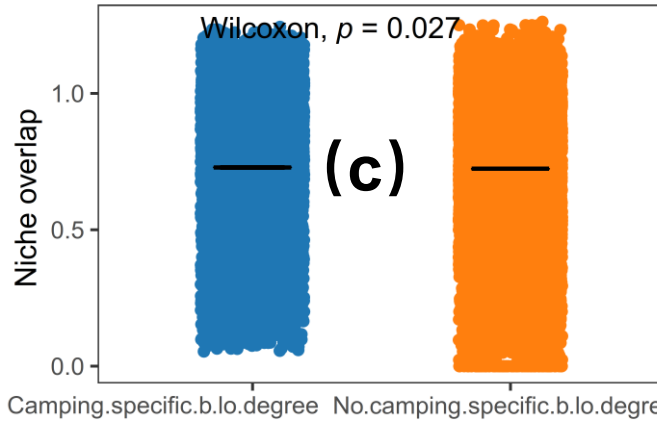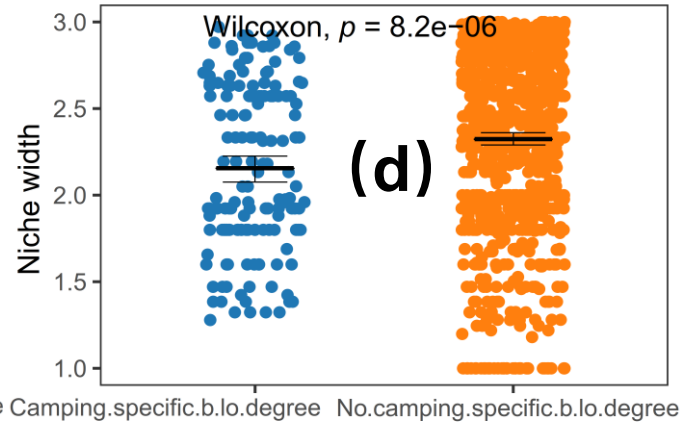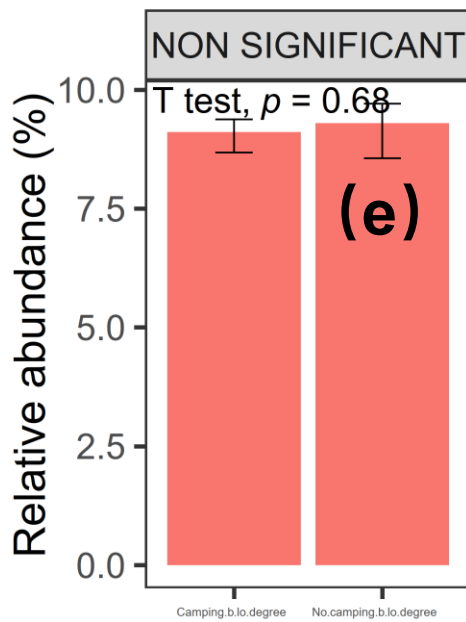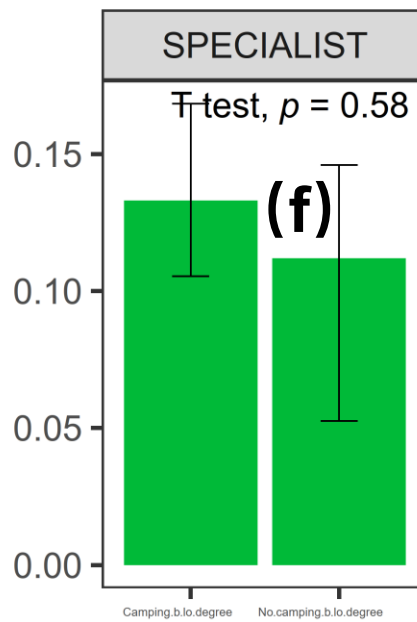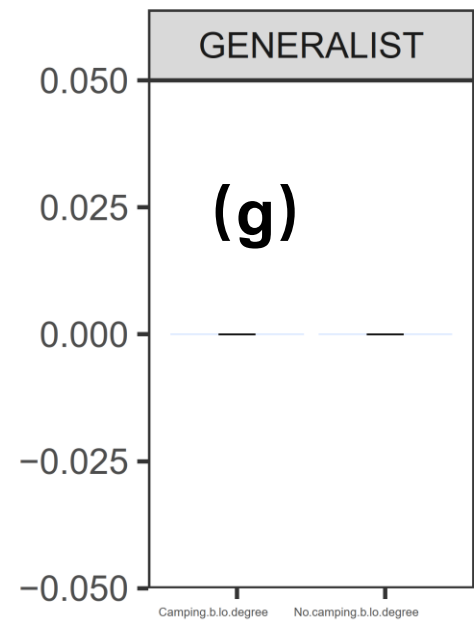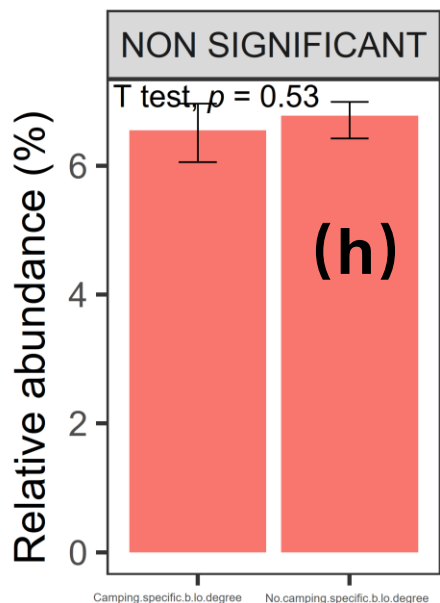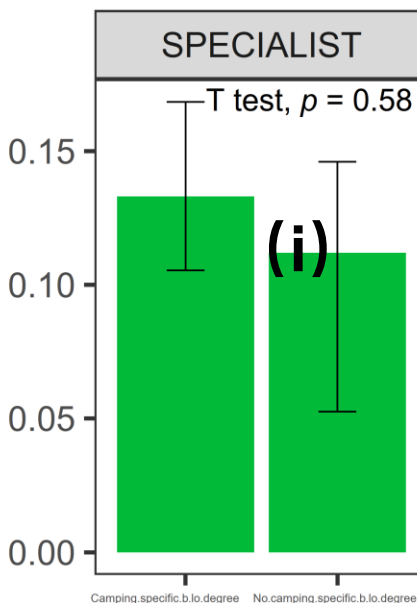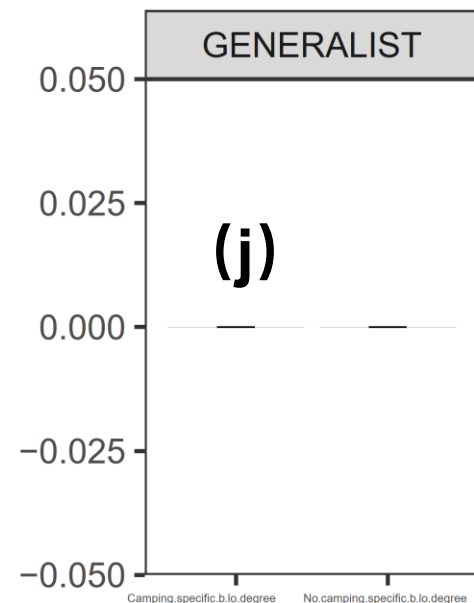

Group

Supplement: Supplemental Information 1 [file peerj-10-14314-s001.zip › Figure S88.pdf]

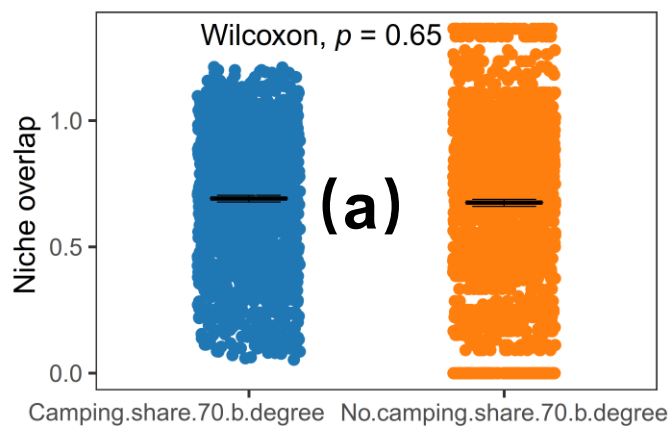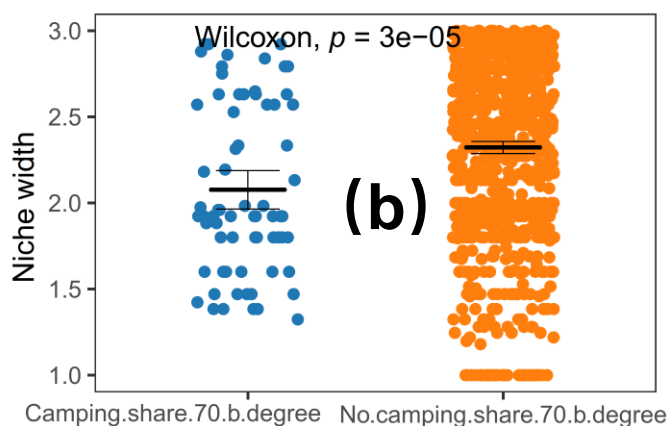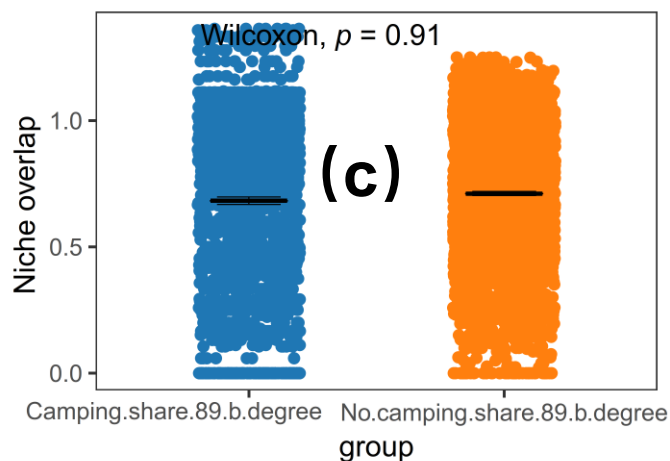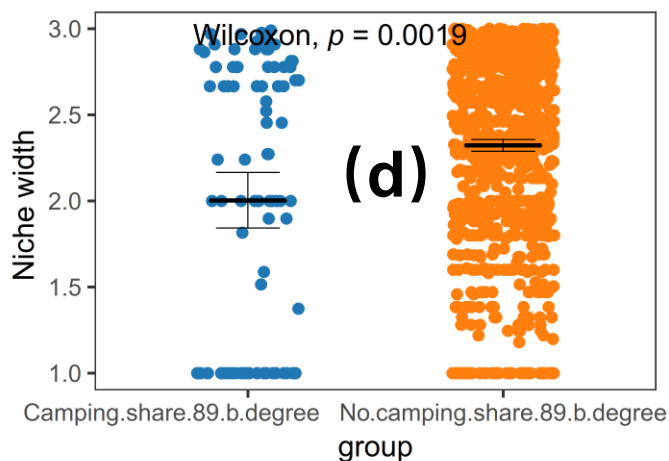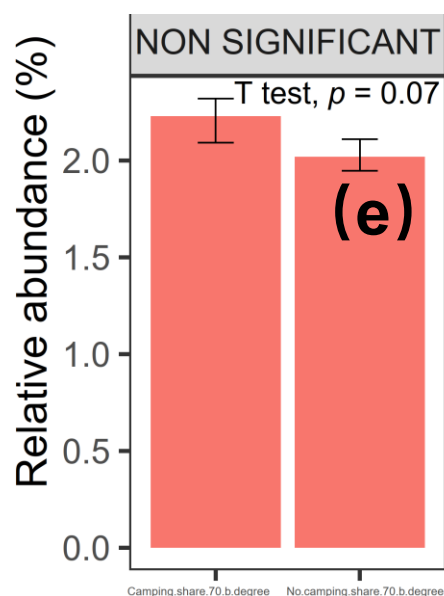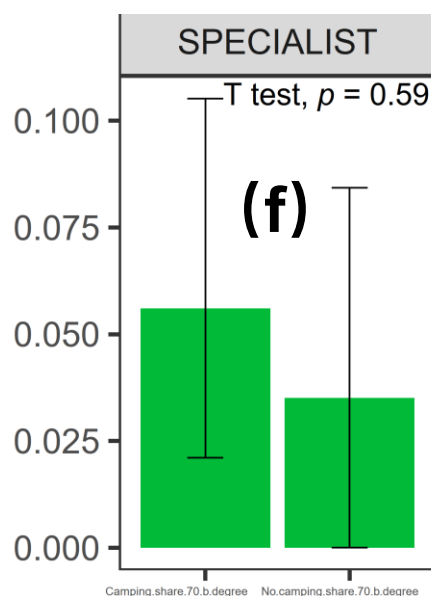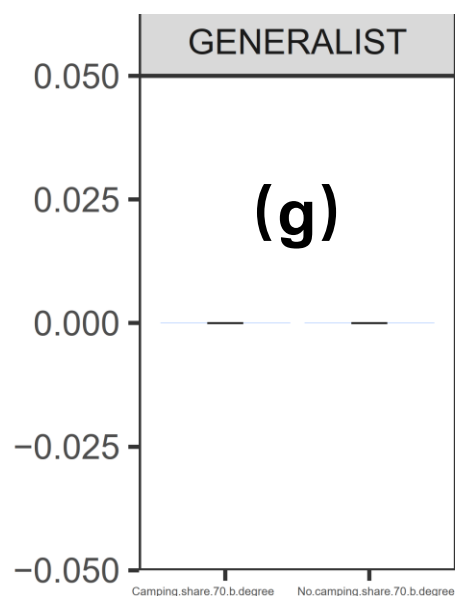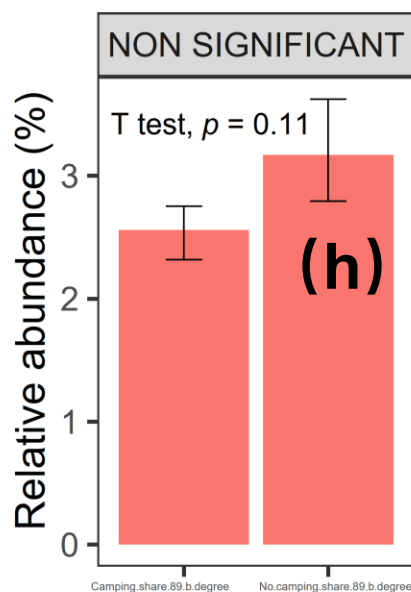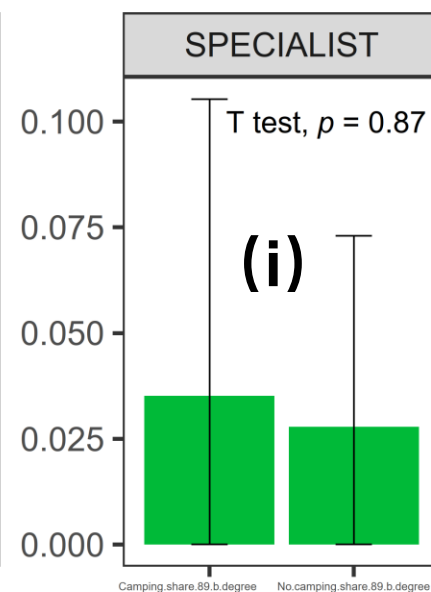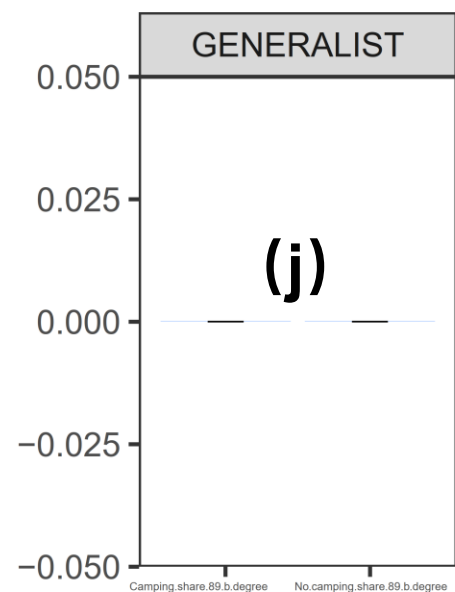

Group

Supplement: Supplemental Information 1 [file peerj-10-14314-s001.zip › Figure S89.pdf]

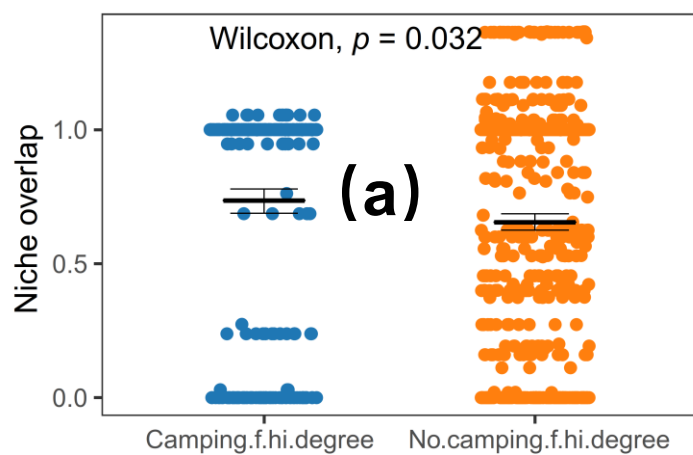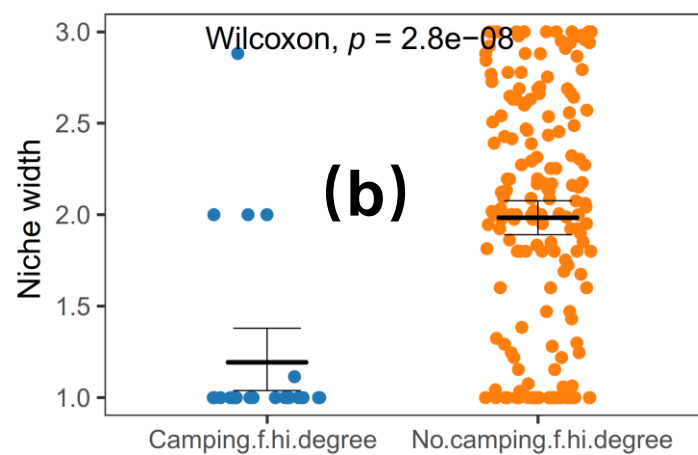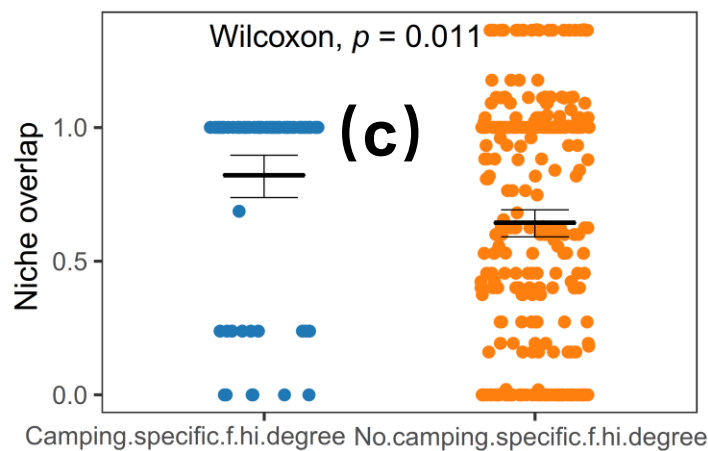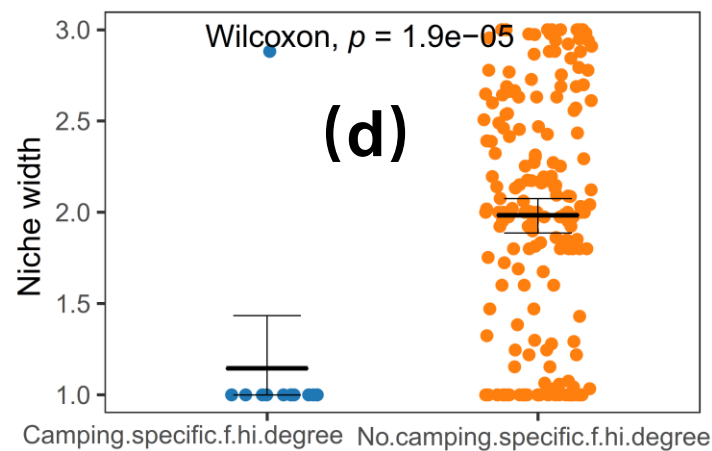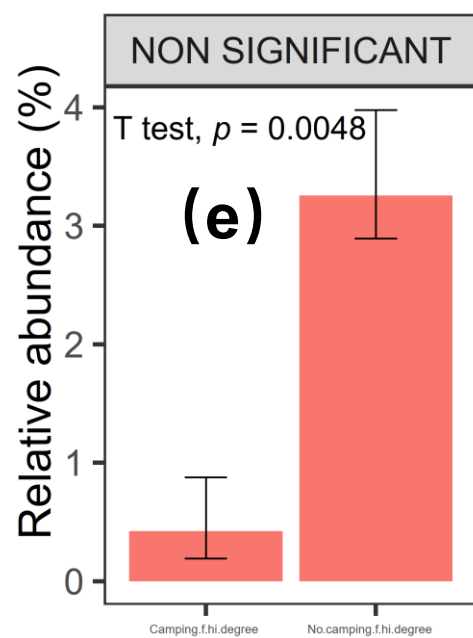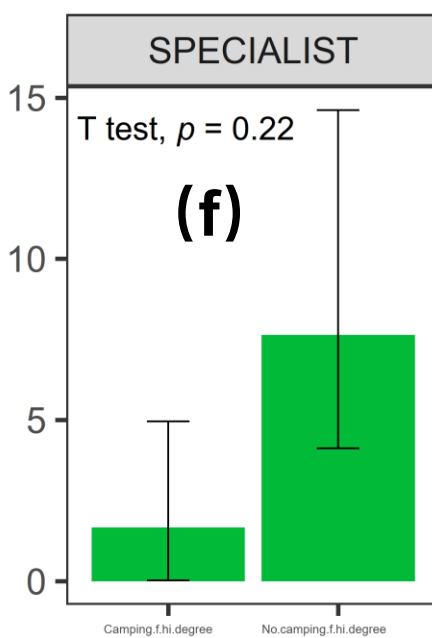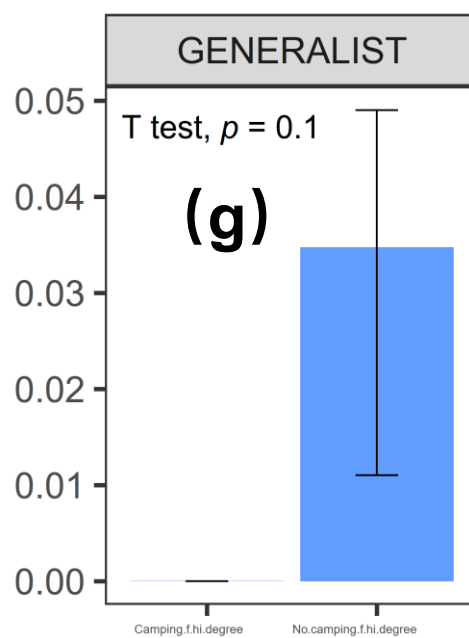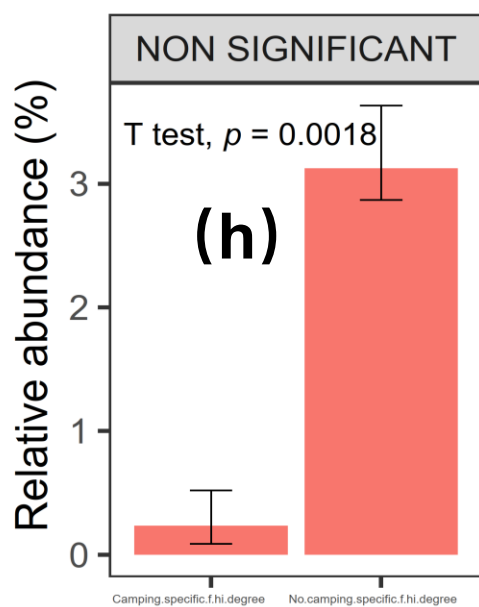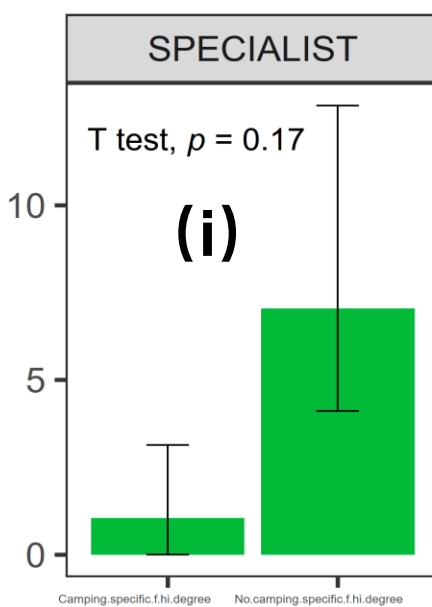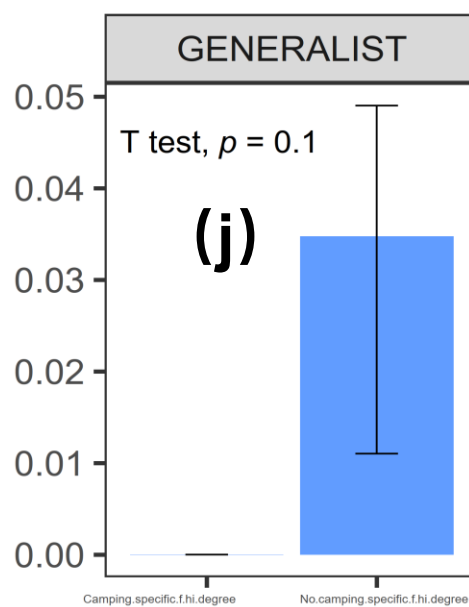

Group

Supplement: Supplemental Information 1 [file peerj-10-14314-s001.zip › Figure S90.pdf]

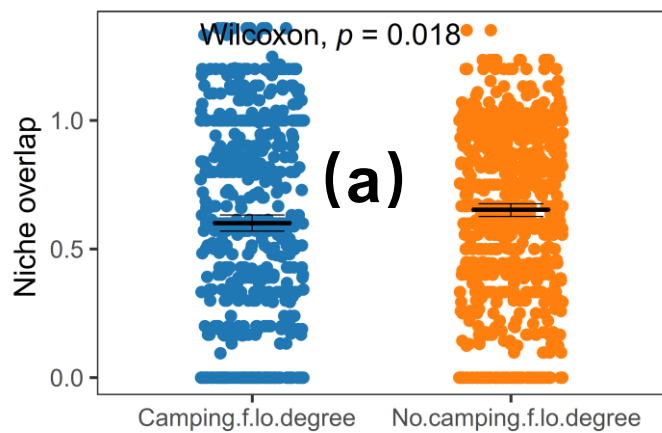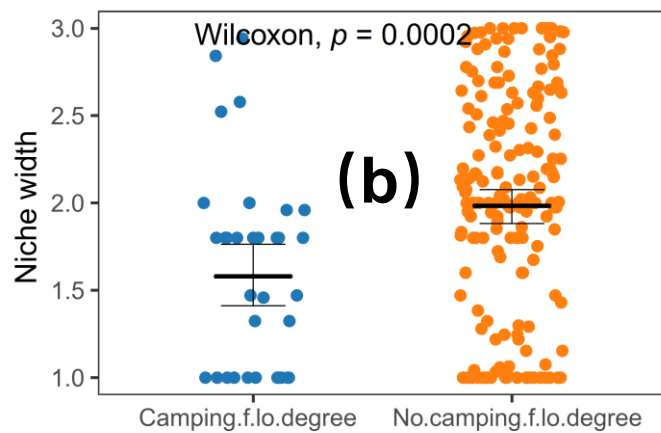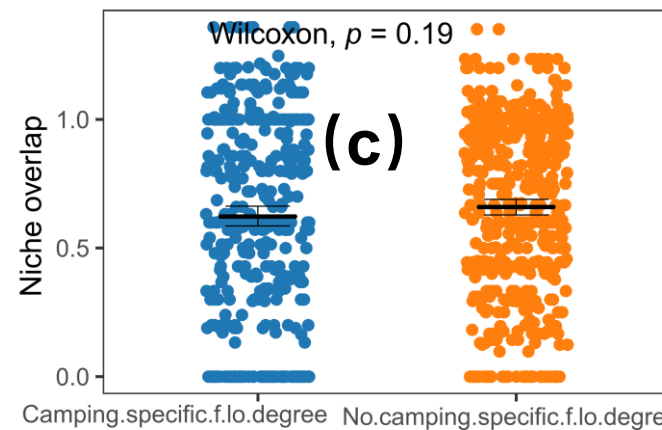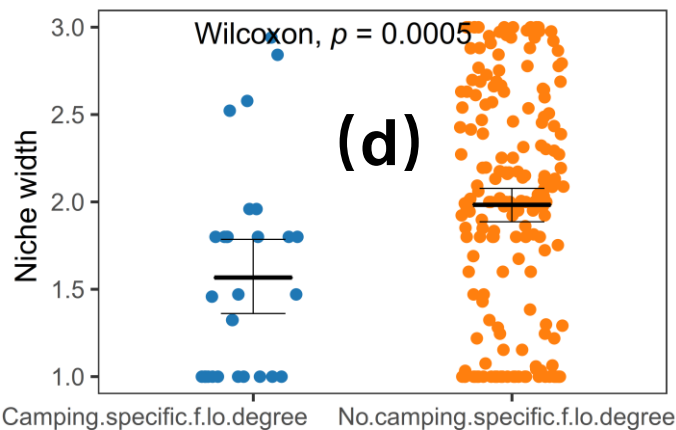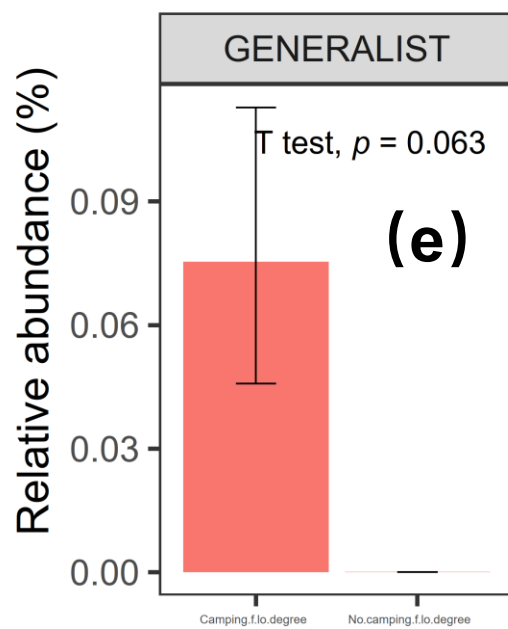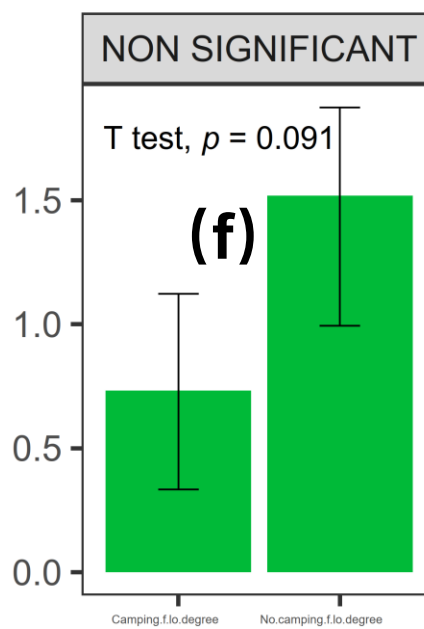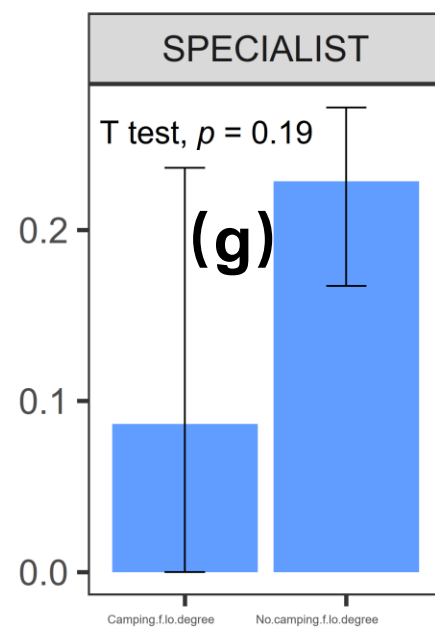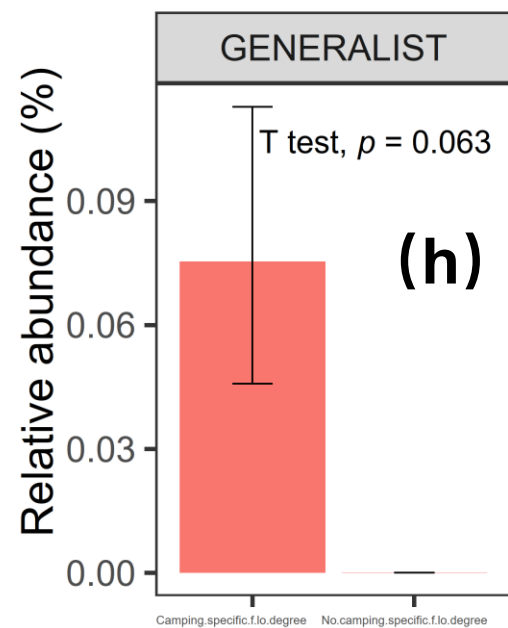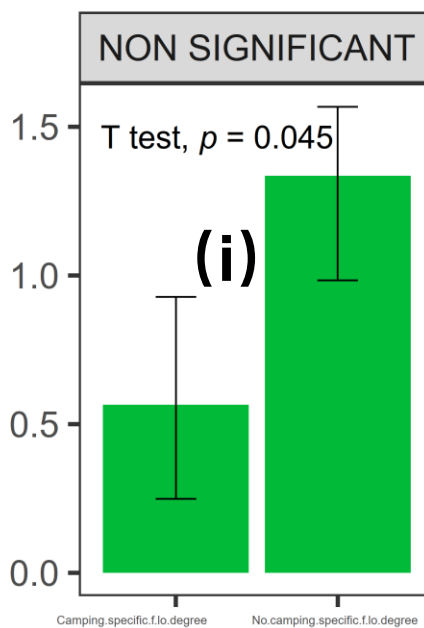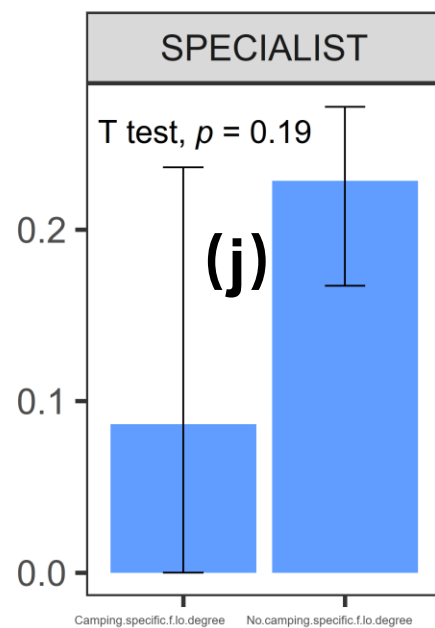

Group

Supplement: Supplemental Information 1 [file peerj-10-14314-s001.zip › Figure S91.pdf]

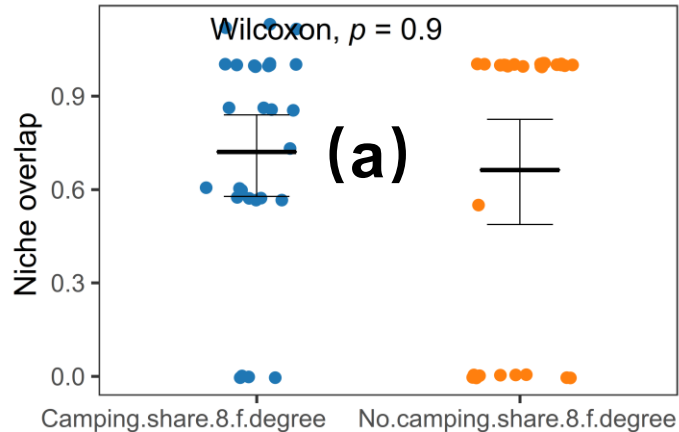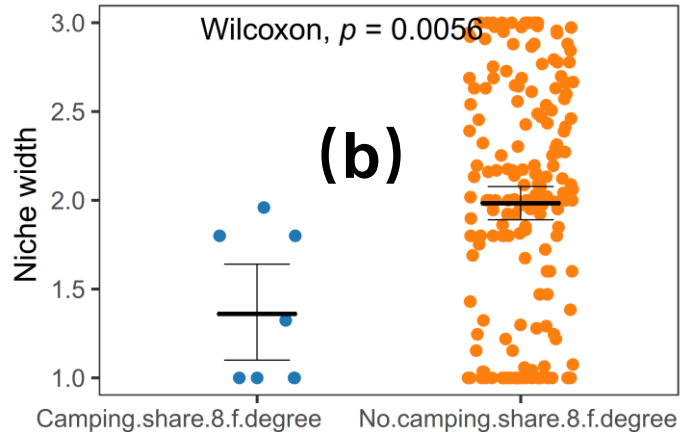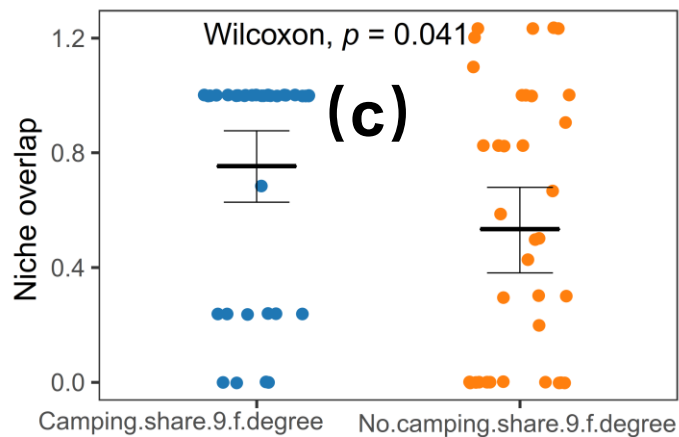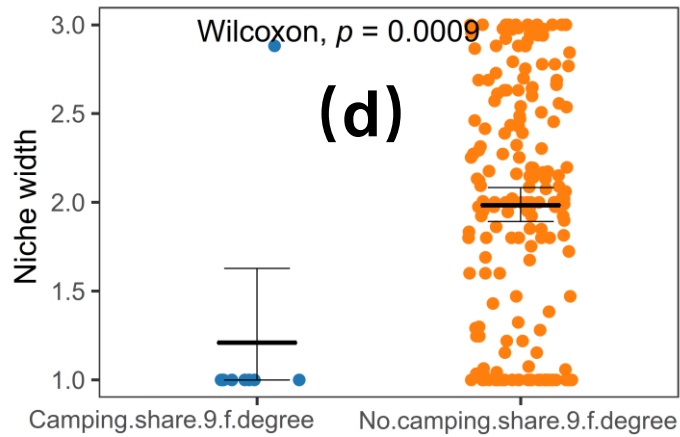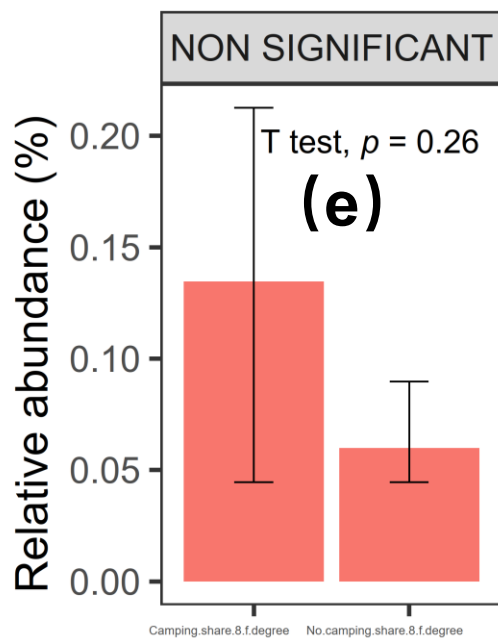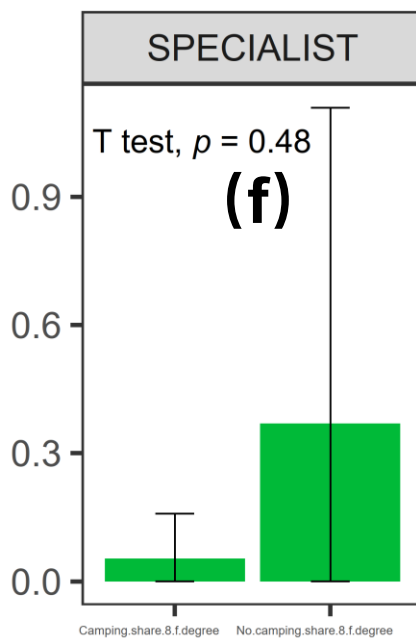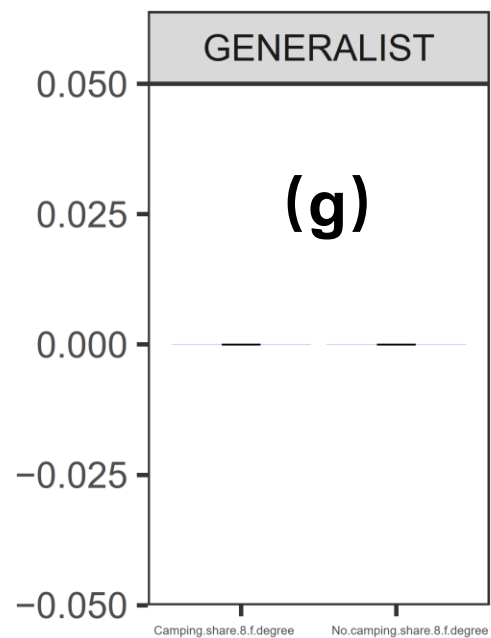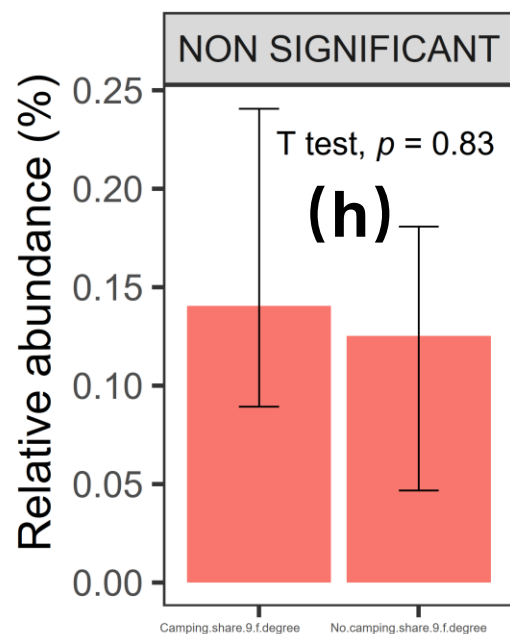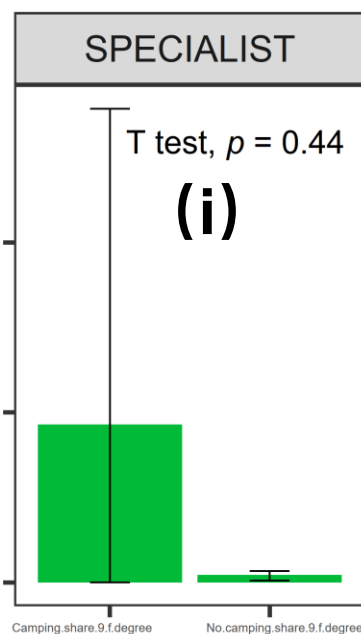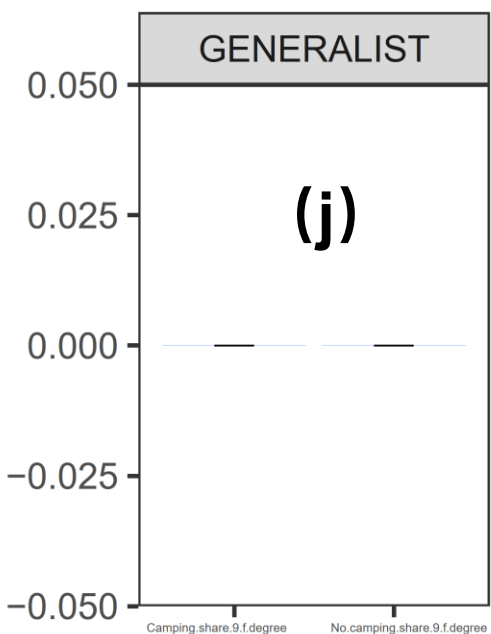

Group

Supplement: Supplemental Information 1 [file peerj-10-14314-s001.zip › Figure S92.pdf]

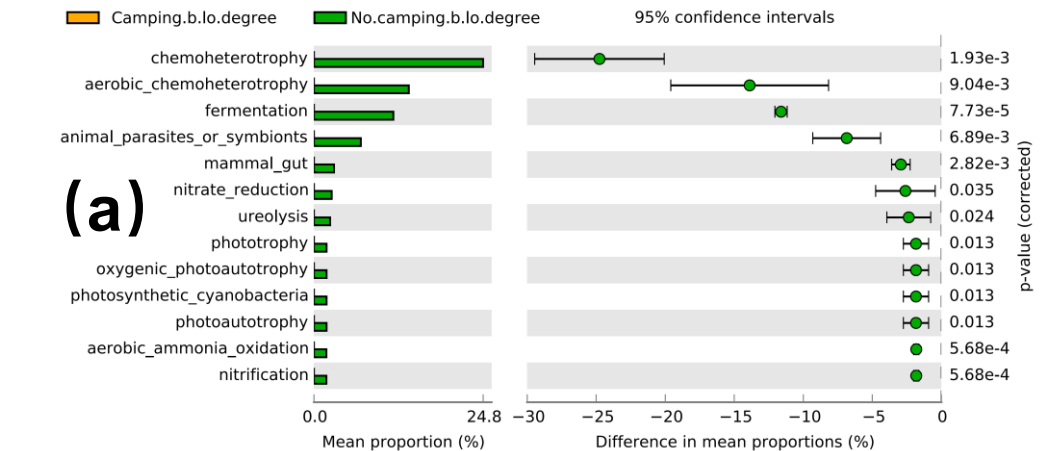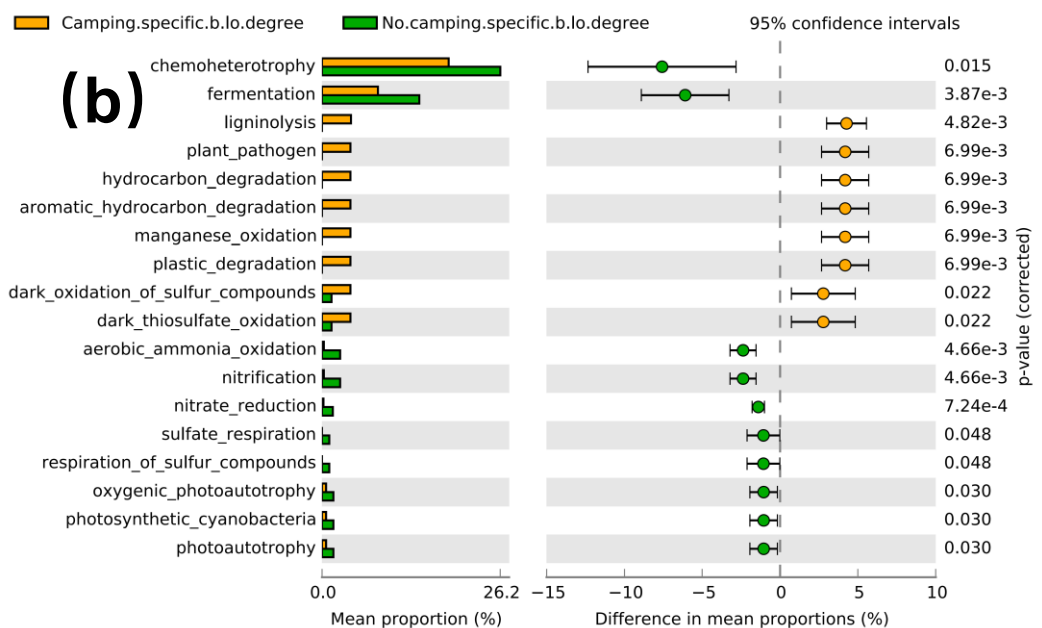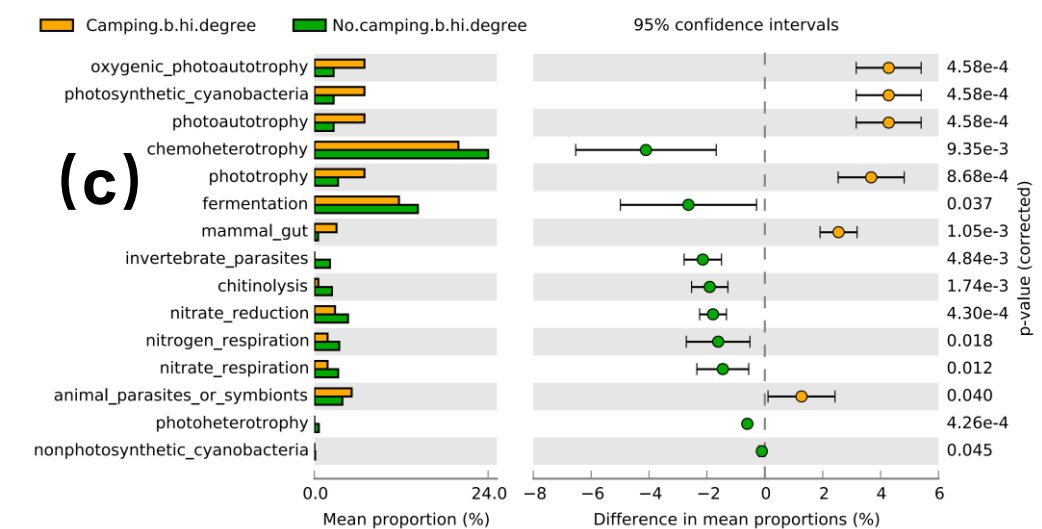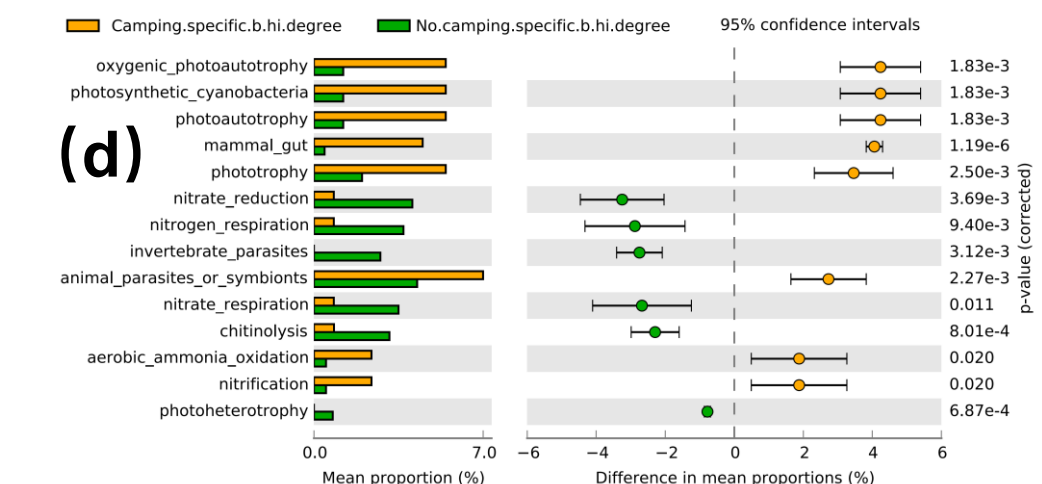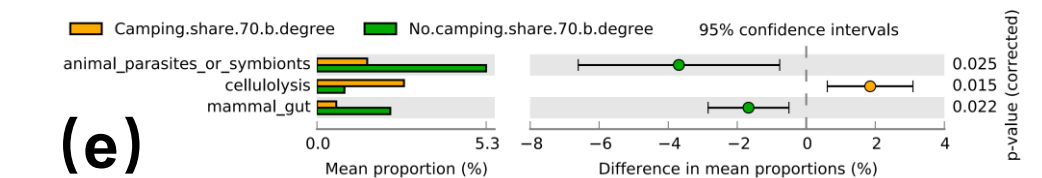

Supplement: Supplemental Information 1 [file peerj-10-14314-s001.zip › Figure S93.pdf]

(a)

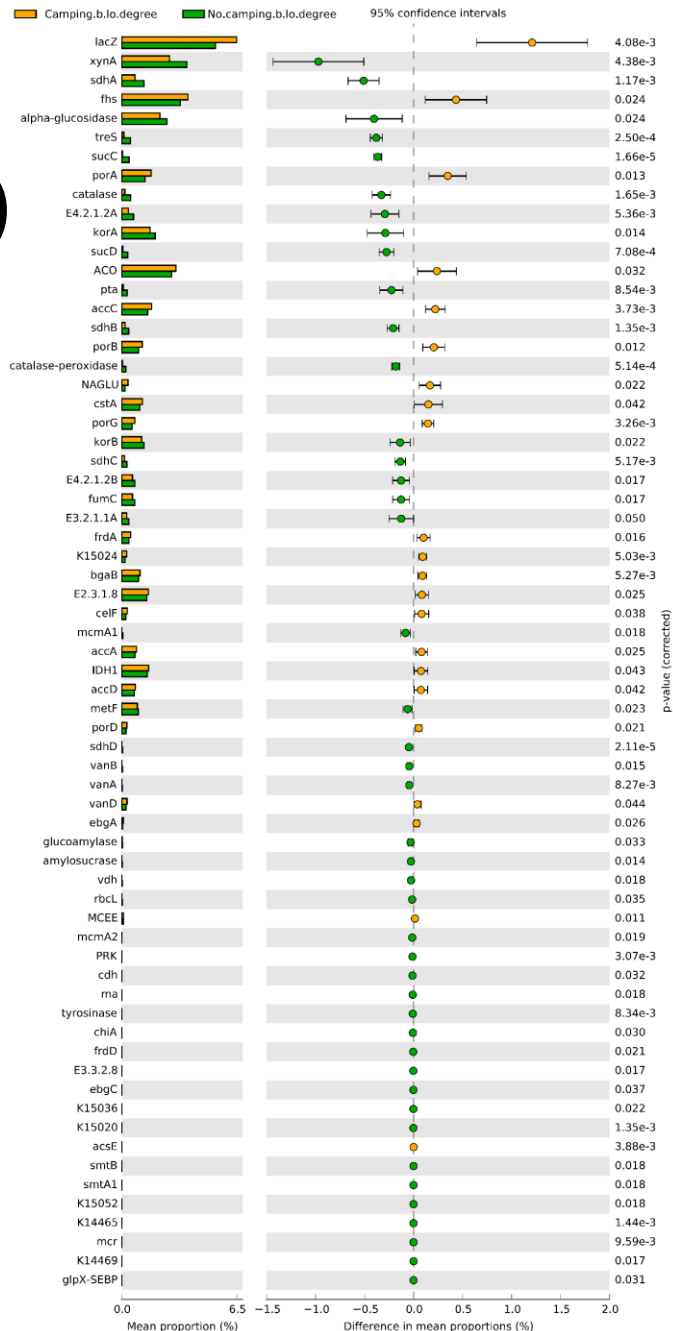

(b)

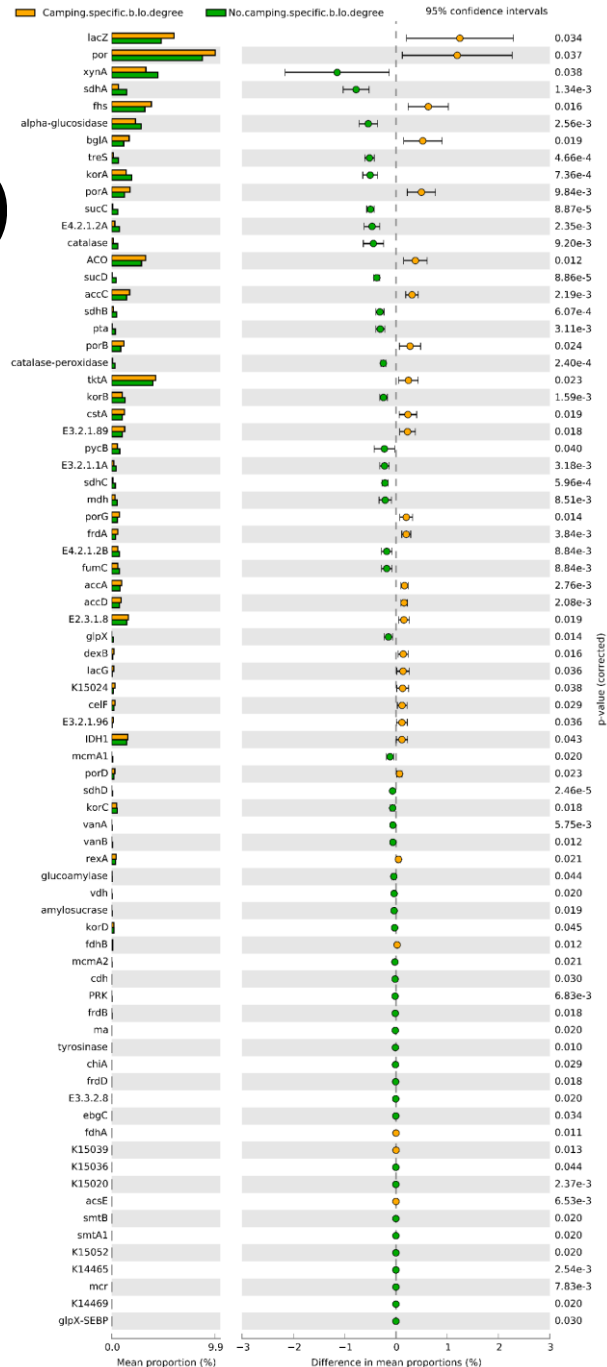

(c)

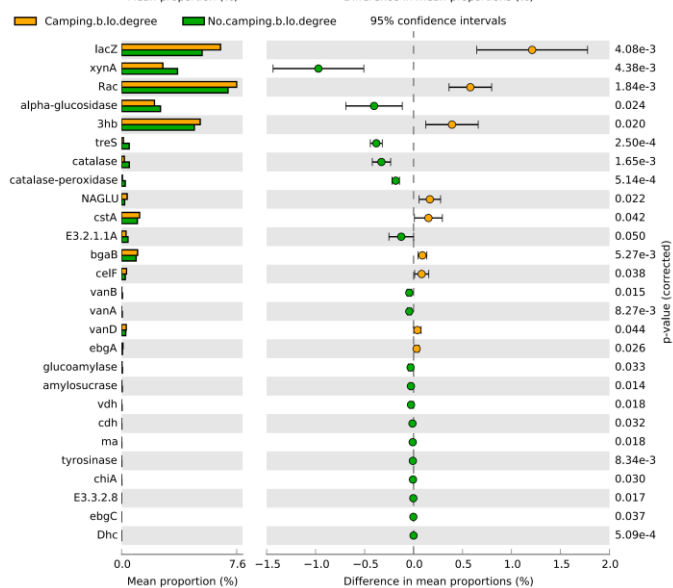

(d)

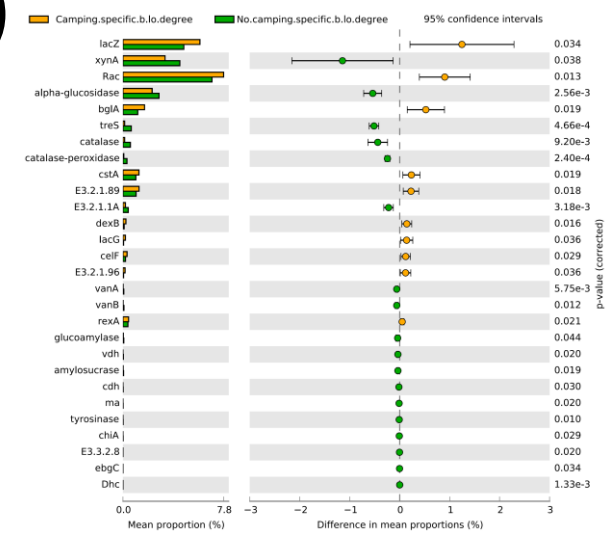

(e)

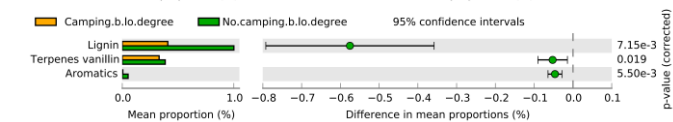

(f)

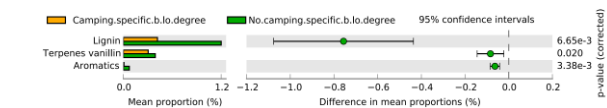

(g)

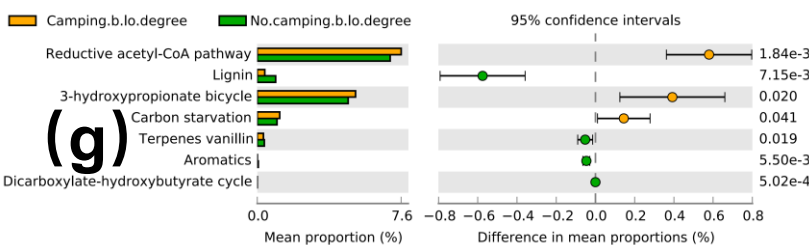

(h)

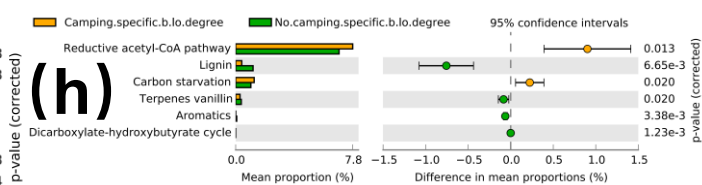

Supplement: Supplemental Information 1 [file peerj-10-14314-s001.zip › Figure S94.pdf]

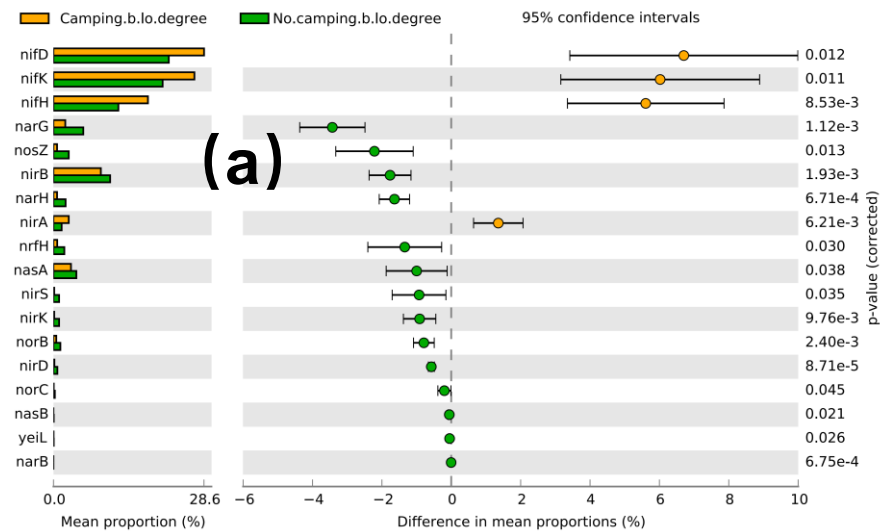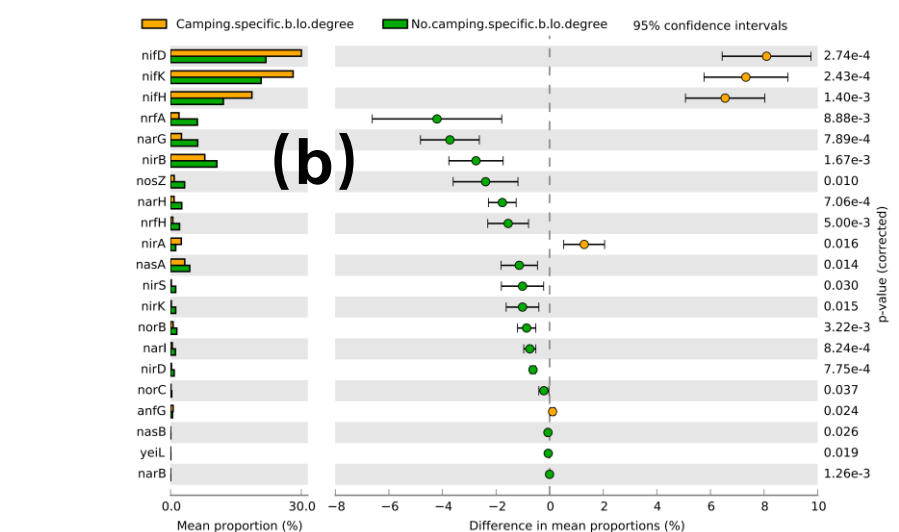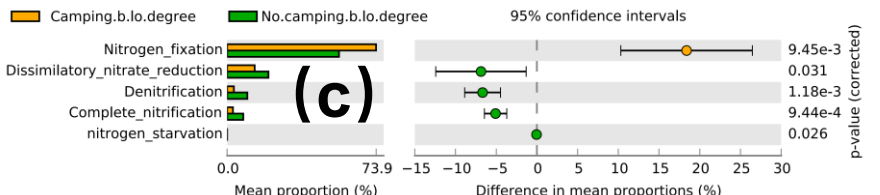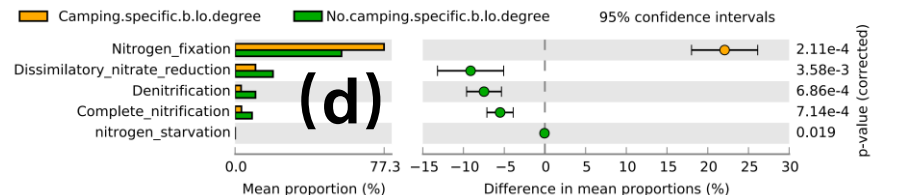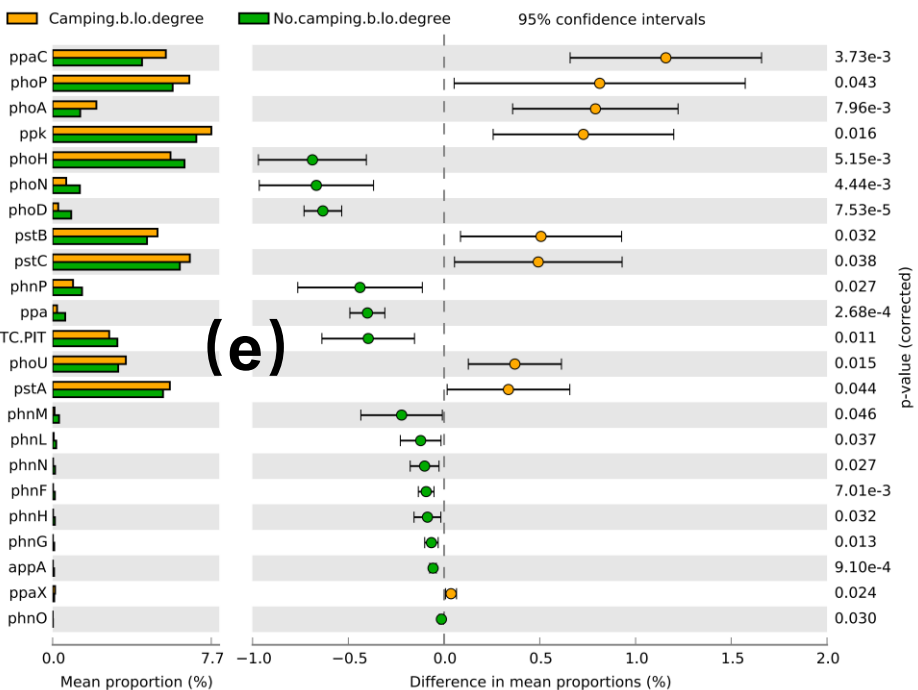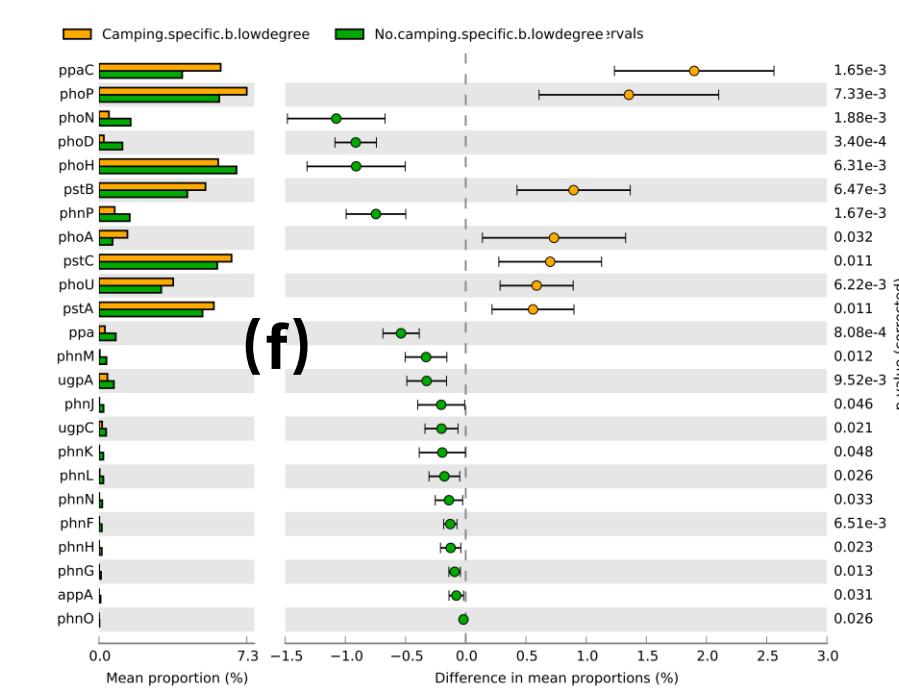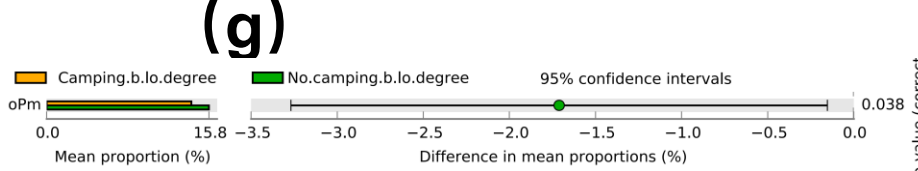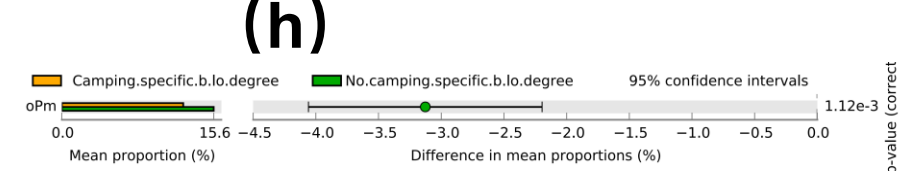

Supplement: Supplemental Information 1 [file peerj-10-14314-s001.zip › Figure S95.pdf]

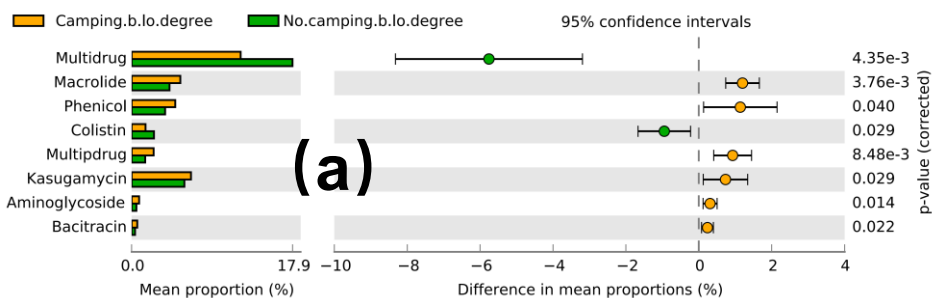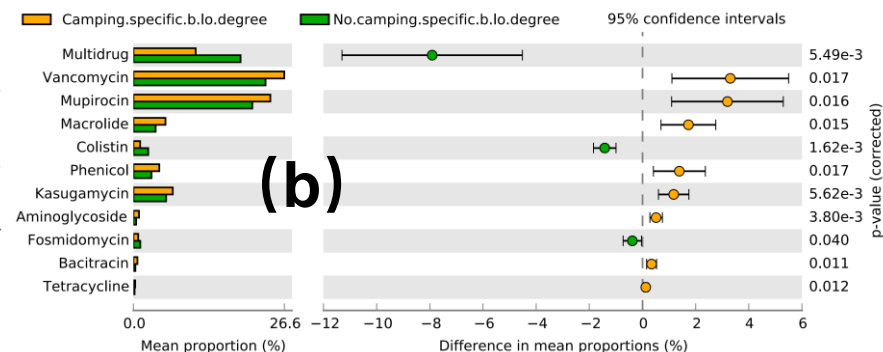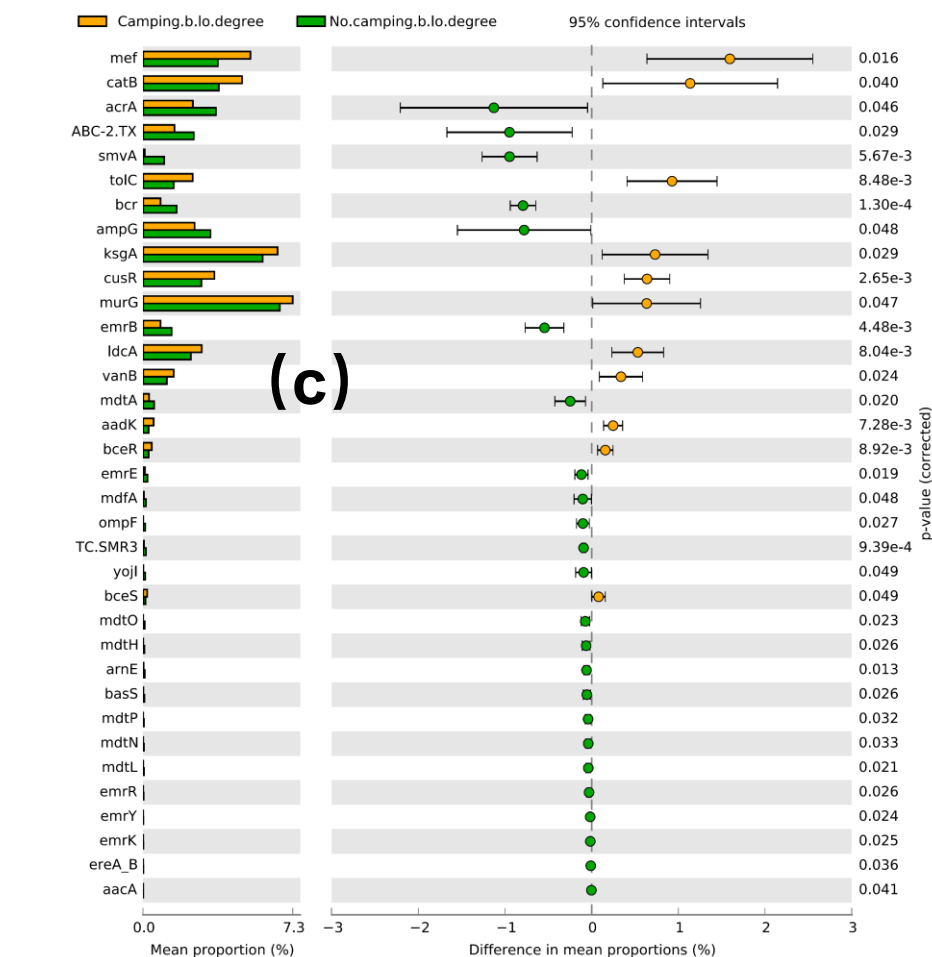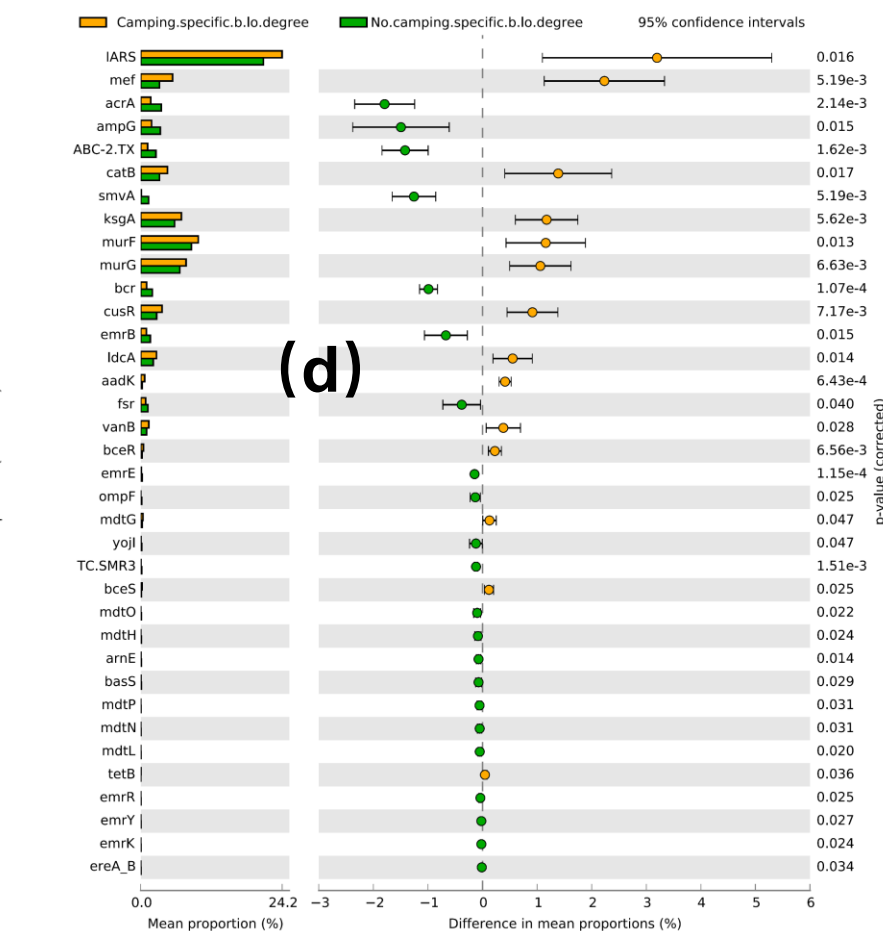

Supplement: Supplemental Information 1 [file peerj-10-14314-s001.zip › Figure S96.pdf]

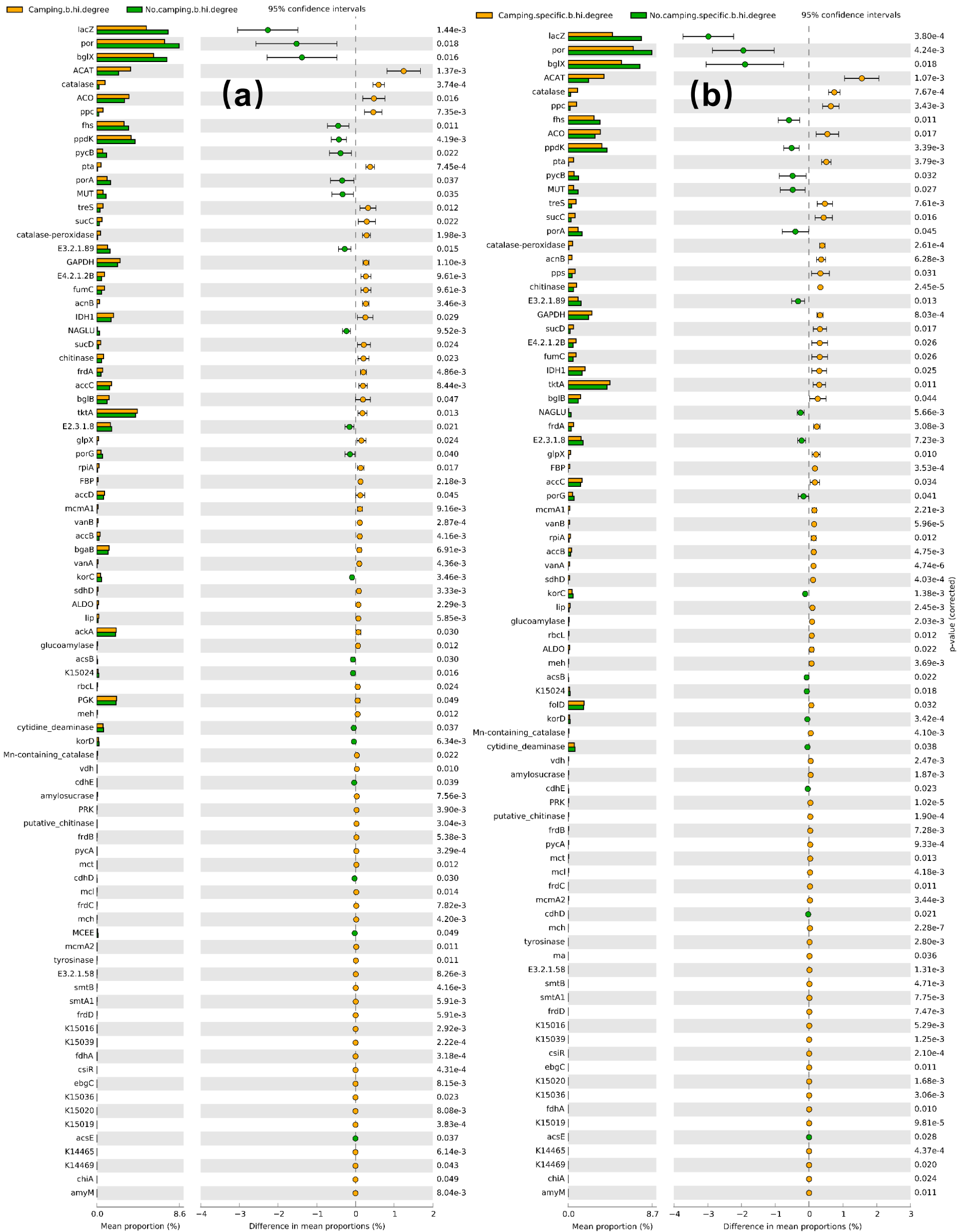

Supplement: Supplemental Information 1 [file peerj-10-14314-s001.zip › Figure S97.pdf]

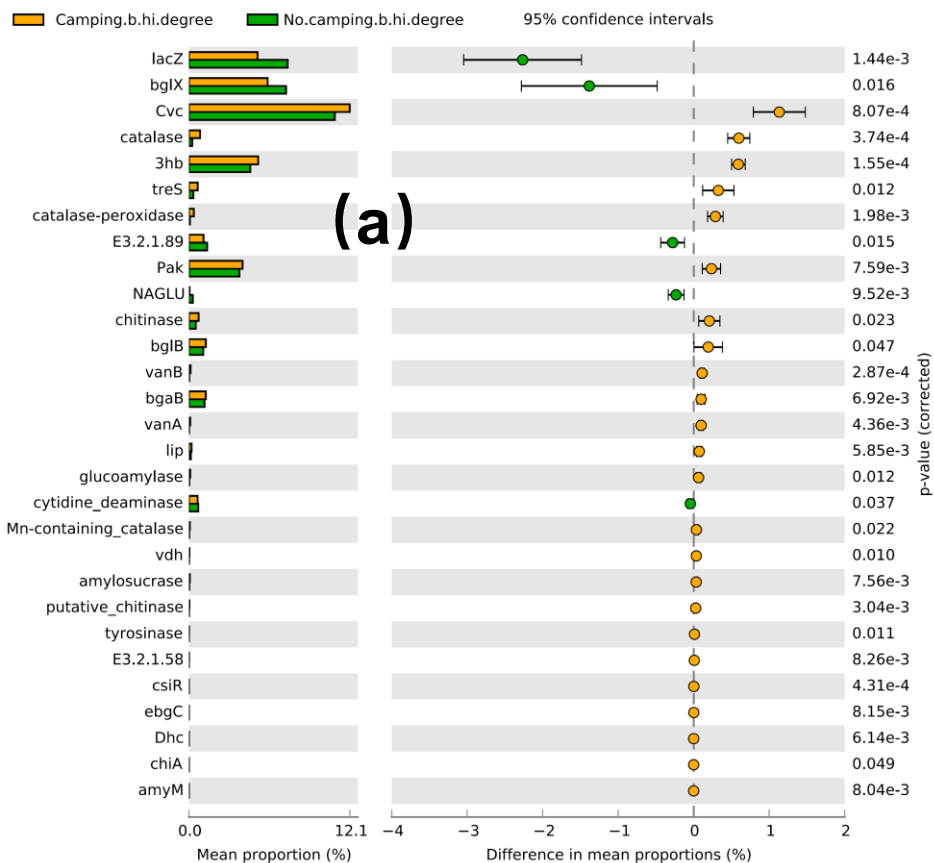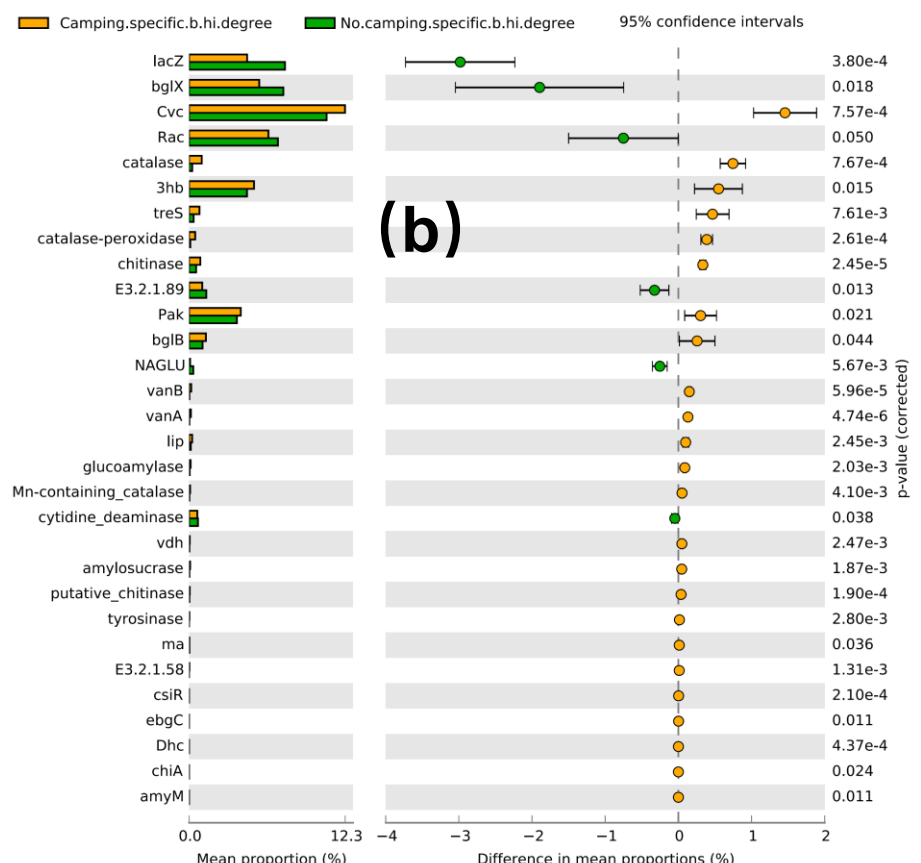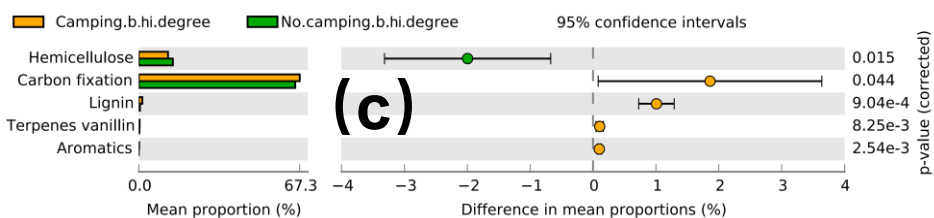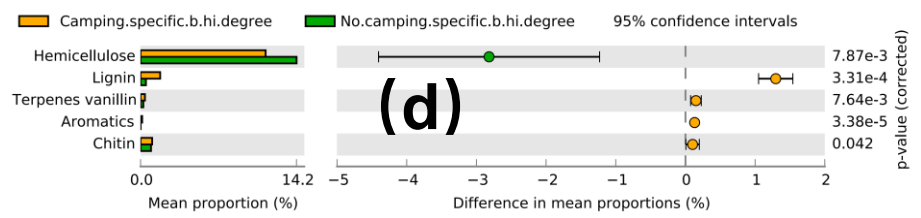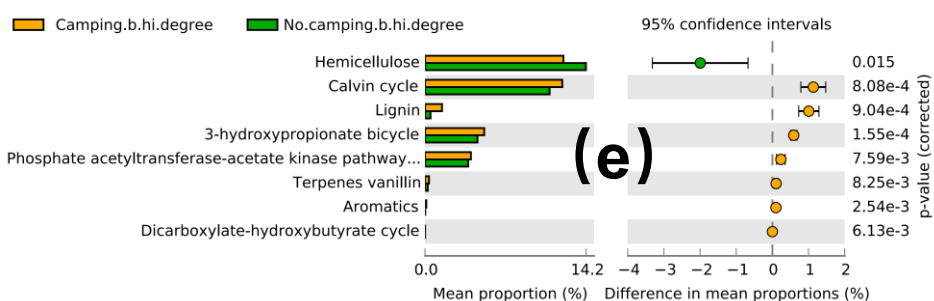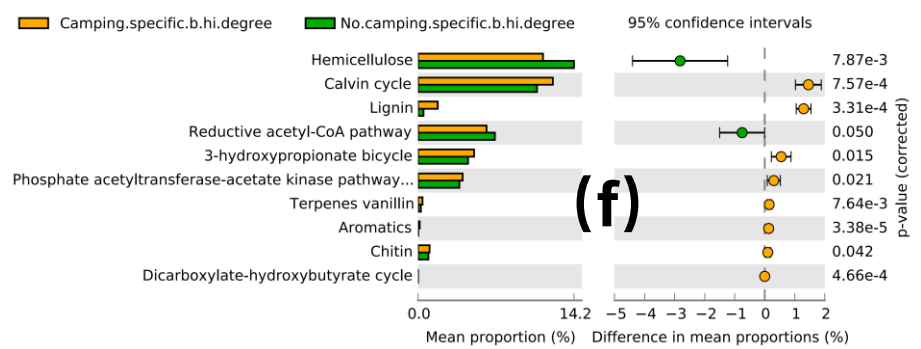

Supplement: Supplemental Information 1 [file peerj-10-14314-s001.zip › Figure S98.pdf]

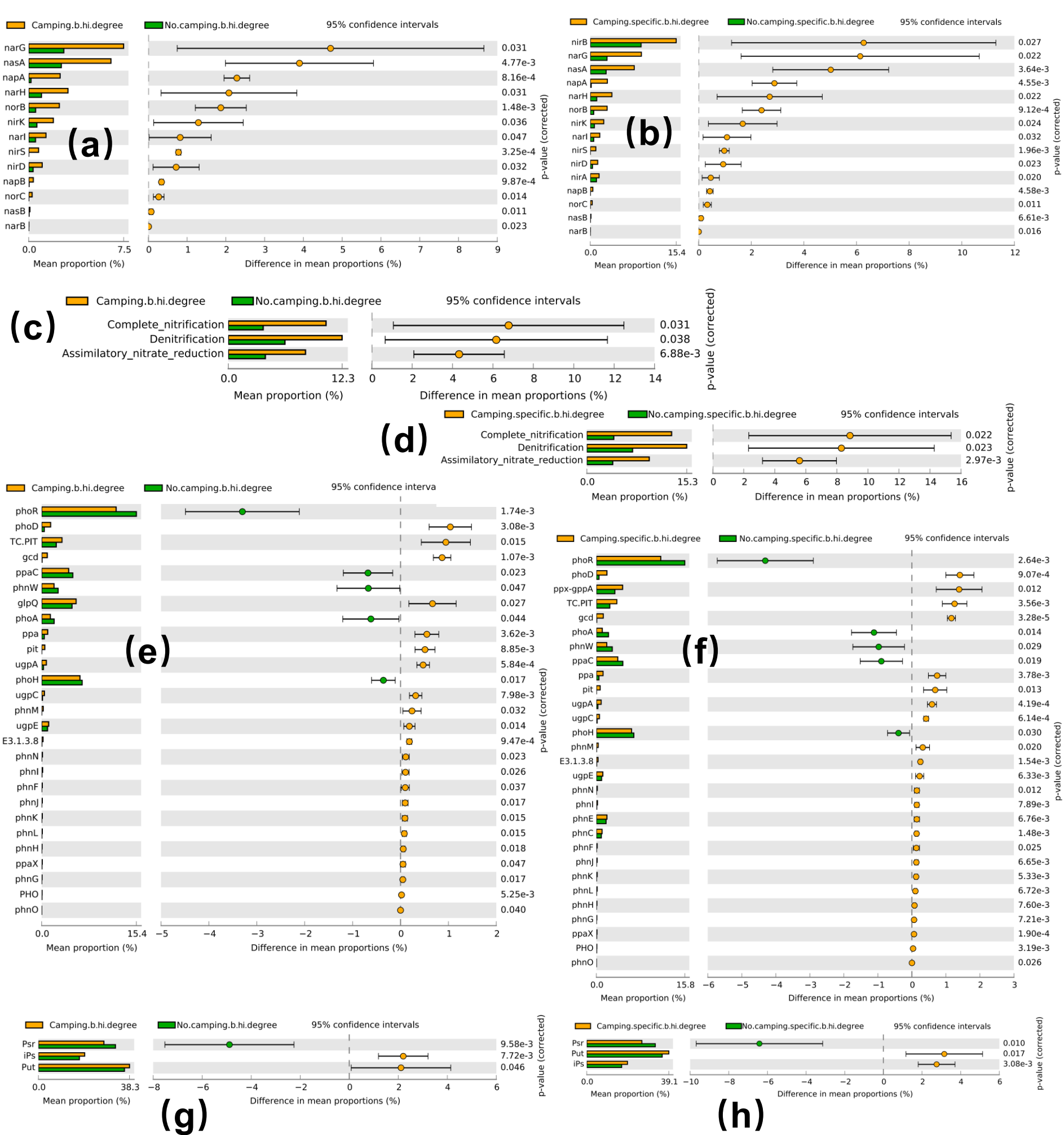

Supplement: Supplemental Information 1 [file peerj-10-14314-s001.zip › Figure S99.pdf]
